# QUINT: Workflow for Quantification and Spatial Analysis of Features in Histological Images From Rodent Brain

**DOI:** 10.3389/fninf.2019.00075

**Published:** 2019-12-03

**Authors:** Sharon C. Yates, Nicolaas E. Groeneboom, Christopher Coello, Stefan F. Lichtenthaler, Peer-Hendrik Kuhn, Hans-Ulrich Demuth, Maike Hartlage-Rübsamen, Steffen Roßner, Trygve Leergaard, Anna Kreshuk, Maja A. Puchades, Jan G. Bjaalie

**Affiliations:** ^1^Neural Systems Laboratory, Institute of Basic Medical Sciences, University of Oslo, Oslo, Norway; ^2^German Center for Neurodegenerative Diseases (DZNE), Munich, Germany; ^3^Neuroproteomics, School of Medicine, Klinikum rechts der Isar, and Institute for Advanced Study, Technical University of Munich, Munich, Germany; ^4^Munich Cluster for Systems Neurology (SyNergy), Munich, Germany; ^5^Institute of Pathology, Technical University of Munich, Munich, Germany; ^6^Department of Molecular Drug Design and Target Validation Fraunhofer Institute for Cell Therapy and Immunology, Halle (Saale), Leipzig, Germany; ^7^Paul Flechsig Institute for Brain Research, University of Leipzig, Leipzig, Germany; ^8^European Molecular Biology Laboratory, Heidelberg, Germany

**Keywords:** rodent brain analysis, Alzheimer’s disease, quantification, workflow, APP—amyloid precursor protein, beta-amyloid

## Abstract

Transgenic animal models are invaluable research tools for elucidating the pathways and mechanisms involved in the development of neurodegenerative diseases. Mechanistic clues can be revealed by applying labelling techniques such as immunohistochemistry or *in situ* hybridisation to brain tissue sections. Precision in both assigning anatomical location to the sections and quantifying labelled features is crucial for output validity, with a stereological approach or image-based feature extraction typically used. However, both approaches are restricted by the need to manually delineate anatomical regions. To circumvent this limitation, we present the QUINT workflow for quantification and spatial analysis of labelling in series of rodent brain section images based on available 3D reference atlases. The workflow is semi-automated, combining three open source software that can be operated without scripting knowledge, making it accessible to most researchers. As an example, a brain region-specific quantification of amyloid plaques across whole transgenic Tg2576 mouse brain series, immunohistochemically labelled for three amyloid-related antigens is demonstrated. First, the whole brain image series were registered to the Allen Mouse Brain Atlas to produce customised atlas maps adapted to match the cutting plan and proportions of the sections (*QuickNII* software). Second, the labelling was segmented from the original images by the Random Forest Algorithm for supervised classification (*ilastik* software). Finally, the segmented images and atlas maps were used to generate plaque quantifications for each region in the reference atlas (*Nutil software*). The method yielded comparable results to manual delineations and to the output of a stereological method. While the use case demonstrates the QUINT workflow for quantification of amyloid plaques only, the workflow is suited to all mouse or rat brain series with labelling that is visually distinct from the background, for example for the quantification of cells or labelled proteins.

## Introduction

Transgenic rodent models are useful tools in the study of neurodegenerative disorders, providing clues to the origins and mechanisms of the protein aggregates that accumulate and harm neurons and synapses in these conditions (Dawson et al., 2018). A common study approach is to section the brains and apply immunohistochemical or other histological techniques to reveal features that can be explored by microscopy. Qualitative assessments of such features can reveal vulnerable brain regions, while understanding the connectivity of affected regions may provide insight into disease mechanisms (Thal et al., 2002; Hurtado et al., 2010). The ability to accurately assign anatomical location to the data is of crucial importance to the validity of the conclusions drawn, and is a limiting factor in these studies. Present resources for assigning anatomical location to whole brain rodent data are not easily applicable to 2D histological series, especially if cutting angles deviate even marginally from the coronal, sagittal or horizontal planes. Even with diligent sectioning, small deviations of a few degrees are common. Our recent registration tool, *QuickNII*, allows users to perform that correction (Puchades et al., 2019). For users with coding expertise, other tools for registration of image series to reference atlases are also available (Kopec et al., 2011; Fürth et al., 2018; Xiong et al., 2018). Furthermore, combining datasets from different sources or comparison of data from different animal models is difficult unless the data are linked to the same atlas reference system (Simmons and Swanson, 2009; Kim et al., 2017; Bjerke et al., 2018).

The gold standard for quantification of features in 2D image series is stereological analysis applied to anatomical regions that have been manually delineated by an expert in the field (Schmitz and Hof, 2005). However, in practical terms, this method is difficult to apply optimally due to a shortage of anatomical expertise, the significant numbers of sections for analysis, and limited availability of time. Large scale projects and multi-centre collaborations would benefit from the automation of both the extraction and spatial analysis steps. The introduction of the machine learning concept has opened up possibilities for semi-automated extraction of features based on supervised machine learning algorithms (Berg et al., 2019). Furthermore, the new generation of three-dimensional digital brain atlases developed for murine brains (Lein et al., 2007; Hawrylycz et al., 2011; Oh et al., 2014; Papp et al., 2014; Kjonigsen et al., 2015) serve as spatial frameworks for data sharing and integration (Boline et al., 2008; Zaslavsky et al., 2014), while also providing possibilities for automation of spatial analysis.

To this end, we have developed the QUINT workflow based on image analysis using a series of neuroinformatic tools. The workflow entails three steps. In the first step, images are registered to a 3D reference atlas. This step utilises a three-dimensional brain atlasing tool, *QuickNII* (Puchades et al., 2019) that supports arbitrary cutting angles, and is used to generate atlas maps that are customised specifically to match each section. In the second step, segmentation of distinct features such as labelled cells or aggregates is performed with *ilastik*. The *ilastik* software benefits from a supervised machine learning approach (Berg et al., 2019) allowing a combination of many parameters for segmentation as is demonstrated in the use cases here. However, the workflow is compatible with segmentations produced by other means, such as *NIH ImageJ* (Schneider et al., 2012), or with another image analysis tool provided that it supports segmented image export. As illustrated by Pallast et al. (2019), different types of features may require different segmentation tools. In the third step, the *Nutil* software draws on the atlas maps and segmentations to quantify segmented objects in relation to the delineated brain regions contained in the atlas. *Nutil* also extracts the xyz position of the segmented objects for viewing in reference atlas space. As an example, we present the quantification of human amyloid precursor protein (hAPP) and β-amyloid deposits across a whole mouse brain series immunohistochemically labelled For the human APP N-Terminus (rat monoclonal antibody; Höfling et al., 2016), Aβ (4G8 mouse monoclonal antibody) and pyro-glutamate modified Aβ [pE-Aβ; J8 mouse monoclonal antibody (Hartlage-Rübsamen et al., 2018)]. The results are validated by comparing the workflow output with ground truth data manually segmented with the *NIH ImageJ* tool (Schneider et al., 2012), and by comparing to stereological counts with the MBF Bioscience *Stereo Investigator* Area Fraction Fractionator probe. A second example is shared to demonstrate the use of the workflow for quantification of another type of labelling (parvalbumin positive cells in an Allen Mouse Brain series).

## Materials and Methods

**Figure 1:**
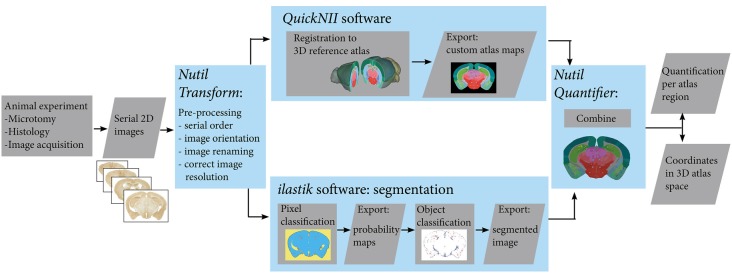
Workflow for automated quantification and spatial analysis. Diagram showing key steps of the workflow (blue frames). After sectioning and labelling, brain sections are digitalised. Serial section images are pre-processed, and then registered to a 3-D reference atlas space using the *QuickNII* tool. The same images are segmented using the *ilastik* tool. Exported custom atlas maps and segmented images are then combined in the *Nutil* tool in order to extract quantification of objects in atlas brain regions as well as 3D coordinates of the objects.

The workflow for serial brain section image analysis comprises several parts (Figure 1): namely, image pre-processing (*Nutil* using the *Transform feature*); registration of images to a reference atlas (*QuickNII*); segmentation of labelled features (*ilastik*); and quantification of features per atlas region (*Nutil* using the *Quantifier feature*).

### Use Case Material: Animal, Immunohistochemical Labelling and Image Acquisition

An 18-month-old male Tg2576 mouse (Hsiao et al., 1996) mimicking the amyloid pathology of Alzheimer’s disease supplied the material for the first use case (plaque quantification). This study was carried out in accordance with the principles of the Basel Declaration and recommendations of the ARRIVE guidelines, National Centre for the Replacement, Refinement and Reduction of Animals in Research, UK. The protocol used was approved by the responsible authority Landesdirektion Sachsen, Germany, license number T28/16. The mouse was sacrificed by CO_2_ inhalation and the brain was fixed using the transcardial perfusion fixation method. First, the brain was perfused with 30 mL of PBS, followed by 30 mL of 4% paraformaldehyde (PFA) solution and post-fixed at 4°C overnight. The brain was cryoprotected by immersion in 30% sucrose for 3 days and sectioned using a freezing microtome in 30 μm thick coronal sections. Every 4th section (60 sections) was used for immunolabelling of hAPP using the species-specific monoclonal rat antibody 1D1 (dilution 1:2; Höfling et al., 2016). Neighbouring sections with the same sampling frequency were labelled with the 4G8 antibody detecting pan-Aβ (BioLegend RRID:AB_2734548, 1:8,000) and with the J8 antibody detecting pE-Aβ (1:2,000; Hartlage-Rübsamen et al., 2018). After incubation with biotinylated secondary antibodies (1:1,000; Dianova; Hamburg, Germany) in TBS with 2% bovine serum albumin for 60 min at room temperature, the ABC method was applied, which comprised incubation with complexed streptavidin–horseradish peroxidase (1:1,000; Sigma; Deisenhofen, Germany). Incubations were separated by washing steps (3-times, 5 min). Binding of peroxidase was visualised by incubation with 4 mg 3,3′-diaminobenzidine and 2.5 μl H_2_O_2_ per 5 ml Tris buffer (0.05 M; pH 7.6) for 1–2 min. Stained brain sections were extensively washed and mounted onto microscope slides. All brain sections were scanned using a Zeiss Axioscan Z1 slide scanner running Zeiss Zen Software (Carl Zeiss MicroImaging, Jena, Germany) with a 20× objective. Images were exported in Tagged Information File Format (TIFF). The background in the raw images was adjusted within the Zen software in order to optimise the signal to noise ratio, with the same parameters for all images, thereby allowing comparative results. The resolution of the exported Tiff images was constant within each series (0.284 μm/pixel for the antibody 1D1 and 0.265 μm/pixel for the antibodies 4G8 and J8).

### Use Case: Allen Mouse Brain Series

To demonstrate quantification of another type of labelling, the QUINT workflow was applied to parvalbumin positive cells in an image series exported from the Allen Mouse Brain Atlas Data Portal. The image series encompassed 20 sagittal mouse brain sections from the left hemisphere labelled for parvalbumin by *in situ* hybridisation, available at http://mouse.brain-map.org/experiment/show/75457579 (© 2004 Allen Institute for Brain Science. Allen Mouse Brain Atlas. Available from: mouse.brain-map.org). All the analysis parameters and workflow output files for this dataset are published on the human brain project (HBP) Platform (DOI: 10.25493/6DYS-M3W; Yates and Puchades, 2019), and so are not described here in full.

### Image Pre-processing Steps: Nutil Transform

The *Transform* feature in the *Nutil* software enables image rotation, renaming, resizing and mirroring and was used to prepare the image series for *QuickNII* alignment and *ilastik* segmentation. Several sets of images were prepared as the input size requirements for the *QuickNII* and *ilastik* software differ. For *QuickNII*, the input requirements are described in Puchades et al. (2019). For *ilastik* the resizing was performed in order to enable efficient processing and to comply with the pixel scale restriction of the features imposed by the *ilastik* software. To clarify, the pixel classification algorithm relies on input from manual user annotations of training images, and the features–intensity, edge and/or texture–of the image pixels. The features at different scales are computed as filters with pre-smoothing by a Gaussian with a sigma ranging from 0.3 to 10. For each pixel, the algorithm thus considers the values of the filters in a small sphere around the pixel (the maximal sphere radius is approximately 35 pixels) in the annotated regions, on a scale of 0.3 to 10 pixels. This means that the pixel features must fall within a maximum 10 × 10 pixel window for detection (for example, a repeating textural pattern). The resize factor was selected with reference to this maximum pixel scale to bring the labelled objects within the detection range for all the features, hence achieving a better segmentation (see the *ilastik* manual for more information). In practise, a test run was performed with *ilastik* on images of different sizes to find the optimal resolution for segmentation, with a final resize factor of 0.1 selected for the pE-Aβ series, and a factor of 0.05 for the hAPP and pan-Aβ series.

The *Nutil software* is shared through the HBP^1^ and is available for download at NITRC^2^ with an extensive user manual. See also Github^3^.

### Alignment of Sections to Atlas Space With QuickNII

**Figure 2:**
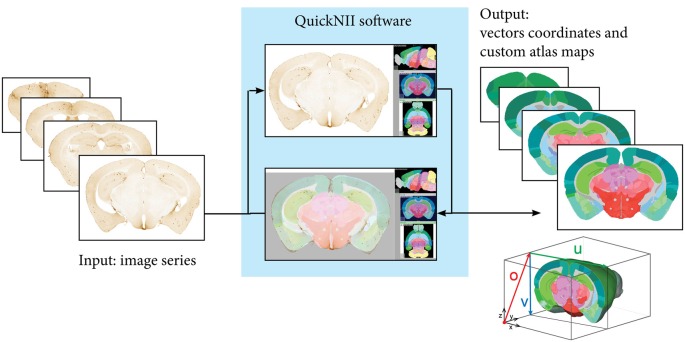
Registration to reference atlas by *QuickNII*. Following initial preprocessing steps where the sequence and orientation of the serial images is validated and a configuration file generated, images are imported to *QuickNII* together with a 3-D reference atlas of the mouse brain. In *QuickNII*, an atlas overlay image which is interactively manipulated to generate an image with position, scale, and orientation (rotation and tilt) that best matches the selected experimental images (DV: +13; ML: −4). *QuickNII* automatically propagates information about position, scale, and tilt to the entire series. By iterative anchoring of selected key sections, the user can optimize the automatically propagated parameters. The rotation and position of the overlay atlas image is validated and if needed adjusted by the user. Output from *QuickNII* is a series of custom atlas plates matching each anchored experimental image, and an XML file describing a set of vectors (o, u, and v) that define the position of each image relative to the technical origin of the reference atlas used.

The three image series (hAPP, pan-Aβ and pE-Aβ) were aligned to reference atlas space with the *QuickNII* atlasing tool (Figure 2; Puchades et al., 2019). This open access software allows assignment of spatial location to serial brain section images. The reference atlases available in the tool are the Waxholm Space Rat Atlas for rat data (Papp et al., 2014; Kjonigsen et al., 2015) and the Allen Mouse Brain Atlas for mouse data (© 2004 Allen Institute for Brain Science. Allen Mouse Brain Atlas. Available from: http://download.alleninstitute.org/informatics-archive/current-release/mouse_ccf/annotation/ccf_2015/) (Lein et al., 2007; Oh et al., 2014).

Within *QuickNII*, the volumetric brain reference atlases are used to generate customised atlas maps that match the spatial orientation and proportions of the experimental sections. In the software, the location is defined by superimposing the reference atlas onto the section images in a process termed “anchoring.” In “anchoring” the cutting angle of the reference atlas is adjusted to match the plane of the sections, with the position of each section identified prior to a manual adaptation of each atlas image to match the section images using affine transformations. Anchoring of a series of, e.g., 100 sections from an animal, typically takes 2–6 h, depending on the quality of the sections in the series (distorted sections are more difficult to anchor).

The *QuickNII* software is available at NITRC^4^ through the HBP^1^.

### Image Segmentation With *Ilastik*

**Figure 3:**
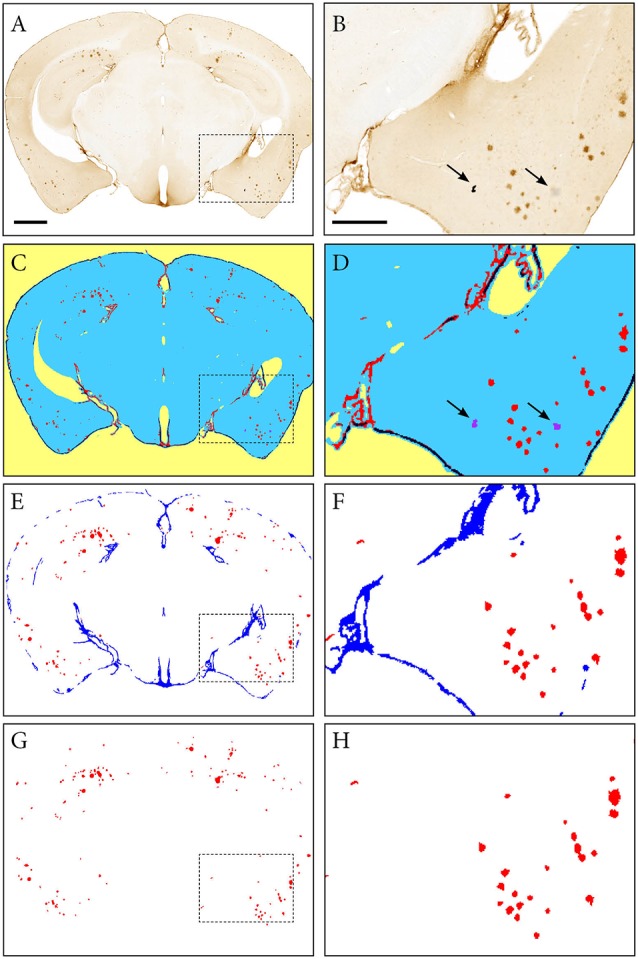
Segmentation of images with the *ilastik* software. An image of a Tg2576 mouse brain section, immunohistochemically labelled for pan-Aβ (4G8), processed with the pixel and object classification workflows in the *ilastik* software (version 1.2.2. post2). Panels **(A,C,E,G)** show the whole image, with **(B,D,F,H)** representing the area identified in the dashed box. **(C,D)** show the output of the pixel classification workflow, with images segmented into five classes based on differences in intensity, edge and texture (red: specific immunohistochemical labelling, blue: unlabelled tissue, purple: artefacts, black: non-specific labelling, yellow: background). The pixel classification workflow is able to differentiate labelling and artefacts such as marks on the coverslip and debris (see arrows). Panels **(E,F)** show the output of the object classification workflow: the probability maps derived from the pixel classification workflow were thresholded at 0.4 for the channel representing the labelling, and classified into two classes based on object-level features such as size and shape (red: β-amyloid plaques, blue: non-specific labelling). Panels **(G,H)** show the object classification output with the blue channel removed to visualize the β-amyloid plaques only. Images **(A,C,E,G)** are displayed at the same magnification with the scale bar representing 1 mm. The scale bar for figures **(B,D,F,H)** represents 500 μm.

The *ilastik* software was used to segment the downscaled section images for immunohistochemically labelled plaques (60 images per series: hAPP, Aβ and pE-Aβ; Berg et al., 2019; version 1.2.2. post2 for Windows, 64-bit). The segmentation was performed in two steps. First using Pixel Classification to differentiate the immunoreactivity from the background, followed by Object Classification to differentiate the specific immunoreactivity from labelled artefacts (Figure 3). For each image series, only 10% of the images were used to train the classifiers, which were then applied to the whole series in a batch mode, saving considerable time compared to an individual segmentation approach (segmentation of a whole image series takes a few hours depending on the size and number of images).

#### *Ilastik* Pixel Classification Workflow

The Pixel Classifiers were trained with the training images selected for each series (approximately every 6th section per series). All the available features (texture, edge, and intensity) and feature scales (0.3–10 pixels) were included in the classification algorithms. In the training phase, annotations were placed on the first training image, a few pixels at a time, with inspection of the predictions with each annotation. To refine the classifier and increase its applicability to the whole series, each training image was annotated in turn until the predictions were of a good standard across all the training images. The trained classifier was then applied to the series in the batch mode, with probability maps exported for the whole series.

#### *Ilastik* Object Classification Workflow

The object classifier differentiates objects based on features such as size and shape, and was applied to the output of the pixel classification to remove artefacts that could not be removed by pixel classification alone (for example, the elongated immunoreactivity around the edges of sections as opposed to the typically circular plaques). The training approach was the same as for the pixel classification, with the same subset of training images used. The probability maps were thresholded at a probability of 0.4 for all the series, with the object size filters set to 8–10,00,000 pixels for the hAPP and pE-Aβ series, and 4–10,00,000 pixels for the pan-Aβ series (the pan-Aβ labelled objects were smaller than the hAPP and pE-Aβ objects). All the object features in the *ilastik* software, except the location features, were included in the classifier (find more information on this in the *ilastik* user manual). The trained classifier was applied to the whole series in the batch mode, with the object prediction maps exported in PNG format. *NIH imageJ* was then used to apply colours to the predictions maps with the glasbey lookup table, and the coloured versions used as input for *Nutil Quantifier*.

### Quantification of Labelling in the Different Brain Regions With Nutil Quantifier

Once the section images were segmented (*ilastik*) and registered to the relevant reference atlas (*QuickNII*), *Nutil*—a software application developed in-house—was used to extract quantitative data about the labelling in each region in the reference atlas (*Quantifier* feature).

*Nutil* is a stand-alone application that allows for object classification from arbitrary image input files. The code for *Quantifier* uses a standard recursive pixel filling algorithm in order to scan for and separate individual objects in a 2D segmented image. This means that for each pixel that is not classified as a background pixel, the algorithm checks whether there are neighbouring pixels that are also not part of the background. If so, *Nutil* applies the same algorithm to these neighbours, and repeats the process until all surrounding pixels are background only. The cluster of collected pixels is considered to be an object, which is added to a global list of objects before being assigned a label ID that is matched with the corresponding reference atlas. This is performed by selecting the top left pixel from each identified object and using this position as a lookup in the reference atlas image files. In addition, the statistical properties of each cluster are calculated and stored (position, width, height, area, size, et cetera). When the entire batch process has completed, reports are produced, which are based on user inputs such as individual colour assignment for different label IDs, areas to exclude, areas to merge, et cetera. Finally, a set of report files are generated, in addition to customised atlas images superimposed with colour-coded (and labelled) objects.

*Nutil* is available for download at NITRC with an extensive user manual^5^. See also Github^6^. The *Nutil* *Quantifier* feature is fast to run, taking seconds to minutes on a desktop computer depending on the size and number of images for analysis.

### Validation of the Image Segmentation

In order to validate the segmentations produced with the *ilastik* software, their area outputs as determined with *Nutil* *Quantifier* were compared to ground truth measurements obtained by manual delineation of plaques for five sections (s14, s54, s94, s134 and s174), and to stereological measurements on 30 sections (s6, s14, s22, s30, s38, s40, s54, s62, s70, s78, s86, s94, s120, s110, s118, s126, s134, s142, s150, s158, s166, s174, s182, s190, s198, s206, s214, s222, s230, s238). The comparisons were performed on section images that were immunohistochemically labelled for hAPP (1D1 antibody) and restricted to clearly visible plaques (we excluded neuronal hAPP labelling). For both the 5 and 30 section subsets, the sections were regularly spaced and spanned the full volume of the brain. The subsets represented 8% and 50% of the full hAPP series, respectively. The section images that were used to train the classifiers (training images) were not selected for the validation.

The ground truth area measurements were obtained for five of the sections by manual delineation of the hAPP immunoreactive plaques by an expert in the field, with the *NIH ImageJ* tool (Analyse function) on images at 5% of the original size. Immunolabelled plaques were delineated for individual objects at the pixel level. For each image, the surface area occupied by plaques was calculated with reference to the resize factor and the pixel length in the original image.

Stereological analysis of hAPP immunoreactivity was performed with the Area Fraction Fractionator probe in the *MBF Stereo Investigator* software (version 2017.02.2; MBF Bioscience, Chicago, IL, USA) with a sampling grid of 300 μm × 300 μm, a counting frame of 200 μm × 200 μm, and a 20 μm point spacing. The settings were selected with reference to the literature (Tucker et al., 2008; Liu et al., 2017; Wagner et al., 2017). Points within the section contours that overlapped the hAPP immunoreactive plaques were marked as positive; with all remaining points marked as negative. hAPP plaque load was calculated by the software with respect to the magnification. The 30 section subset included the five sections for which ground truth measurements were available, allowing comparison of three methods for the five section subset.

### Validation of the Atlas Delineations

To validate the atlas delineations derived from the *QuickNII* software, we compared the plaque loads for five sections in three anatomical brain regions delineated by two alternative methods. The comparisons were performed on section images that were immunohistochemically labelled for hAPP (1D1 antibody) and restricted to clearly visible plaques (we excluded neuronal hAPP labelling). For the first delineation method, five section images were segmented to extract labelled plaques with the *ilastik* method. The segmentations were then visualised on top of the original images, and the cortex, olfactory region and hippocampus manually delineated with the *NIH ImageJ* tool with guidance from the Franklin and Paxinos mouse brain atlas version 3 (Franklin and Paxinos, 2008). The *Analyse* function in *NIH ImageJ* was used to quantify hAPP plaques in the delineated regions for each brain section. Brain region-specific hAPP load was calculated by dividing the area occupied by hAPP labelling within the selected brain region by the total area occupied by the brain region. For the second method, the same five segmentations were processed with the QUINT workflow with the delineations derived from the *QuickNII* atlas maps. The hAPP loads were extracted for the cortex, olfactory region and hippocampus for the five sections from the output reports.

## Results

### Workflow Description

We present the QUINT workflow for quantification and spatial analysis of features in large series of labelled mouse or rat brain sections (Figure 1). The different steps are indicated below:

1. Image pre-processing (change the contrast, resolution, file type) with the *Transform* feature in the *Nutil* software
2. Registration of sections to reference atlas space using the *QuickNII software* to generate atlas maps adapted to the orientation of the images
3. Segmentation of the labelling with the *ilastik* image analysis software using two classifiers
4. Data analysis with the *Quantifier* feature in the *Nutil* software (combines the segmentation results with input from the atlas maps to give a list of individual plaque features, region level features and whole brain features, enabling quantitative regional analysis).

The procedures used in all steps (*a to d*) are detailed in the “Materials and Methods” section. Image pre-processing: (a) is necessary in order to produce copies of the images that are suitable for each tool in the workflow. The registration of the sections; (b) to the Allen Mouse Brain Atlas (© 2004 Allen Institute for Brain Science. Allen Mouse Brain Atlas, Available from: http://download.alleninstitute.org/informatics-archive/current-release/mouse_ccf/annotation/ccf_2015/; Lein et al., 2007; Oh et al., 2014) is performed with the *QuickNII* tool (Puchades et al., 2019). Briefly, as illustrated in Figure 2, image series are uploaded to the software and visualised with reference atlas overlays that have adjustable transparency. A few sections with highly distinguishable landmarks are selected for adjusting the dorso-ventral and the medio-lateral angles of the atlas in order to match the cutting angles of the brain sections. Once these sections are registered to the atlas, the software automatically propagates the spatial information to the rest of the image series. After a rapid overview of the registration results and eventual minor positional adjustments, atlas maps corresponding to each image are exported. These atlas maps are used for the region-based analysis of the labelled features.

The next step (c) consists of segmentation of the labelled features present in the brain sections (Figures 3A,B). In the first *ilastik* step, a subset of the image series is used to train the classifier with the pixel classification workflow (Figures 3C,D). The user defines classes based on intensity, edge and texture and annotates a few example pixels of each class. The second *ilastik* step, object classification, is used for the removal of artefacts (Figures 3E,F). The resulting segmentations identify the plaques in a colour with a unique RGB colour code (Figures 3G,H).

In the last step (d), the *Quantifier* feature in *Nutil* enables quantitative regional analysis of labelling based on the segmentations and corresponding atlas maps. The software is simple to run, requiring no specialist computing or programming knowledge. The user specifies the path to the input and output directories in a simple Microsoft Excel template titled *Quantifier* (the input directories should contain the segmentations and the atlas maps), in addition to entering analysis parameters. The necessary input parameters are the pixel scale (area represented by one pixel in the segmentations), and the minimum and maximum object size cut-offs. The template is then uploaded to the *Nutil* software, which drawn on the information in the template to perform the analysis. The output files are automatically saved to the specified output directory and consist of quantitative reports with variables such as number of objects and surface area of objects per region. Text files listing the xyz coordinates of each segmented pixel in reference atlas space are also generated for viewing with the *Meshview* atlas viewer (provided *via* the MediaWiki link at https://www.nitrc.org/projects/meshview). *Nutil* also generates customised atlas images with the segmented objects superimposed providing an overview of the objects per atlas region.

### Validation of Object Segmentation

**Figure 4:**
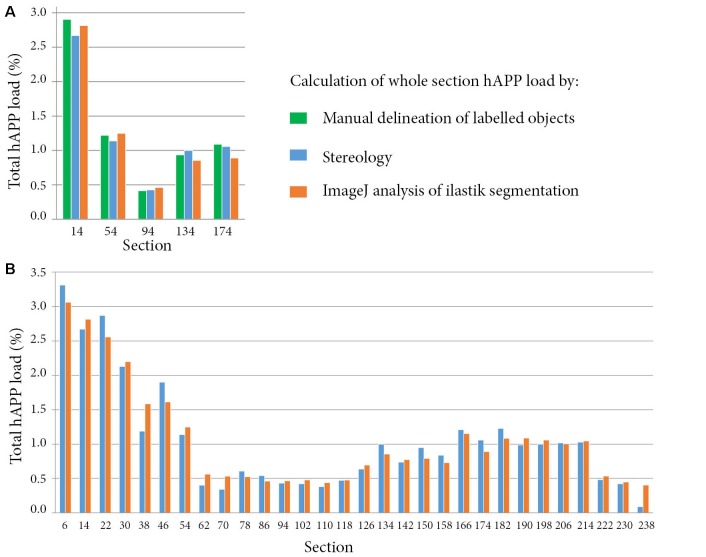
Comparison of whole section human amyloid precursor protein (hAPP) load outputs from three alternative quantitative methods. For all the methods, the whole section hAPP load was calculated by dividing the area occupied by hAPP labelling by the total section area. Calculations were restricted to plaques that were immuno-labelled with the hAPP antibody (1D1). Panel **(A)** compares hAPP load outputs from three alternative methods for five sections. The methods include expert manual delineation of hAPP labelled objects (green), stereological estimate with the area fraction fractionator probe (blue), and quantification with NIH *ImageJ* based on the *ilastik* segmentations (orange). Panel **(B)** compares hAPP load outputs from the stereological method and from the segmentations for thirty whole brain sections that were regularly spaced and spanned the full volume of the brain.

As accurate segmentation of the labelled objects is important for a valid quantitative result, we decided to compare our results with three different methods. The segmentations generated with *ilastik* were compared to manual delineation of labelled objects by an expert in the field (five sections), and to measurements obtained with a stereological method (30 sections). The hAPP labelled series was selected for the validation. The *ilastik* segmentations gave hAPP load estimates that were similar to the stereological estimates, and that represented the outputs from manual object delineations for the five sections for which manual object delineations were available (error of *ilastik* estimates relative to manual object delineations: mean −0.06% with a SD of 0.09%; error of stereological estimate relative to manual object delineations: mean −0.05% with a SD of 0.11%; see Figure 4A).

For the 30 sections, the mean error of the hAPP loads from the *ilastik* method relative to the stereological method was 2.79 × 10^−3^% with a SD of 0.16% (see Figure 4B). To summarise, this means that for this image series, the *ilastik* method allows the user to establish the plaque load (restricted to hAPP labelled plaques) with 95% confidence to within an error of ±0.32%. As described in the results, the plaque load variations detected from section to section and between brain regions were of a much greater magnitude than this error, indicating that the *ilastik* method is suitable for detecting these differences.

### Validation of Anatomical Delineations From QuickNII

**Figure 5:**
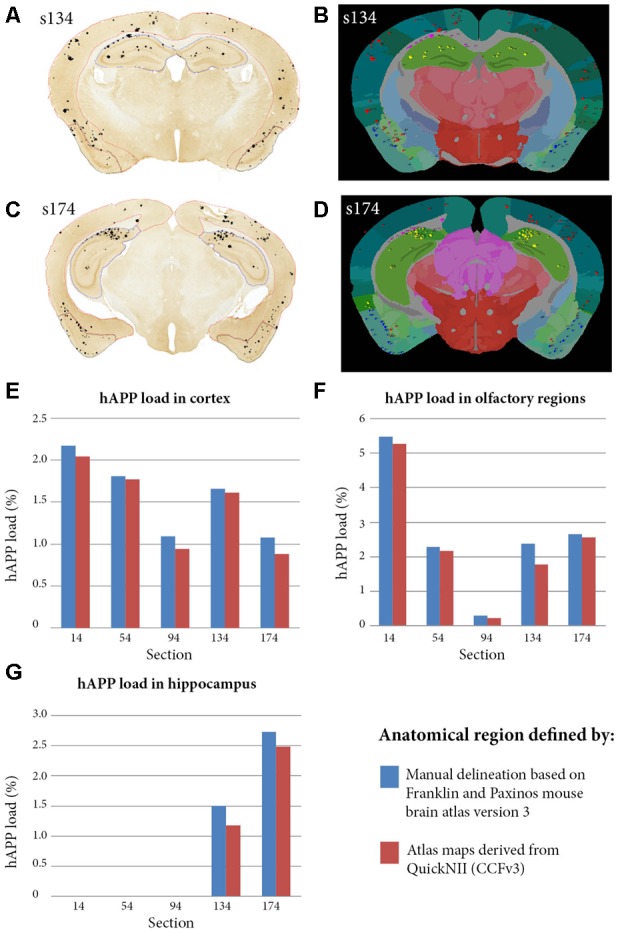
Comparison of hAPP load outputs in three anatomical brain regions defined by two alternative anatomical delineation methods. Brain region-specific hAPP load was calculated by dividing the area occupied by hAPP labelling within the selected brain region, by the total area occupied by the brain region. The calculations were restricted to plaques that were immuno-labelled with the hAPP antibody (1D1). For the first method, five brain section images were segmented with *ilastik* and visualised on top of the sections to allow manual delineation of brain regions. The cortex, olfactory region and hippocampus were delineated with *NIH* *ImageJ* with guidance from the Franklin and Paxinos mouse brain atlas version 3 (panels **A,C** show the cortex, olfactory region and hippocampus delineated in red, blue and yellow in section s134 and s174 respectively). The analyse function in NIH *Image J* was used to quantify hAPP load in the delineated regions. For the second method, the five segmentations were processed with the QUINT workflow with input from the *QuickNII* derived atlas maps (panels **B,D** show examples for s134 and s174 respectively). hAPP loads were extracted for the cortex, olfactory region and hippocampus from the output reports. Panels **(E–G)** compare hAPP loads in the cortex, olfactory regions and hippocampus respectively for the five sections, with the loads calculated by the two alternative methods described.

In a separate study, to validate the accuracy of the atlas delineations from *QuickNII*, we compared the hAPP loads from the QUINT workflow to loads calculated based on manual delineations of three brain regions for five sections (cortex, olfactory region and hippocampus; Figure 5). The *QuickNII* delineations gave hAPP loads that were representative of the loads from the manual delineations for all the sections and brain regions that were investigated (Figures 5E–G). Overall, the QUINT workflow slightly underestimated the hAPP loads relative to the manual method for all the explored brain regions (deviation of the workflow derived cortical hAPP load from the manual method: mean of −0.11% with SD of 0.07%; deviation of workflow derived olfactory hAPP load from manual method: mean of −0.21% with SD of 0.23%; mean and SD are not provided for the hippocampus as only two sections contained this region).

### Use Case Analysis

The QUINT workflow was used to analyse and compare three consecutive series labelled for hAPP (1D1), pan-Aβ (4G8) and pE-Aβ (J8) in one Tg2576 mouse model for Alzheimer’s disease. Each series were composed of approximately 60 sections extending from the olfactory lobes to the cerebrum (the cerebellum was not included). All three series were registered to the Allen Mouse Brain Atlas (© 2004 Allen Institute for Brain Science. Allen Mouse Brain Atlas, Available from: http://download.alleninstitute.org/informatics-archive/current-release/mouse_ccf/annotation/ccf_2015/) using *QuickNII*. The section images can be viewed with custom atlas overlays adjusted for angle deviations (Supplementary Data Sheet 1).

**Figure 6:**
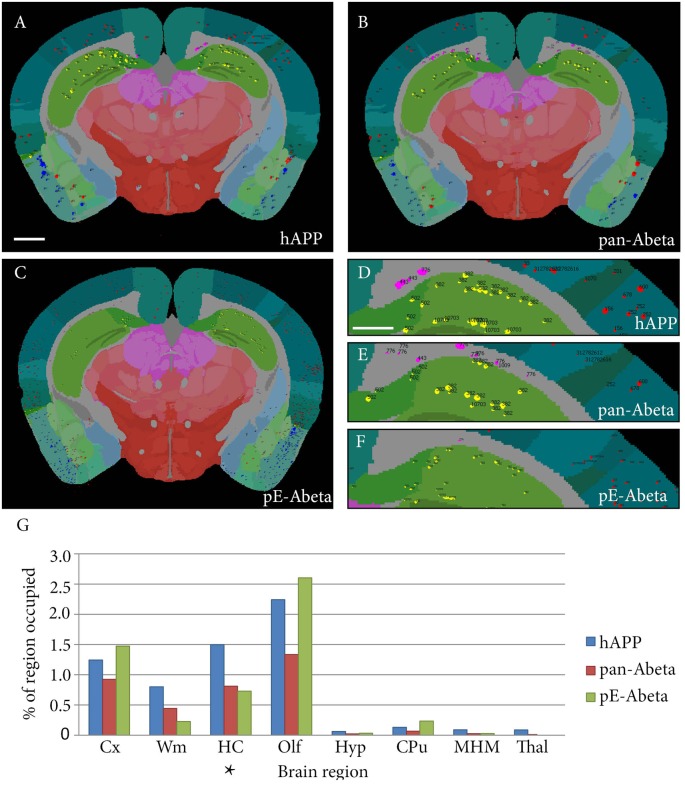
Whole brain comparative analysis of three series labelled for hAPP, pE-Aβ and pan-Aβ in a Tg 2576 Mouse. Examples of Nutil image output (segmentations superimposed on the atlas maps) for the hAPP **(A,D)**, pan-Aβ **(B,E)** and pE-Aβ series **(C,F)**. The segmented object colours represent their anatomical location: isocortex (red); hippocampus (yellow); white matter tracts (pink); olfactory regions (blue); caudate putamen (CPu; black). Panel **(G)** shows the comparative quantification results for the whole brain for the three series (the blue, red and green bars represent hAPP, pan-Aβ and pE-Aβ labelling respectively). The abbreviations in the graph represent the following brain regions: isocortex (Cx); white matter tracts (Wm); hippocampal region (HC); olfactory regions (Olf); hypothalamus (Hyp); CPu; midbrain, hind brain and medulla (MHM); thalamus (Thal). Images **(A–C)** are displayed at the same magnification with the scale bar representing 1 mm. The scale bar for figures **(D–F)** represents 500 μm. The asterisk in panel **(G)** indicates the region represented in Figure 7.

**Figure 7:**
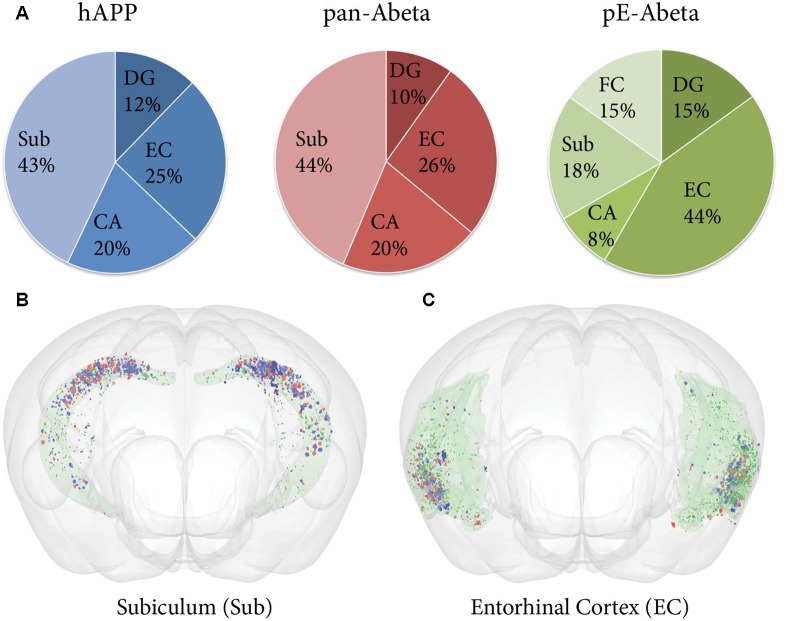
Comparative analysis of hAPP, pE-Aβ and pan-Aβ labelling in the hippocampus of a Tg 2576 mouse.** (A)** The pie charts show the percentage of the total labelling of hAPP (blue chart), pan-Aβ (red chart) and pE-Aβ (green chart) in the hippocampus expressed in the subiculum (Sub), dentate gyrus (DG), entorhinal cortex (EC), cornu ammonis (CA) and fasciola cinereum (FC; **B,C**). The expression differences are visualised for the subiculum and the EC with the MeshView atlas viewer (the regions are shown in pale green with the Nutil output from the three series covisualised, with objects labelled for hAPP, pan-Aβ and pE-Aβ in blue, red and dark green respectively). Both the pie charts and the brain images reveal spatial expression differences for the three markers in the hippocampus.

**Figure 8:**
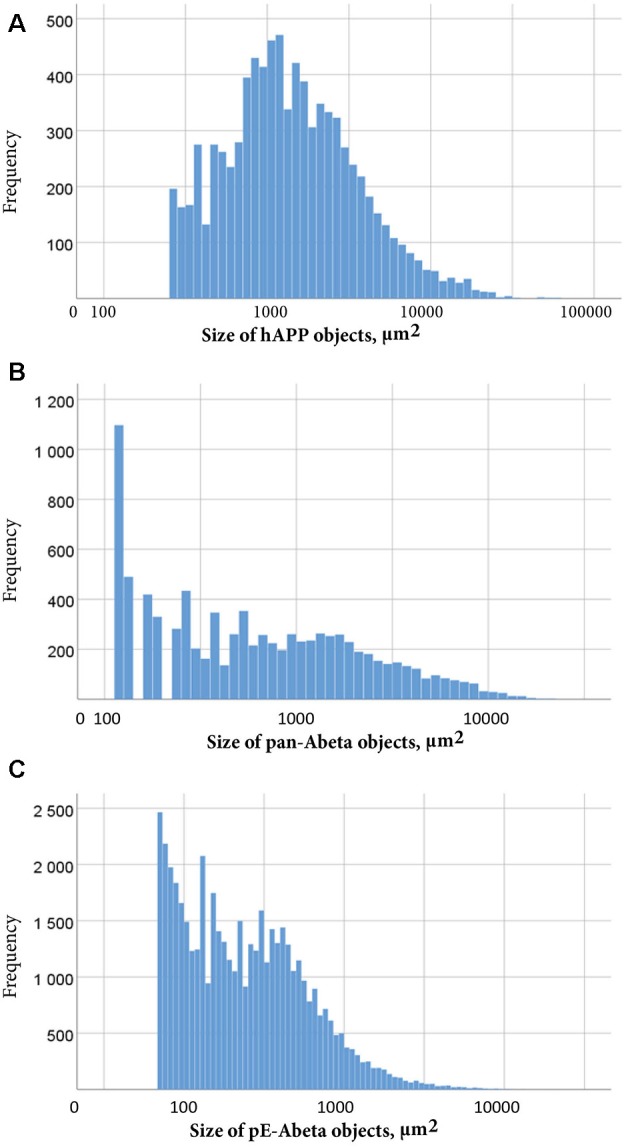
Size distribution of objects labelled for hAPP **(A)**, pan-Aβ **(B)** and pE-Aβ **(C)** in a whole T2576 mouse brain series. Object size in μm^2^ is represented on the x-axis on a common logarithmic scale with frequency on the y-axis. To remove false positive objects, minimum object size cut-offs of 258 μm^2^, 112 μm^2^ and 56 μm^2^ were applied to the hAPP **(A)**, pan-Aβ **(B)** and pE-Aβ **(C)** series, respectively.

Labelled plaques were segmented and quantified using the QUINT workflow as represented in Figures 6, 7. Plaques were found mainly in the olfactory regions (1.5–2.5%), the neocortex (1–1.5%), the hippocampal region (1–1.5%) and white matter tracts (0.5%; Figure 6G). The plaque burden was lower in the striatum, thalamic regions and midbrain (less than 0.5%). All Aβ and APP species co-localised in the same regions, with small differences, as seen in Figures 6A–C. The customised atlas images superimposed with colour-coded objects are found in the Supplementary Material. The size of plaques and their distribution in the whole series is illustrated in Figure 8. The pE-Aβ positive plaques were more numerous but much smaller in size than plaques labelled for hAPP or pan-Aβ labelled with 4G8 (Figure 7). As we were interested to detect subregion expression differences in the hippocampus, we refined the analysis to smaller brain regions. As demonstrated in Figure 7, the subiculum showed more hAPP and pan-Aβ labelling than the entorhinal cortex (EC), the cornu ammonis (CA) region of the hippocampus and the dentate gyrus (DG), whereas the subregion with highest pE-Aβ labelling was the EC.

Our workflow is demonstrated here on brain section images from one animal only, with analysis restricted to hAPP and Aβ plaques. However, the QUINT workflow can also be applied to other types of labelling like cell somas, as demonstrated by the quantification and spatial analysis of parvalbumin positive cells from an Allen mouse brain *in situ* hybridisation experiment shared through the HBP platform: DOI: 10.25493/6DYS-M3W (Yates and Puchades, 2019).

## Discussion

In this report, we present a new workflow for analysis of labelling in brain-wide image series. The QUINT workflow builds on newly developed tools and resources for brain atlasing and segmentation, and consists of three main steps. In the first step, *QuickNII* (Puchades et al., 2019) is used to generate customised atlas maps corresponding to experimental brain sections for mice, using the Allen Mouse Brain Atlas (© 2004 Allen Institute for Brain Science. Allen Mouse Brain Atlas, Available from: http://download.alleninstitute.org/informatics-archive/current-release/mouse_ccf/; Lein et al., 2007; Oh et al., 2014), and for rats using the Waxholm rat brain atlas version 2.0 (Papp et al., 2014; Kjonigsen et al., 2015). In the second step, the machine learning-based image analysis tool, *ilastik*, is used to segment the objects of interest from the immunolabelled images. In the final step, *Nutil* is used to combine the customised atlas maps and segmented images and to extract and quantify objects in each parcellated brain region for each section and for the whole image series. The tools allow users to perform analyses at different levels, and to customise the granularity of such analyses. Furthermore, *Nutil* supports the extraction of spatial coordinates for each segmented object for viewing in the MeshView brain atlas viewer (AMBA version 3 2015, available at www.nitrc.org/projects/meshview *via* the MediaWiki link). The QUINT workflow is also compatible with segmentations generated with other image analysis software, so users are not restricted to using *ilastik* for segmentation.

As a proof of concept, and to further characterise the amyloid expression in the Tg2576 Alzheimer mouse model, the workflow was applied to three series labelled with antibodies against the hAPP N-terminus (1D1), pan-Aβ (4G8) and pE-Aβ (J8). Our results show a plaque load of 1–3% depending on the brain region, and are in accordance with other studies (Schilling et al., 2008; Liu et al., 2017). When analysing the plaque load in more detailed brain regions, we were able to detect subregional differences. This was particularly true of the hippocampal regions where we detected the highest load for hAPP and pan-Aβ in the subiculum, compared to pE-Aβ that had more prominent labelling in the entorhinal cortex. This subregional difference could be of relevance to the pathophysiology and may be related to the expression of the enzyme that catalyses pE-Aβ formation (Hartlage-Rübsamen et al., 2009). Studies indicate that this protein might influence or even seed the aggregation of other amyloid peptide species (Schilling et al., 2006; Schlenzig et al., 2009; Nussbaum et al., 2012), and so it is interesting to observe its localisation from a mechanistic point of view. Our workflow allows comparison of the expression of different proteins across brain regions for any region defined in the Allen Mouse Brain Atlas, potentially highlighting associations that would otherwise remain undetected.

We conducted a validation study of the workflow in two parts, with the first exploring how well the outputs from the *ilastik* segmentations corresponded to outputs from two alternative quantitative methods. The alternative methods included manual delineation of labelled objects, which was performed on five sections that were regularly spaced thoughout the whole brain, and a stereological method that was applied to thirty sections (half of the full dataset).

For all the sections, the segmentations gave plaque load estimates that were similar to the outputs from the other two methods, with the *ilastik* method establishing the plaque load with 95% confidence to within an error of ±0.32% of the stereological output. In other words, the *ilastik* method was as good at detecting the absolute plaque load per section as the stereological method in this mouse. The absolute error rate could be reduced further by introducing a manual adjustment step to remove false-positive labelling from the segmentations. However, even without this manual adjustment, the method is sensitive enough to detect the significant differences in plaque expression that are seen between different sections. A known challenge for the stereological evaluation was the sparse distribution of the plaques throughout the brain, and the concentration of the plaques in the frontal regions, which could at least partially account for the discrepancy between the stereological outputs and the outputs from the manual object delineations. As suggested by Boyce and Gundersen (Boyce and Gundersen, 2018), the classic fractionator approaches that rely on systematic random sampling are highly inefficient and impractical for sparse labelling. However, by increasing the sampling frequency in our stereological analysis, we obtained results very close to the manual delineation of objects.

As demonstrated there are clear advantages to a segmentation based workflow. However, segmentation also introduces some limiting factors. One limitation is that it imposes restrictions on the resolution of the images that can be used as input. In the examples shown here, we segment relatively large objects (plaques) and therefore had the option to downscale the raw images to speed up the analysis, while still achieving good quantification of labelling (*ilastik* has an upper image size limit). However, when segmenting smaller protein aggregates, such as nuclear Huntingtin (not shown), downsizing is not an option. In this case, the images would first have to be split into high resolution tiles, in order to perform the segmentation, and then retiled prior to analysis. Furthermore, as explained in the methods part, the size of the object (number of pixels) has an impact on the segmentation quality as there are restrictions on the pixel scales of the features that can be included in the *ilastik* algorithm (scale up to 10 pixels for intensity, edge and texture in the *ilastik* version used here). A test run with some representative images of different sizes is therefore recommended to determine the optimal image resolution for segmentation. Alternatively, another software or analysis approach could be used to generate the segmentations. The workflow is compatible with segmentations from other image analysis software as long as they comply with the *Nutil* input requirement (segmentations must be 24-bit colour images in PNG format). Users are therefore not restricted to *ilastik* for segmentation.

In the second part of the validation study, we tested the accuracy of region-based quantification by comparing the plaque load outputs from the QUINT workflow to loads determined by atlas delineations that were manually applied for three regions (cortex, olfactory region and hippocampus) on five sections. We demonstrate that the QUINT workflow is able to detect the regional expression differences seen at this level of granularity for this image series. In this particular case, we found that the workflow slightly underestimated the real plaque load. Closer inspection of the *QuickNII* atlas maps for the selected sections showed that the anatomical location of a minority of the plaques were incorrectly assigned. For example, some of the hippocampal plaques were incorrectly assigned to the corpus callosum. Indeed, the accuracy of the workflow for region-based quantification is entirely dependent on the accuracy of overlap between the experimental section and the corresponding atlas map. Currently, we have to adapt the image registration of the *QuickNII* tool from a global fit (whole slide) to a more local fit when we want to analyse specific regions of the brain more precisely (this is particularly relevant for the analysis of smaller regions). However, this limitation would be circumvented if *QuickNII* supported non-linear registration of the image sections to the atlas, and this is planned for implementation in a future release.

One of the main advantages of the QUINT workflow for quantification is that it uses a reference atlas to delineate the regions, allowing studies on brain regions that are not usually explored. As most stereological studies require the experimenter to manually delineate the region of analysis, some regions with very few visible landmarks (i.e., thalamus or olfactory bulb) are typically not included in these studies. More importantly, as rodent reference atlas delineations are improved and extended, scientists will be able to conduct even more targeted studies enabling detailed mapping of subregional expression differences. By registering many datasets to the same reference atlas, the data are made more comparable and interoperable (Bjerke et al., 2018), increasing the likelihood for reuse. Importantly, our method relies on histological sections, without a need for block-face images.

The whole workflow is rapid, user-friendly and does not necessitate coding aptitudes as is often the case for similar image analysis software (Vandenberghe et al., 2016; Fürth et al., 2018; Xiong et al., 2018). The optimal dataset for the workflow would include images of undistorted whole brain tissue sections spanning the full volume and with clearly distinguishable features. We do not recommend the workflow for sections with major tissue distortions as no correction in QuickNII can compensate for this. Sections representing only one hemisphere will also lead to higher uncertainty, as it is not possible to determine the mediolateral cutting angle without assessing the appearance of landmarks in both the right and left hemisphere. This is also true for incomplete sections in which major parts of the brain are missing. There are, however, other types of limitations that the workflow can overcome. For example, non-specific labelling can be filtered out with the Object Classification workflow, and sections with tissue distortions may benefit from a local anchoring approach. Introducing corrections for limitations of these kinds may, however, increase the processing time or marginally increase the error rate.

The workflow is intended to enable more efficient and comprehensive analysis than is currently possible with traditional tools, but does not compensate for a lack of anatomical or biological expertise on the part of the researcher. Both the output of the segmentation and registration steps should be validated by visual inspection prior to quantification, and interpretations must be made in light of limitations. A complete analysis of a set of images such as those analysed here (approximately 60 images of sections of average quality) takes less than 24 h. The atlas registration can be done in 2–3 h, with the segmentation taking from 1 to 2 h depending on the image size, and the analysis with *Nutil* being very quick (less than 30 min). In conclusion, we believe that this workflow will enable large scale studies and the integration of results from many studies in different laboratories.

## Data Availability Statement

The datasets generated for this study are available on request to the corresponding author.

## Ethics Statement

All experiments were performed according to ethical guidelines (License number T28/16 of the Landesdirektion Sachsen, Germany).

## Author Contributions

SY performed *ilastik* and *Nutil* analyses, performed the validation studies, contributed to the development of the *Nutil* software and contributed to writing the article. NG created the *Nutil* software, contributed to the writing of the technical parts of the article and to the design of the validation studies. CC contributed to the development of workflows and validation studies. SL, P-HK, H-UD, MH-R and SR provided animal tissue and antibodies used in the use-case, and contributed to writing the article. TL contributed to the development of workflows and to writing the article. AK contributed with *ilastik* software support. MP conceived the study, supervised the analysis and the development of software tools, performed file pre-processing and *QuickNII* registrations and coordinated the writing of the article. JB conceived the study, supervised development of software tools, contributed with infrastructure, and contributed to writing of the article. All the authors reviewed and approved the manuscript.

## Conflict of Interest

The authors declare that the research was conducted in the absence of any commercial or financial relationships that could be construed as a potential conflict of interest.

## Footnotes

^1^www.humanbrainproject.eu

^2^https://www.nitrc.org/projects/nutil/

^3^https://github.com/leuat/nutil

^4^https://www.nitrc.org/projects/quicknii/

^5^https://www.nitrc.org/projects/nutil/

^6^https://github.com/leuat/nutil

## Supporting Information

A folder containing the customised atlas maps can be found online. The Supplementary File S1, contains a Table with overview of the different software as well as links to a viewer for raw data inspection.
