# Supplementary material for: QUINT: Workflow for Quantification and Spatial Analysis of Features in Histological Images From Rodent Brain
Source: Front Neuroinform. 2019 Dec 3;13:75. doi: 10.3389/fninf.2019.00075 (PMC6901597; doi:10.3389/fninf.2019.00075)
Supplement: Supplementary file 1 [file Table_1.DOCX]

**Supplementary File 1**

**Image series with custom atlas overlays**

**(**The links are for information only and are not part of the QUINT workflow).

For the series labelled with the 4G8 antibody detecting pan-Abeta: <http://cmbn-navigator.uio.no/navigator/filmstripzoom/filmstripzoom.html?atlas=300000&series=4806&preview=ABAMousev2Preview.png>

For the series labelled with the J8 antibody recognizing pE-Abeta: <http://cmbn-navigator.uio.no/navigator/filmstripzoom/filmstripzoom.html?atlas=300000&series=4807&preview=ABAMousev2Preview.png>

For the series labelled with the 1D1 antibody recognizing hAPP: <http://cmbn-navigator.uio.no/navigator/filmstripzoom/filmstripzoom.html?atlas=300000&series=4247&preview=ABAMousev2Preview.png>

**Table 1. Software and resources used in the QUINT workflow**

| **Software** | **Description** | **URL** |
| --- | --- | --- |
| *QuickNII* | Tool for registration of section images to a reference atlas (either mouse or rat) | <https://www.nitrc.org/projects/quicknii/> |
| *ilastik* | Segmentation of labelled features using Pixel Classification workflow and Object Classification workflow | [www.ilastik.org](http://www.ilastik.org) |
| *Nutil* | Transform feature for preprocessing the images and Quantifier feature for region based analysis | <https://www.nitrc.org/projects/nutil/> |
| *NIH ImageJ* | Tool used to apply the Glasbey lookup table to the ilastik segmentations | <https://imagej.nih.gov/ij/> |
| *Allen Brain Atlas CCF* | Reference atlas for mouse | <http://download.alleninstitute.org/informatics-archive/current-release/mouse_ccf/annotation/ccf_2015/> |
| *Waxholm Space Atlas of the Sprague Dawley rat* | Reference atlas for rat | <https://www.nitrc.org/projects/whs-sd-atlas> |
| *Meshview* | Online brain atlas viewer compatible with the *Nutil* coordinate output | <https://www.nitrc.org/projects/meshview/> |
