# Supplementary figures and images for: QUINT: Workflow for Quantification and Spatial Analysis of Features in Histological Images From Rodent Brain (part 2 of 2)
Source: Front Neuroinform. 2019 Dec 3;13:75. doi: 10.3389/fninf.2019.00075 (PMC6901597; doi:10.3389/fninf.2019.00075)

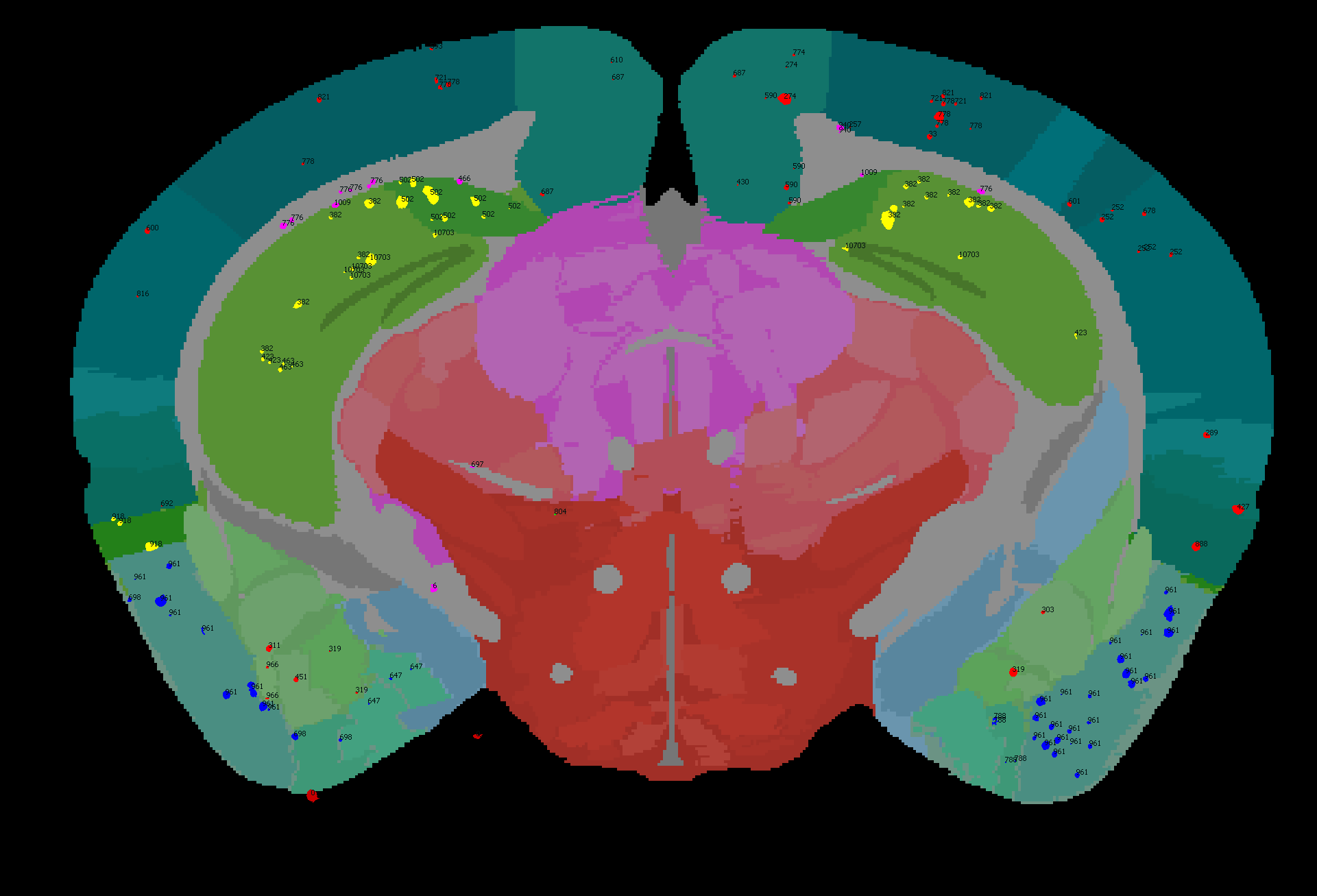

Supplement: Supplementary file 2 [file Data_Sheet_1.ZIP › Supplementary_material_Yates/pan-Abeta/tg2576_m287_4G8_s171_resize_Object Predictions.png]

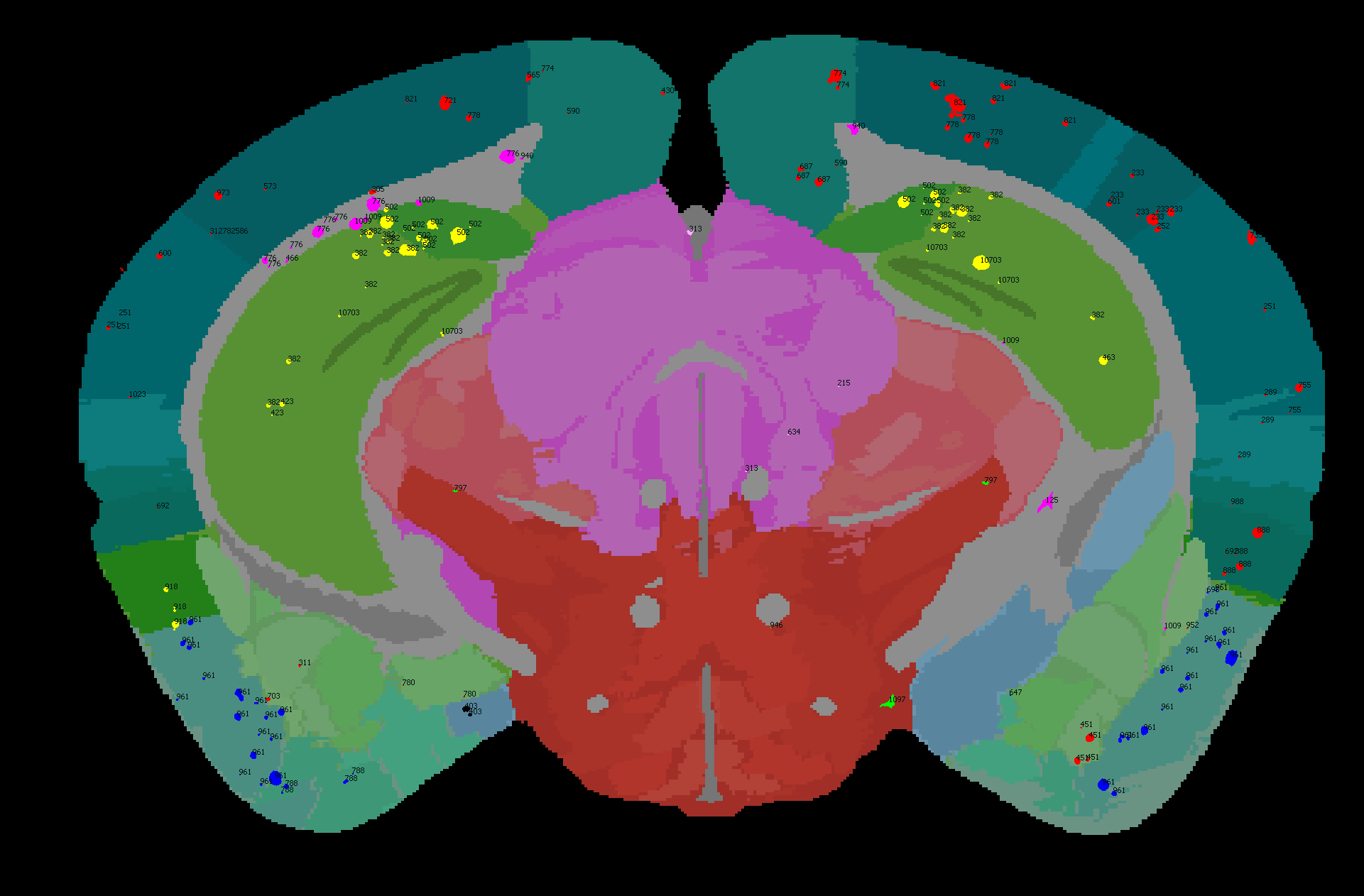

Supplement: Supplementary file 2 [file Data_Sheet_1.ZIP › Supplementary_material_Yates/pan-Abeta/tg2576_m287_4G8_s175_resize_Object Predictions.png]

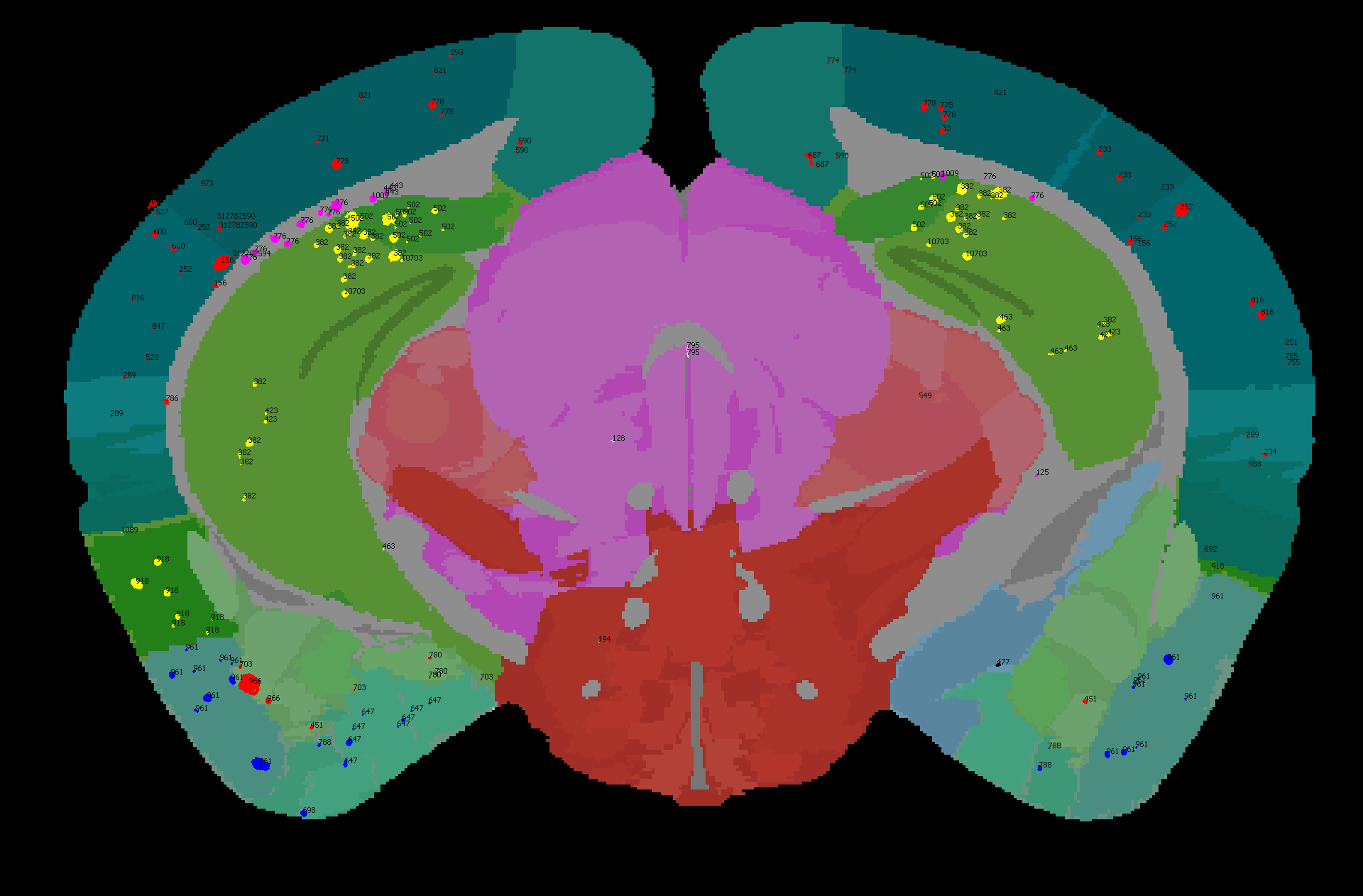

Supplement: Supplementary file 2 [file Data_Sheet_1.ZIP › Supplementary_material_Yates/pan-Abeta/tg2576_m287_4G8_s179_resize_Object Predictions.png]

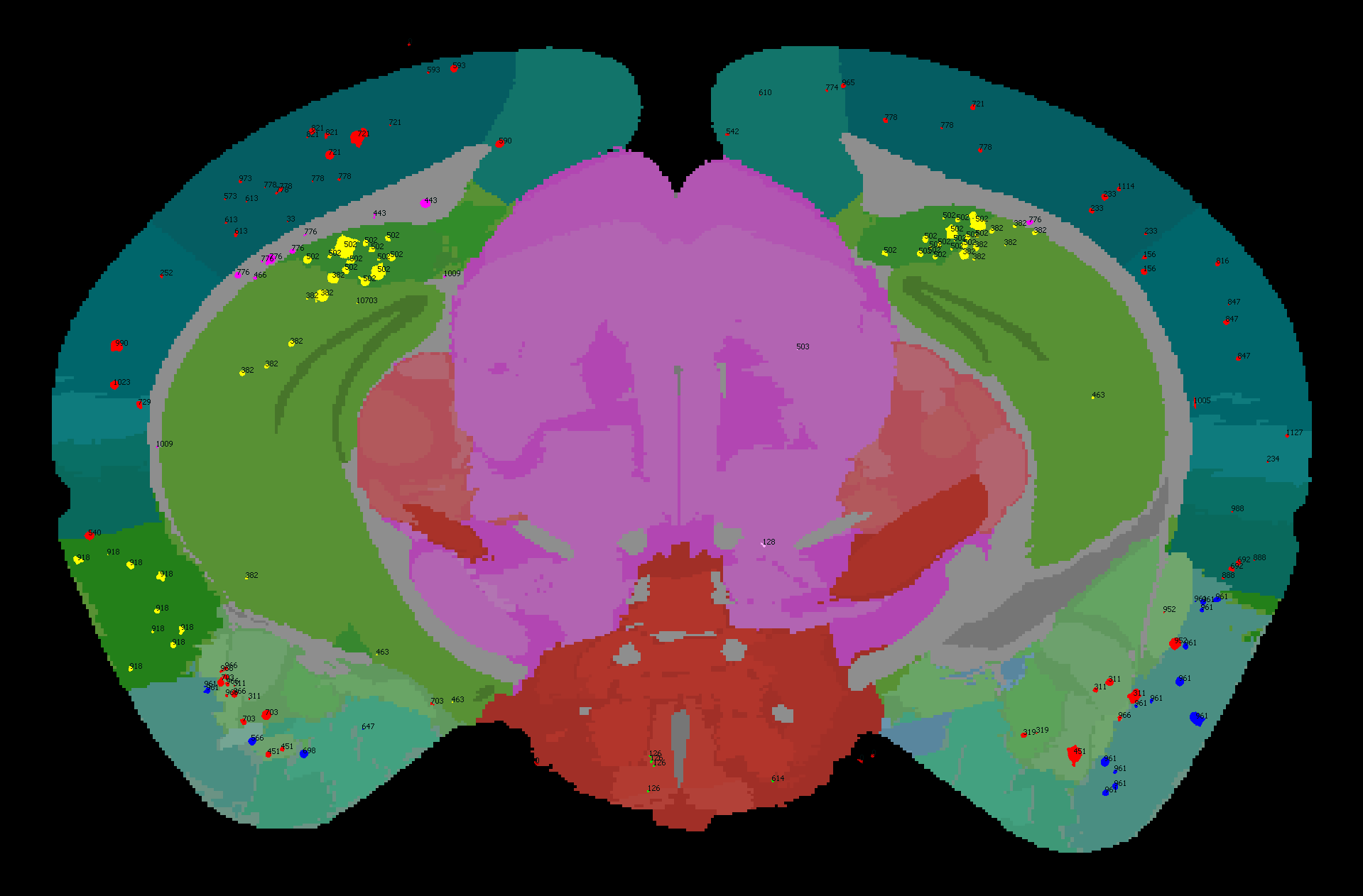

Supplement: Supplementary file 2 [file Data_Sheet_1.ZIP › Supplementary_material_Yates/pan-Abeta/tg2576_m287_4G8_s183_resize_Object Predictions.png]

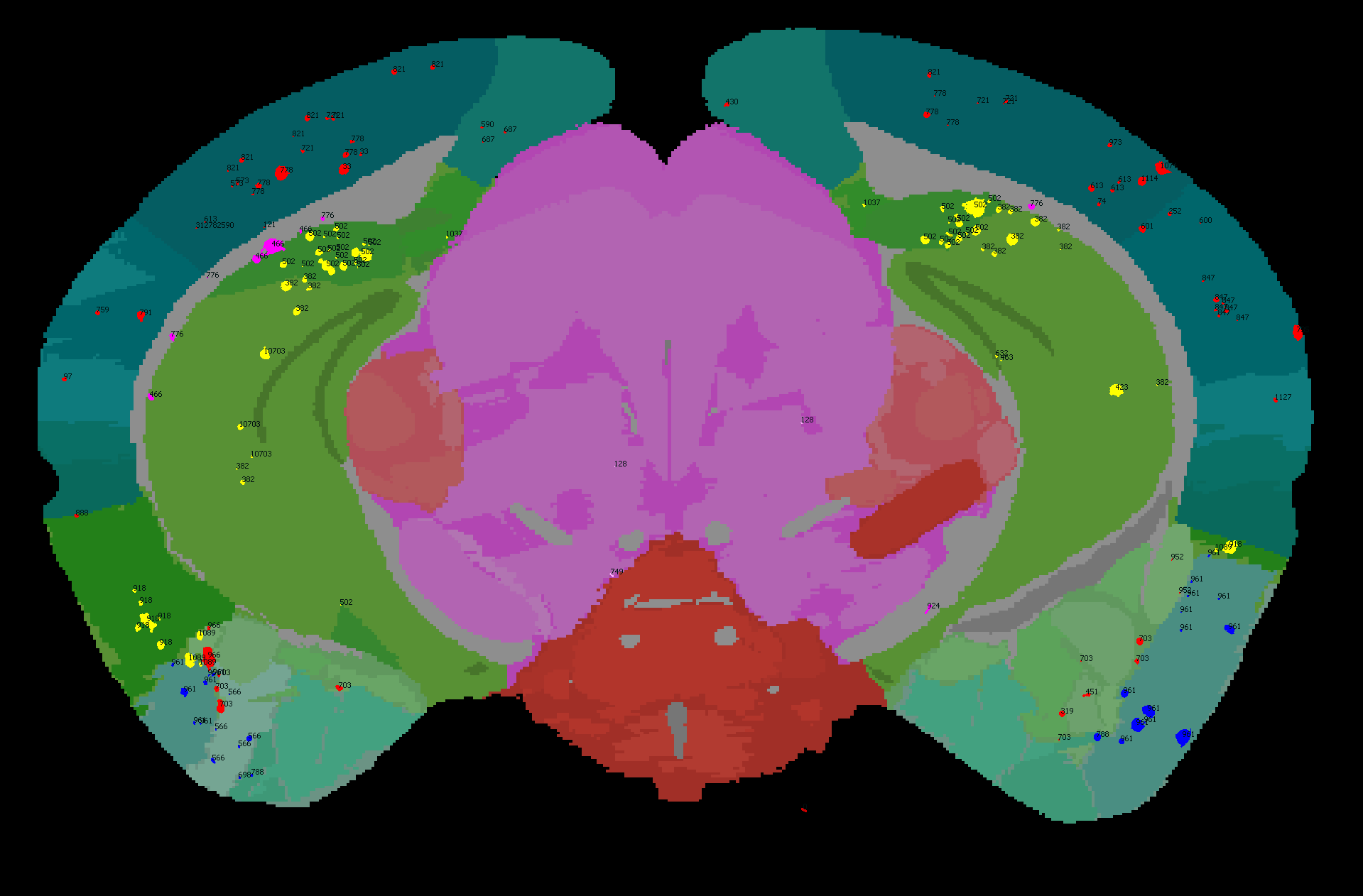

Supplement: Supplementary file 2 [file Data_Sheet_1.ZIP › Supplementary_material_Yates/pan-Abeta/tg2576_m287_4G8_s187_resize_Object Predictions.png]

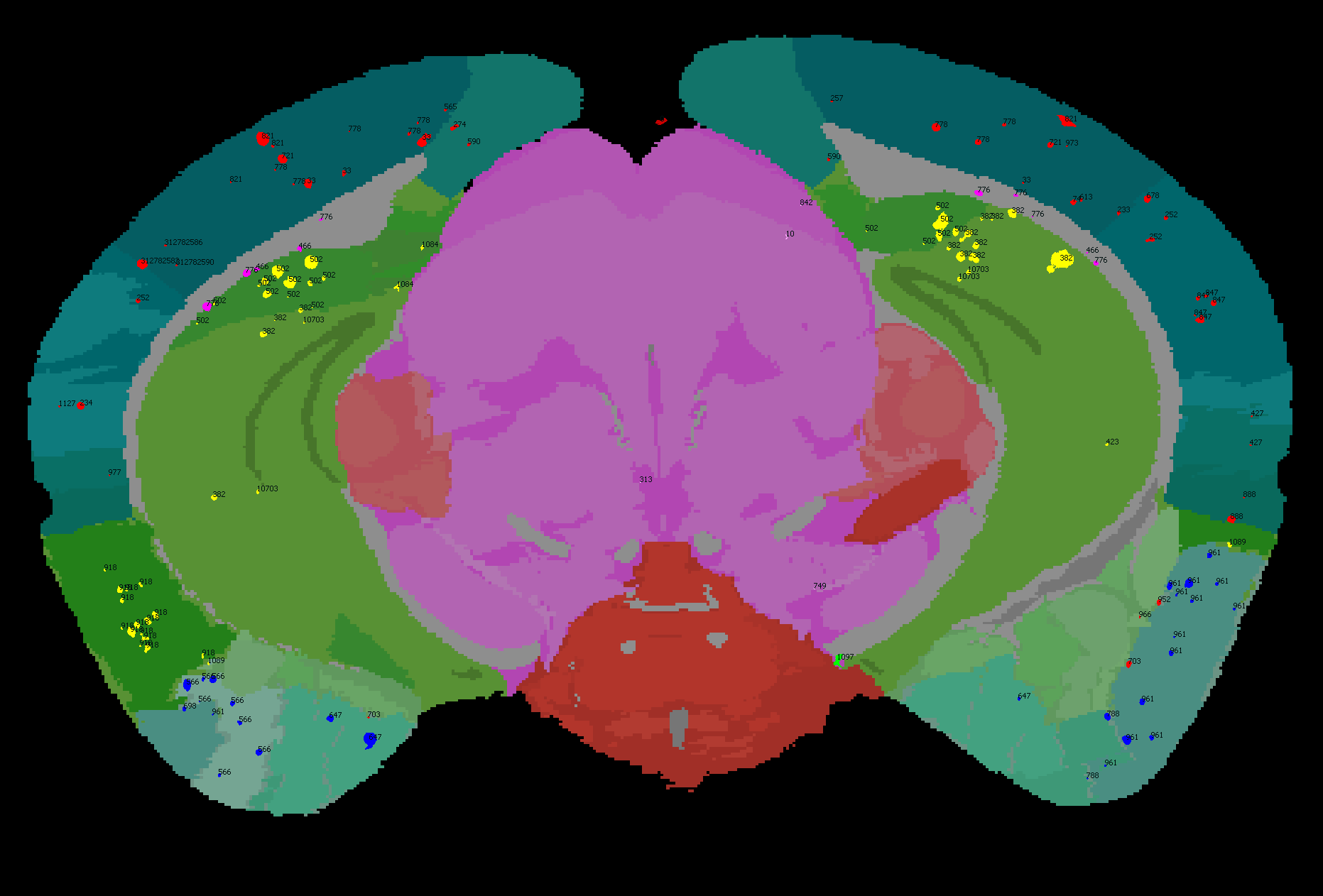

Supplement: Supplementary file 2 [file Data_Sheet_1.ZIP › Supplementary_material_Yates/pan-Abeta/tg2576_m287_4G8_s191_resize_Object Predictions.png]

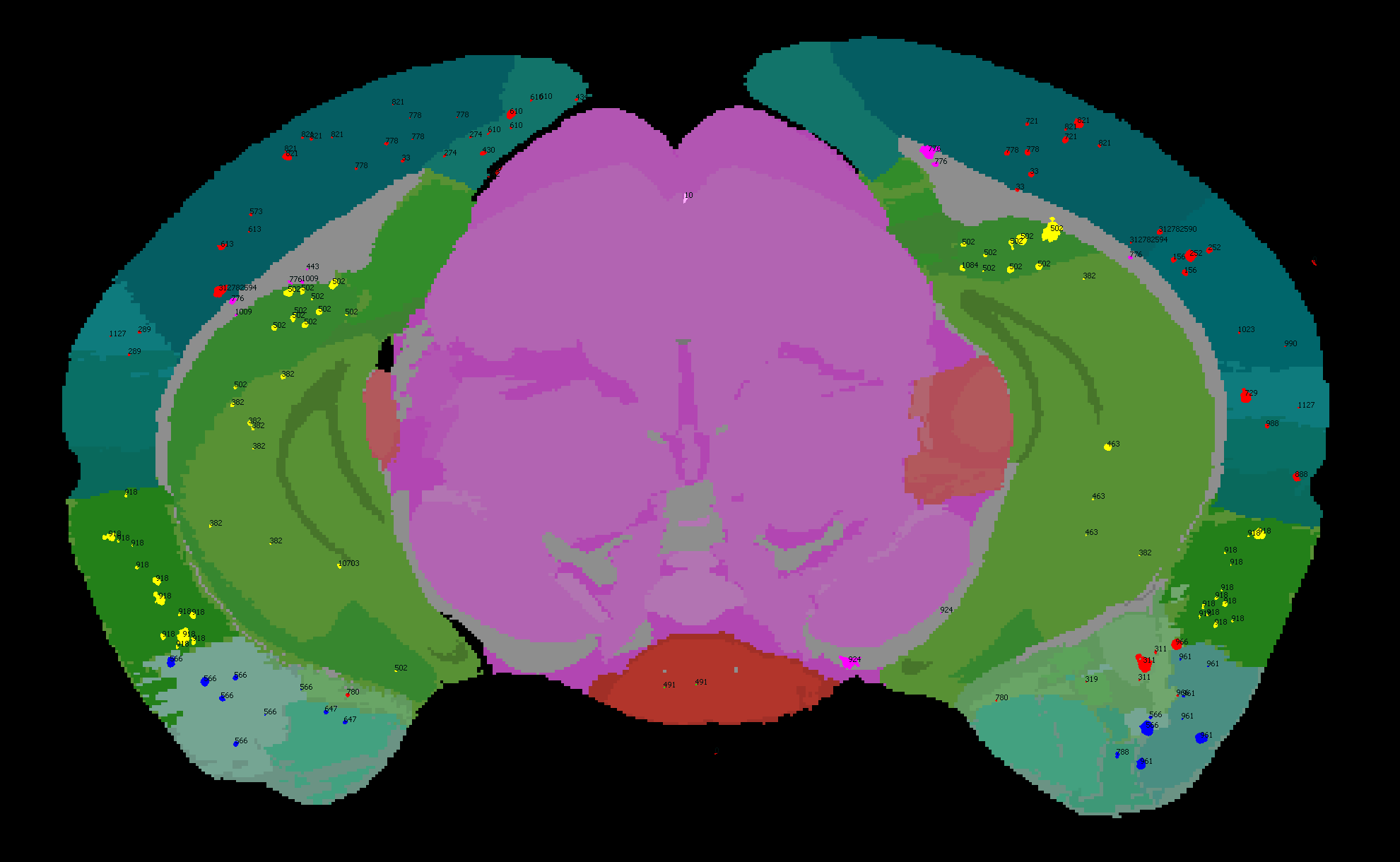

Supplement: Supplementary file 2 [file Data_Sheet_1.ZIP › Supplementary_material_Yates/pan-Abeta/tg2576_m287_4G8_s195_resize_Object Predictions.png]

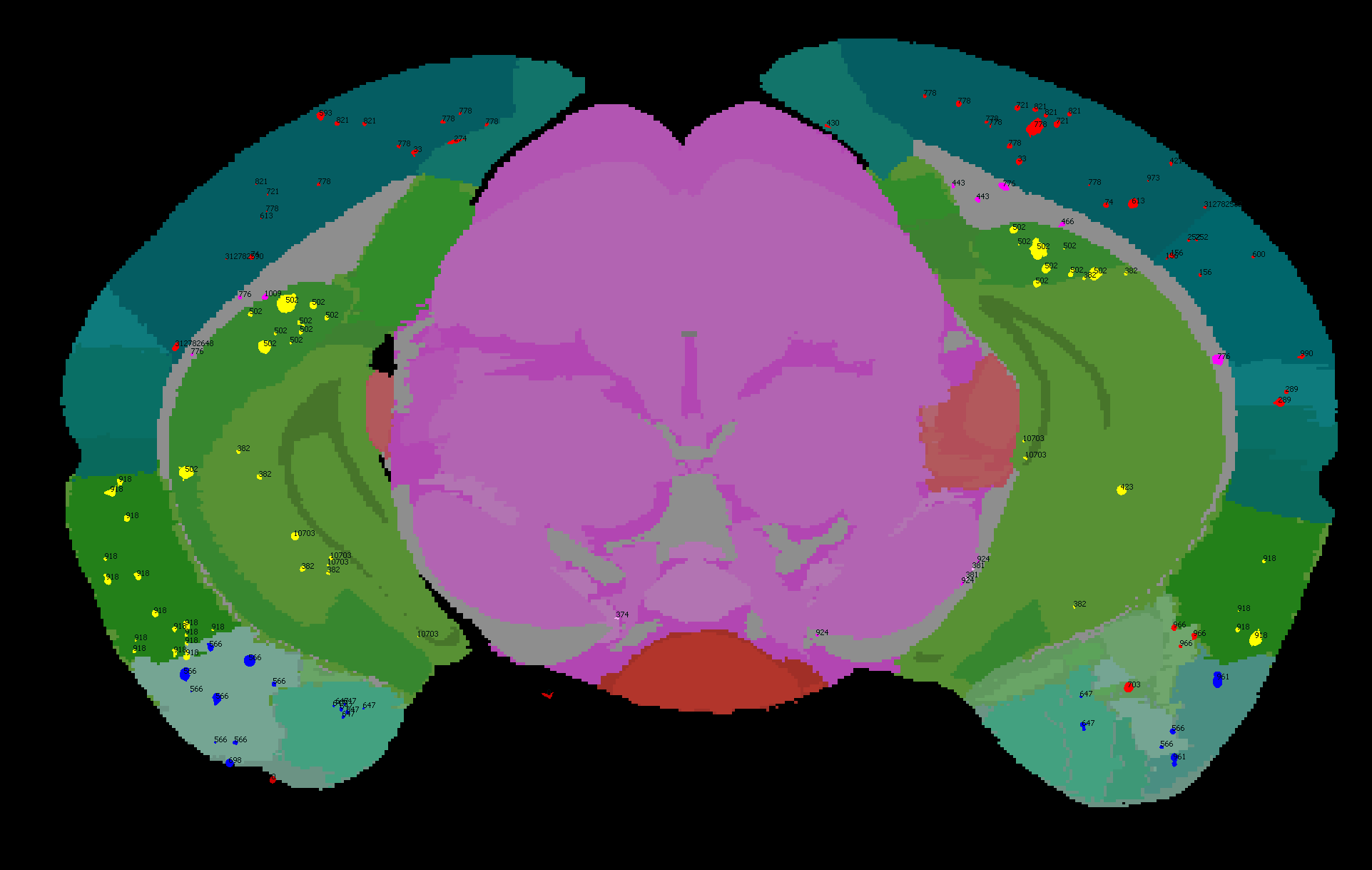

Supplement: Supplementary file 2 [file Data_Sheet_1.ZIP › Supplementary_material_Yates/pan-Abeta/tg2576_m287_4G8_s199_resize_Object Predictions.png]

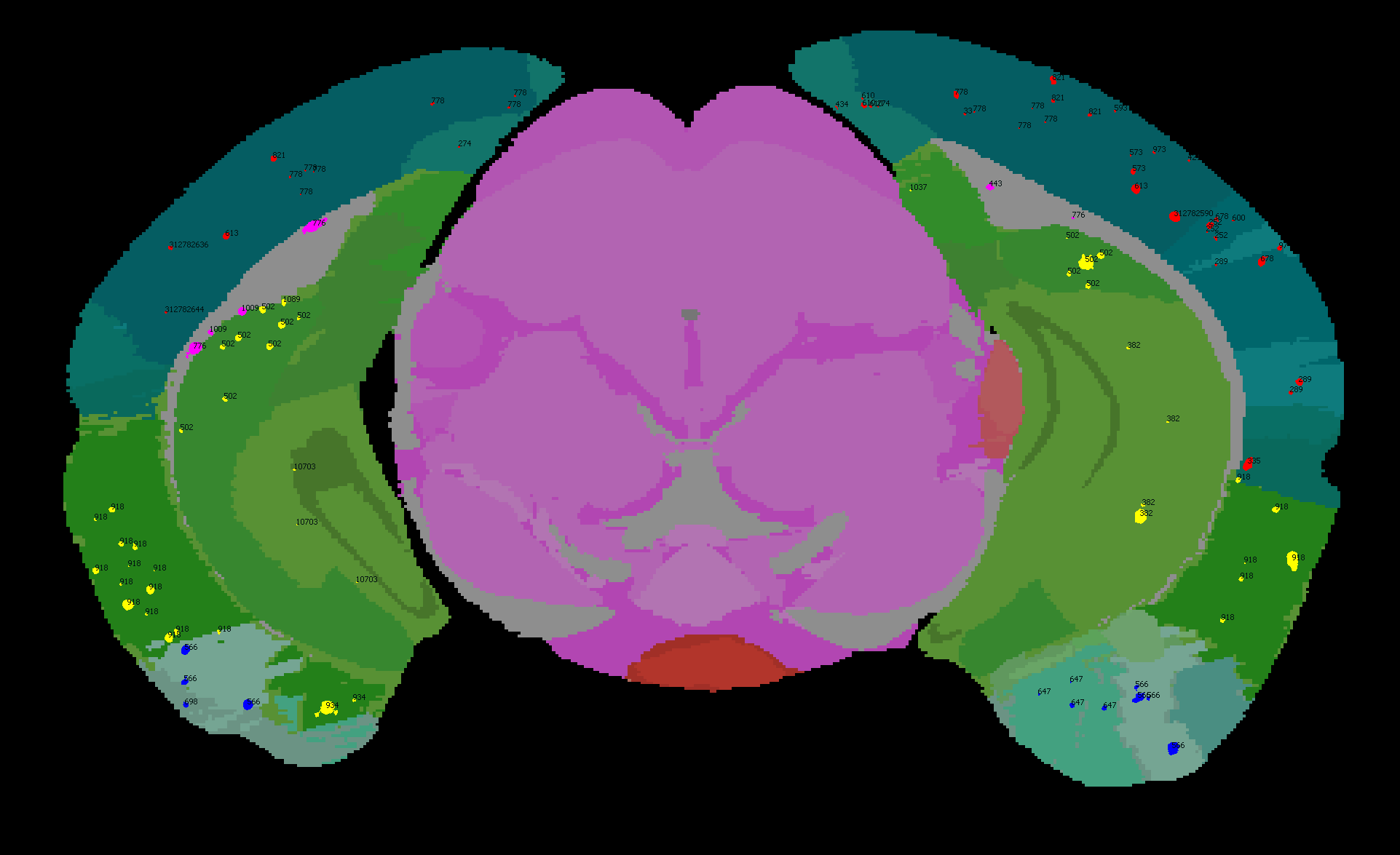

Supplement: Supplementary file 2 [file Data_Sheet_1.ZIP › Supplementary_material_Yates/pan-Abeta/tg2576_m287_4G8_s203_resize_Object Predictions.png]

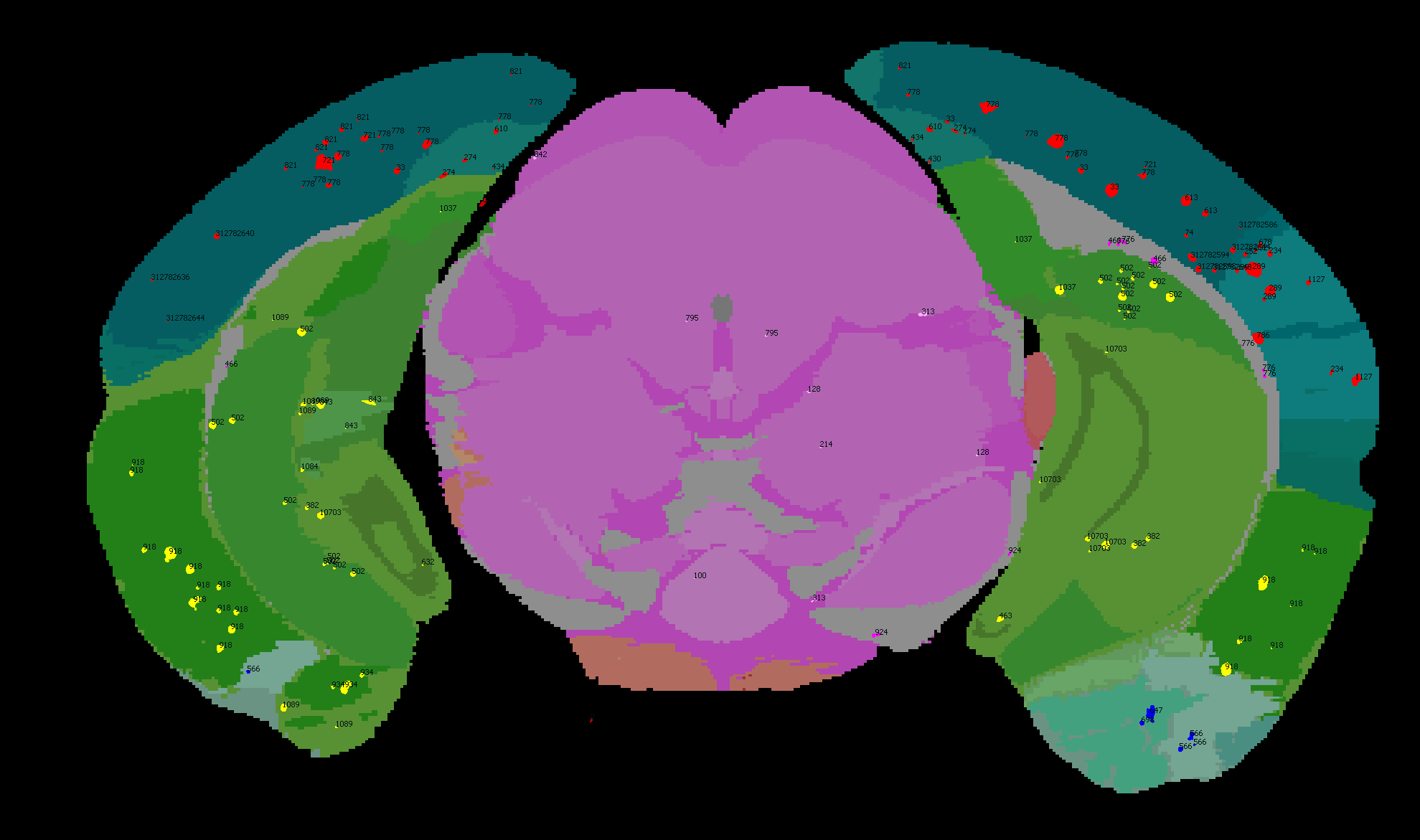

Supplement: Supplementary file 2 [file Data_Sheet_1.ZIP › Supplementary_material_Yates/pan-Abeta/tg2576_m287_4G8_s207_resize_Object Predictions.png]

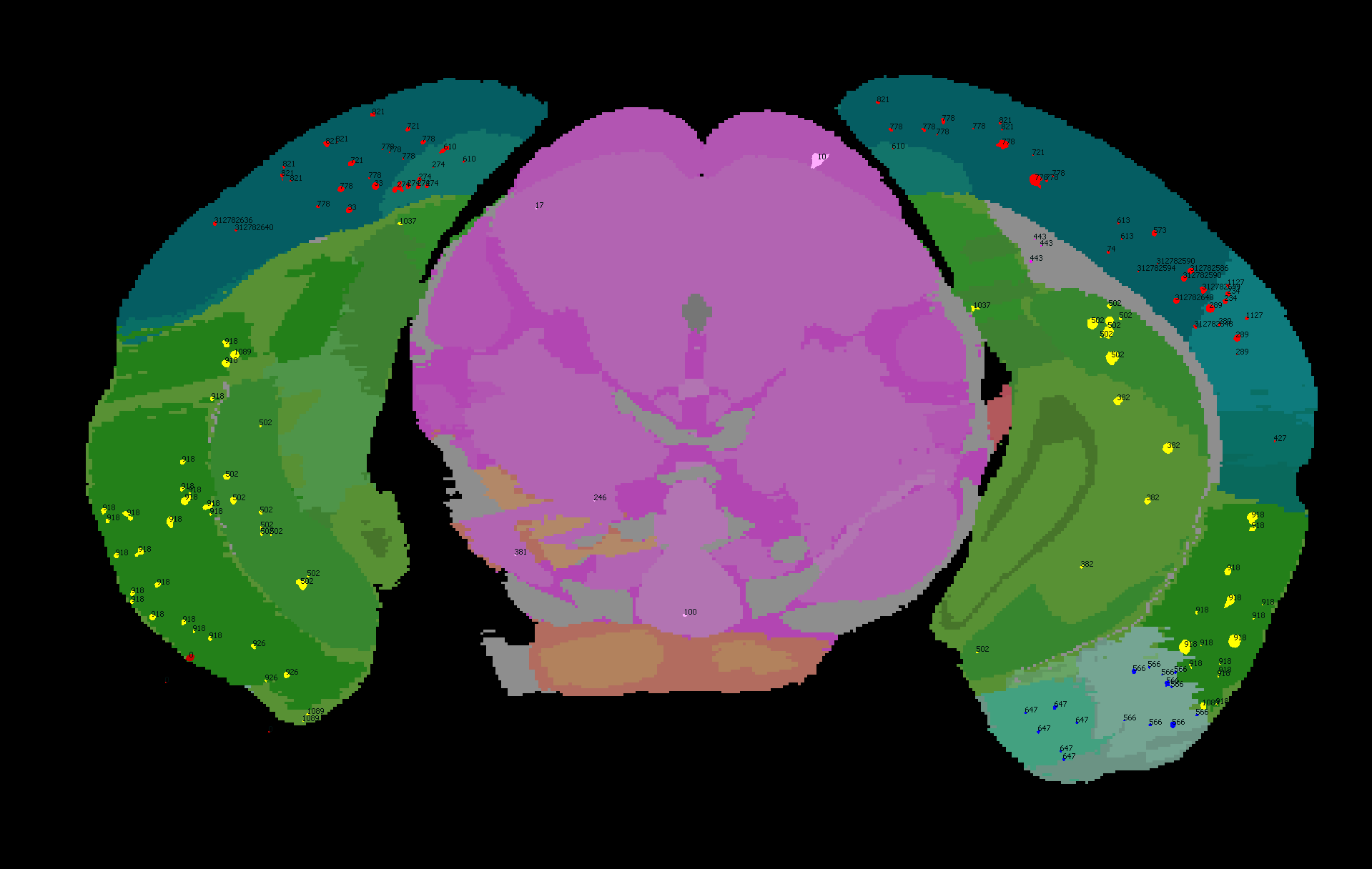

Supplement: Supplementary file 2 [file Data_Sheet_1.ZIP › Supplementary_material_Yates/pan-Abeta/tg2576_m287_4G8_s211_resize_Object Predictions.png]

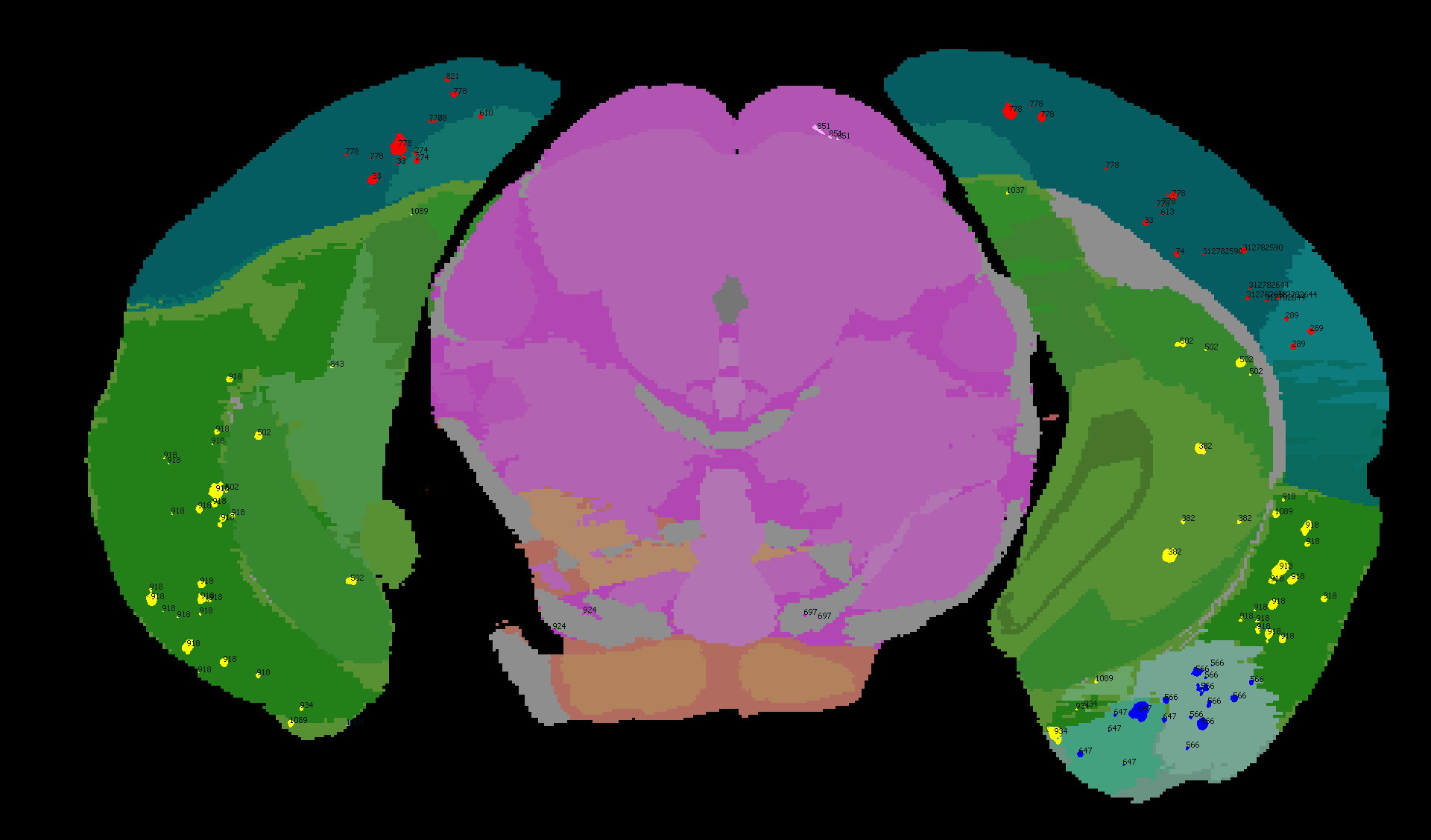

Supplement: Supplementary file 2 [file Data_Sheet_1.ZIP › Supplementary_material_Yates/pan-Abeta/tg2576_m287_4G8_s215_resize_Object Predictions.png]

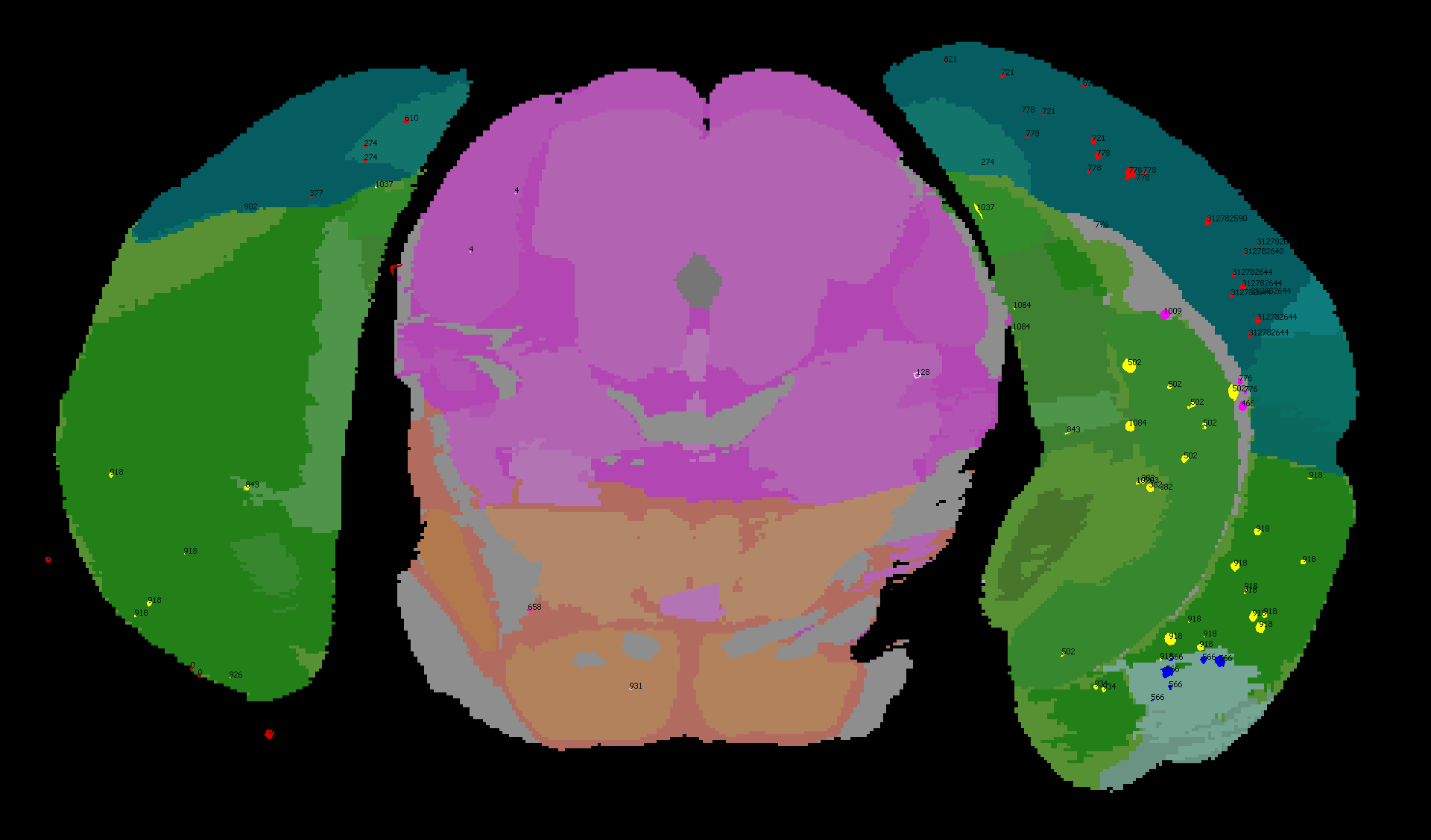

Supplement: Supplementary file 2 [file Data_Sheet_1.ZIP › Supplementary_material_Yates/pan-Abeta/tg2576_m287_4G8_s219_resize_Object Predictions.png]

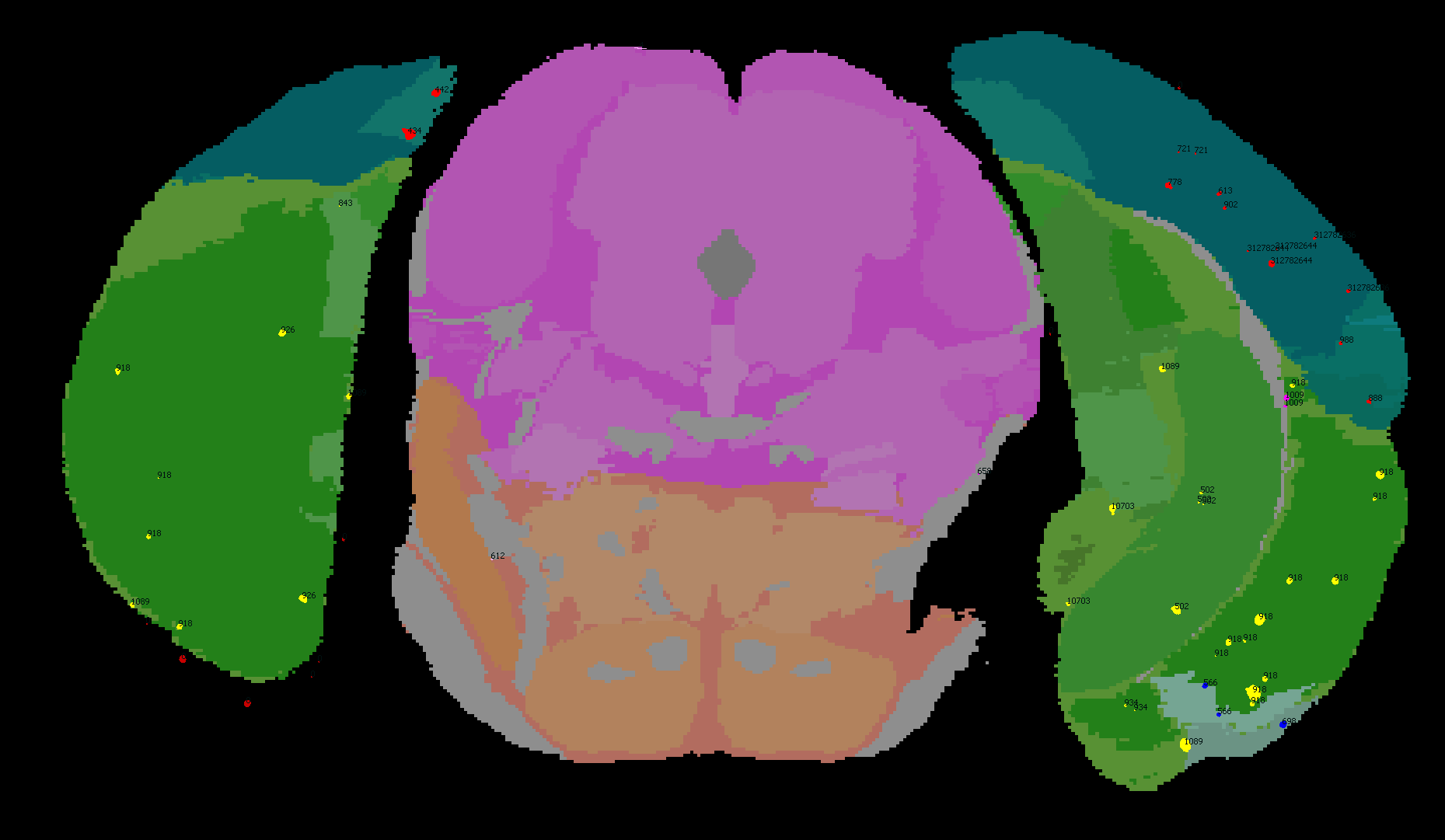

Supplement: Supplementary file 2 [file Data_Sheet_1.ZIP › Supplementary_material_Yates/pan-Abeta/tg2576_m287_4G8_s223_resize_Object Predictions.png]

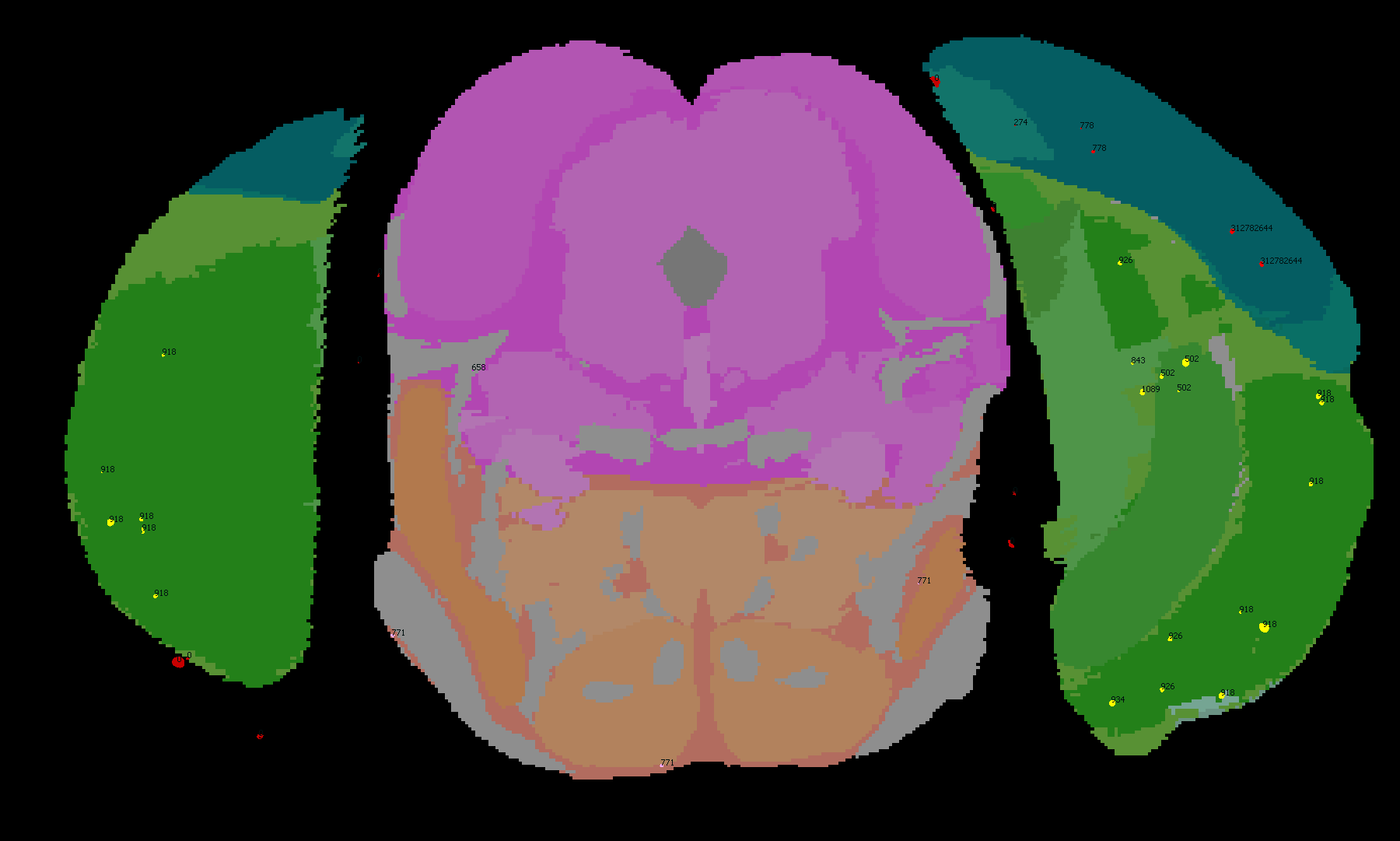

Supplement: Supplementary file 2 [file Data_Sheet_1.ZIP › Supplementary_material_Yates/pan-Abeta/tg2576_m287_4G8_s227_resize_Object Predictions.png]

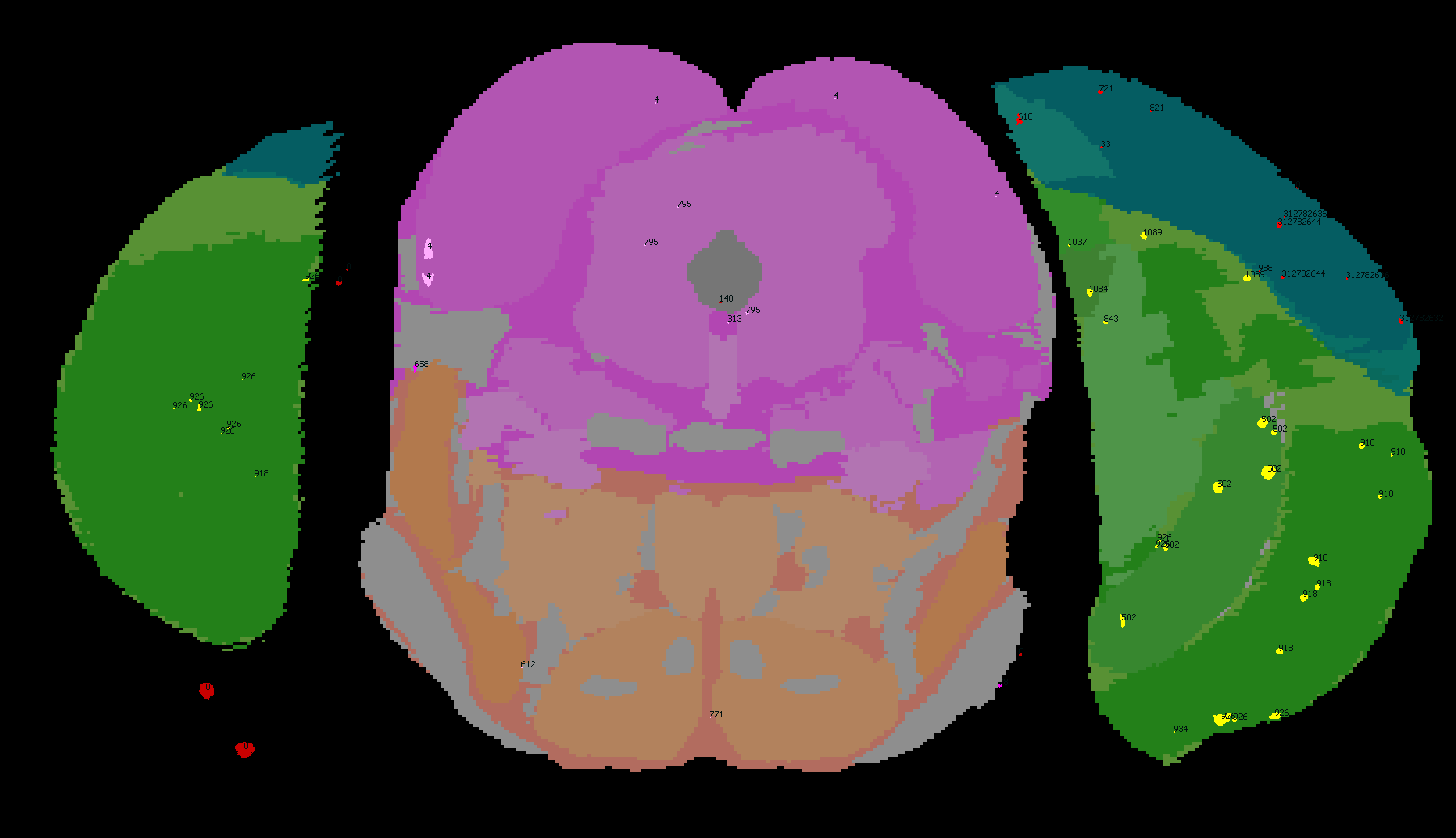

Supplement: Supplementary file 2 [file Data_Sheet_1.ZIP › Supplementary_material_Yates/pan-Abeta/tg2576_m287_4G8_s231_resize_Object Predictions.png]

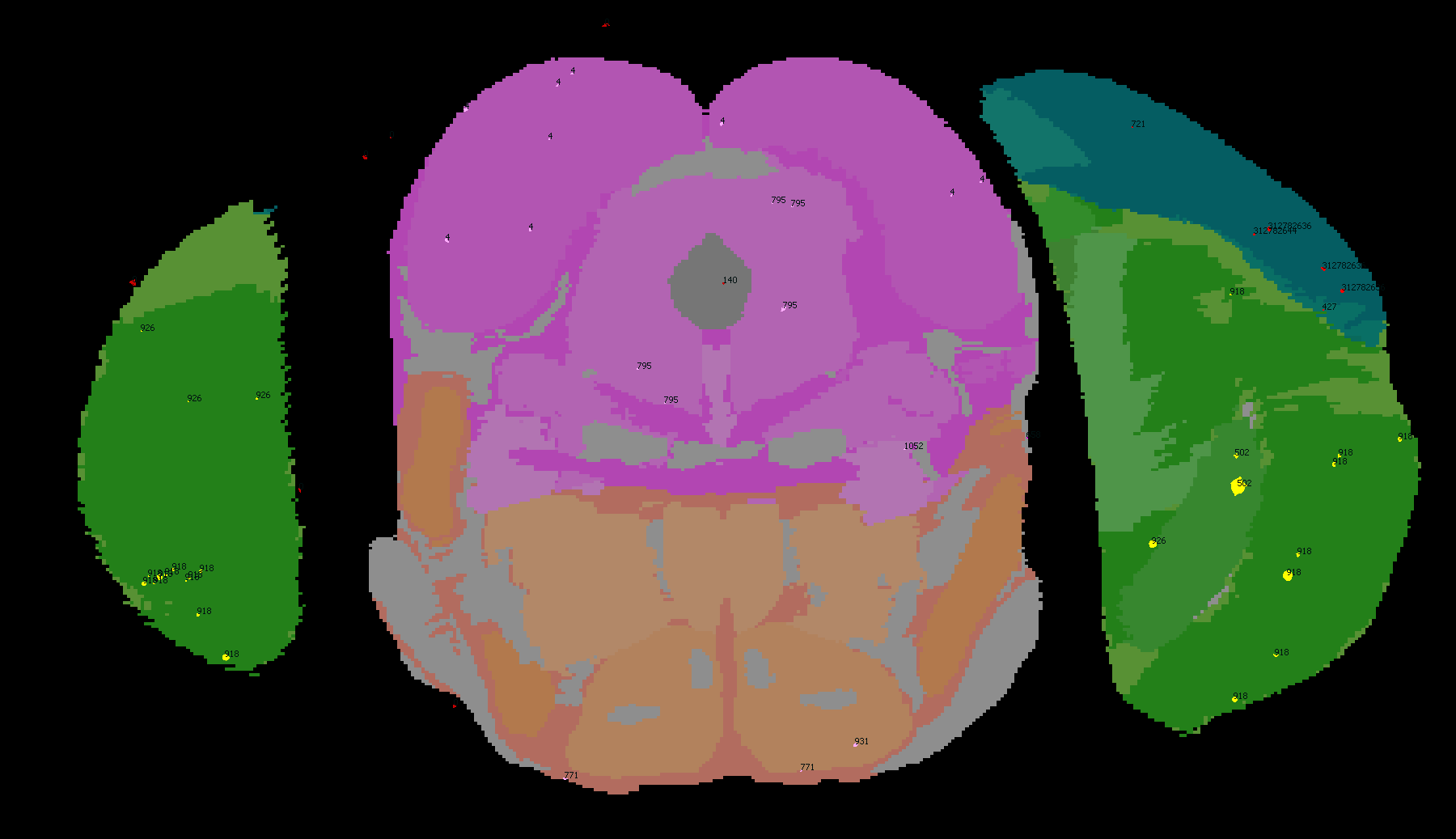

Supplement: Supplementary file 2 [file Data_Sheet_1.ZIP › Supplementary_material_Yates/pan-Abeta/tg2576_m287_4G8_s235_resize_Object Predictions.png]

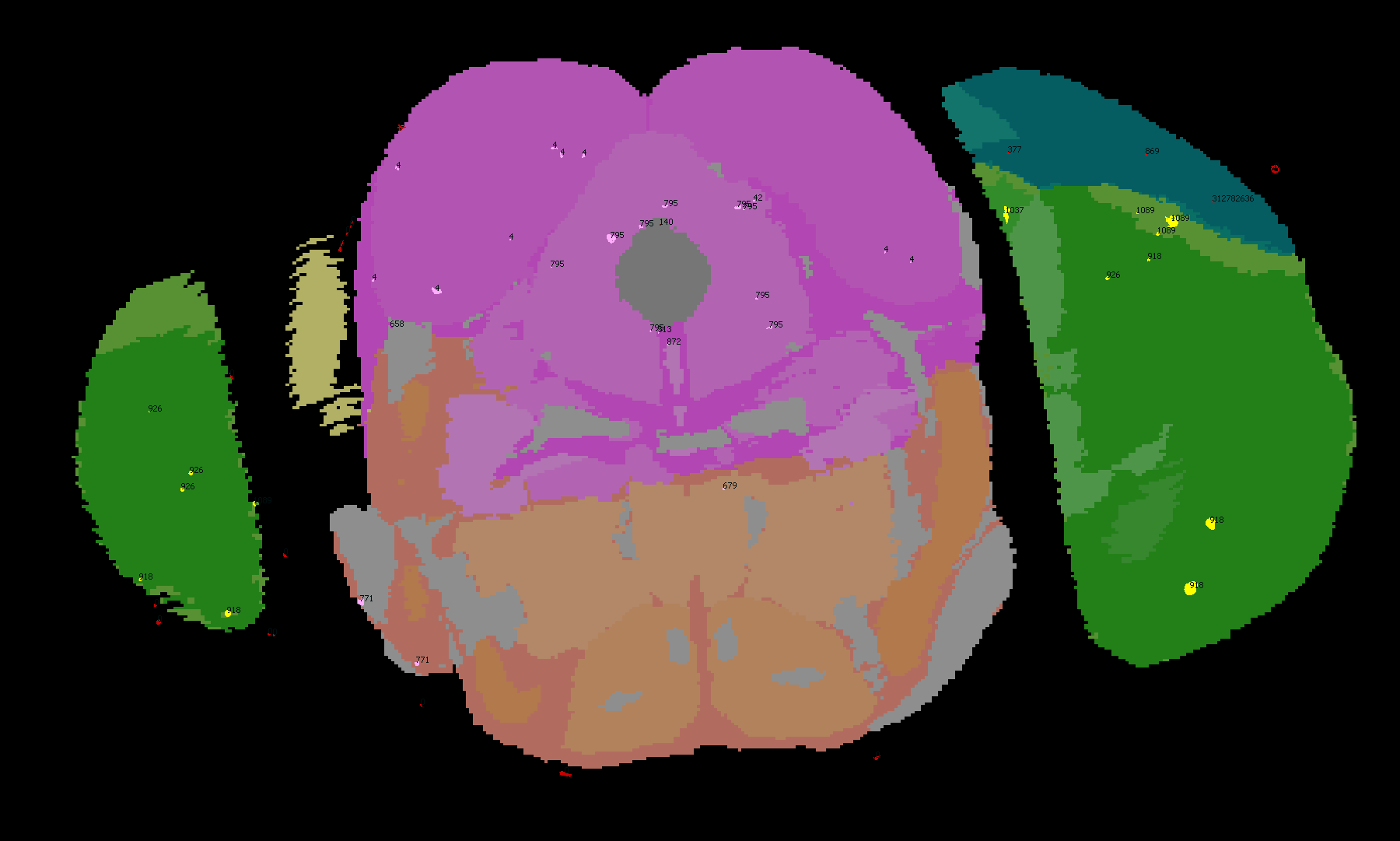

Supplement: Supplementary file 2 [file Data_Sheet_1.ZIP › Supplementary_material_Yates/pan-Abeta/tg2576_m287_4G8_s239_resize_Object Predictions.png]

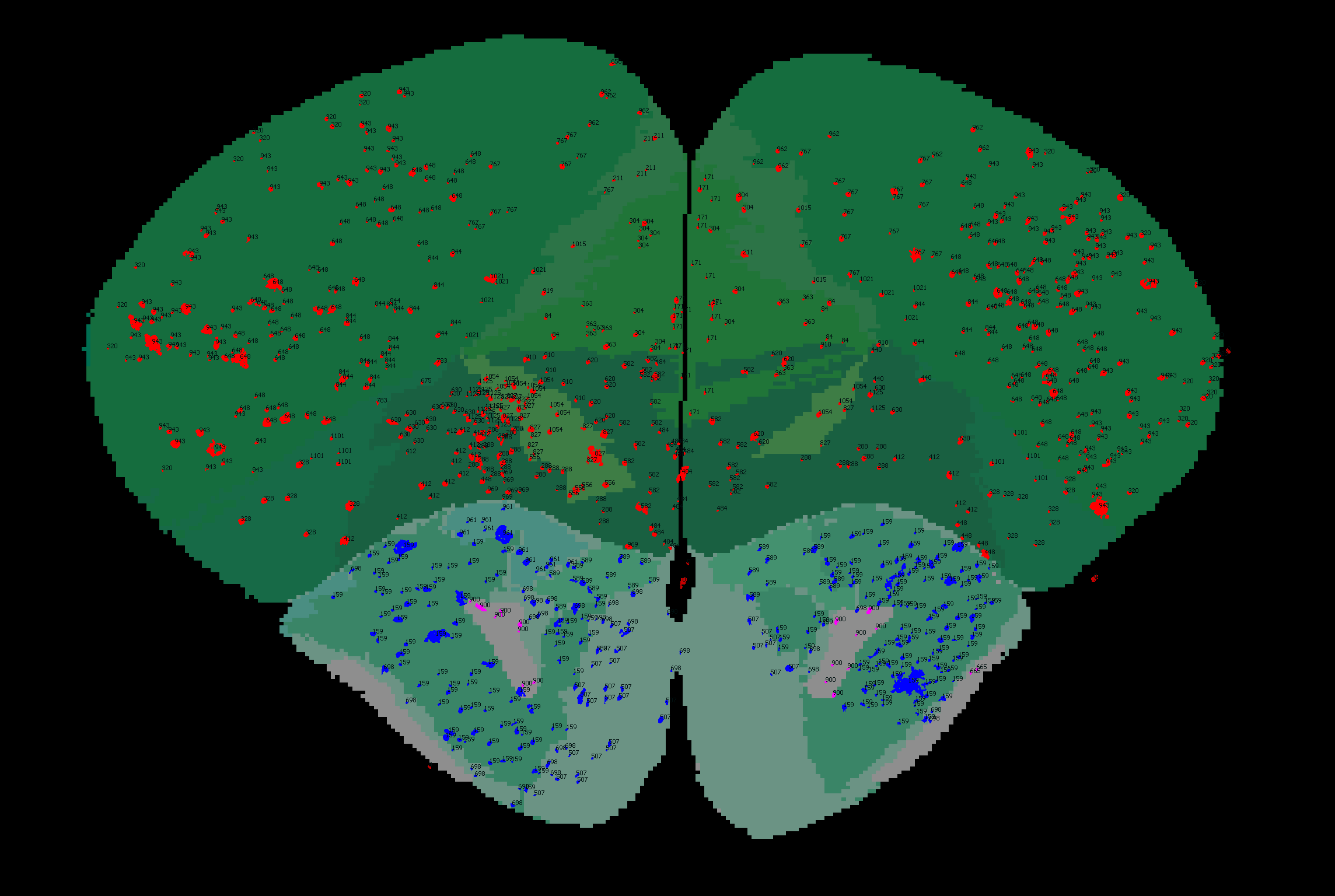

Supplement: Supplementary file 2 [file Data_Sheet_1.ZIP › Supplementary_material_Yates/pE-Abeta/tg2576_m287_pGlu_s004_Object Predictions.png]

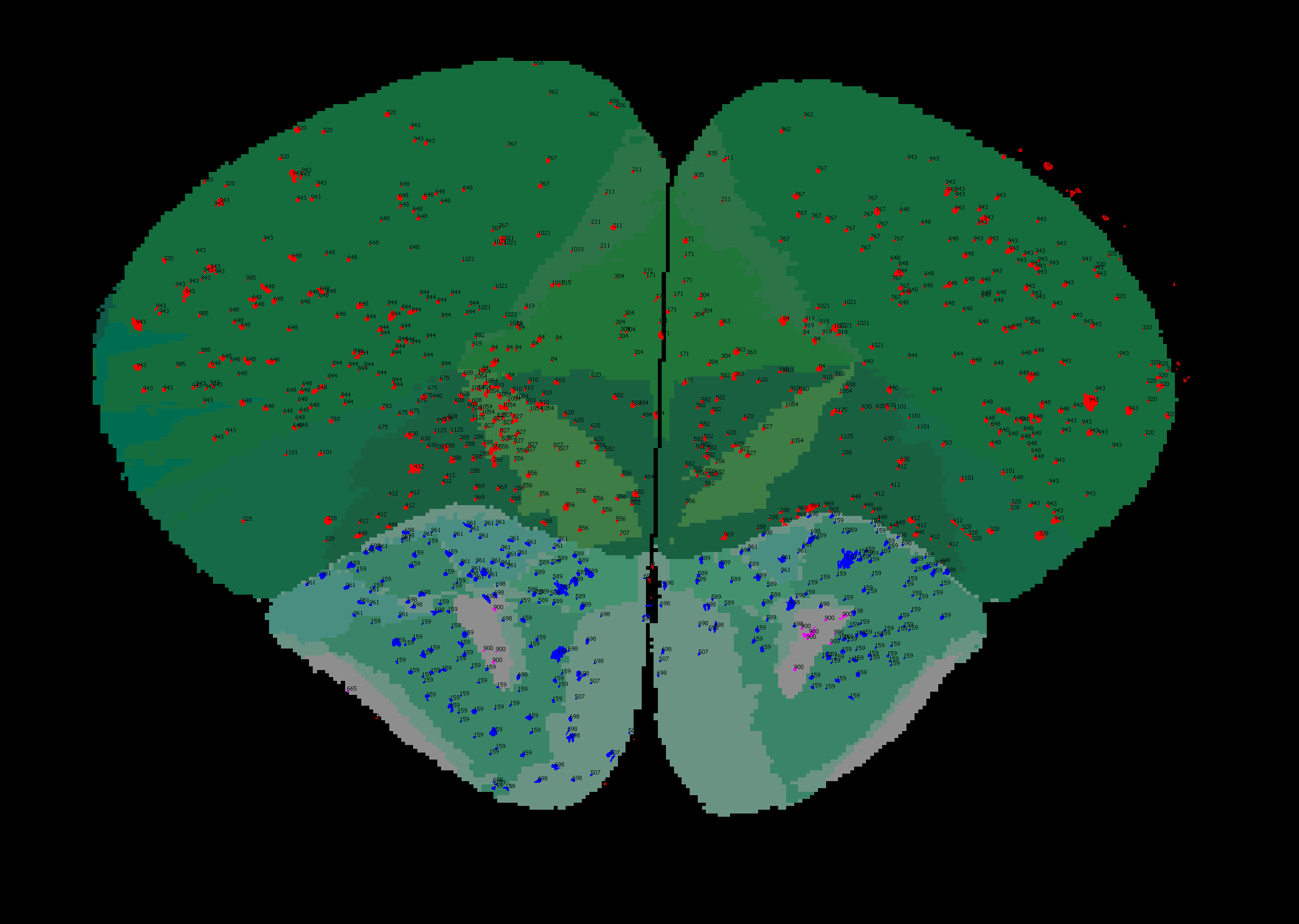

Supplement: Supplementary file 2 [file Data_Sheet_1.ZIP › Supplementary_material_Yates/pE-Abeta/tg2576_m287_pGlu_s008_Object Predictions.png]

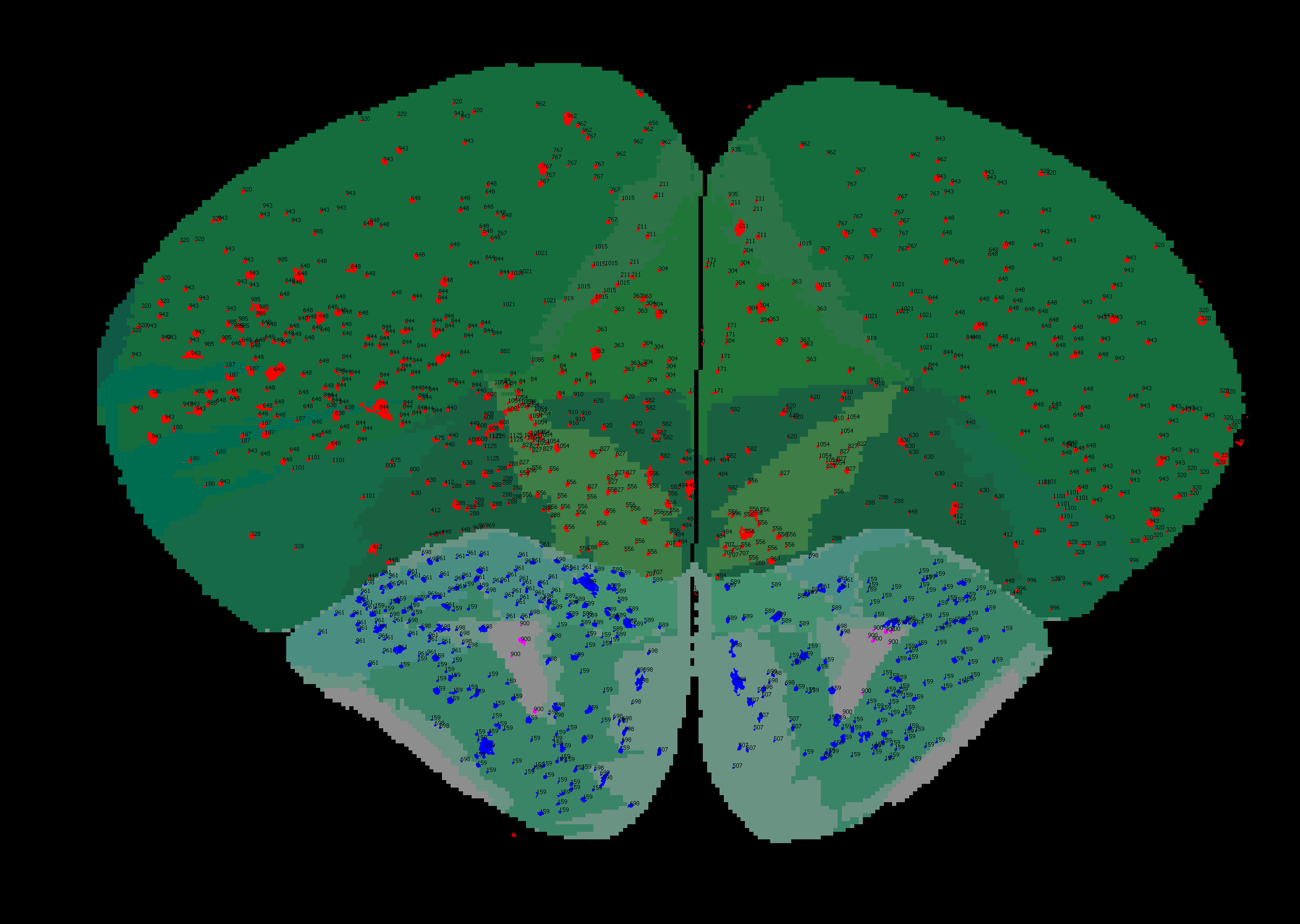

Supplement: Supplementary file 2 [file Data_Sheet_1.ZIP › Supplementary_material_Yates/pE-Abeta/tg2576_m287_pGlu_s012_Object Predictions.png]

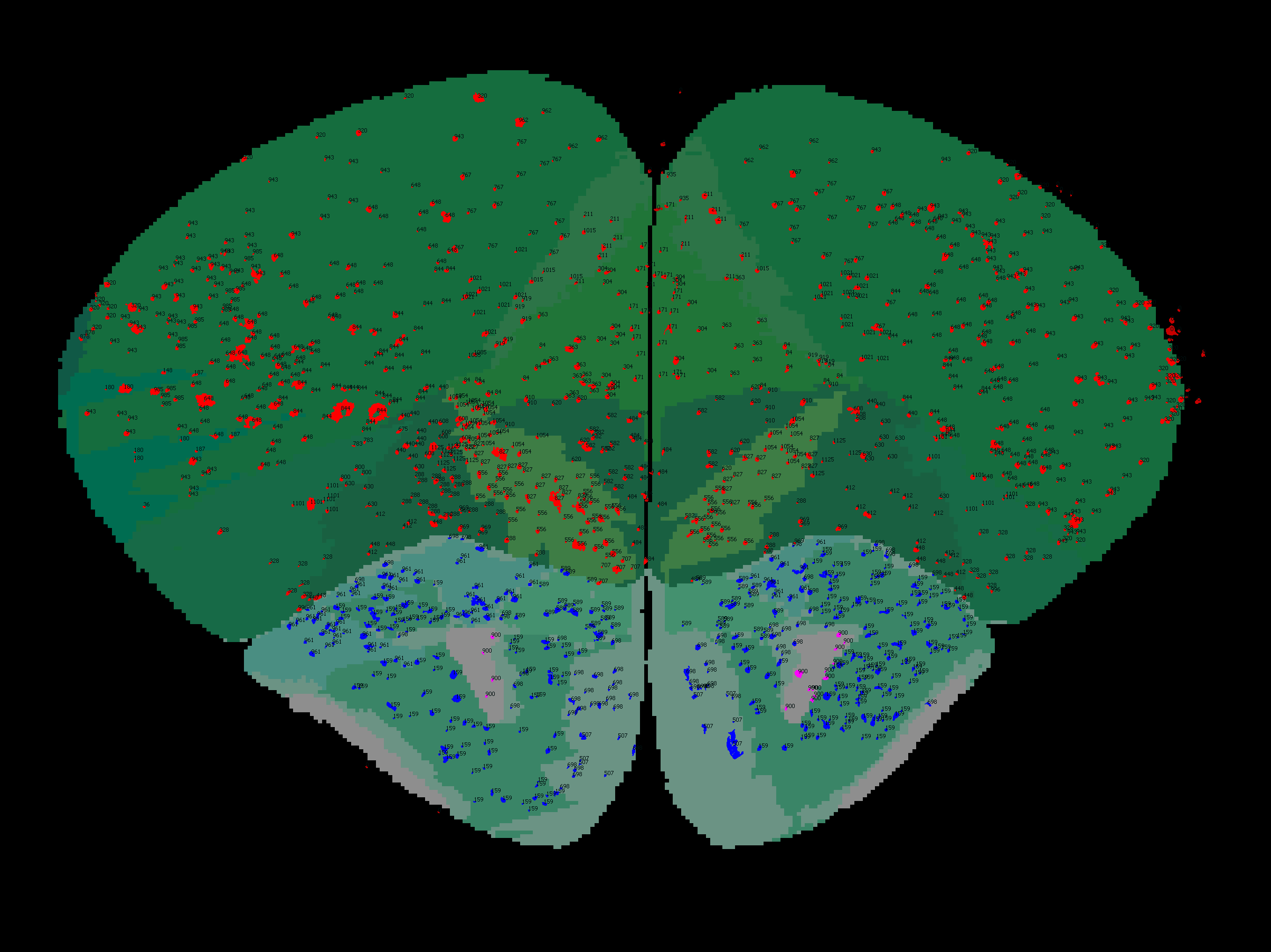

Supplement: Supplementary file 2 [file Data_Sheet_1.ZIP › Supplementary_material_Yates/pE-Abeta/tg2576_m287_pGlu_s016_Object Predictions.png]

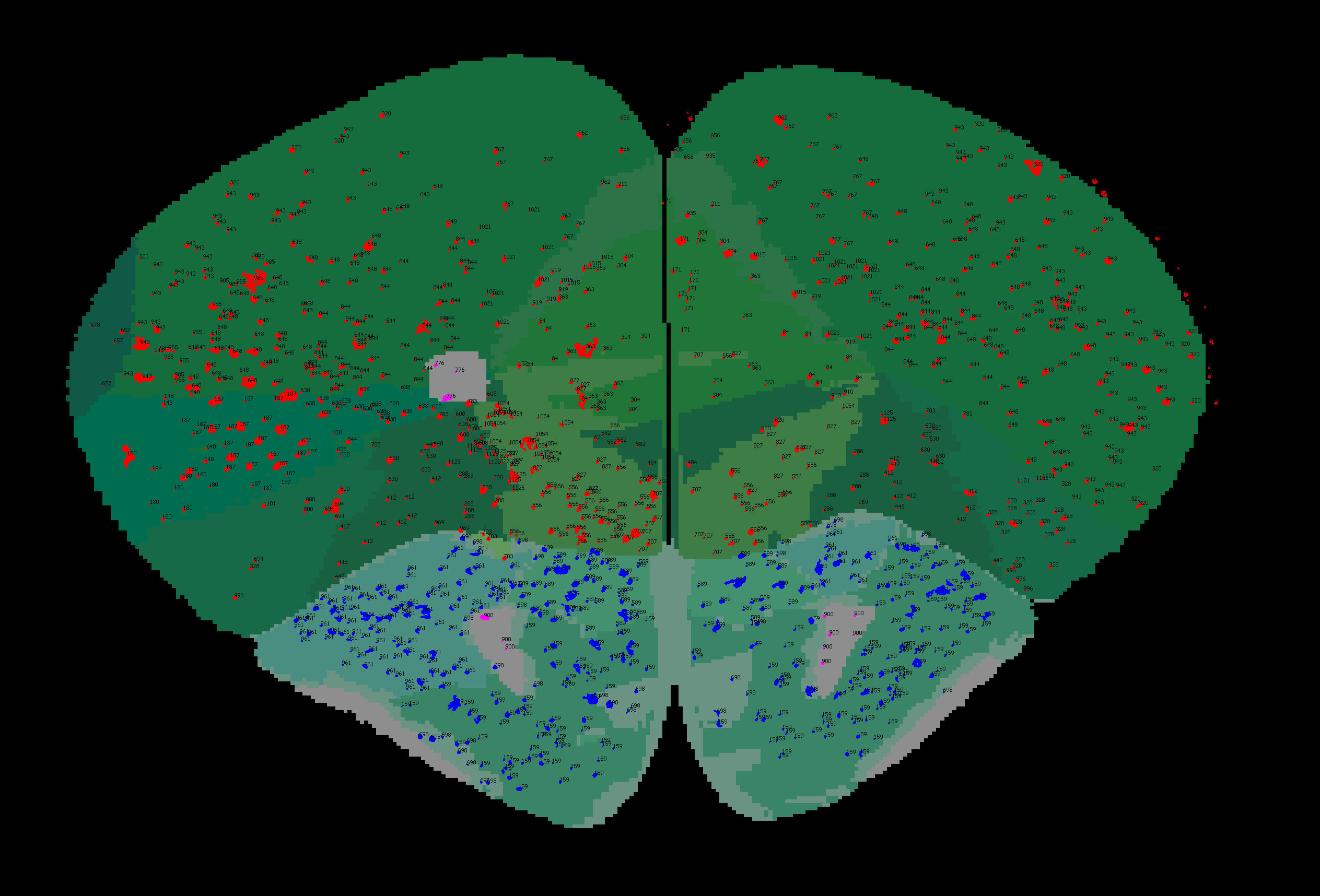

Supplement: Supplementary file 2 [file Data_Sheet_1.ZIP › Supplementary_material_Yates/pE-Abeta/tg2576_m287_pGlu_s020_Object Predictions.png]

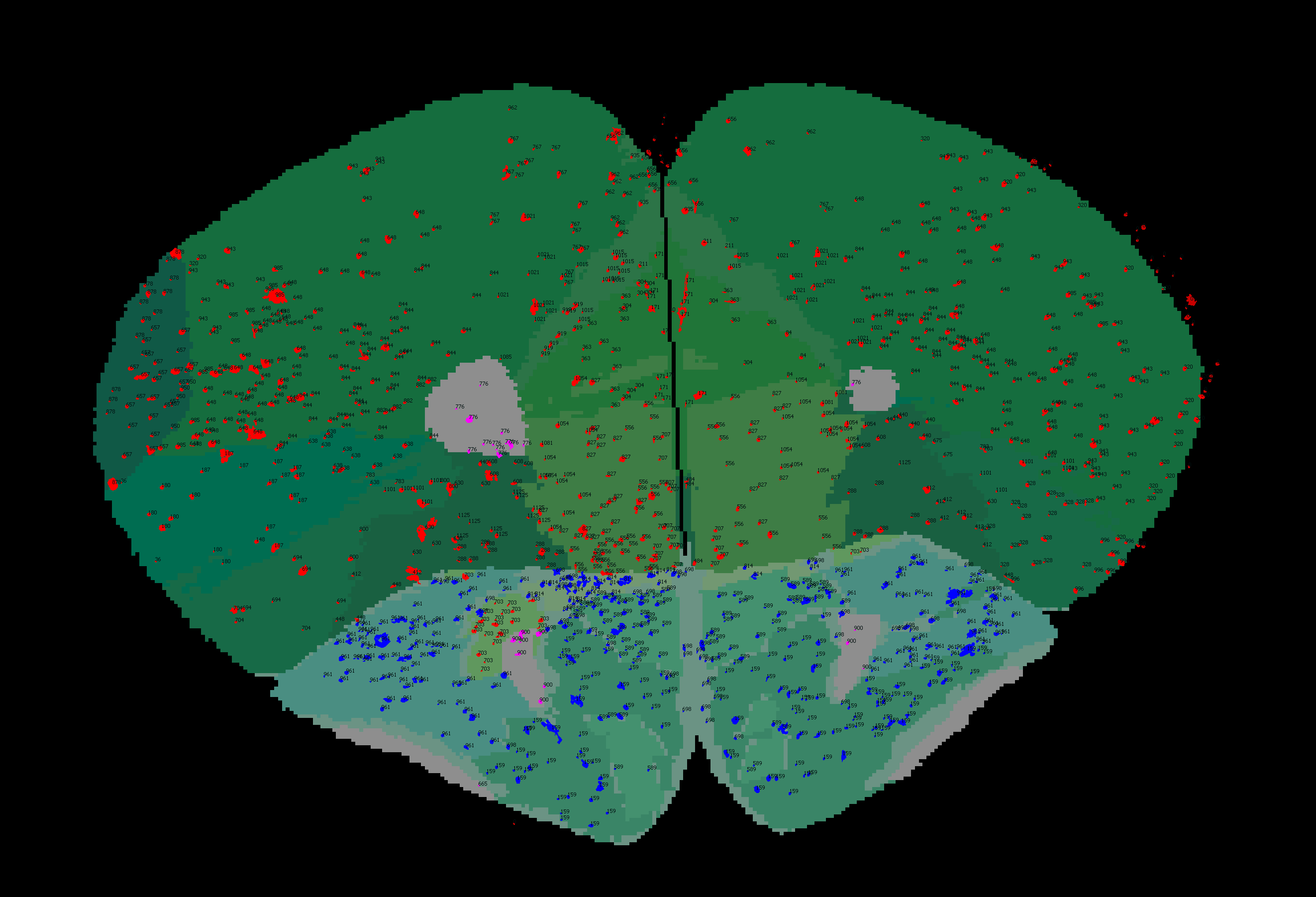

Supplement: Supplementary file 2 [file Data_Sheet_1.ZIP › Supplementary_material_Yates/pE-Abeta/tg2576_m287_pGlu_s024_Object Predictions.png]

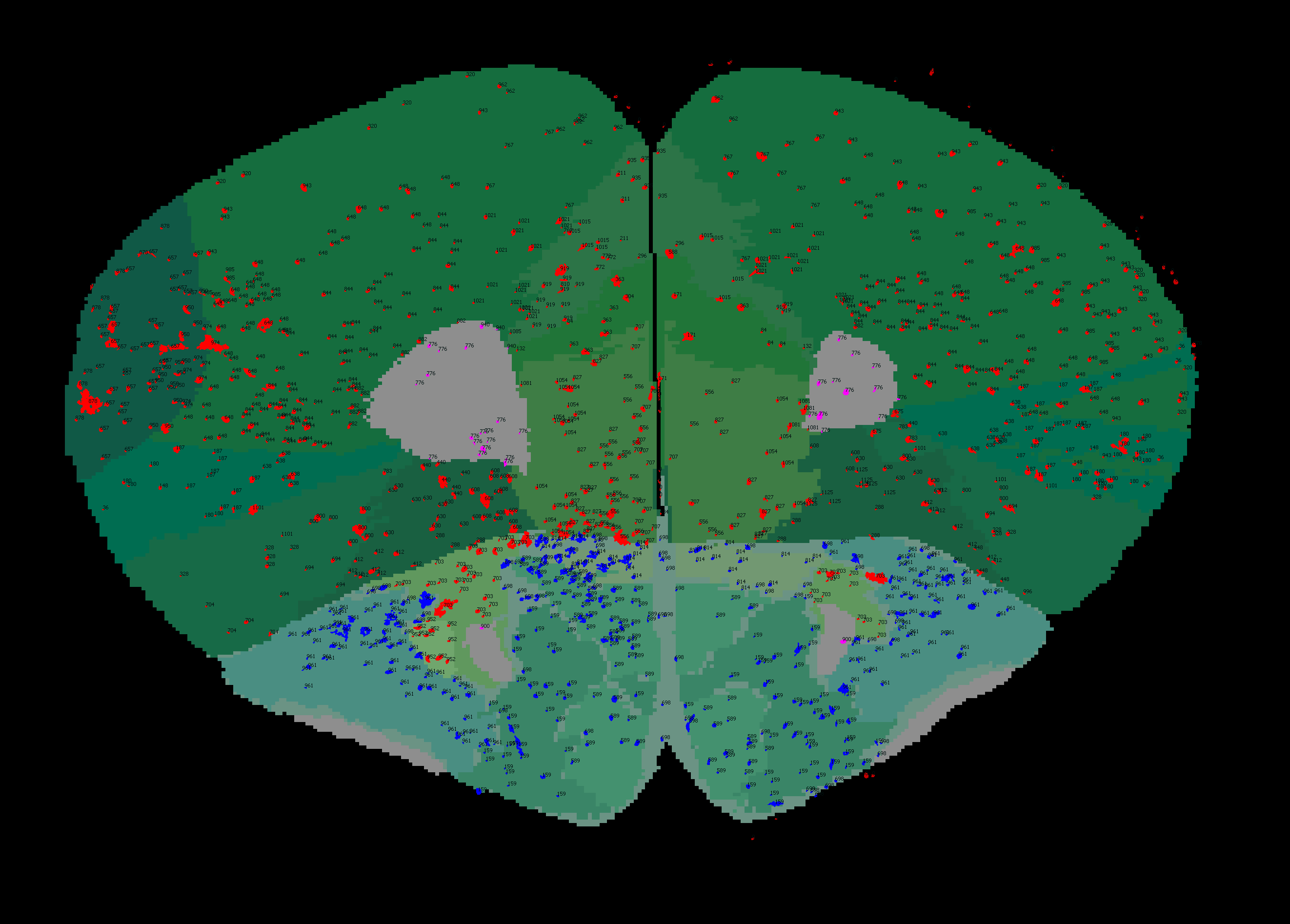

Supplement: Supplementary file 2 [file Data_Sheet_1.ZIP › Supplementary_material_Yates/pE-Abeta/tg2576_m287_pGlu_s028_Object Predictions.png]

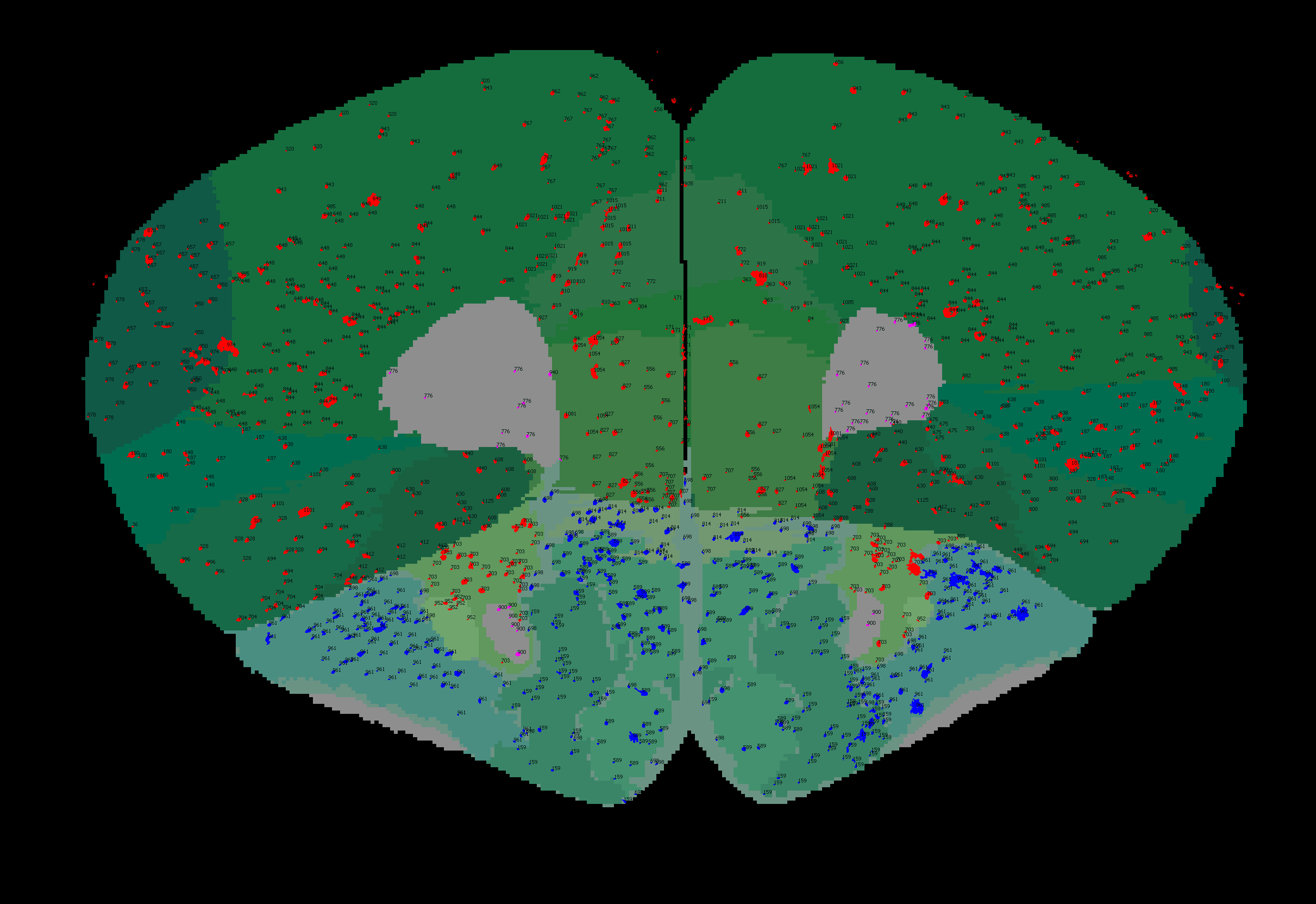

Supplement: Supplementary file 2 [file Data_Sheet_1.ZIP › Supplementary_material_Yates/pE-Abeta/tg2576_m287_pGlu_s032_Object Predictions.png]

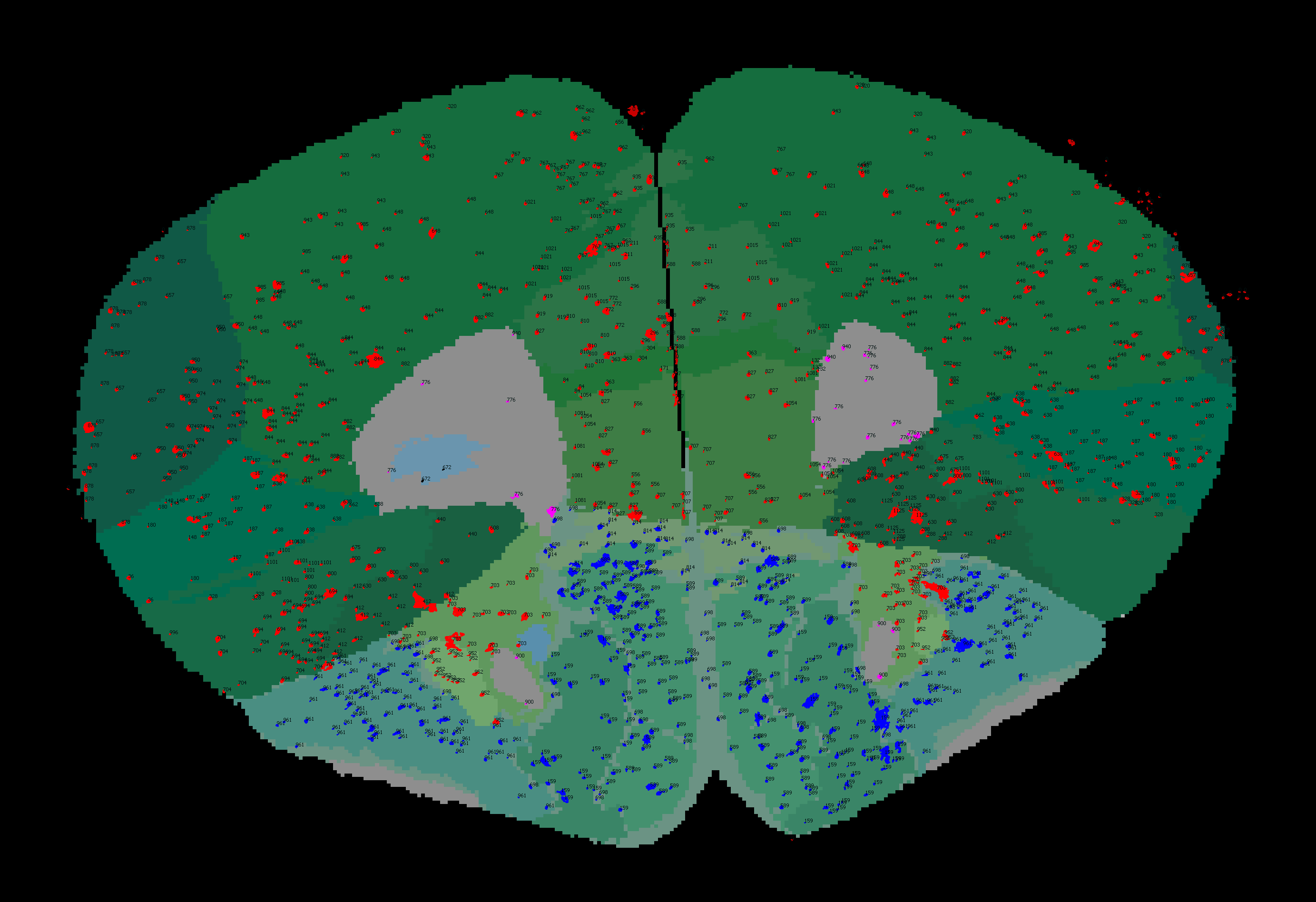

Supplement: Supplementary file 2 [file Data_Sheet_1.ZIP › Supplementary_material_Yates/pE-Abeta/tg2576_m287_pGlu_s036_Object Predictions.png]

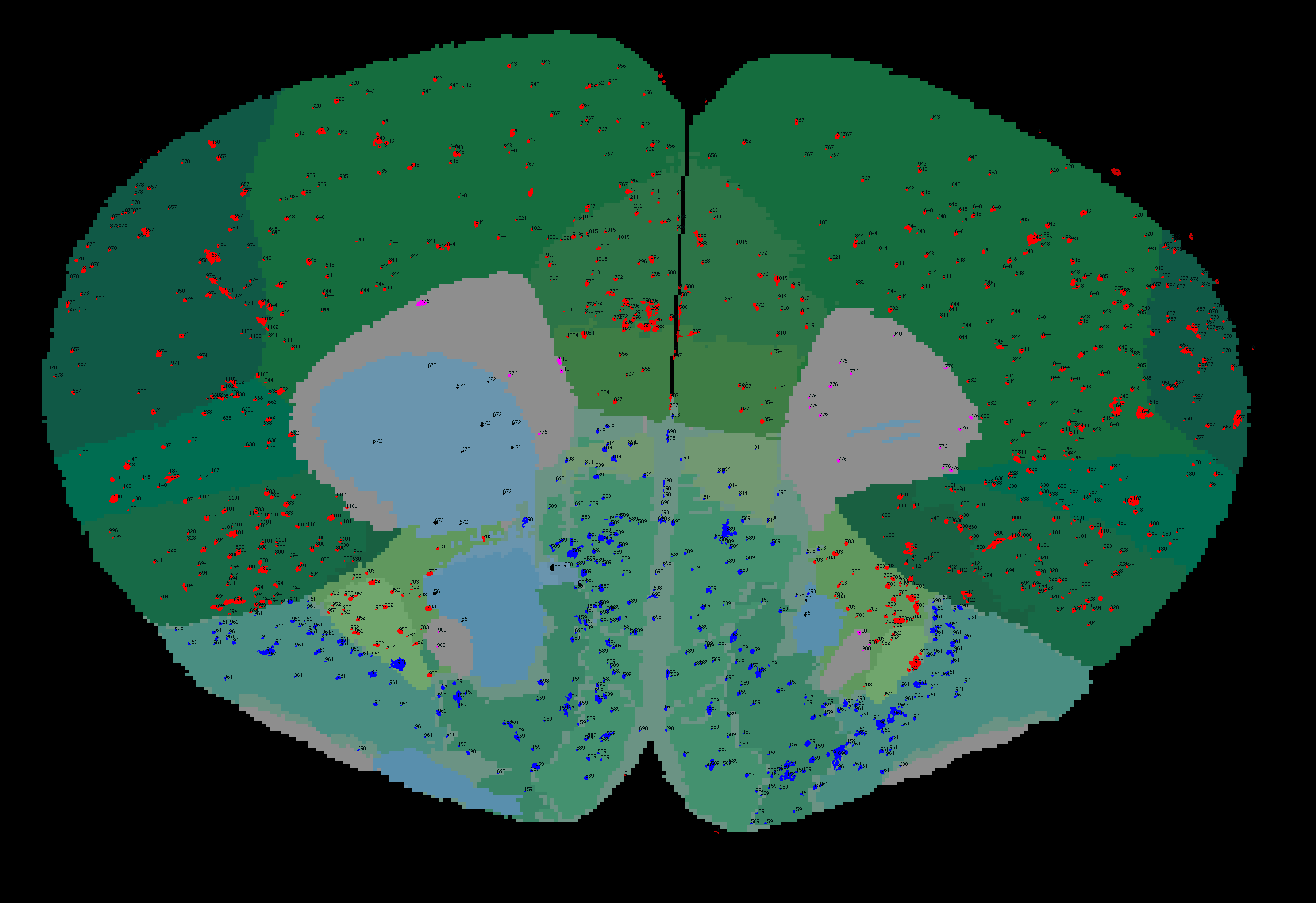

Supplement: Supplementary file 2 [file Data_Sheet_1.ZIP › Supplementary_material_Yates/pE-Abeta/tg2576_m287_pGlu_s040_Object Predictions.png]

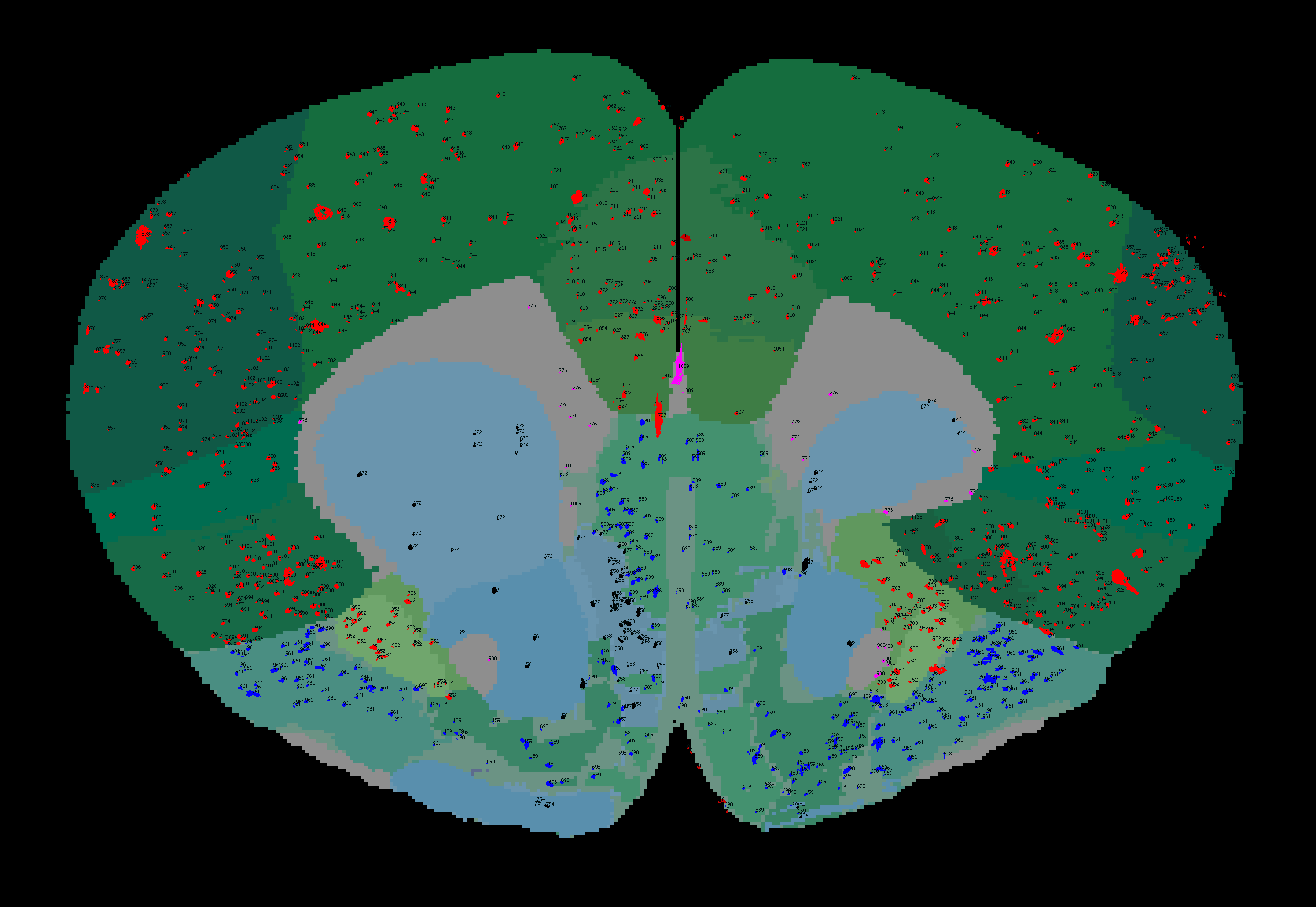

Supplement: Supplementary file 2 [file Data_Sheet_1.ZIP › Supplementary_material_Yates/pE-Abeta/tg2576_m287_pGlu_s044_Object Predictions.png]

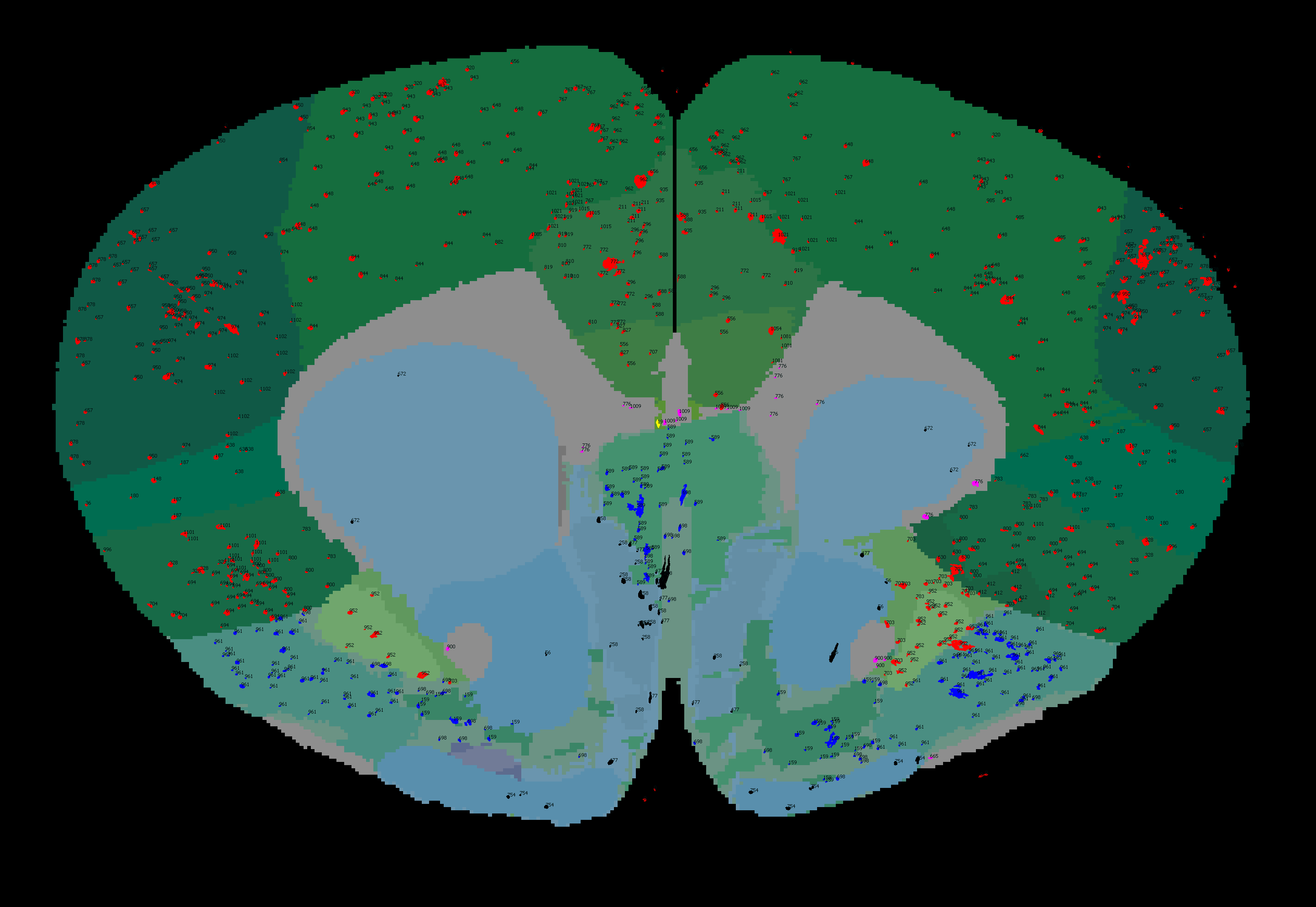

Supplement: Supplementary file 2 [file Data_Sheet_1.ZIP › Supplementary_material_Yates/pE-Abeta/tg2576_m287_pGlu_s048_Object Predictions.png]

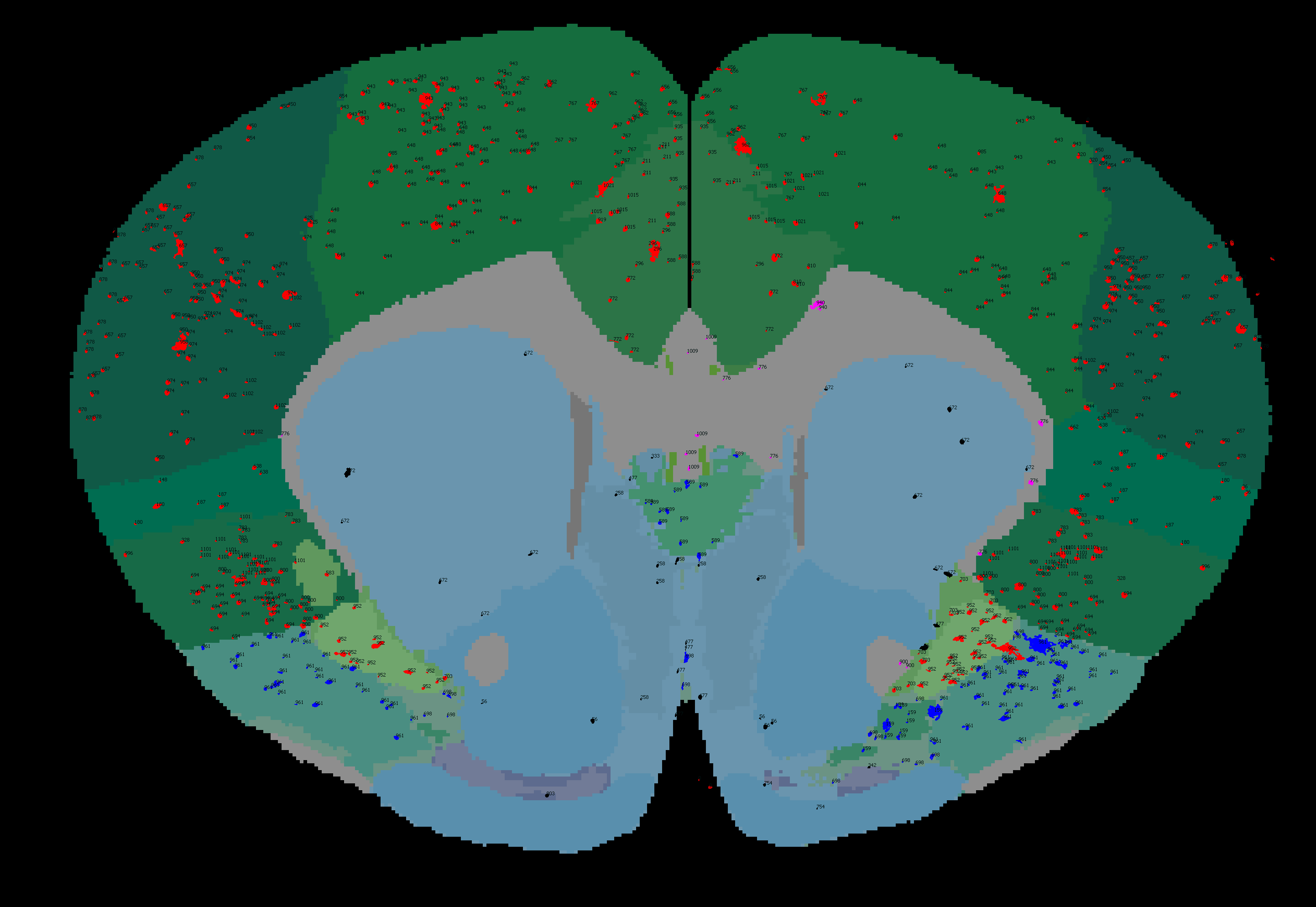

Supplement: Supplementary file 2 [file Data_Sheet_1.ZIP › Supplementary_material_Yates/pE-Abeta/tg2576_m287_pGlu_s052_Object Predictions.png]

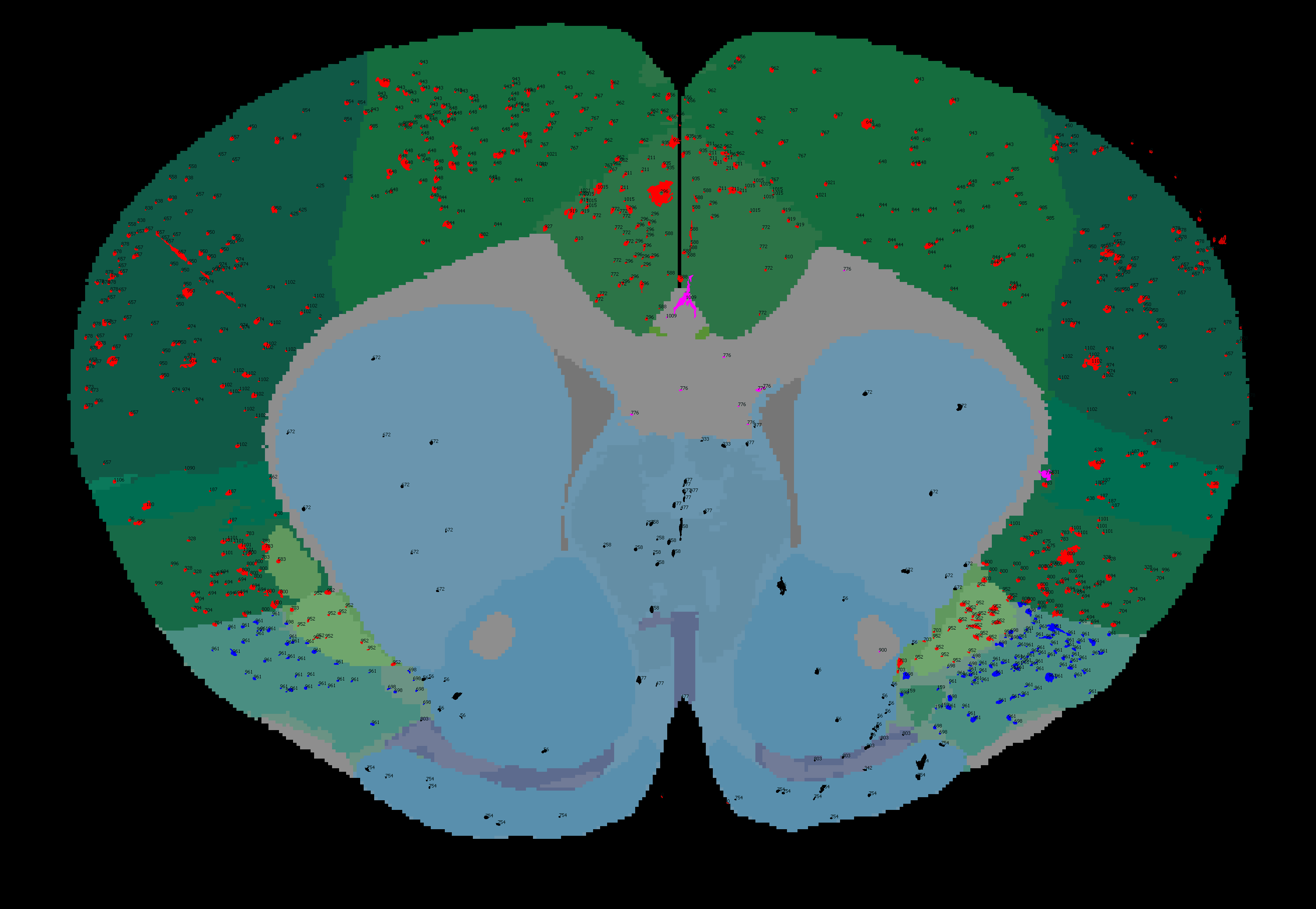

Supplement: Supplementary file 2 [file Data_Sheet_1.ZIP › Supplementary_material_Yates/pE-Abeta/tg2576_m287_pGlu_s056_Object Predictions.png]

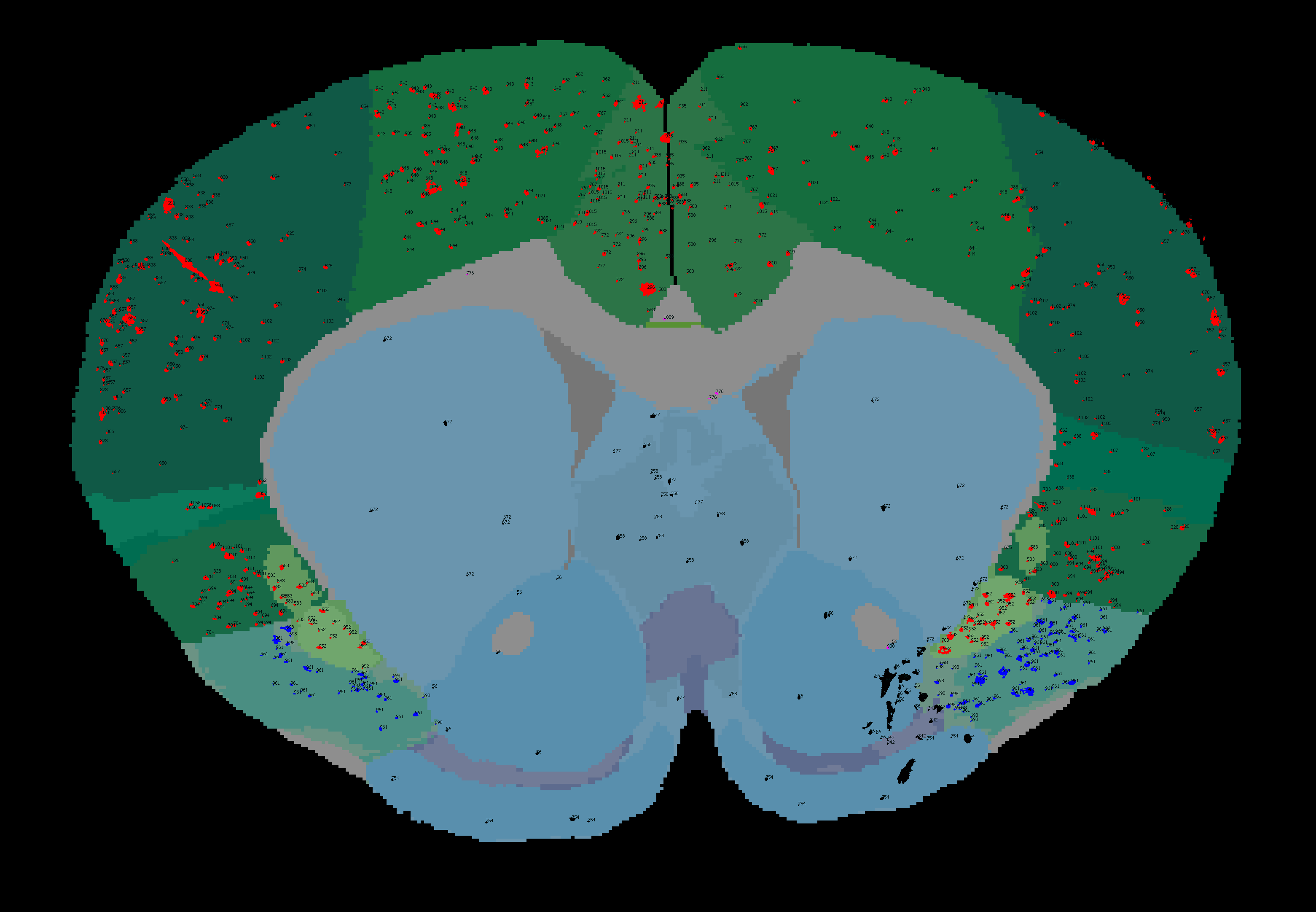

Supplement: Supplementary file 2 [file Data_Sheet_1.ZIP › Supplementary_material_Yates/pE-Abeta/tg2576_m287_pGlu_s060_Object Predictions.png]

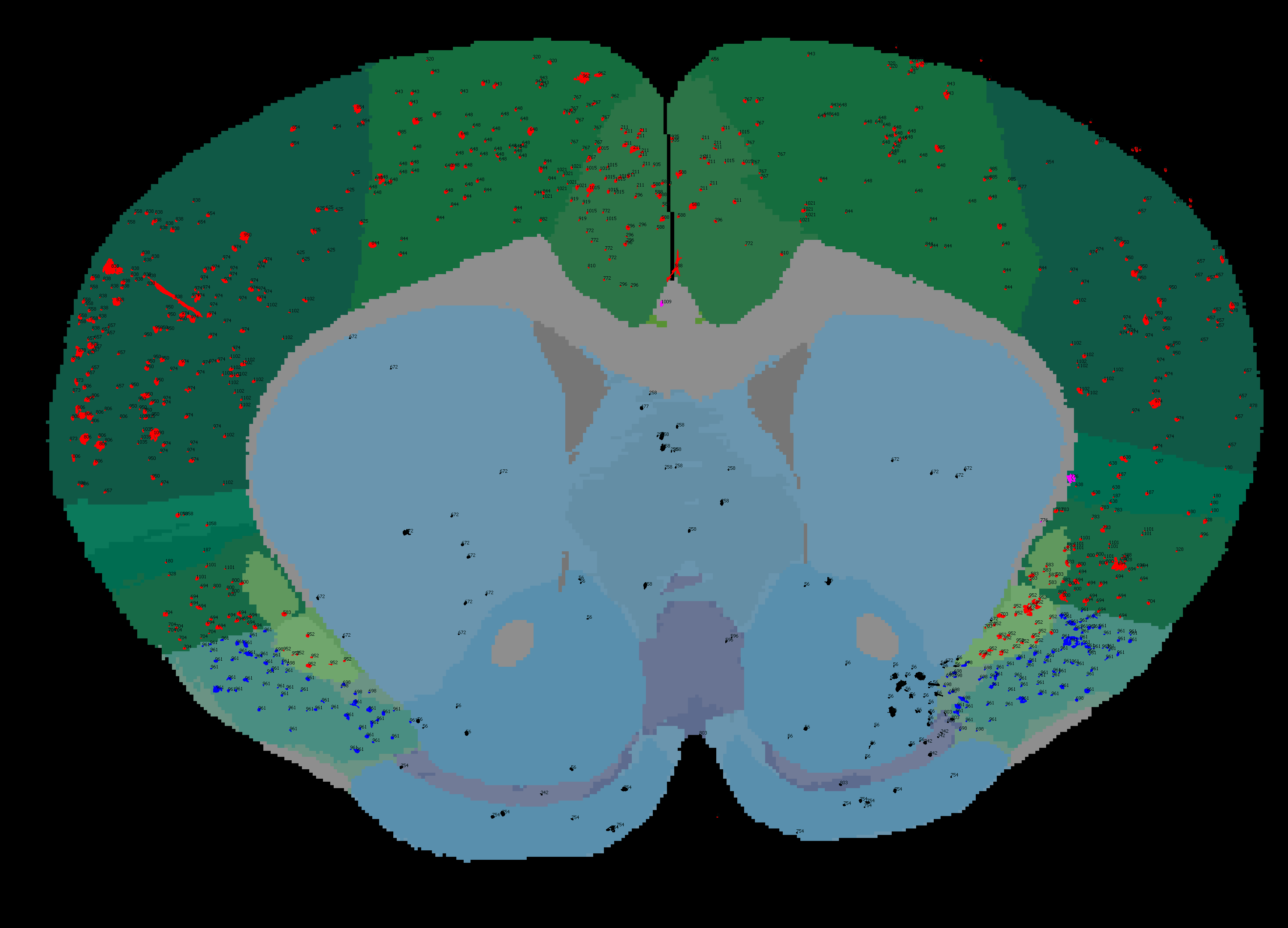

Supplement: Supplementary file 2 [file Data_Sheet_1.ZIP › Supplementary_material_Yates/pE-Abeta/tg2576_m287_pGlu_s064_Object Predictions.png]

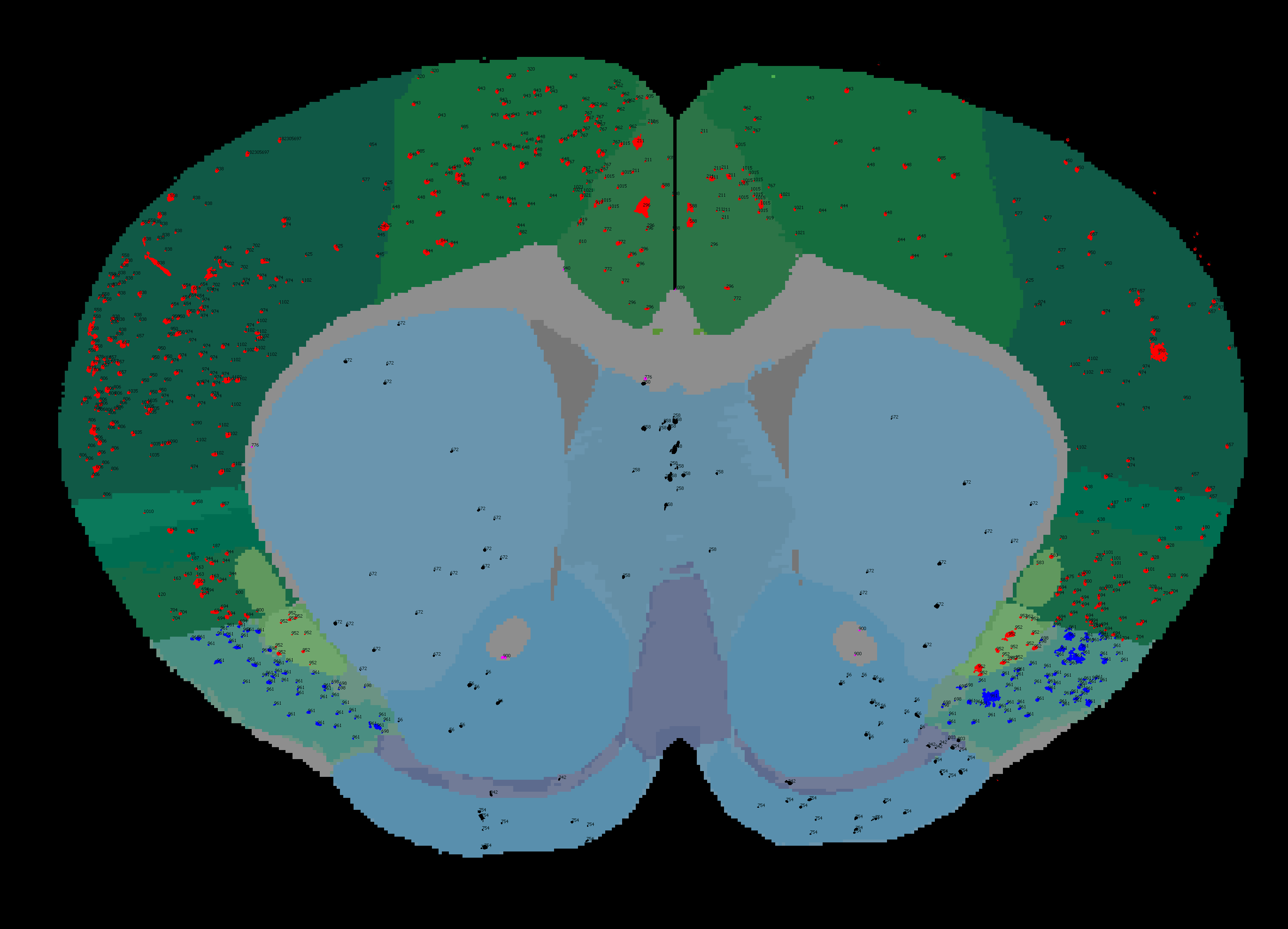

Supplement: Supplementary file 2 [file Data_Sheet_1.ZIP › Supplementary_material_Yates/pE-Abeta/tg2576_m287_pGlu_s068_Object Predictions.png]

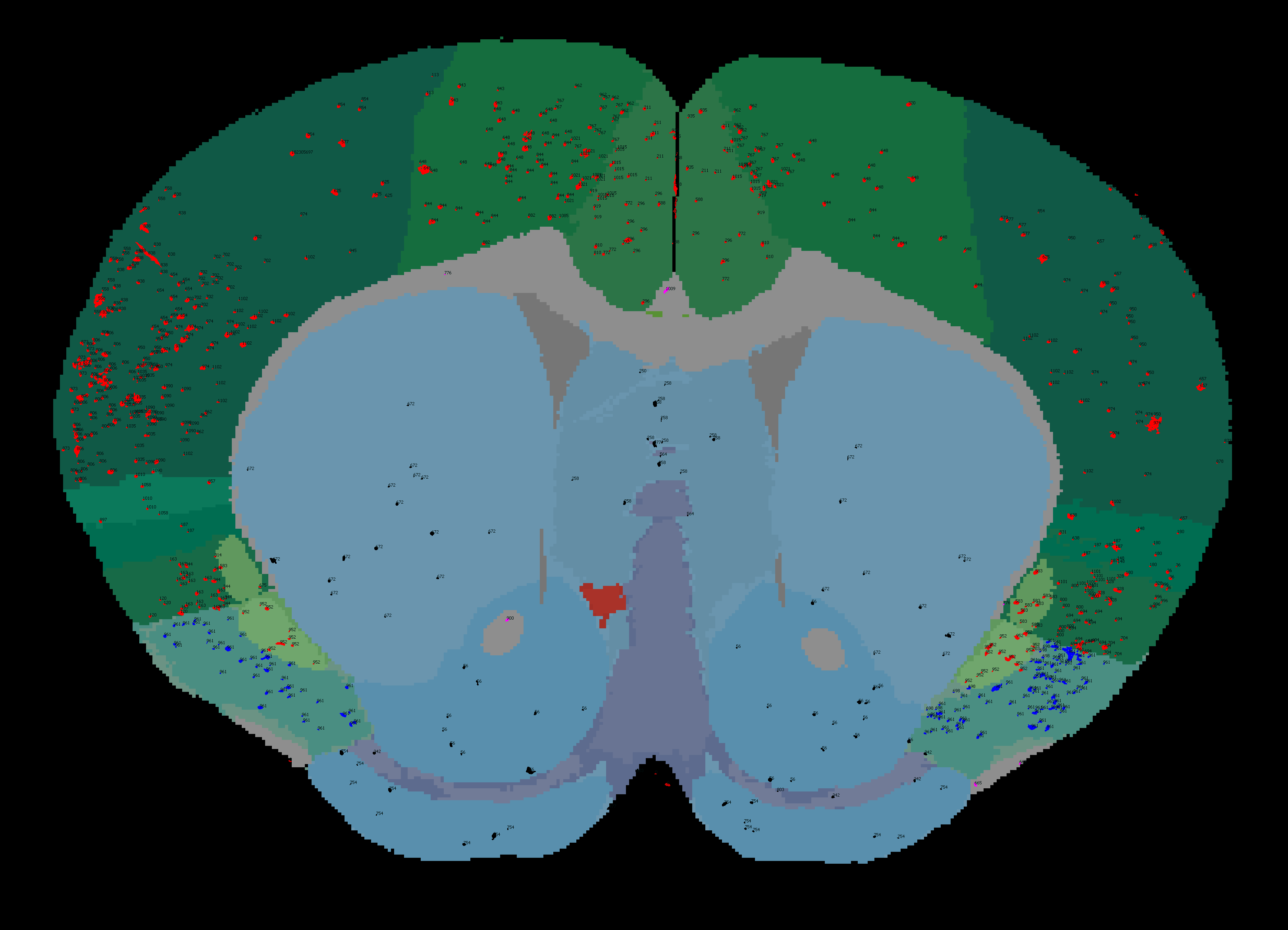

Supplement: Supplementary file 2 [file Data_Sheet_1.ZIP › Supplementary_material_Yates/pE-Abeta/tg2576_m287_pGlu_s072_Object Predictions.png]

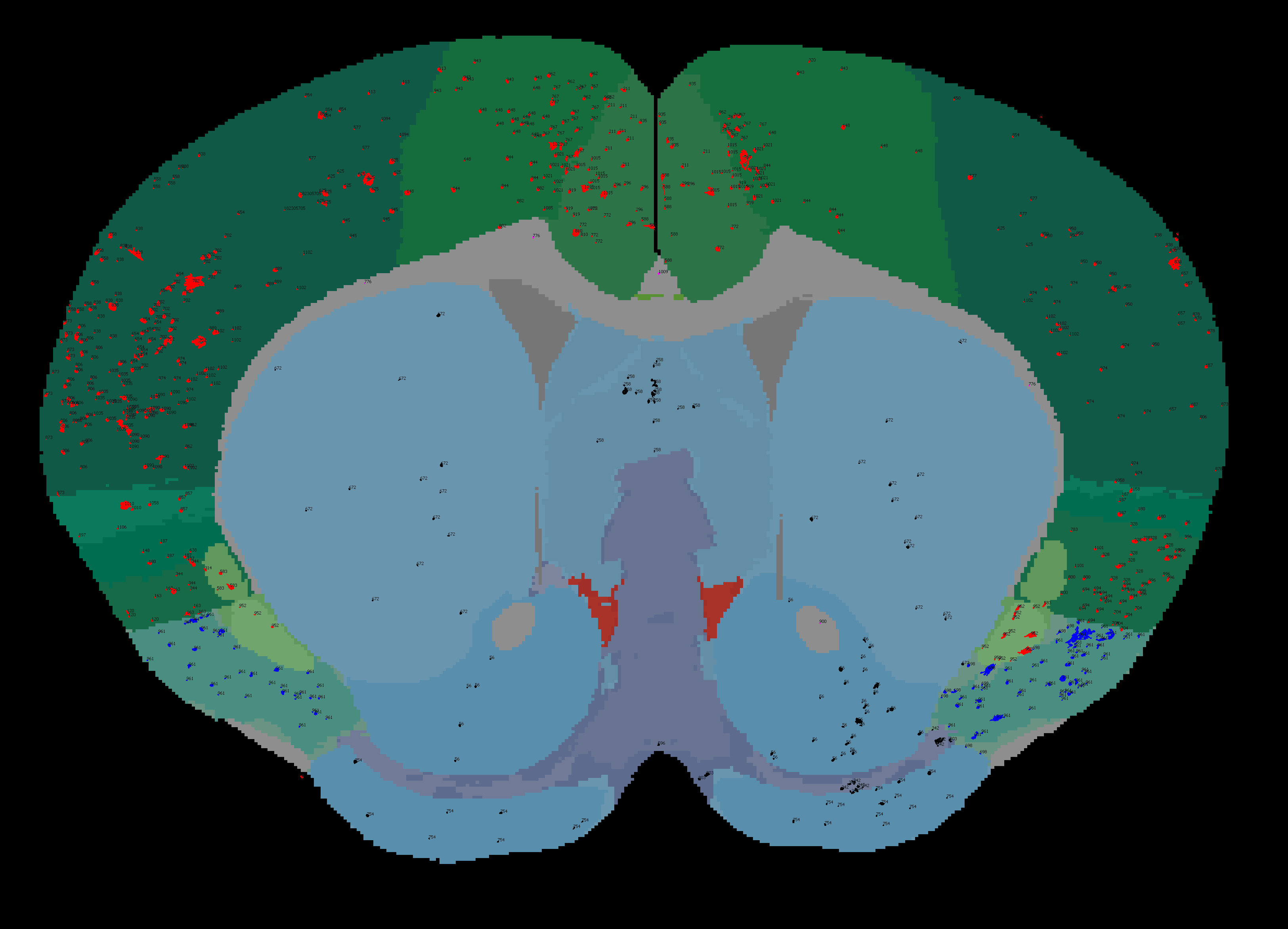

Supplement: Supplementary file 2 [file Data_Sheet_1.ZIP › Supplementary_material_Yates/pE-Abeta/tg2576_m287_pGlu_s076_Object Predictions.png]

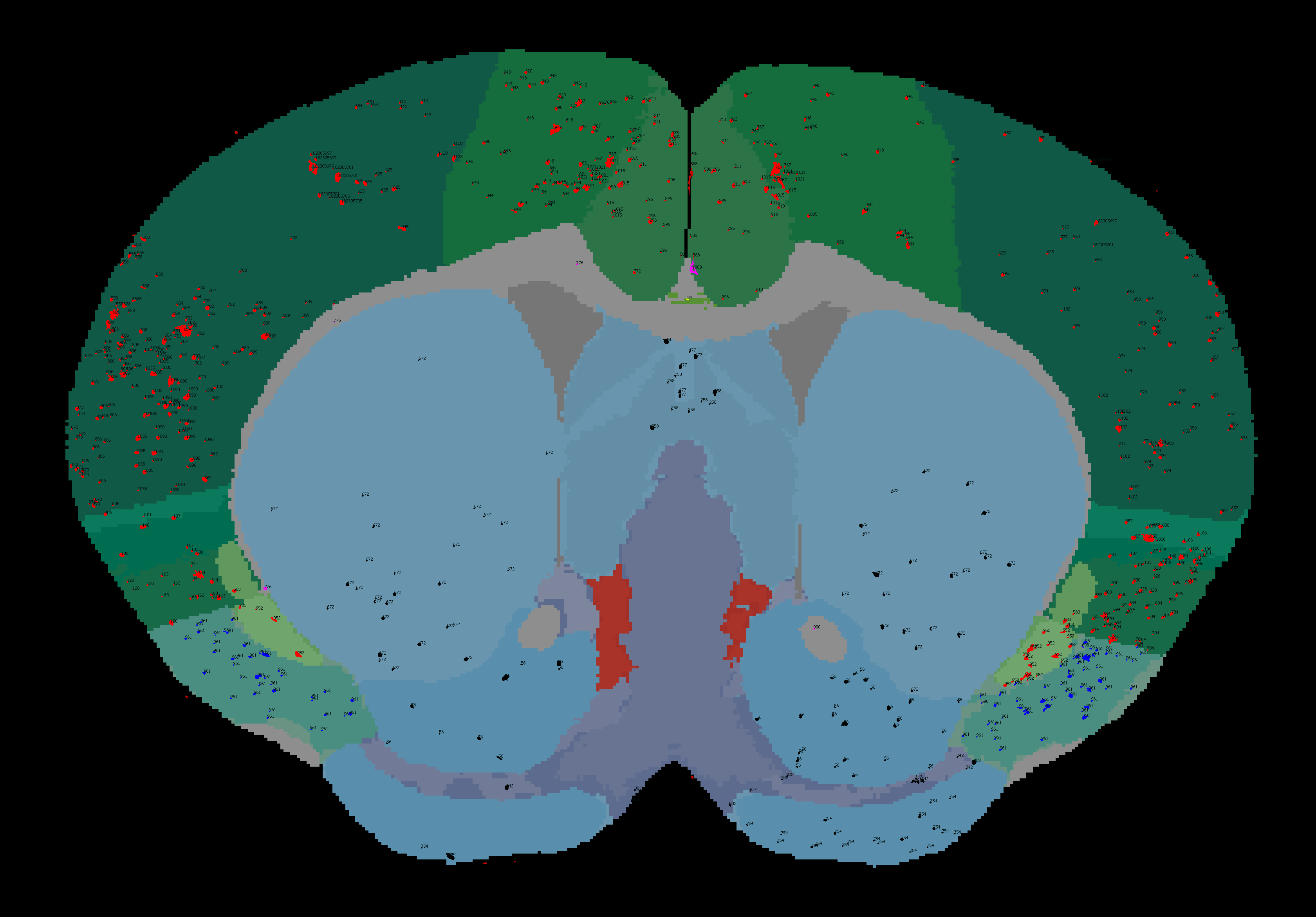

Supplement: Supplementary file 2 [file Data_Sheet_1.ZIP › Supplementary_material_Yates/pE-Abeta/tg2576_m287_pGlu_s080_Object Predictions.png]

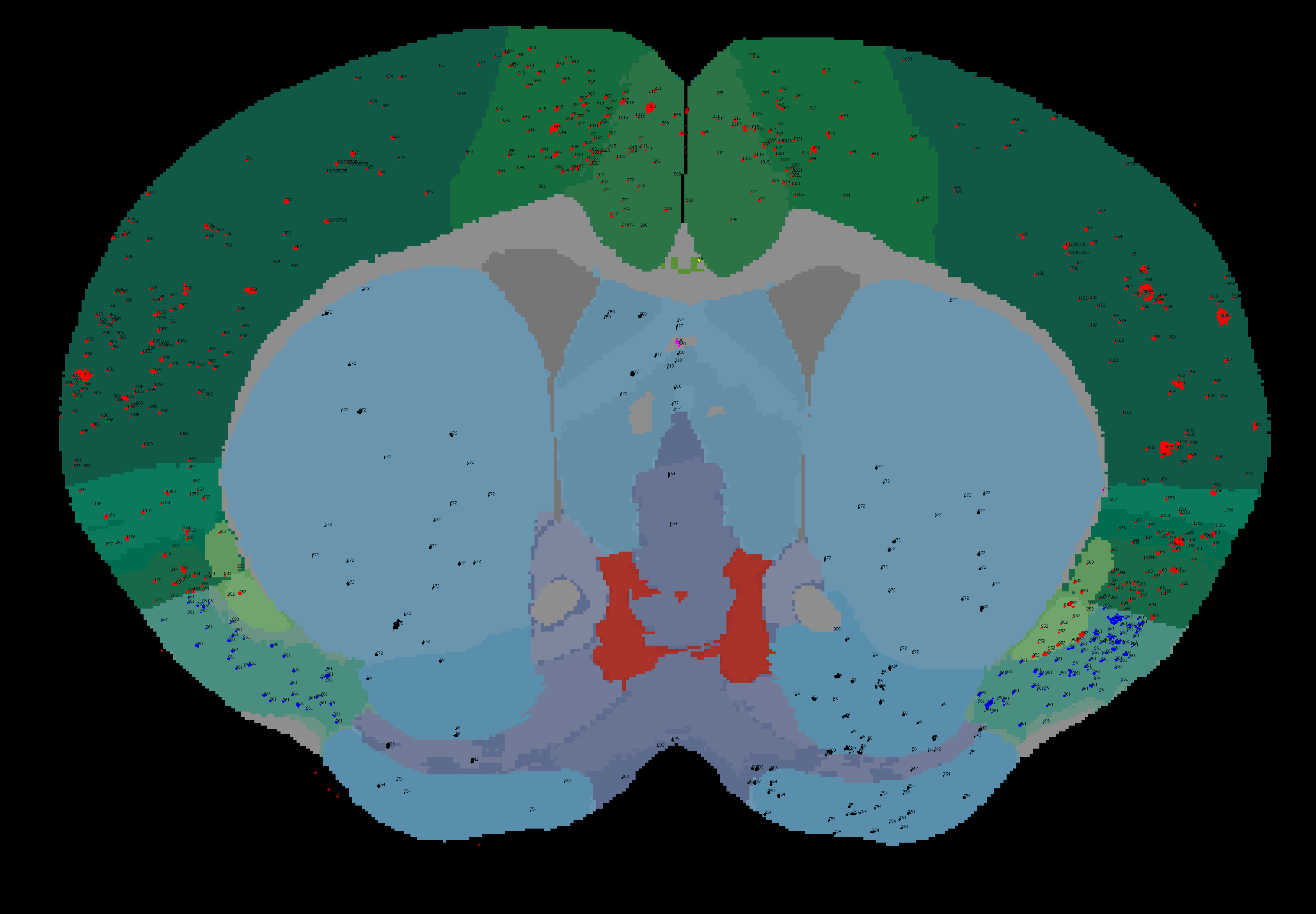

Supplement: Supplementary file 2 [file Data_Sheet_1.ZIP › Supplementary_material_Yates/pE-Abeta/tg2576_m287_pGlu_s084_Object Predictions.png]

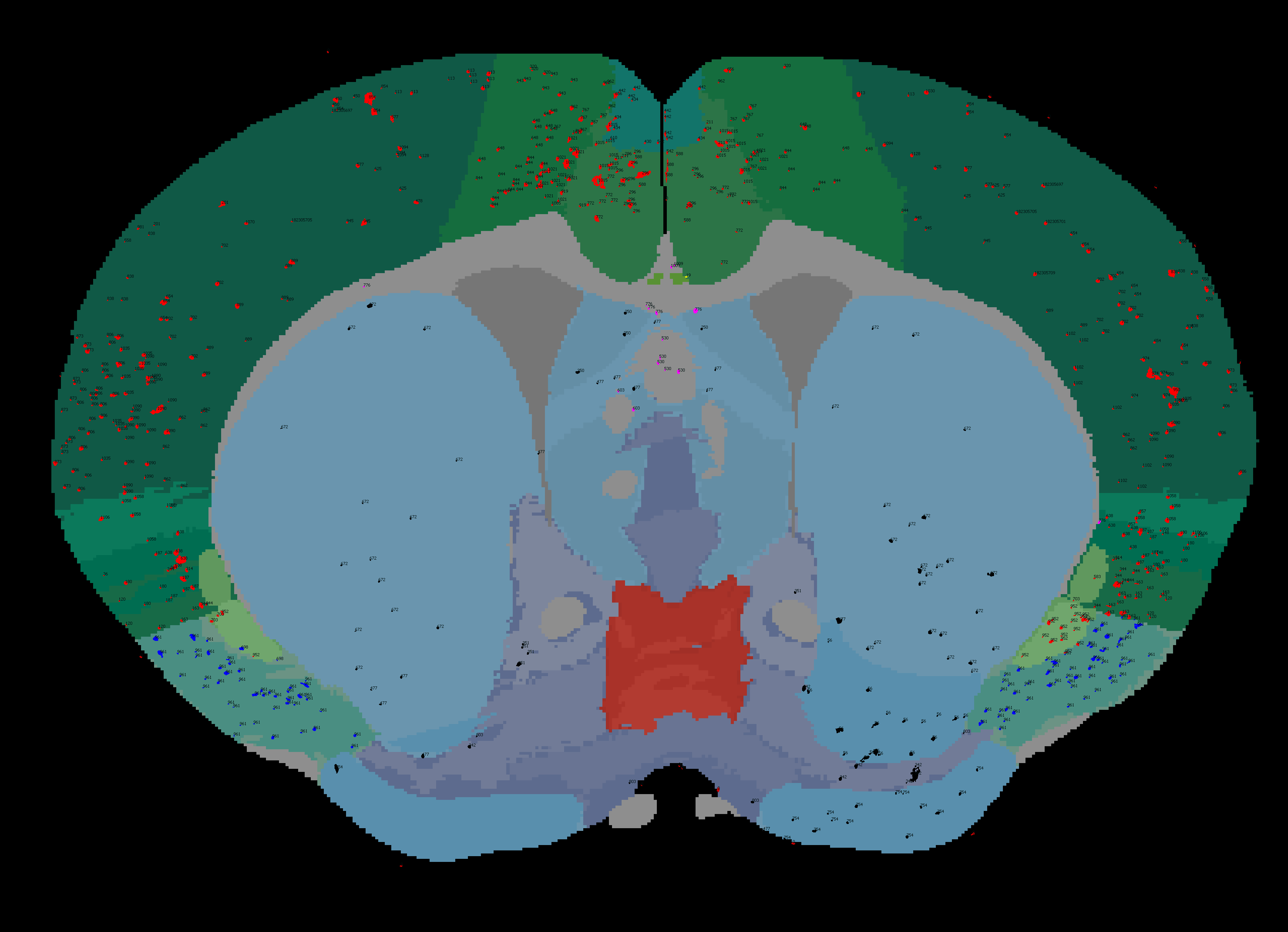

Supplement: Supplementary file 2 [file Data_Sheet_1.ZIP › Supplementary_material_Yates/pE-Abeta/tg2576_m287_pGlu_s088_Object Predictions.png]

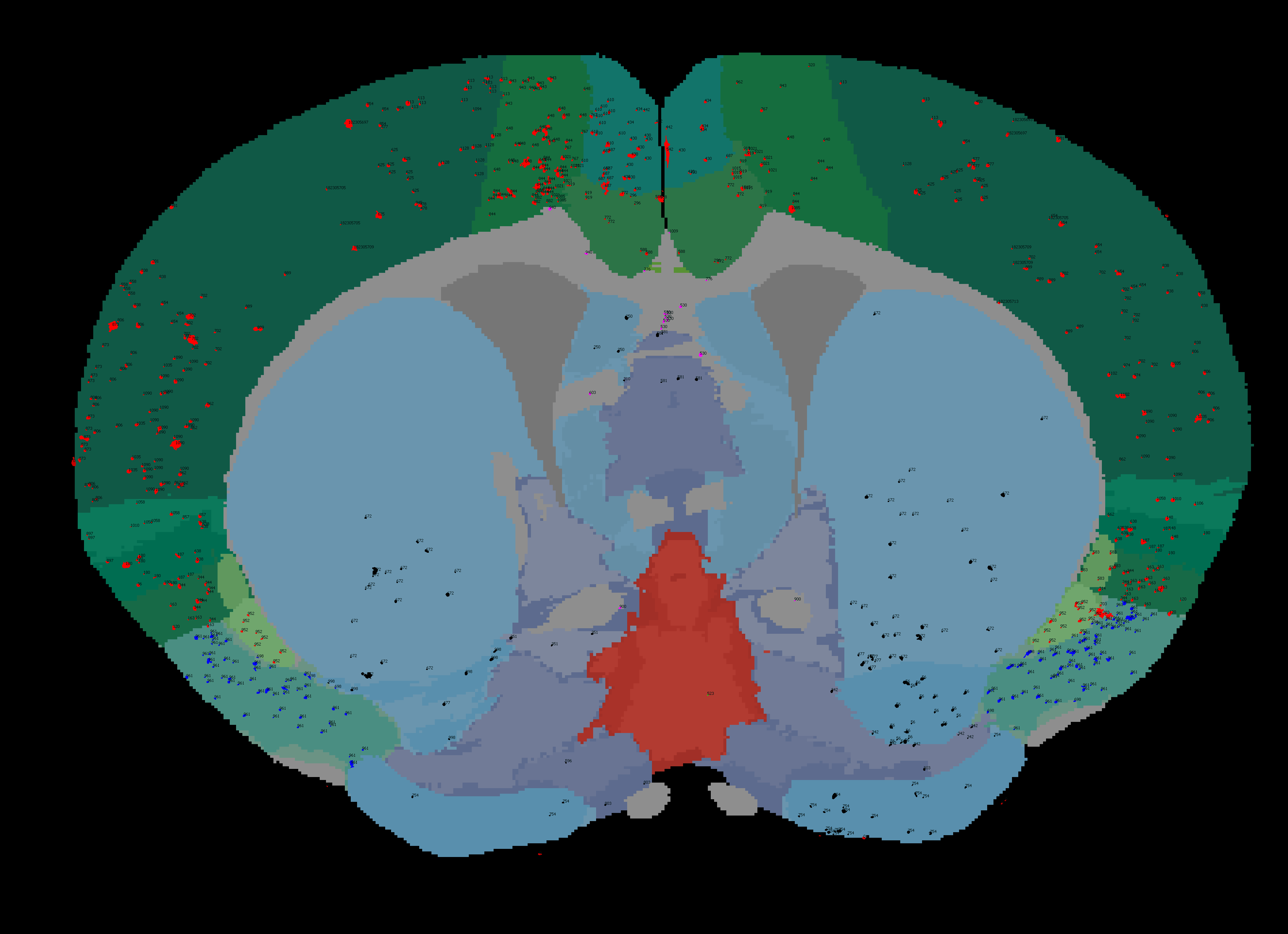

Supplement: Supplementary file 2 [file Data_Sheet_1.ZIP › Supplementary_material_Yates/pE-Abeta/tg2576_m287_pGlu_s092_Object Predictions.png]

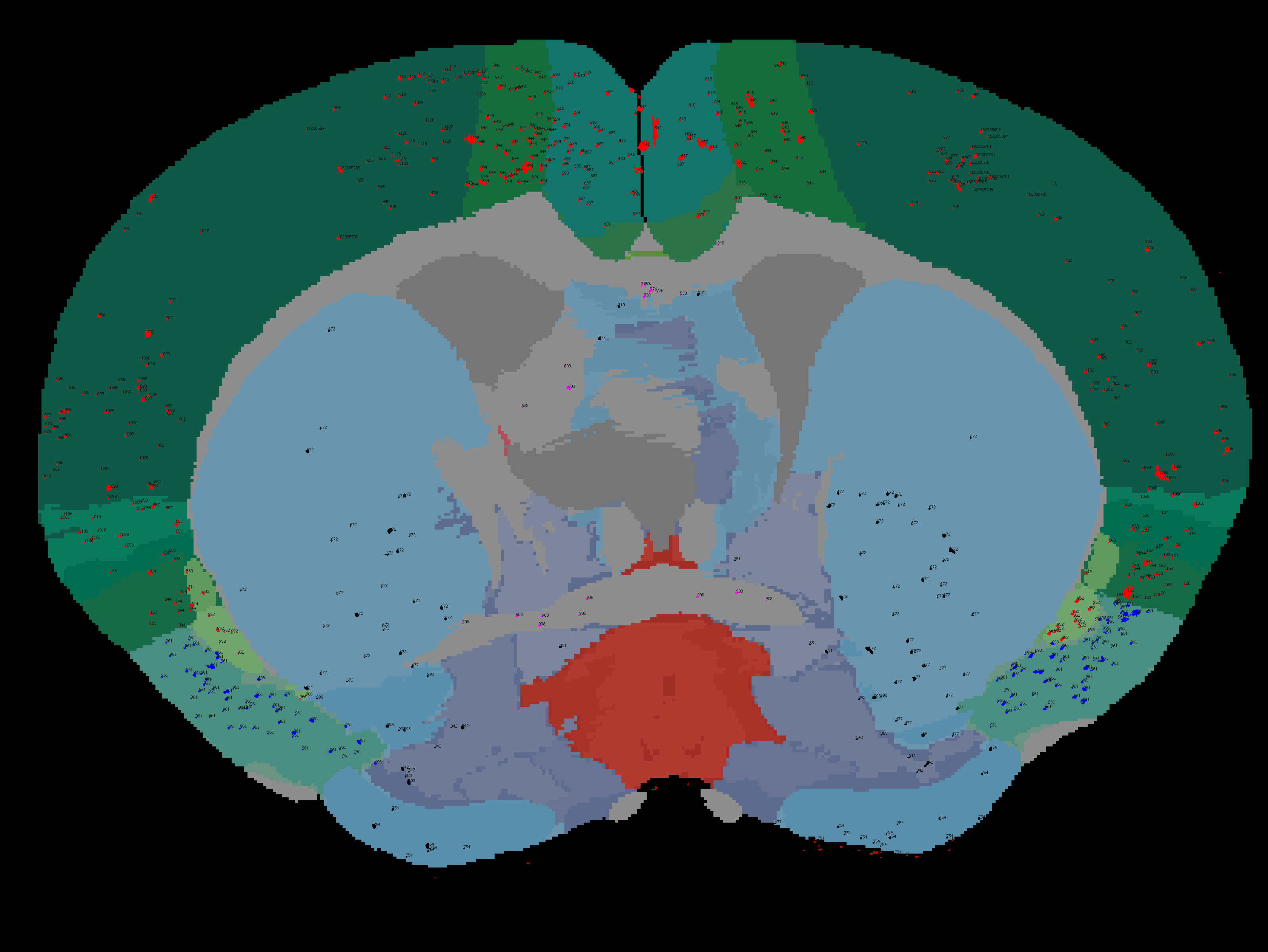

Supplement: Supplementary file 2 [file Data_Sheet_1.ZIP › Supplementary_material_Yates/pE-Abeta/tg2576_m287_pGlu_s096_Object Predictions.png]

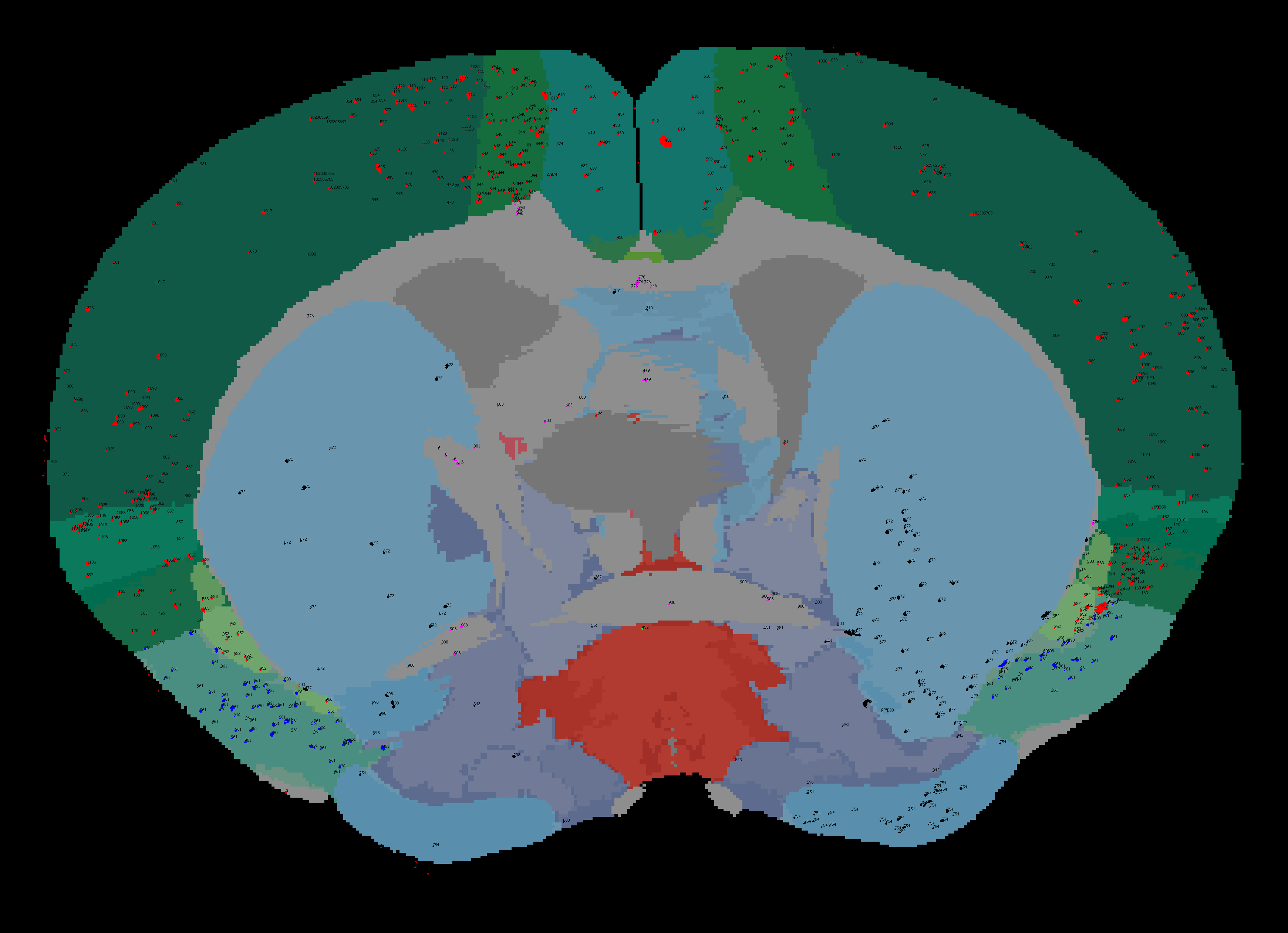

Supplement: Supplementary file 2 [file Data_Sheet_1.ZIP › Supplementary_material_Yates/pE-Abeta/tg2576_m287_pGlu_s100_Object Predictions.png]

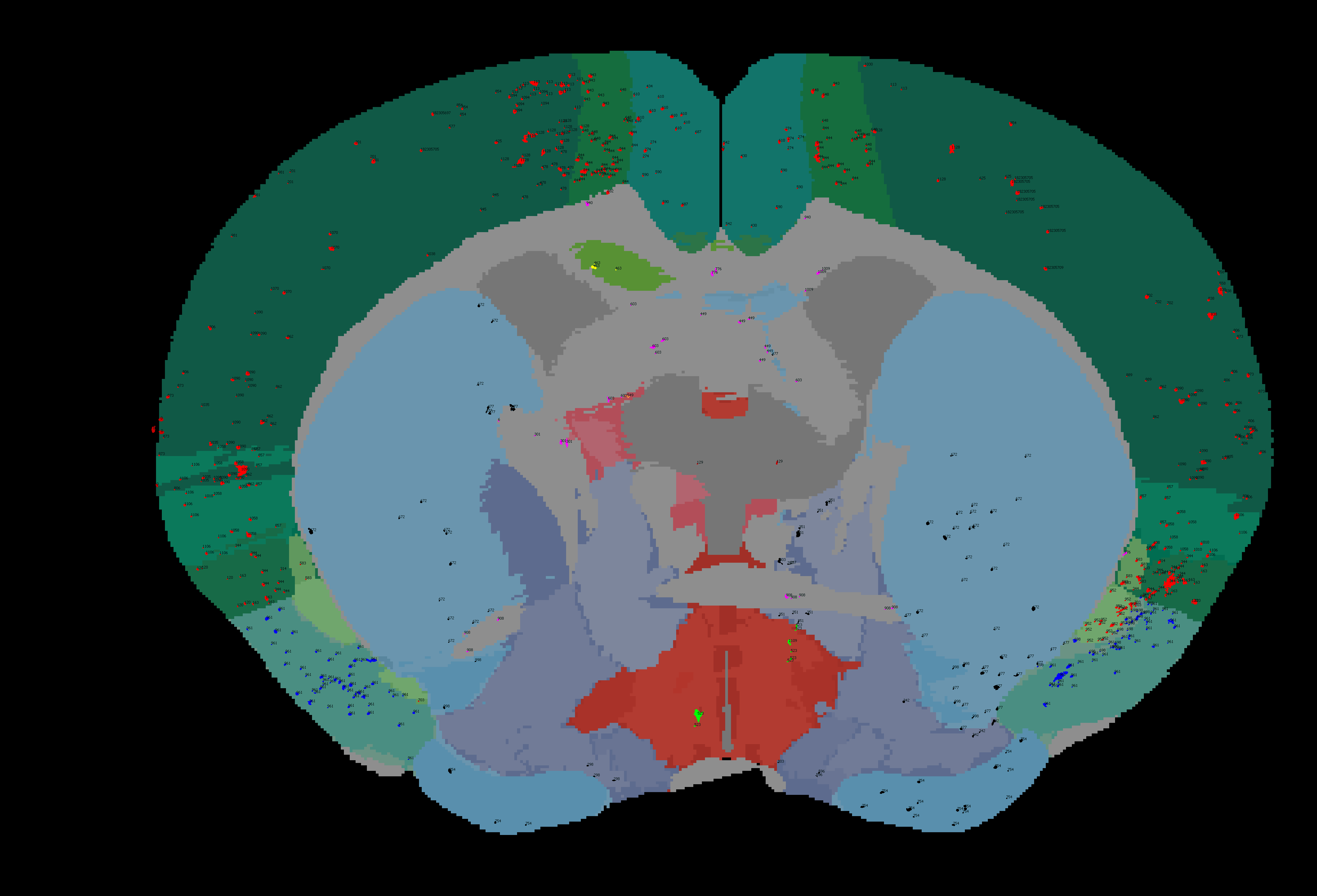

Supplement: Supplementary file 2 [file Data_Sheet_1.ZIP › Supplementary_material_Yates/pE-Abeta/tg2576_m287_pGlu_s104_Object Predictions.png]

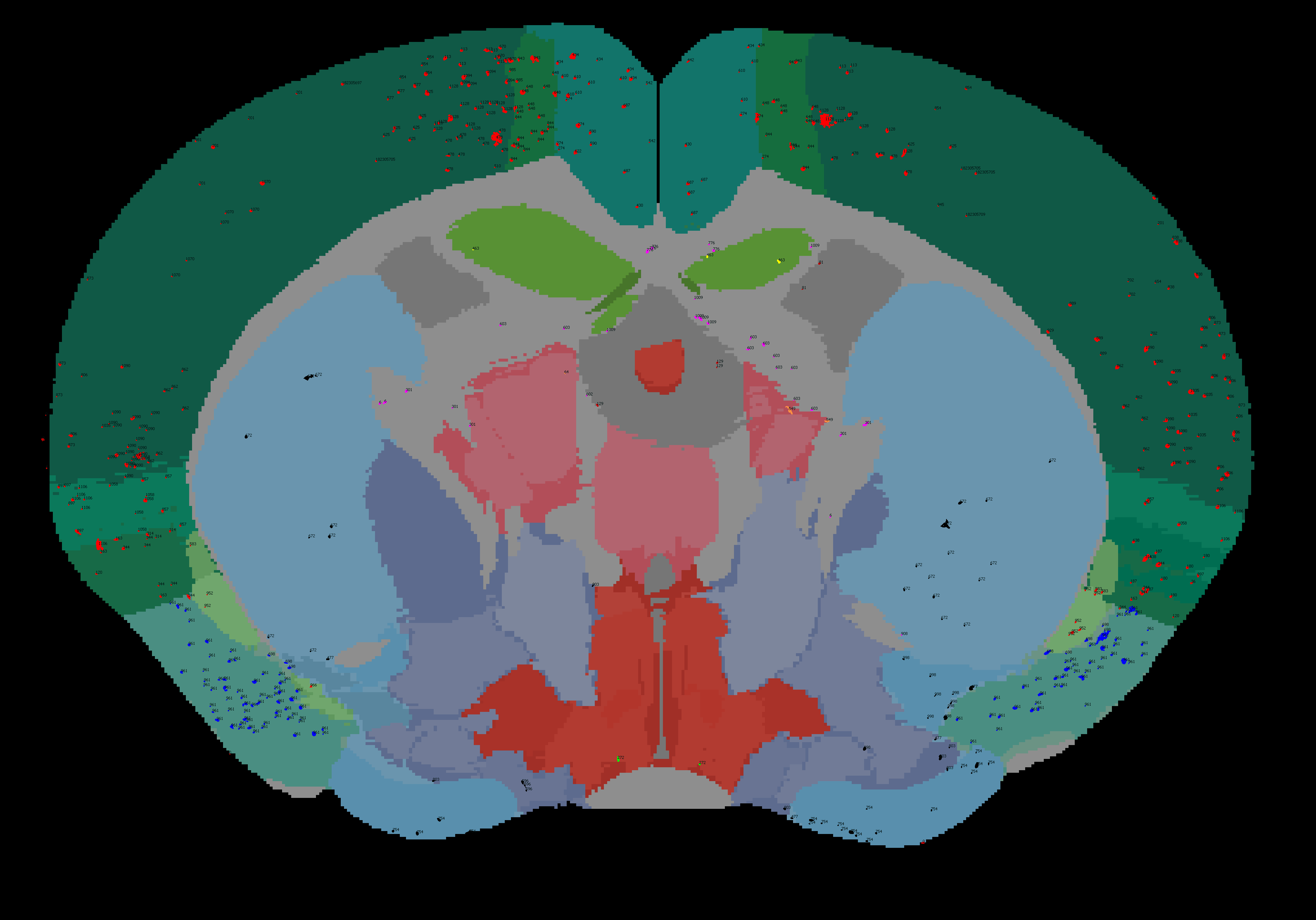

Supplement: Supplementary file 2 [file Data_Sheet_1.ZIP › Supplementary_material_Yates/pE-Abeta/tg2576_m287_pGlu_s108_Object Predictions.png]

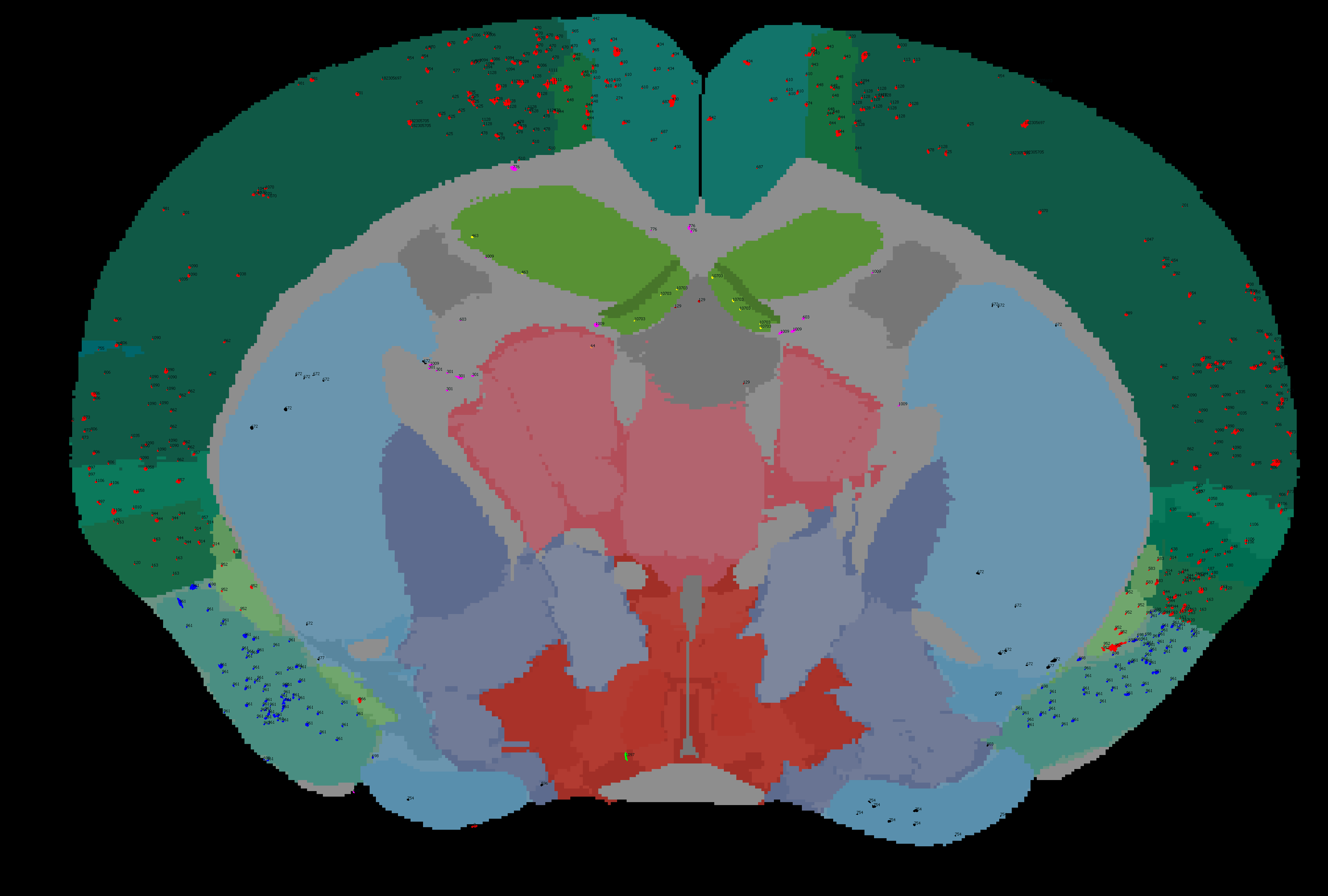

Supplement: Supplementary file 2 [file Data_Sheet_1.ZIP › Supplementary_material_Yates/pE-Abeta/tg2576_m287_pGlu_s112_Object Predictions.png]

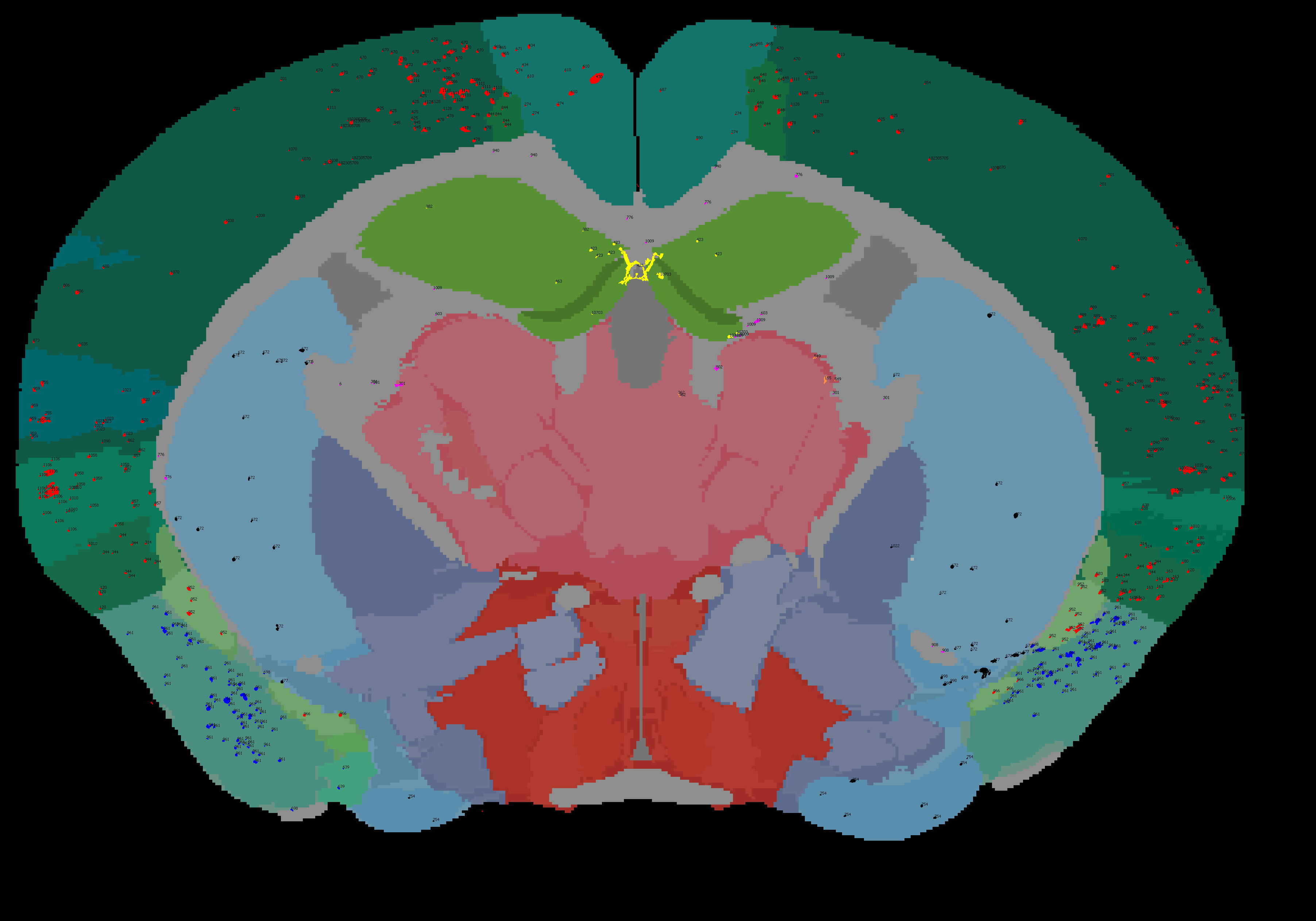

Supplement: Supplementary file 2 [file Data_Sheet_1.ZIP › Supplementary_material_Yates/pE-Abeta/tg2576_m287_pGlu_s116_Object Predictions.png]

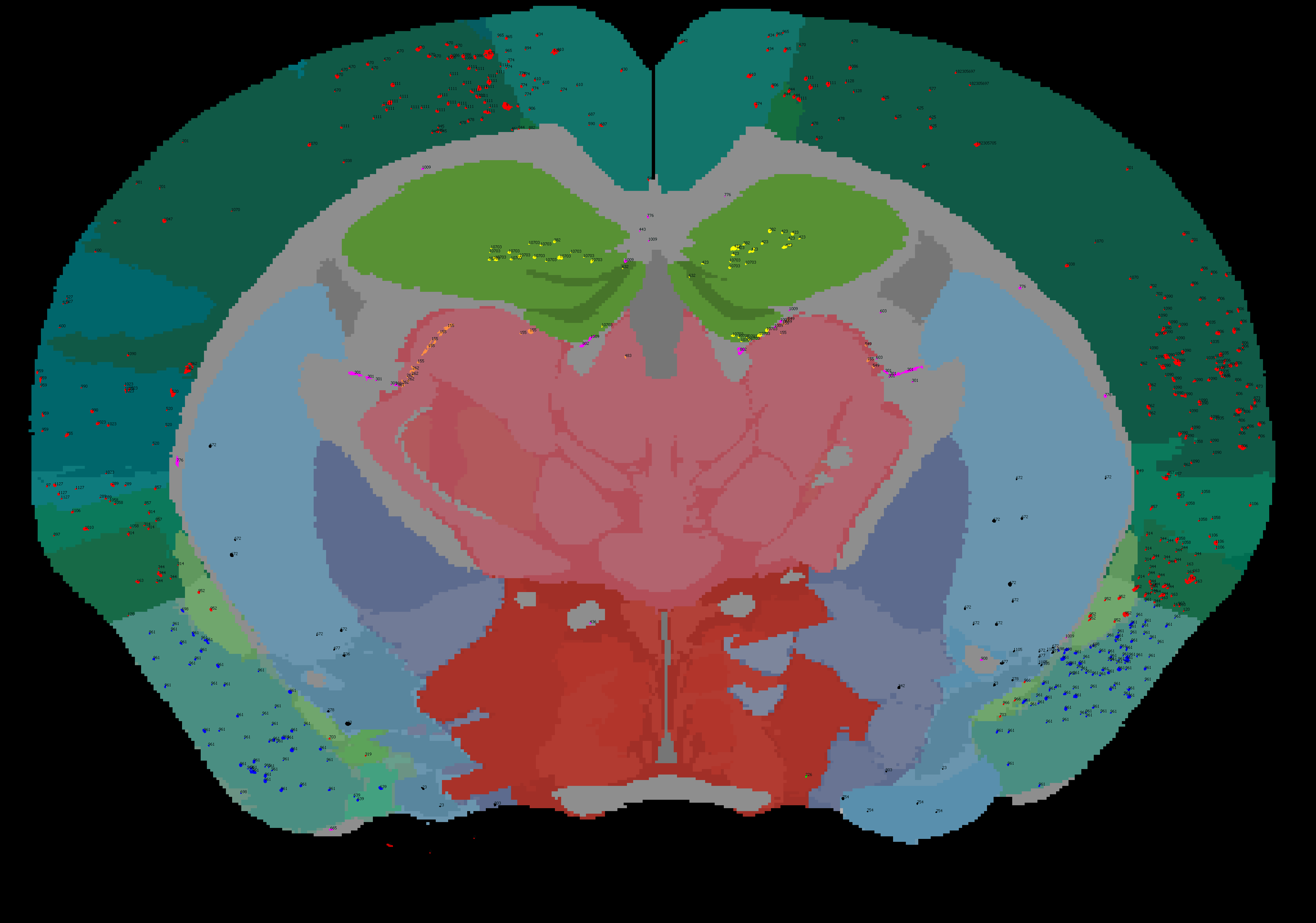

Supplement: Supplementary file 2 [file Data_Sheet_1.ZIP › Supplementary_material_Yates/pE-Abeta/tg2576_m287_pGlu_s120_Object Predictions.png]

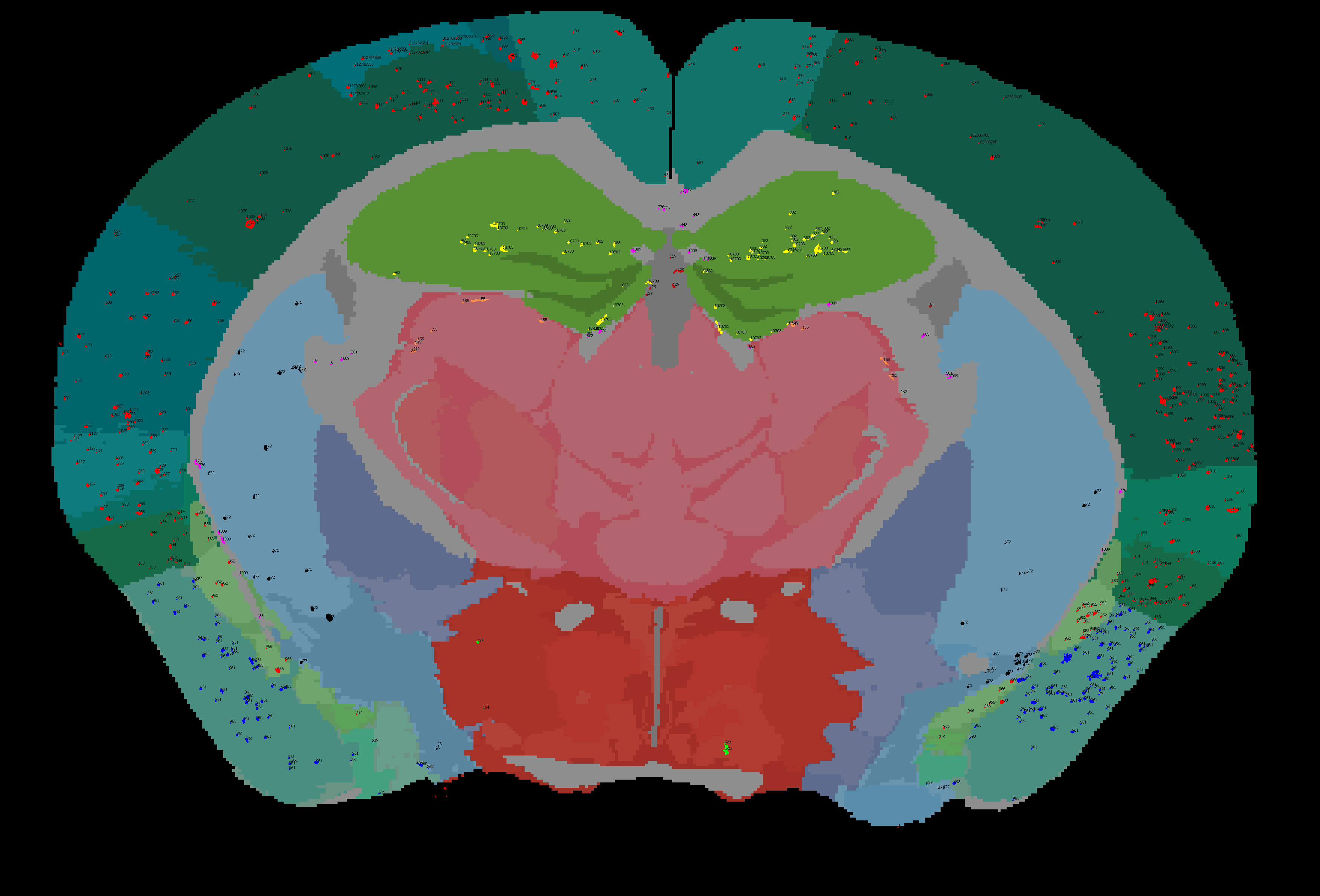

Supplement: Supplementary file 2 [file Data_Sheet_1.ZIP › Supplementary_material_Yates/pE-Abeta/tg2576_m287_pGlu_s124_Object Predictions.png]

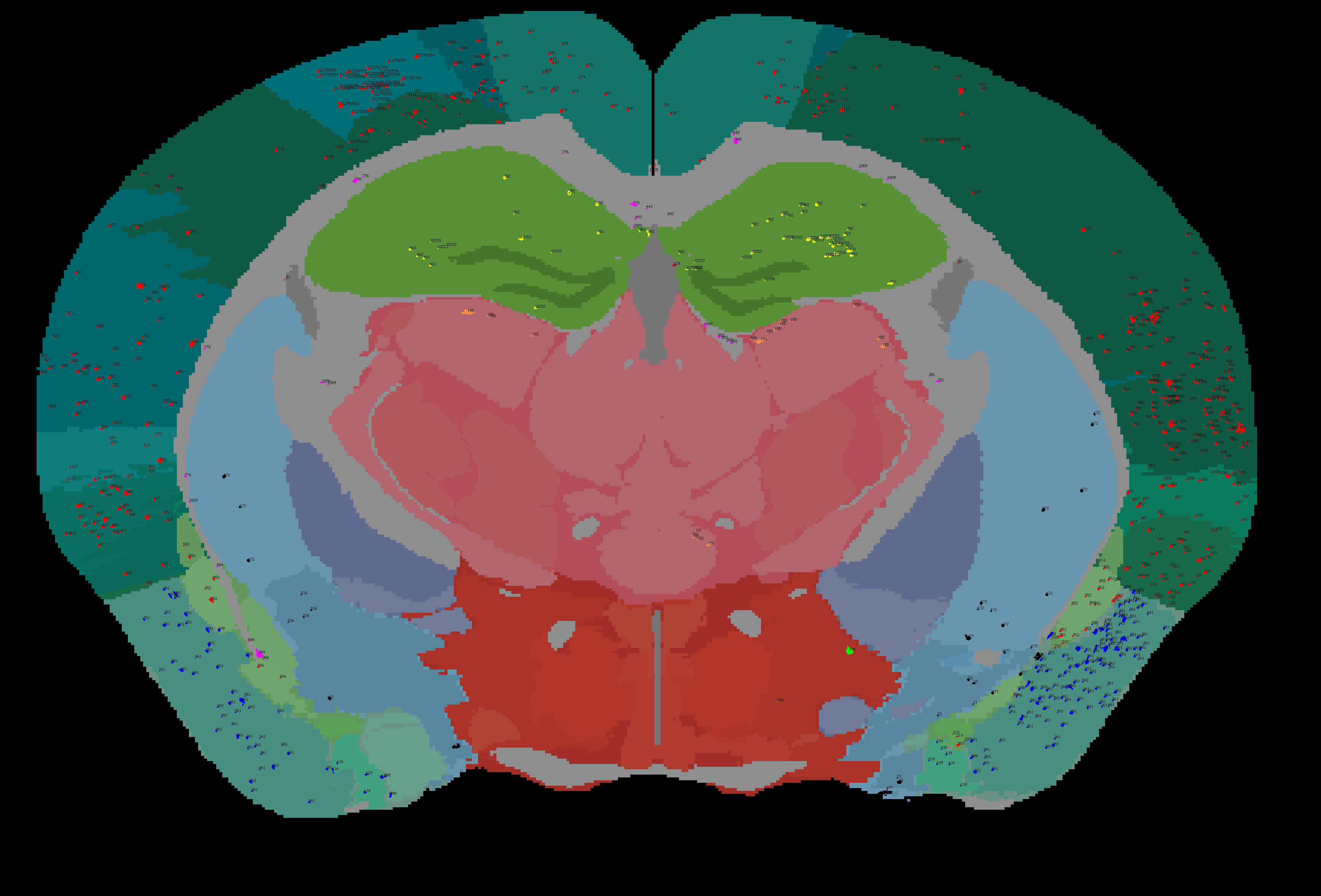

Supplement: Supplementary file 2 [file Data_Sheet_1.ZIP › Supplementary_material_Yates/pE-Abeta/tg2576_m287_pGlu_s128_Object Predictions.png]

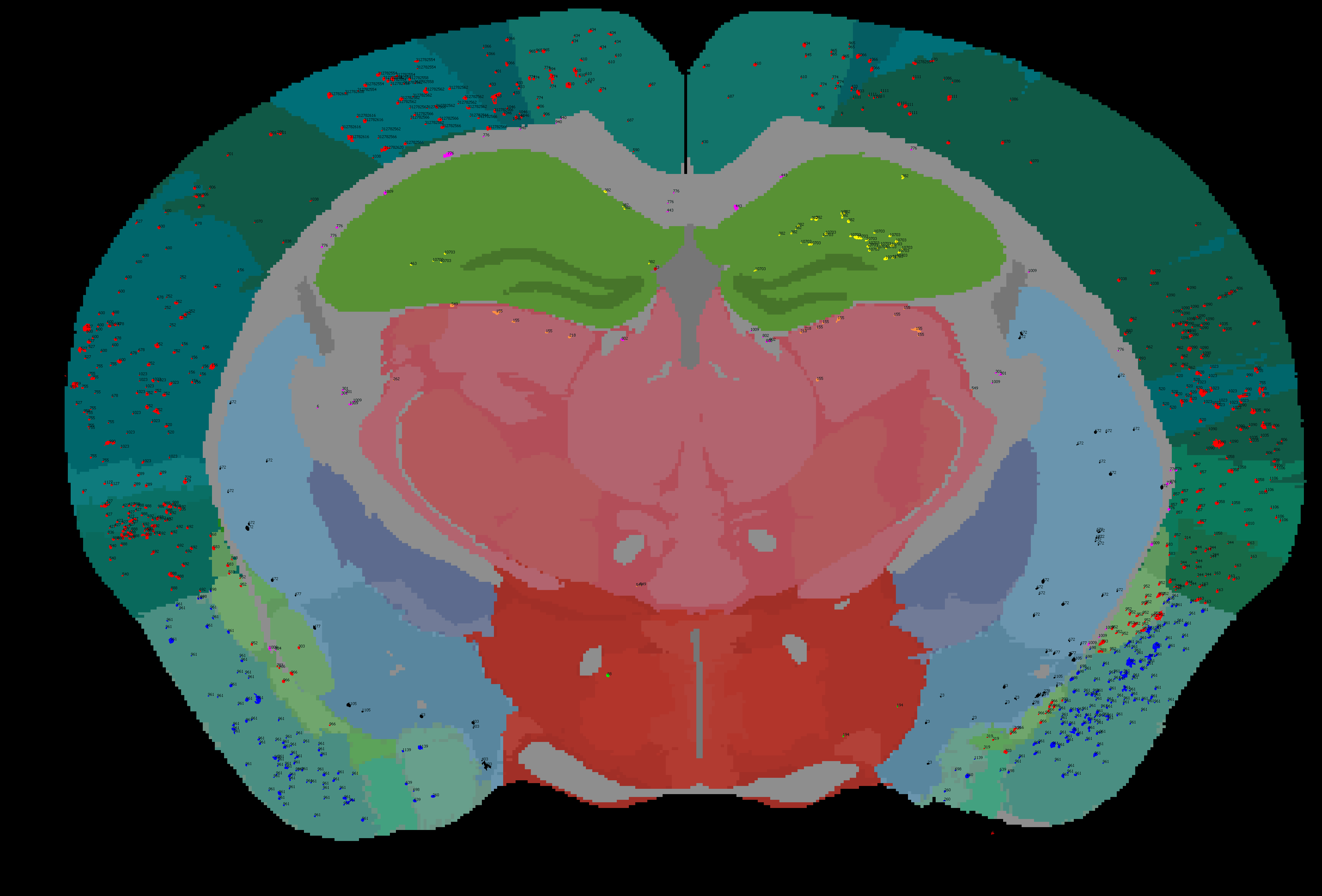

Supplement: Supplementary file 2 [file Data_Sheet_1.ZIP › Supplementary_material_Yates/pE-Abeta/tg2576_m287_pGlu_s132_Object Predictions.png]

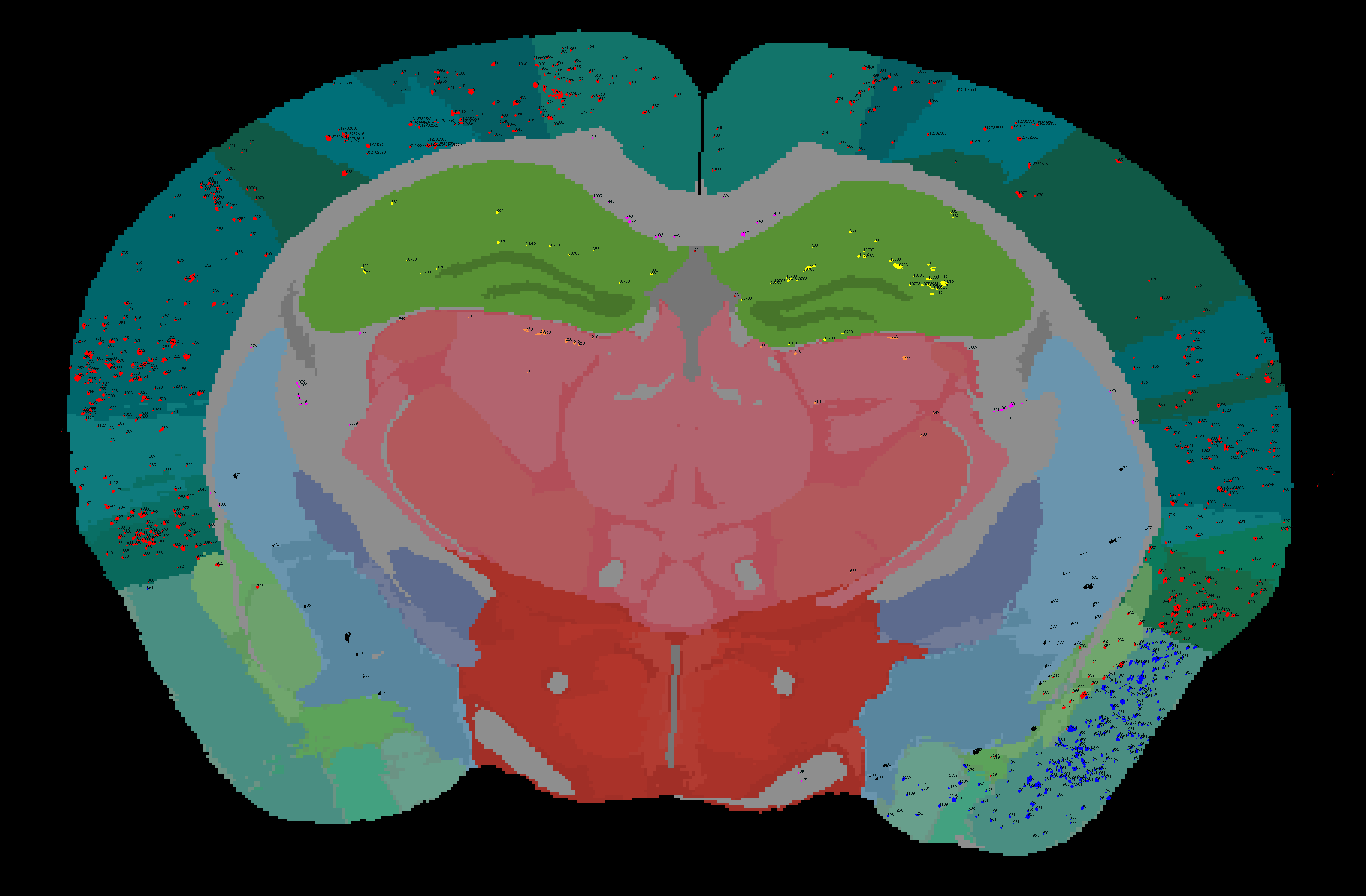

Supplement: Supplementary file 2 [file Data_Sheet_1.ZIP › Supplementary_material_Yates/pE-Abeta/tg2576_m287_pGlu_s136_Object Predictions.png]

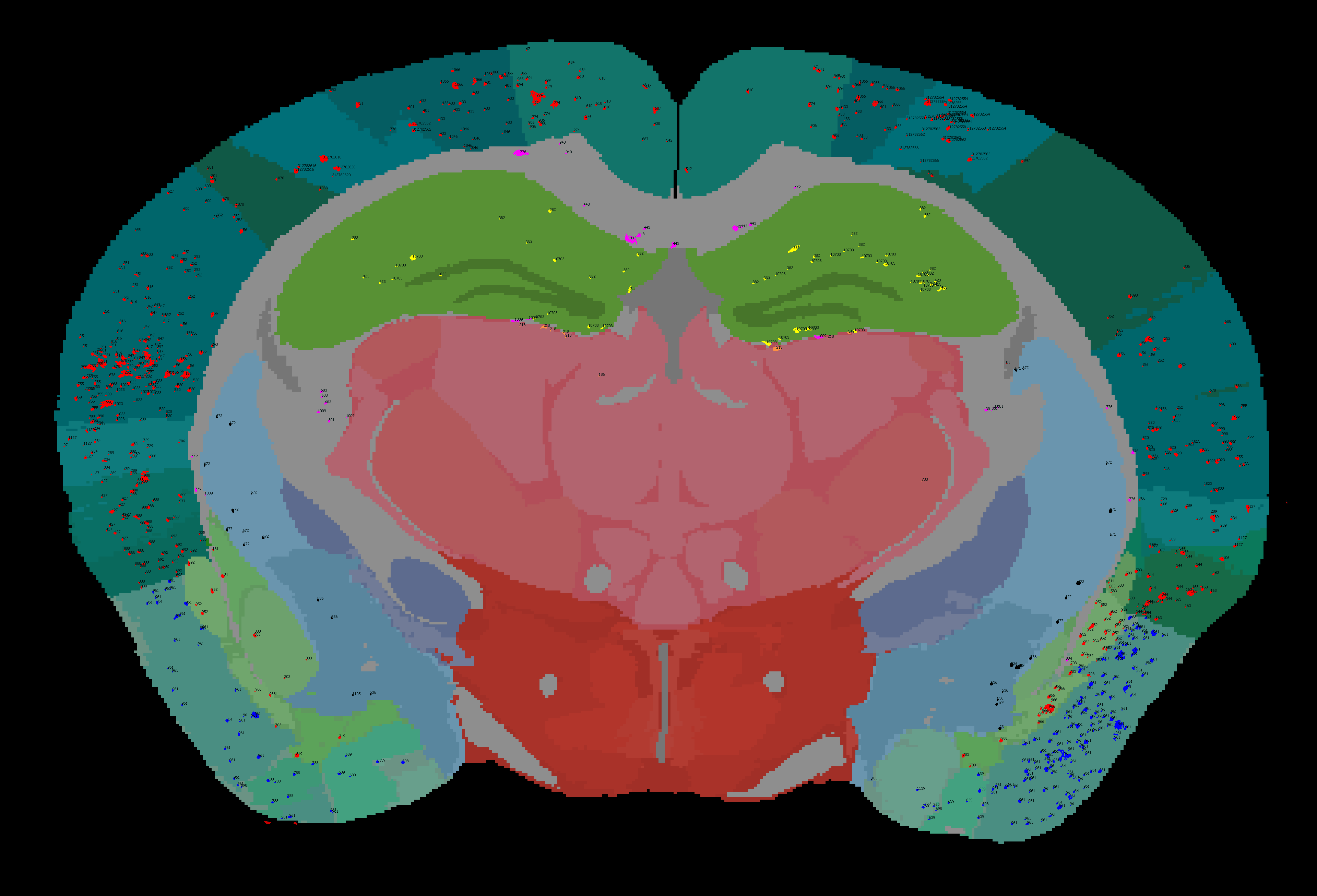

Supplement: Supplementary file 2 [file Data_Sheet_1.ZIP › Supplementary_material_Yates/pE-Abeta/tg2576_m287_pGlu_s140_Object Predictions.png]

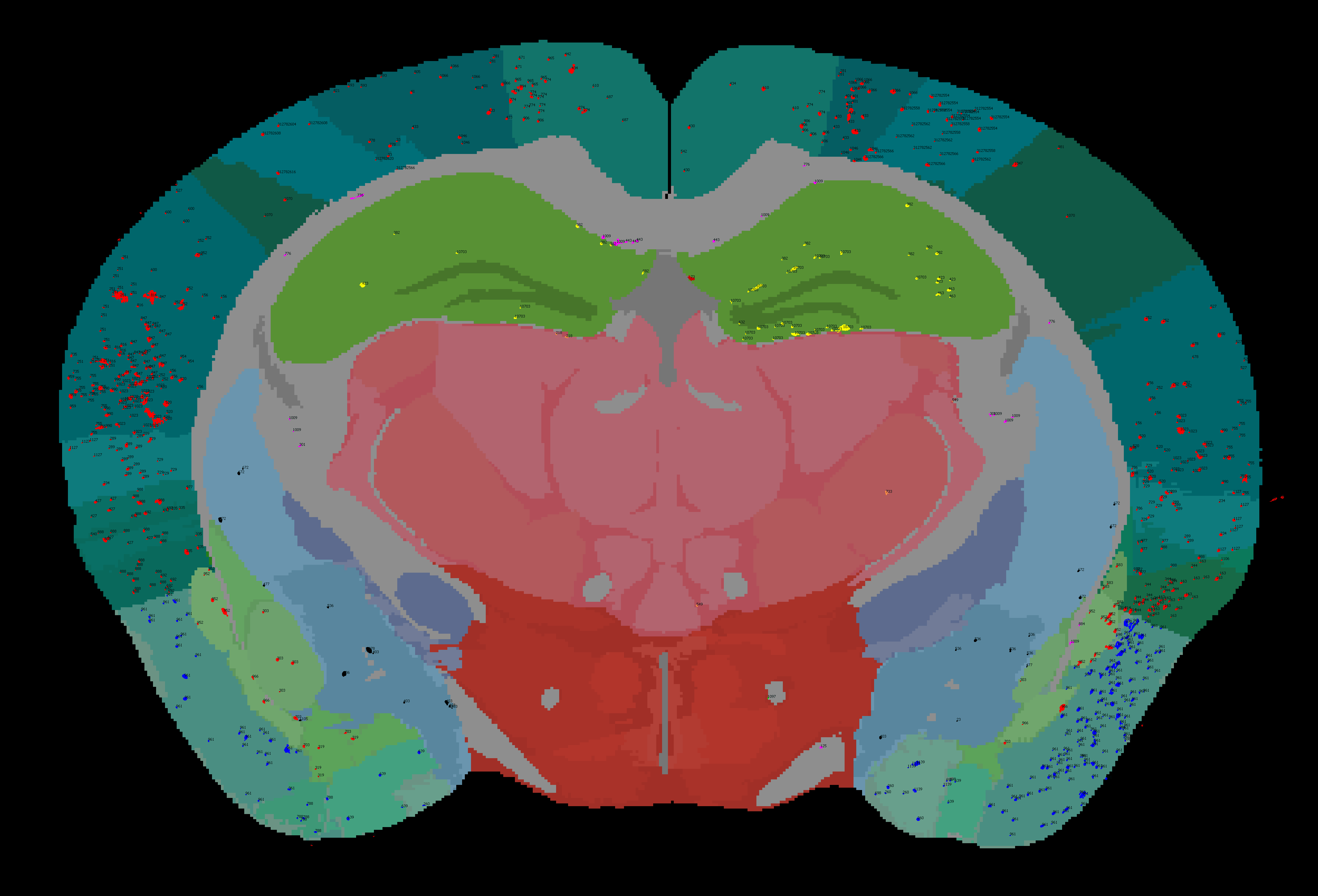

Supplement: Supplementary file 2 [file Data_Sheet_1.ZIP › Supplementary_material_Yates/pE-Abeta/tg2576_m287_pGlu_s144_Object Predictions.png]

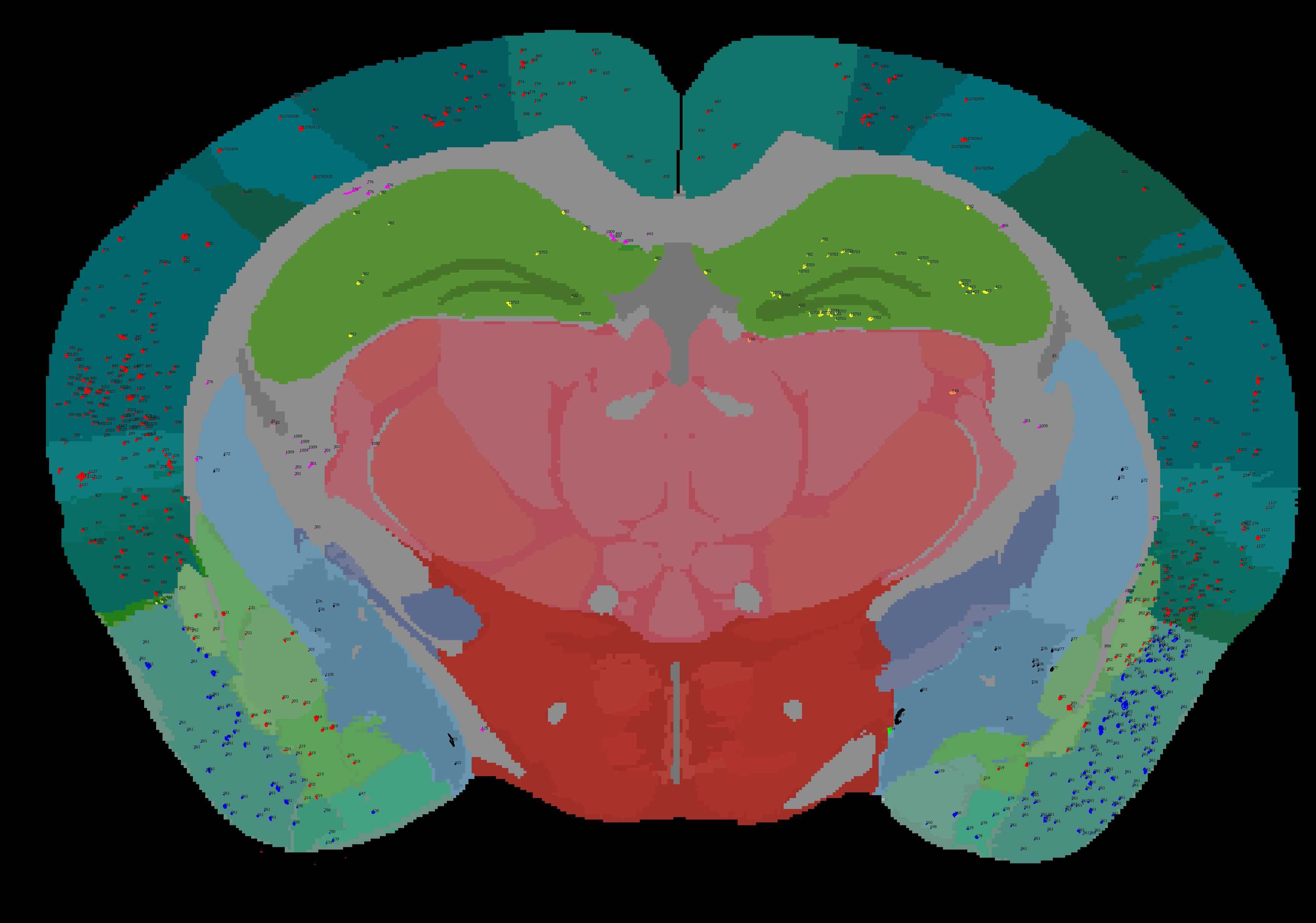

Supplement: Supplementary file 2 [file Data_Sheet_1.ZIP › Supplementary_material_Yates/pE-Abeta/tg2576_m287_pGlu_s148_Object Predictions.png]

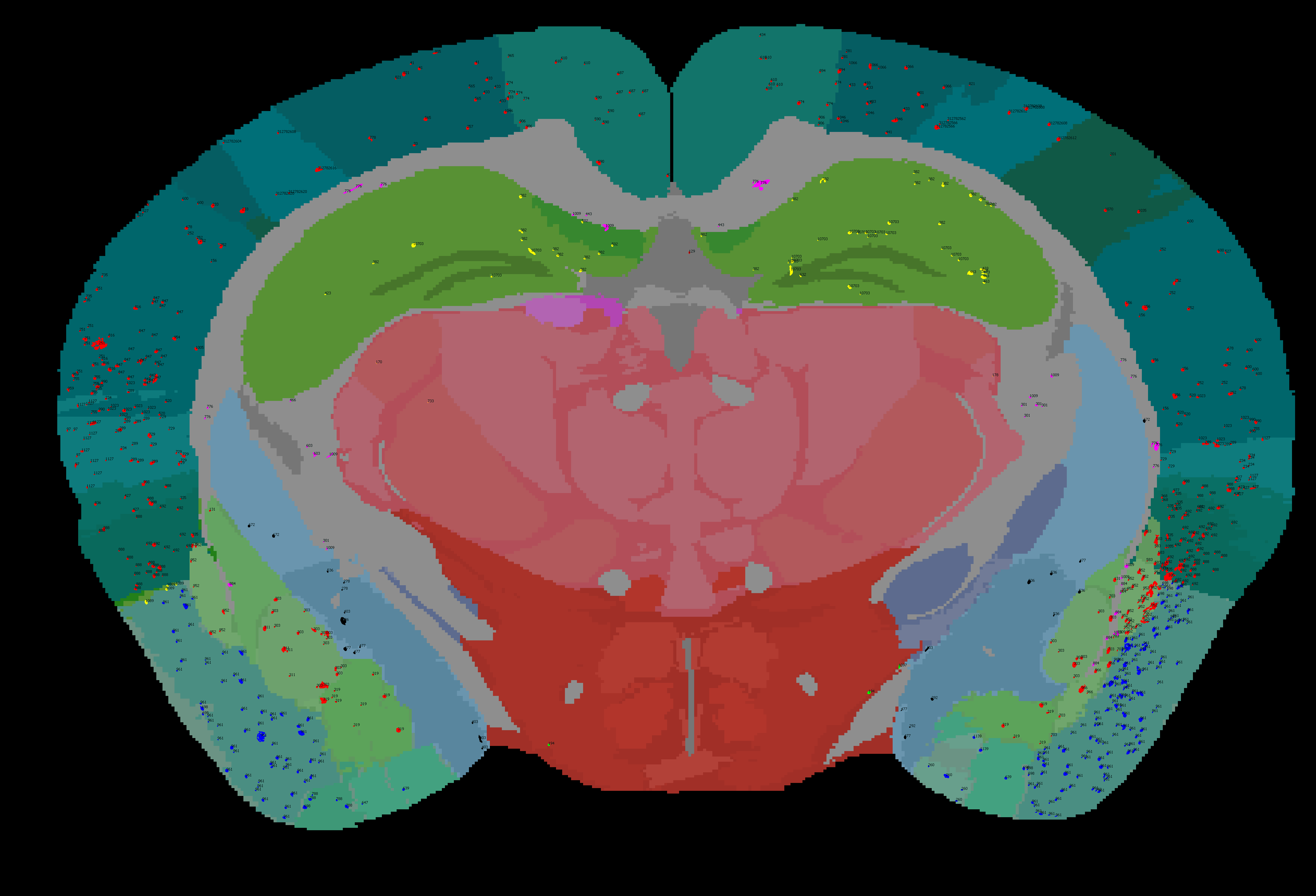

Supplement: Supplementary file 2 [file Data_Sheet_1.ZIP › Supplementary_material_Yates/pE-Abeta/tg2576_m287_pGlu_s152_Object Predictions.png]

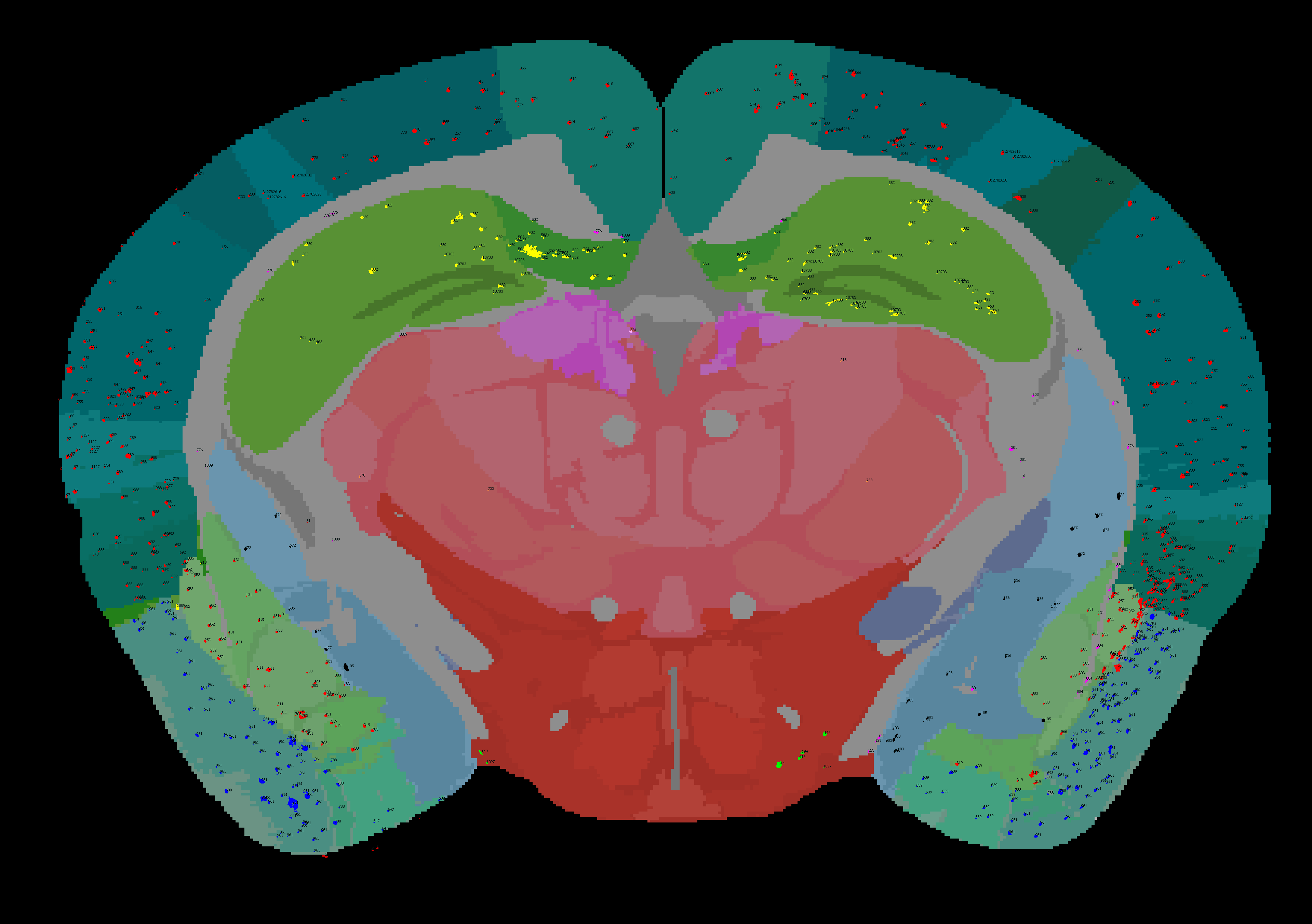

Supplement: Supplementary file 2 [file Data_Sheet_1.ZIP › Supplementary_material_Yates/pE-Abeta/tg2576_m287_pGlu_s156_Object Predictions.png]

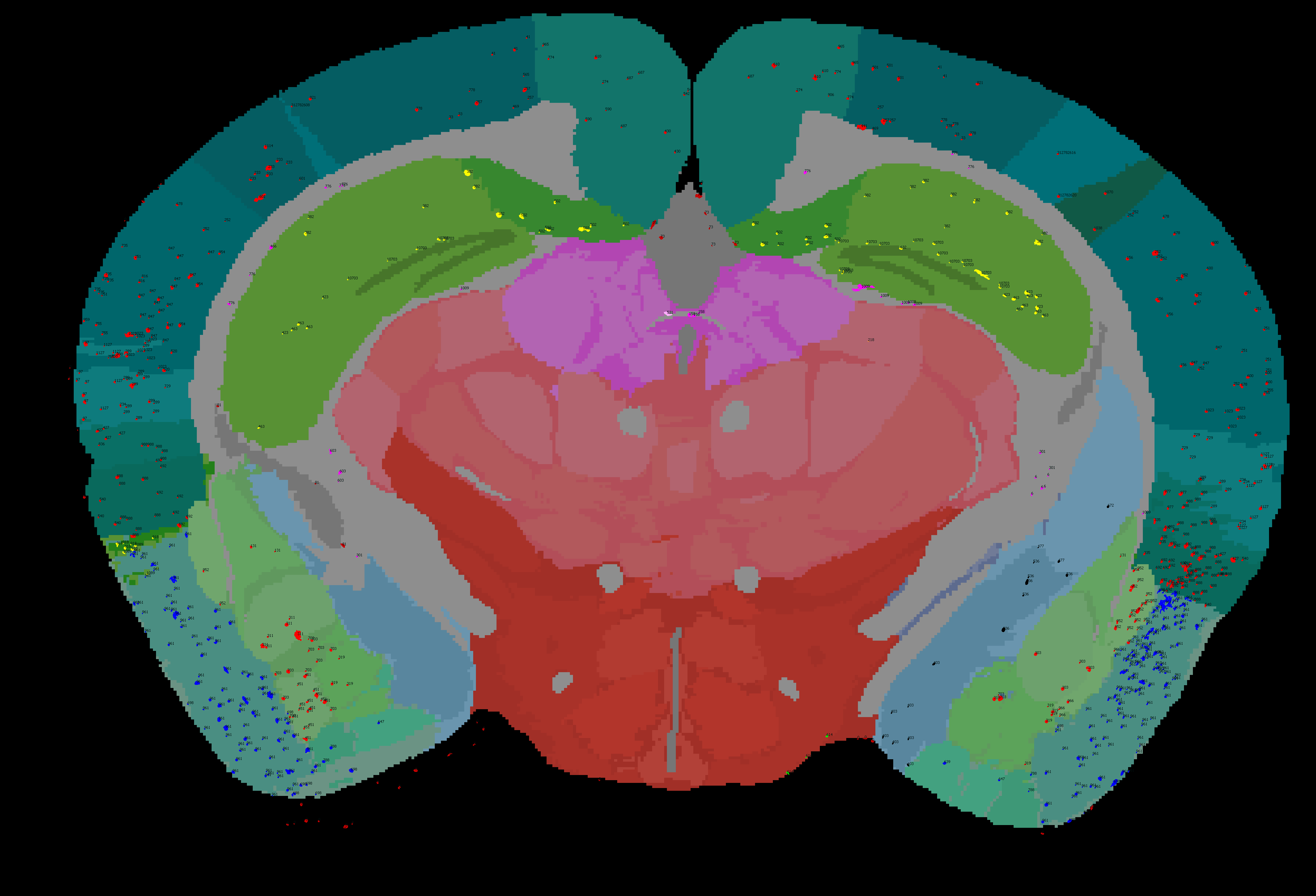

Supplement: Supplementary file 2 [file Data_Sheet_1.ZIP › Supplementary_material_Yates/pE-Abeta/tg2576_m287_pGlu_s160_Object Predictions.png]

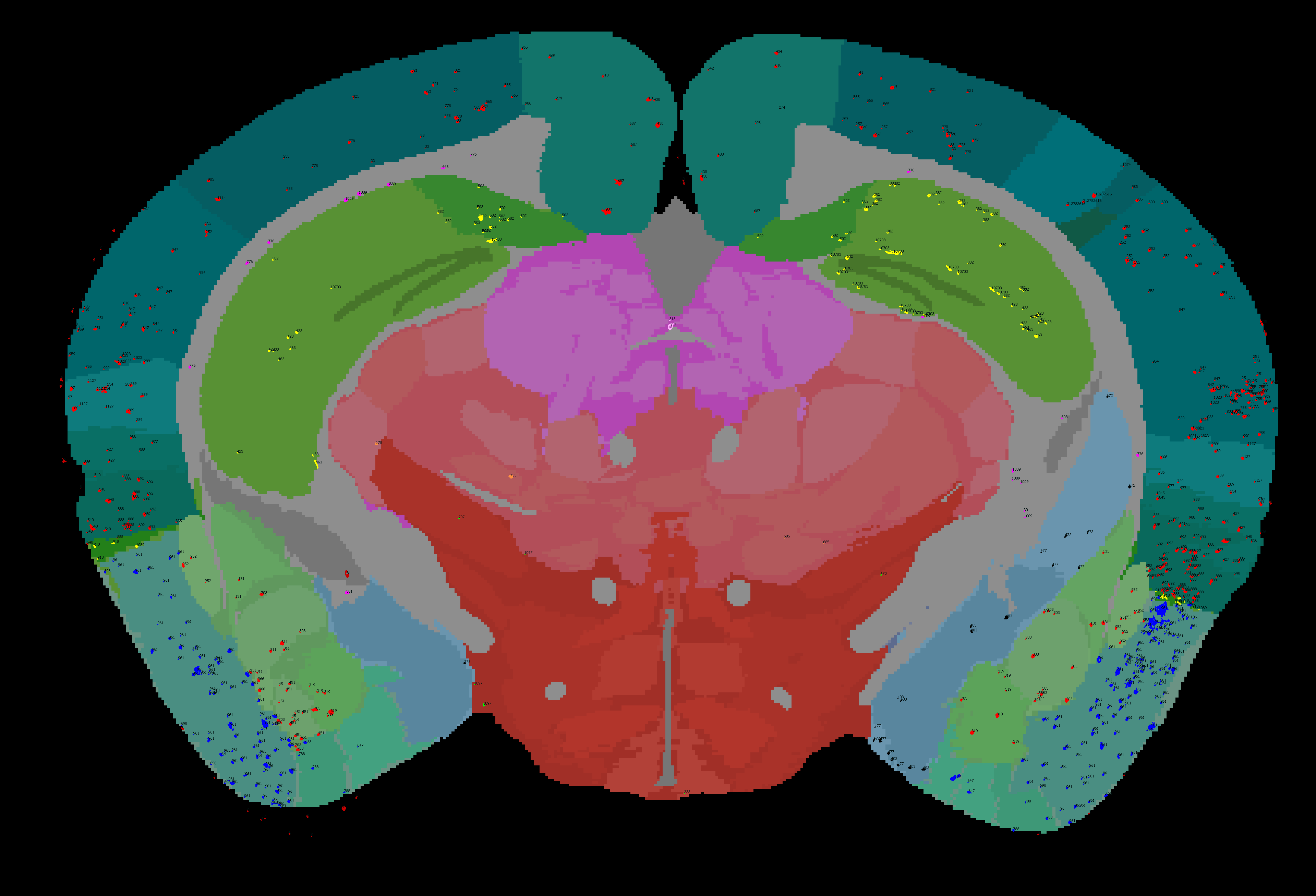

Supplement: Supplementary file 2 [file Data_Sheet_1.ZIP › Supplementary_material_Yates/pE-Abeta/tg2576_m287_pGlu_s164_Object Predictions.png]

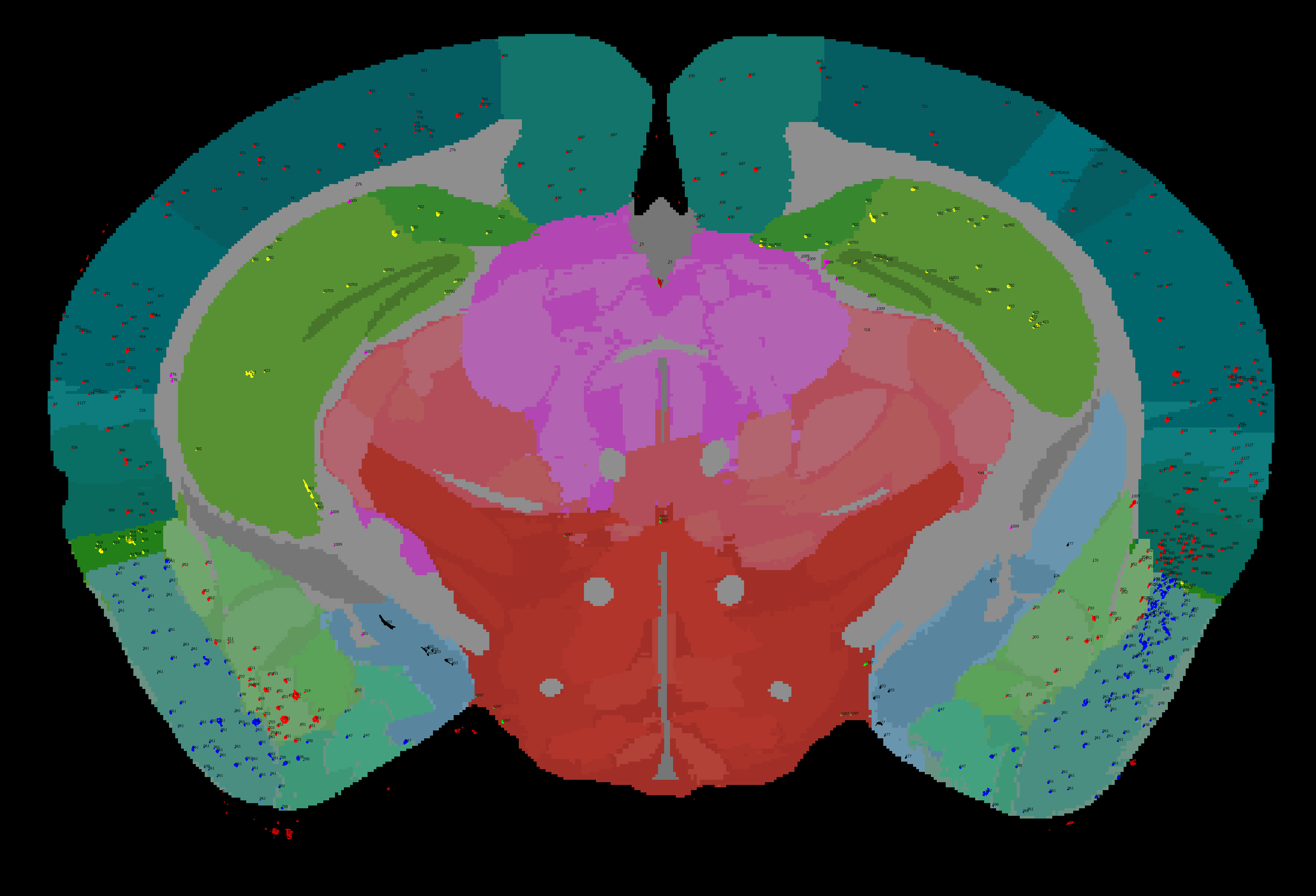

Supplement: Supplementary file 2 [file Data_Sheet_1.ZIP › Supplementary_material_Yates/pE-Abeta/tg2576_m287_pGlu_s168_Object Predictions.png]

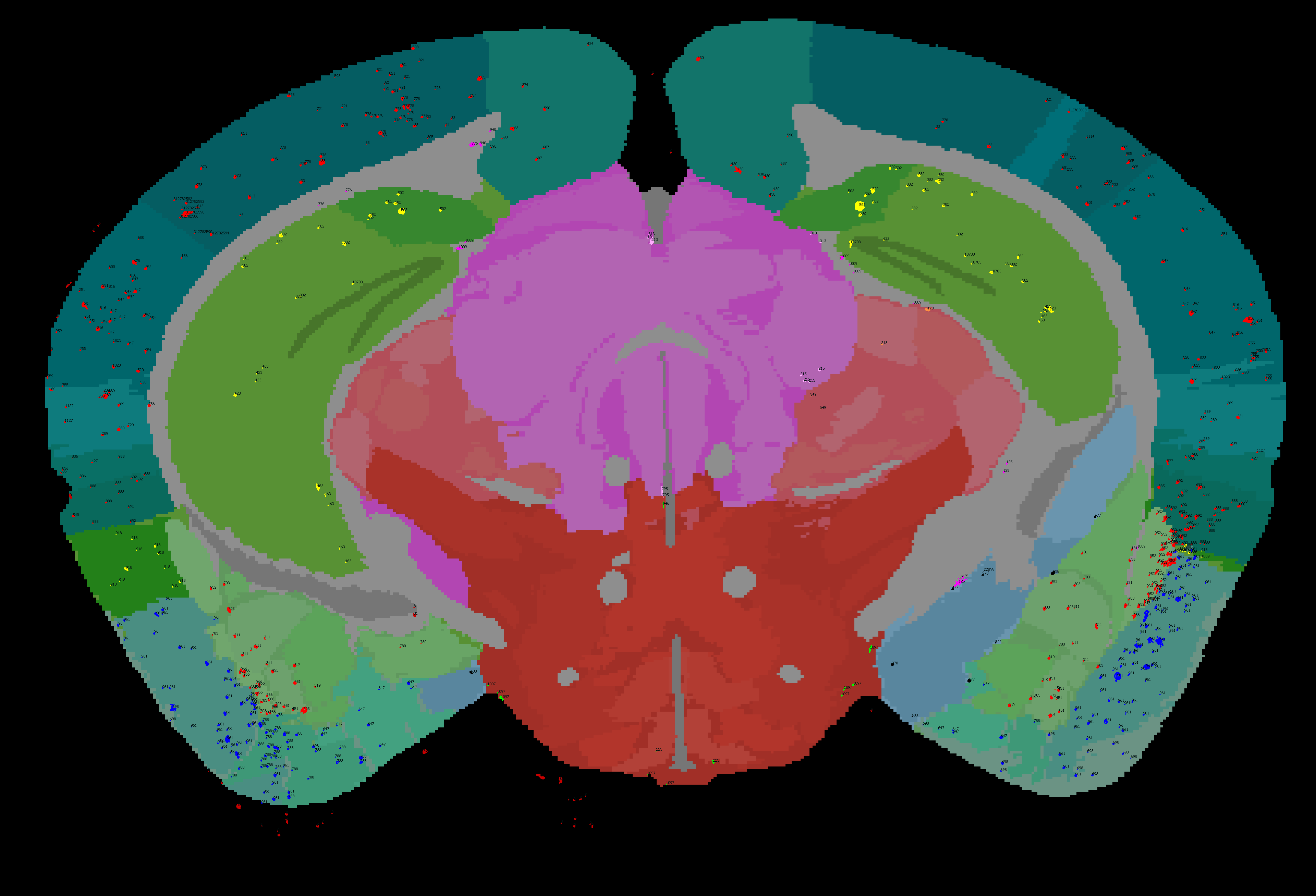

Supplement: Supplementary file 2 [file Data_Sheet_1.ZIP › Supplementary_material_Yates/pE-Abeta/tg2576_m287_pGlu_s172_Object Predictions.png]

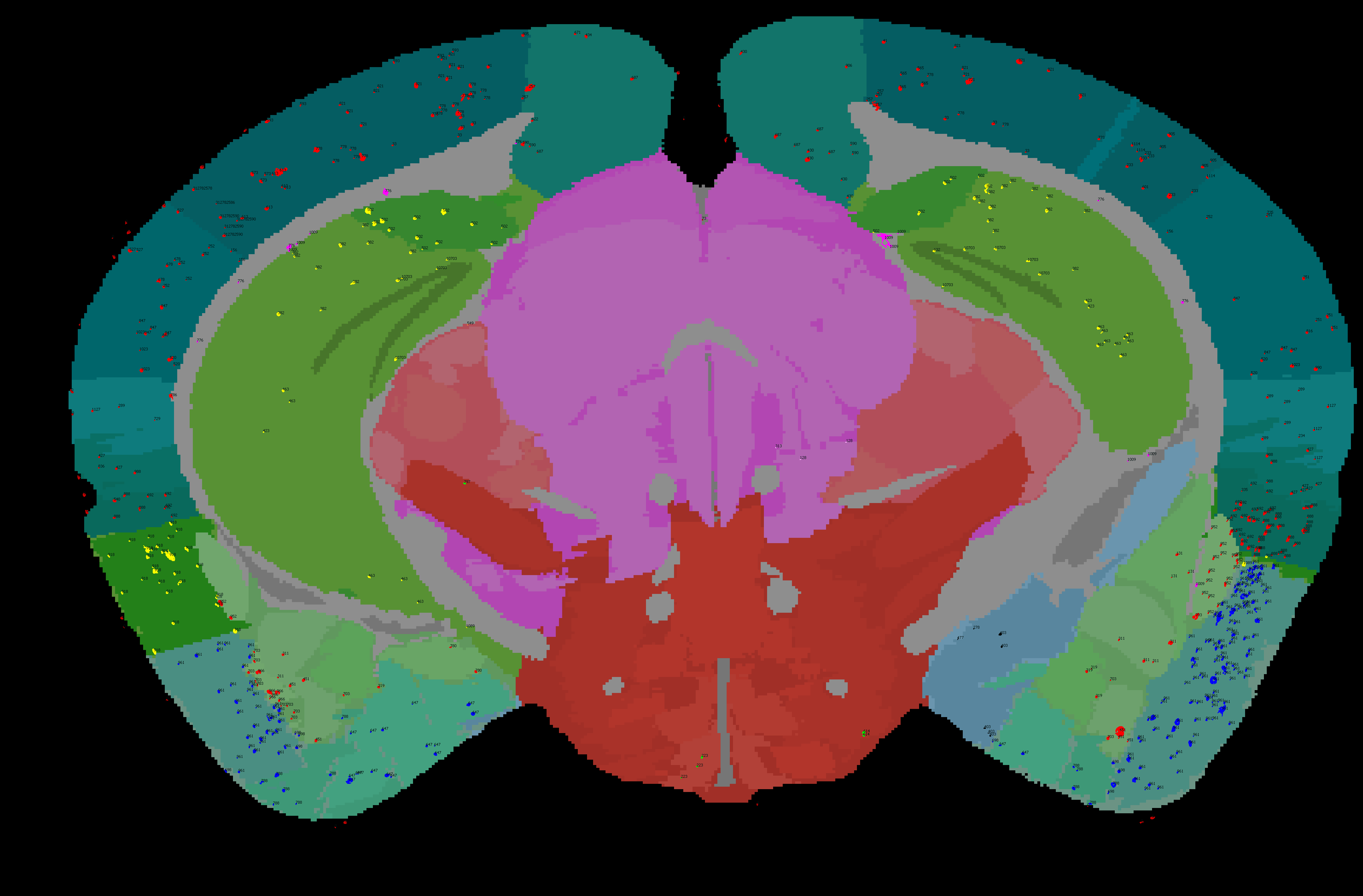

Supplement: Supplementary file 2 [file Data_Sheet_1.ZIP › Supplementary_material_Yates/pE-Abeta/tg2576_m287_pGlu_s176_Object Predictions.png]

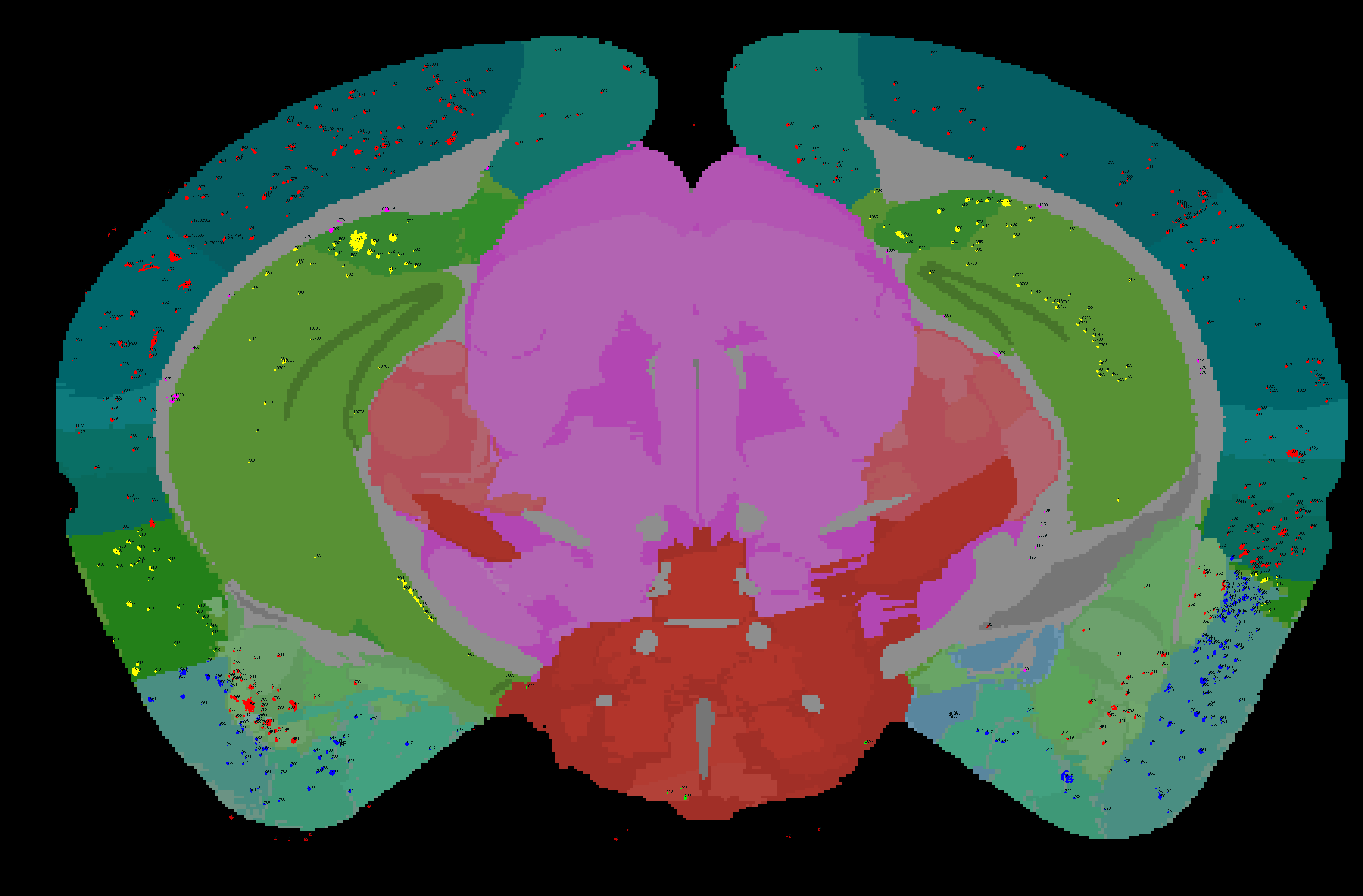

Supplement: Supplementary file 2 [file Data_Sheet_1.ZIP › Supplementary_material_Yates/pE-Abeta/tg2576_m287_pGlu_s180_Object Predictions.png]

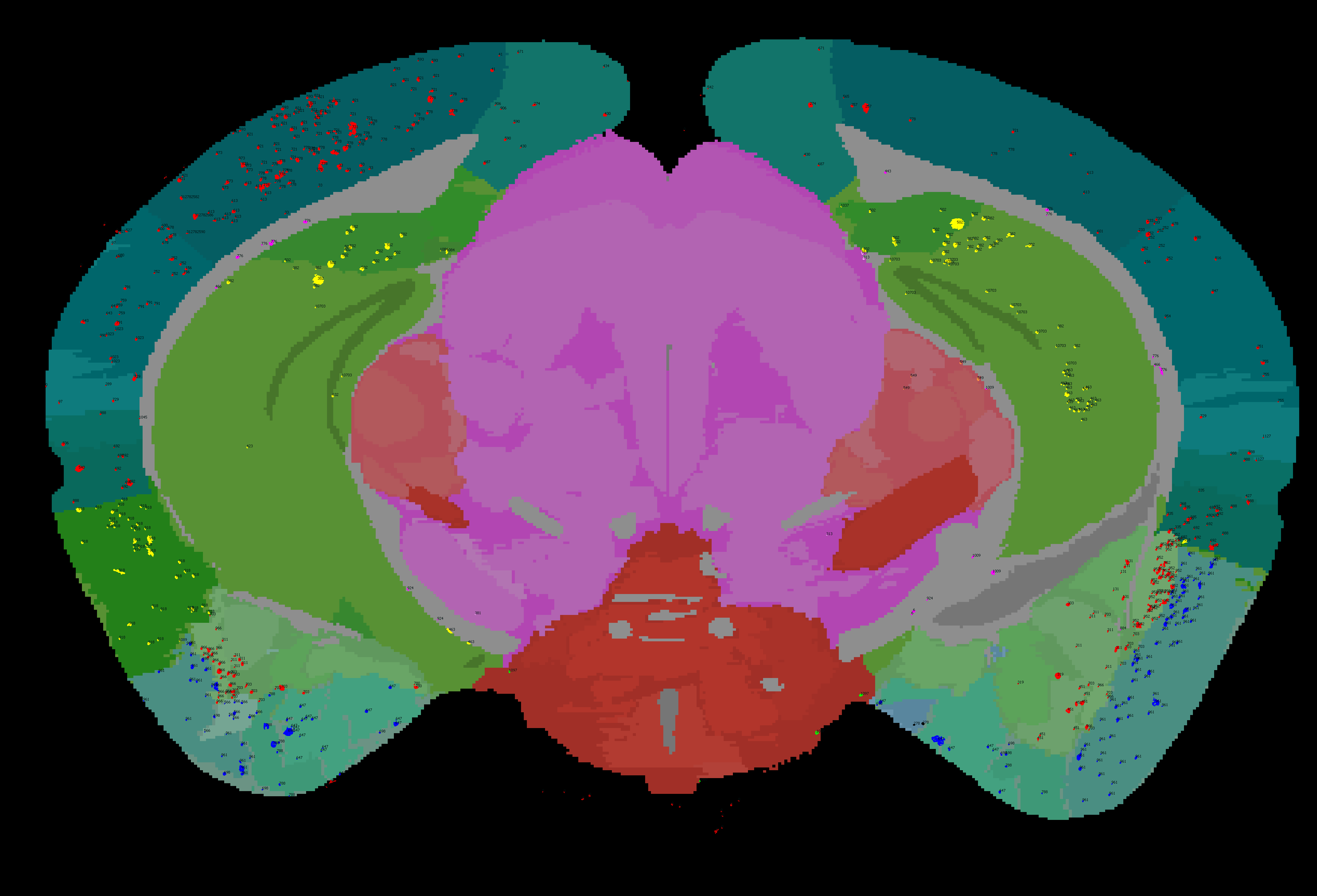

Supplement: Supplementary file 2 [file Data_Sheet_1.ZIP › Supplementary_material_Yates/pE-Abeta/tg2576_m287_pGlu_s184_Object Predictions.png]

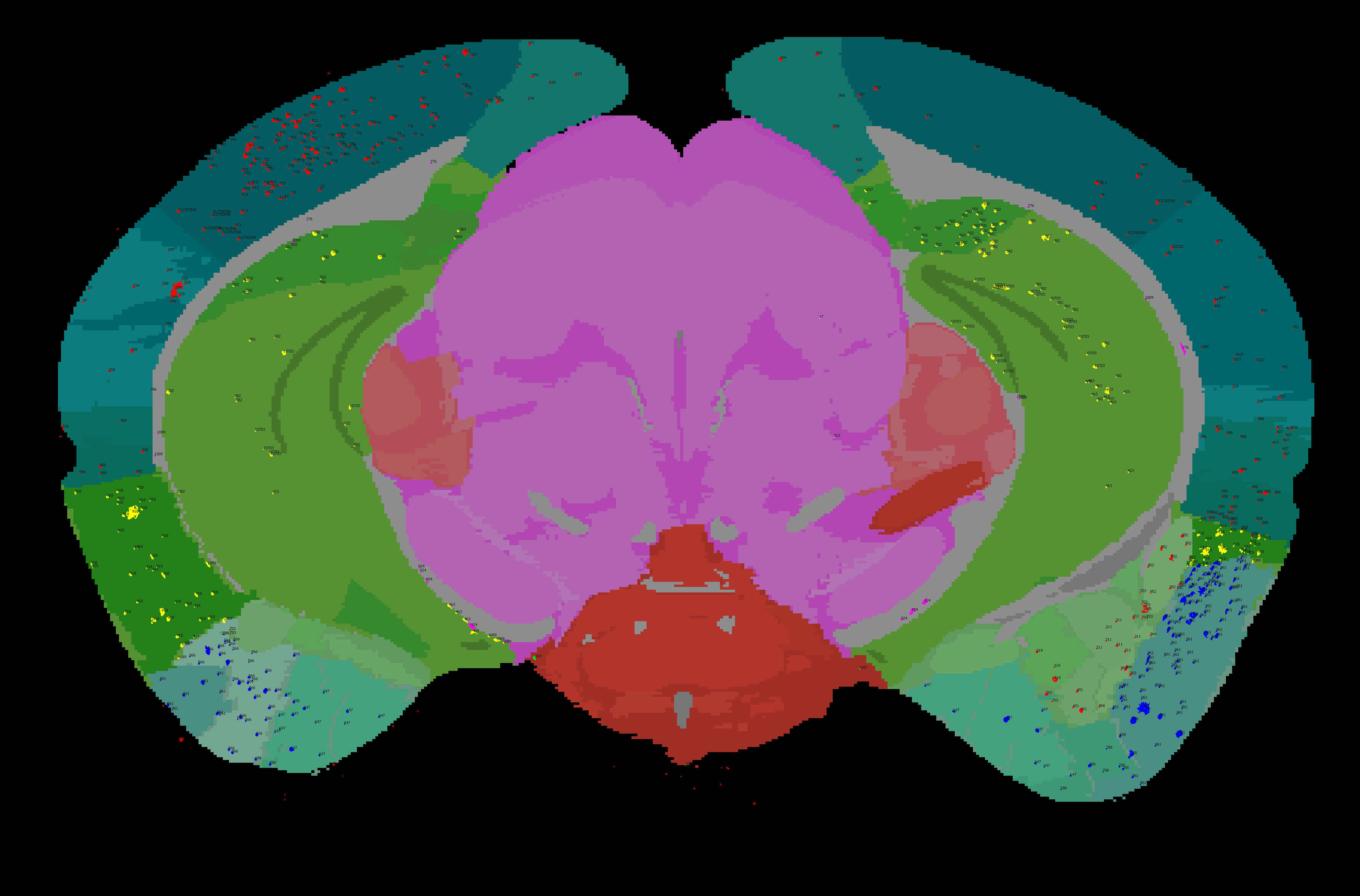

Supplement: Supplementary file 2 [file Data_Sheet_1.ZIP › Supplementary_material_Yates/pE-Abeta/tg2576_m287_pGlu_s188_Object Predictions.png]

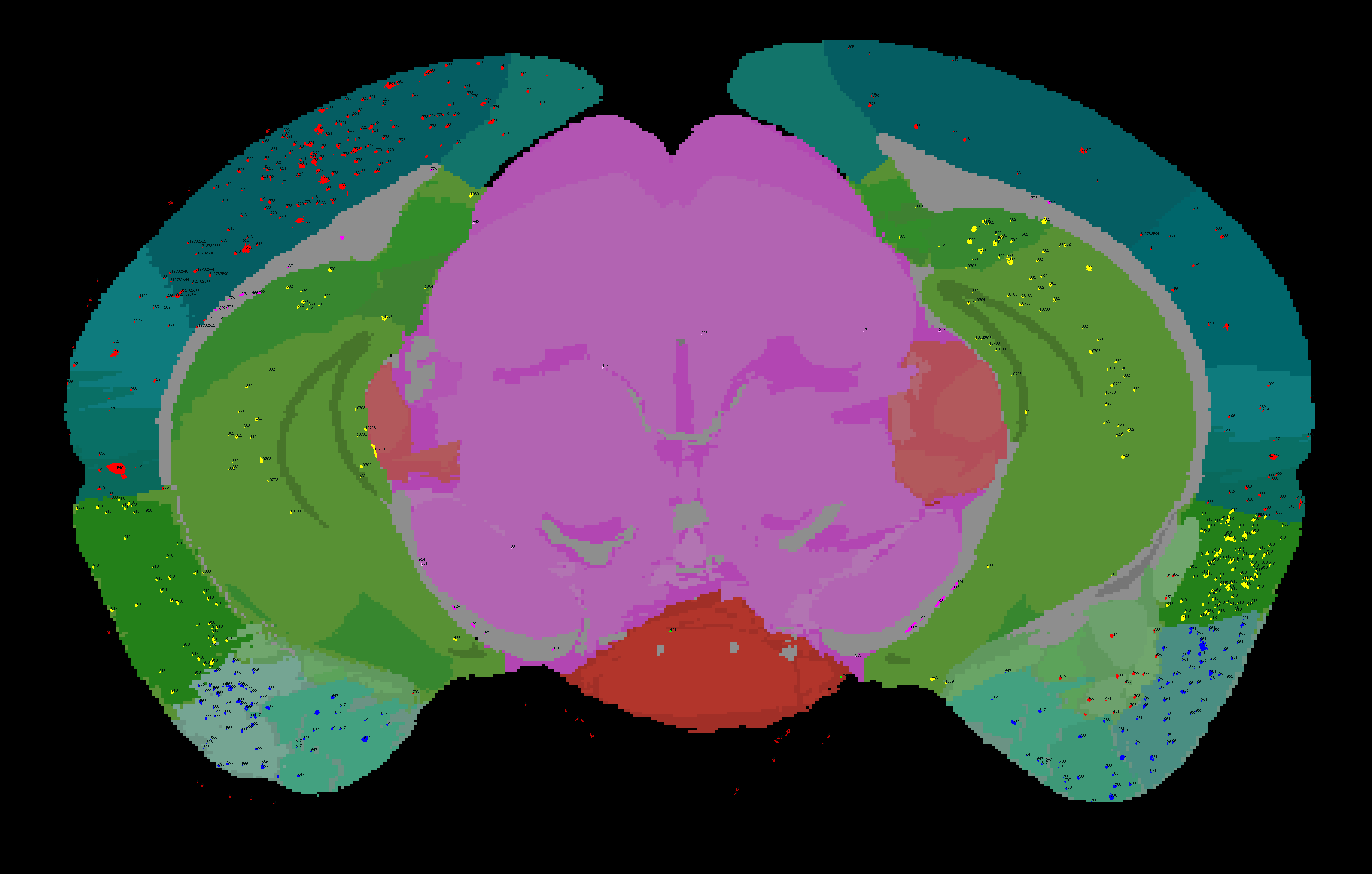

Supplement: Supplementary file 2 [file Data_Sheet_1.ZIP › Supplementary_material_Yates/pE-Abeta/tg2576_m287_pGlu_s192_Object Predictions.png]

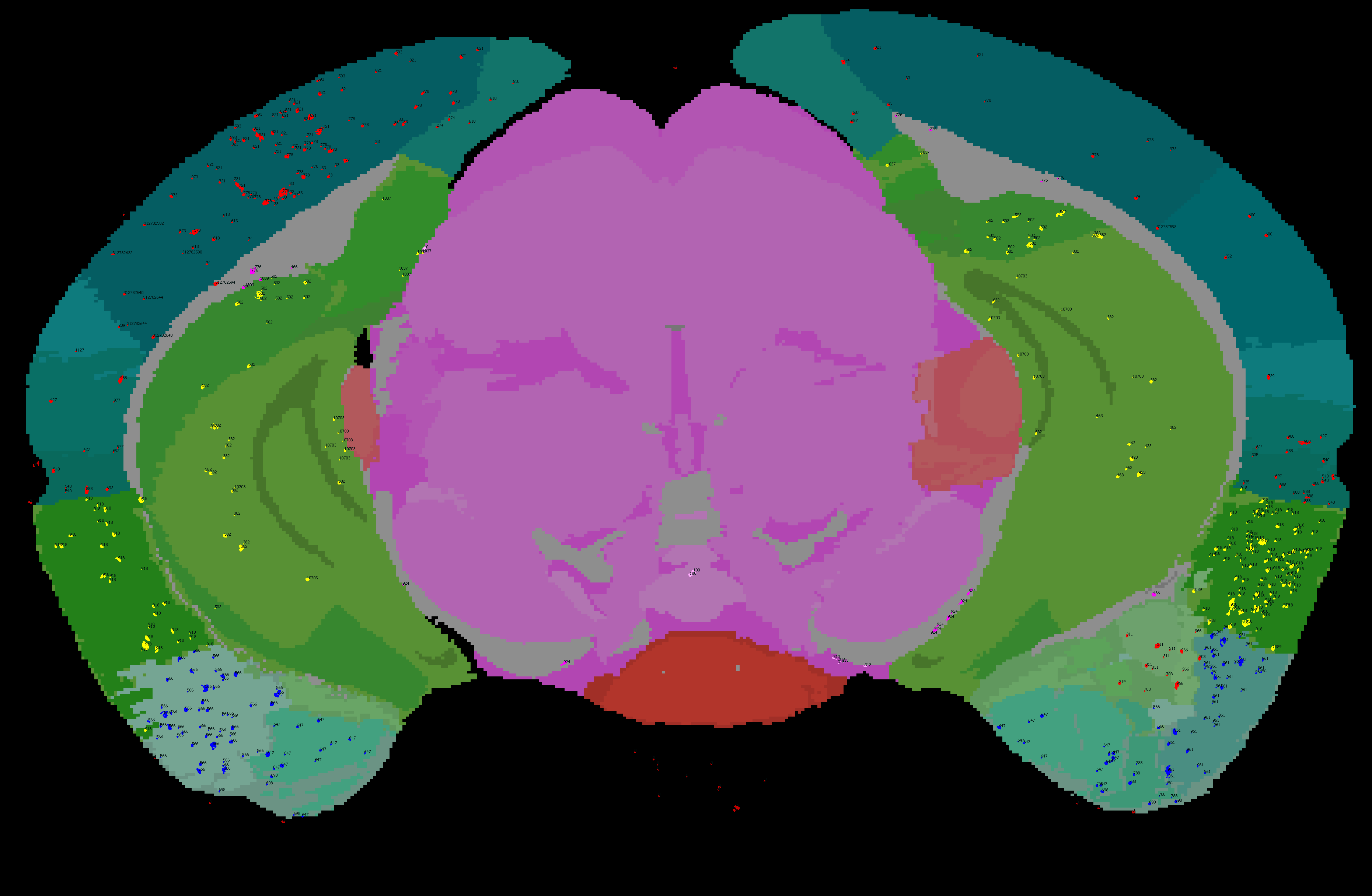

Supplement: Supplementary file 2 [file Data_Sheet_1.ZIP › Supplementary_material_Yates/pE-Abeta/tg2576_m287_pGlu_s196_Object Predictions.png]

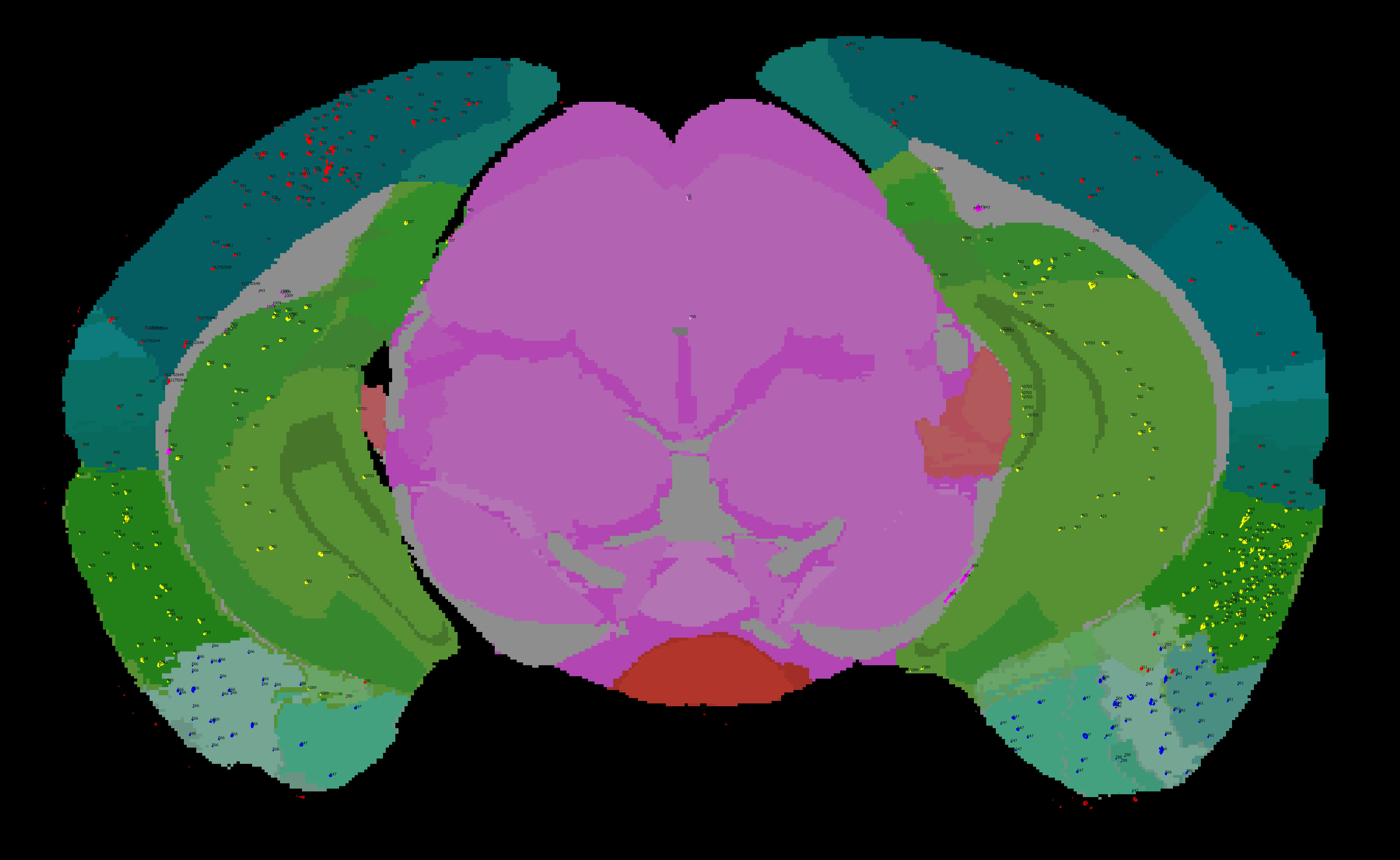

Supplement: Supplementary file 2 [file Data_Sheet_1.ZIP › Supplementary_material_Yates/pE-Abeta/tg2576_m287_pGlu_s200_Object Predictions.png]

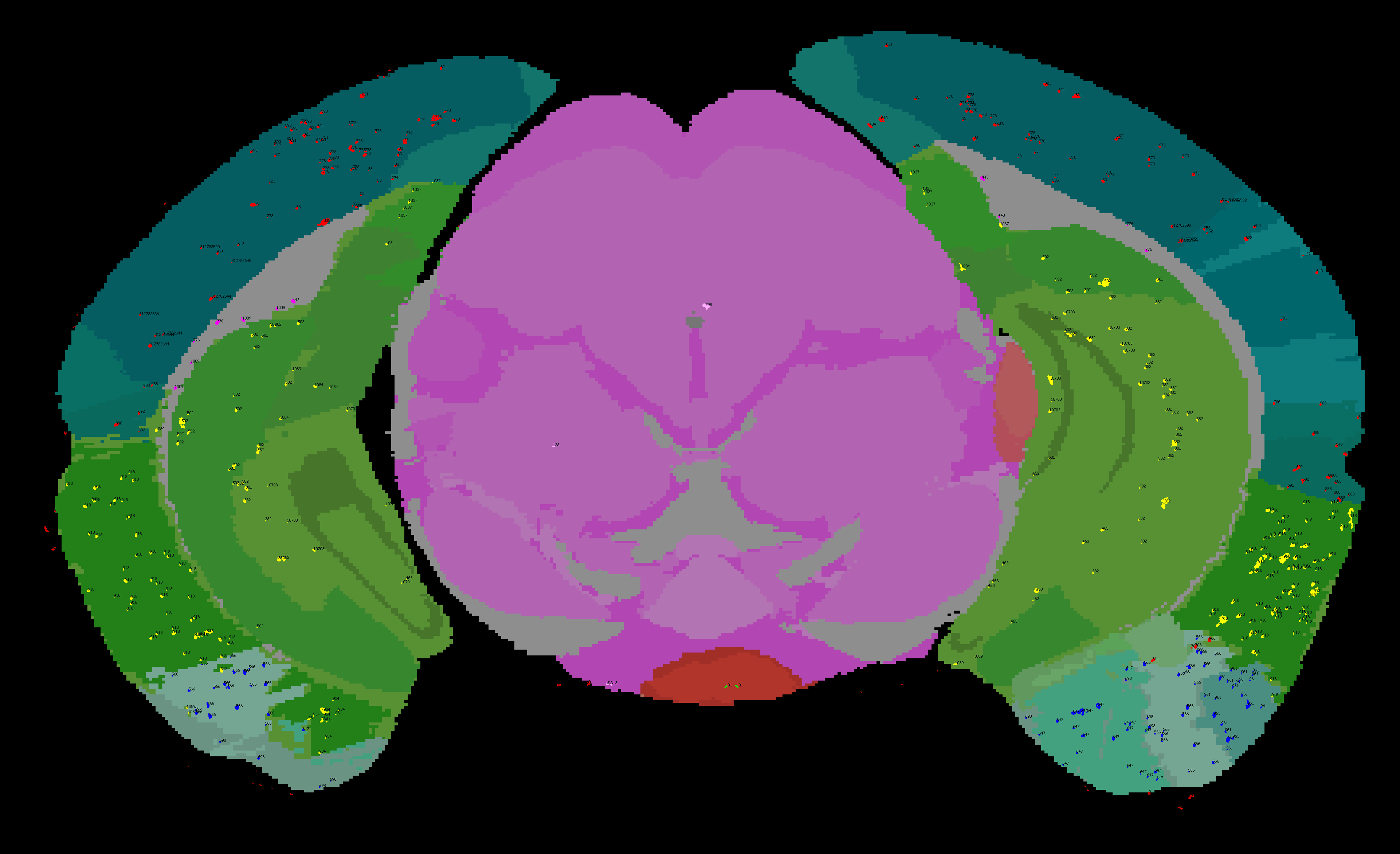

Supplement: Supplementary file 2 [file Data_Sheet_1.ZIP › Supplementary_material_Yates/pE-Abeta/tg2576_m287_pGlu_s204_Object Predictions.png]

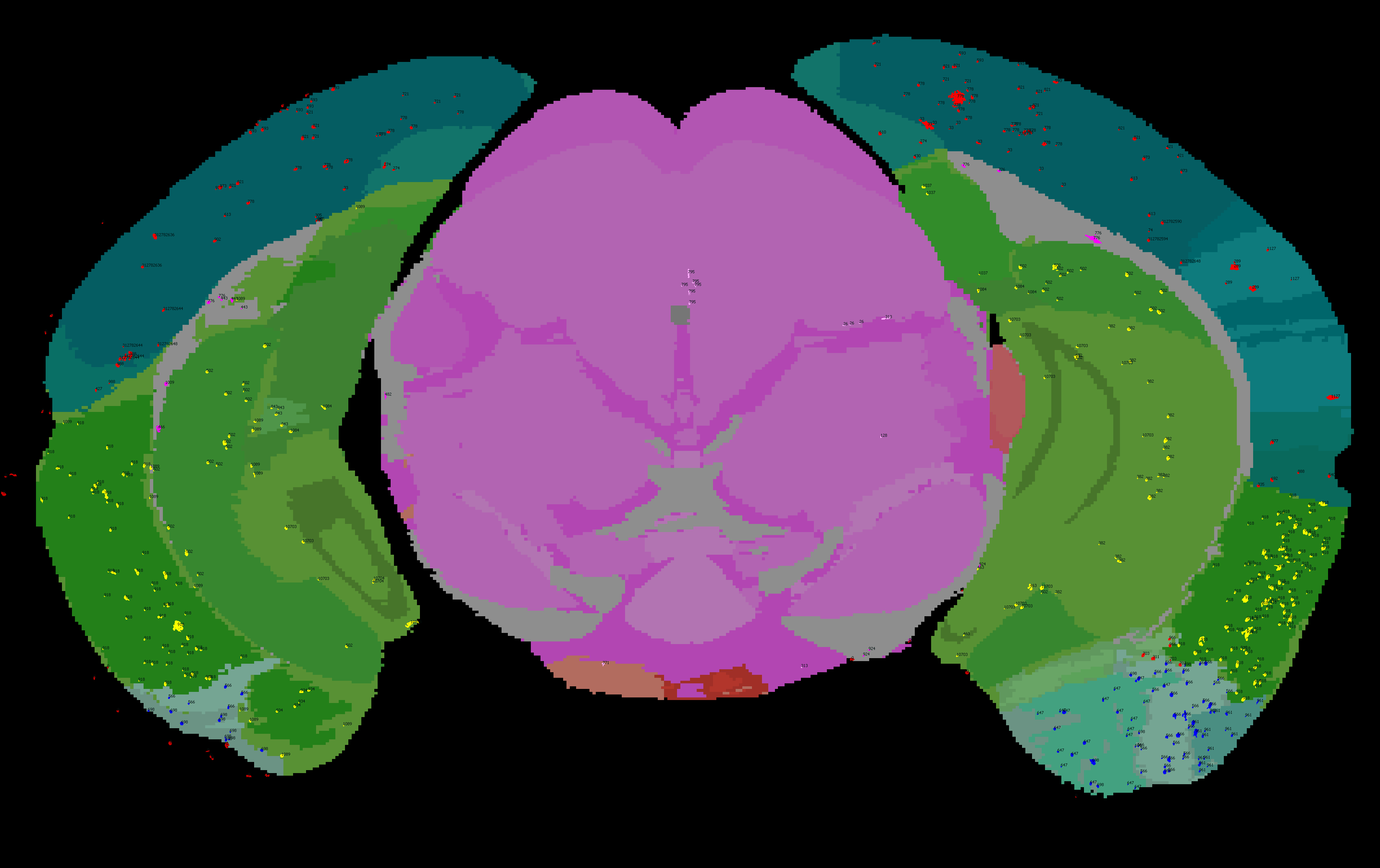

Supplement: Supplementary file 2 [file Data_Sheet_1.ZIP › Supplementary_material_Yates/pE-Abeta/tg2576_m287_pGlu_s208_Object Predictions.png]

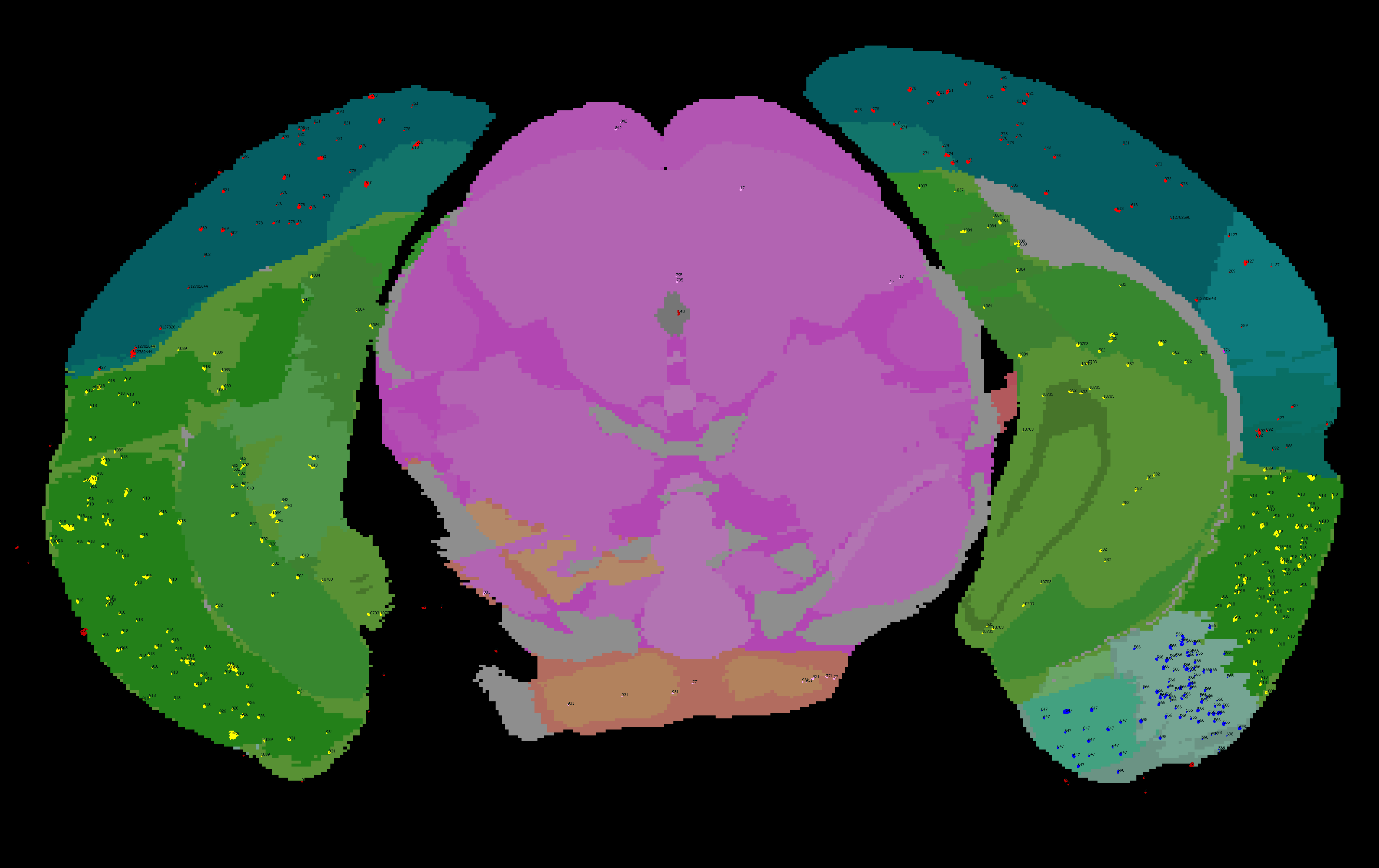

Supplement: Supplementary file 2 [file Data_Sheet_1.ZIP › Supplementary_material_Yates/pE-Abeta/tg2576_m287_pGlu_s212_Object Predictions.png]

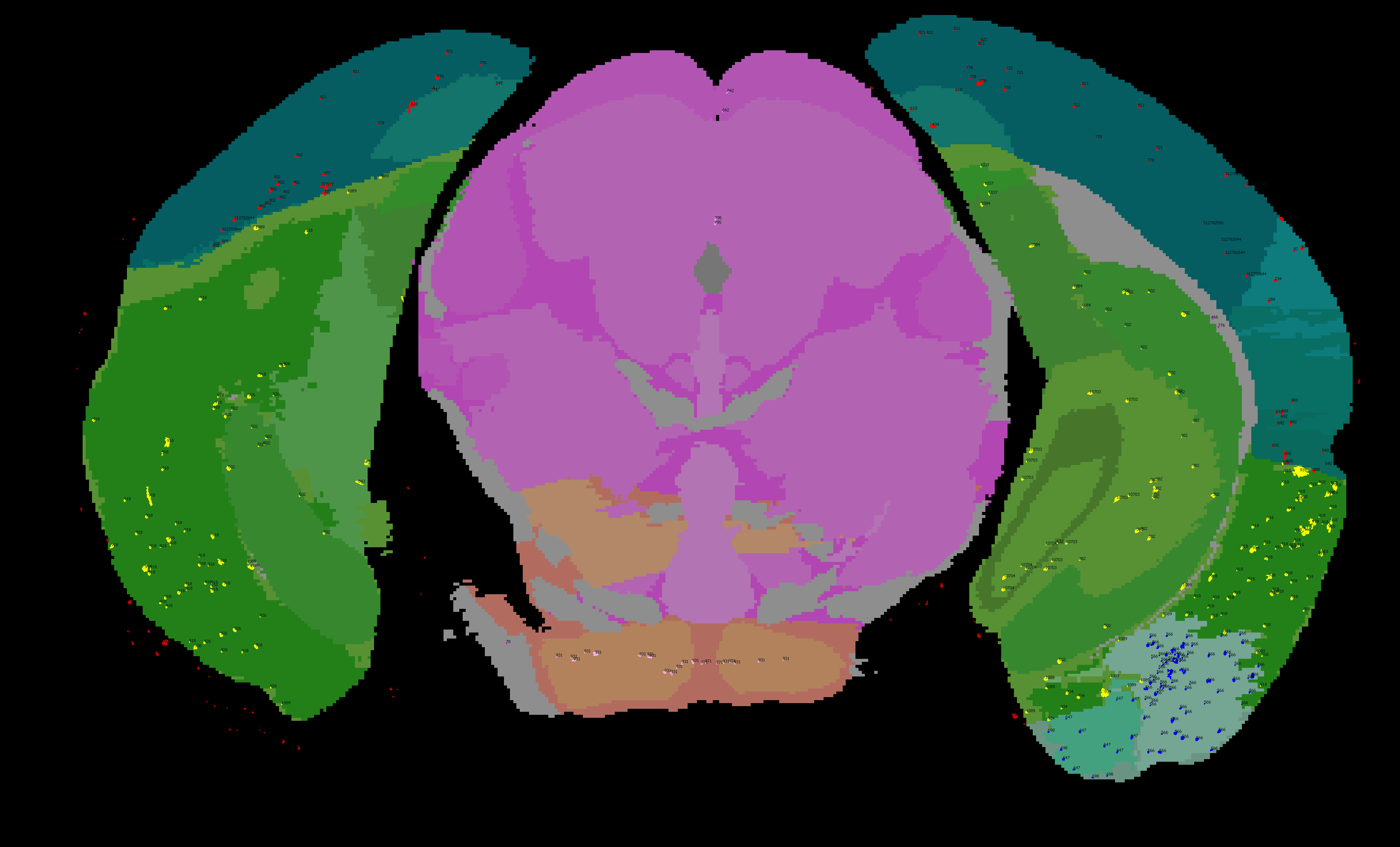

Supplement: Supplementary file 2 [file Data_Sheet_1.ZIP › Supplementary_material_Yates/pE-Abeta/tg2576_m287_pGlu_s216_Object Predictions.png]

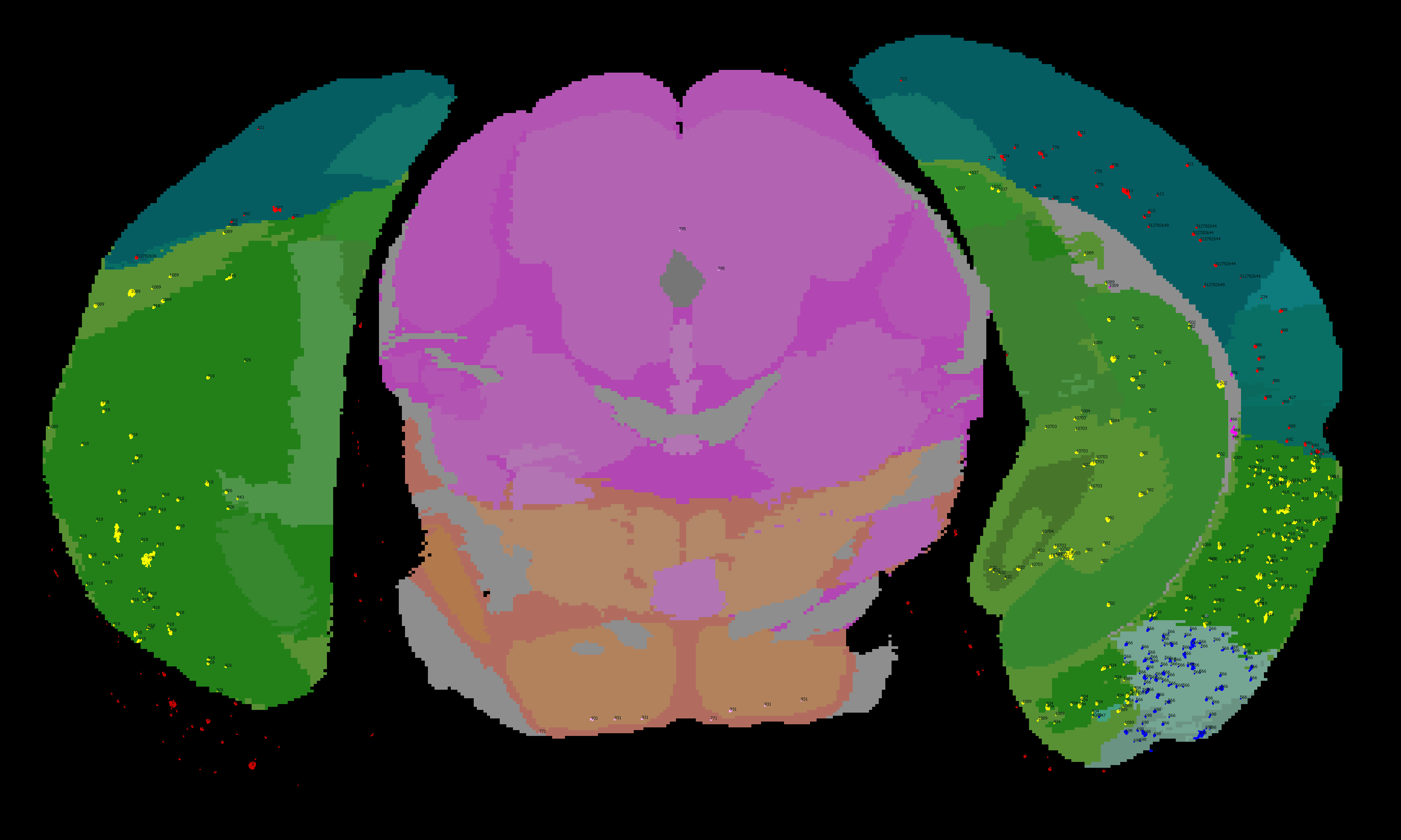

Supplement: Supplementary file 2 [file Data_Sheet_1.ZIP › Supplementary_material_Yates/pE-Abeta/tg2576_m287_pGlu_s220_Object Predictions.png]

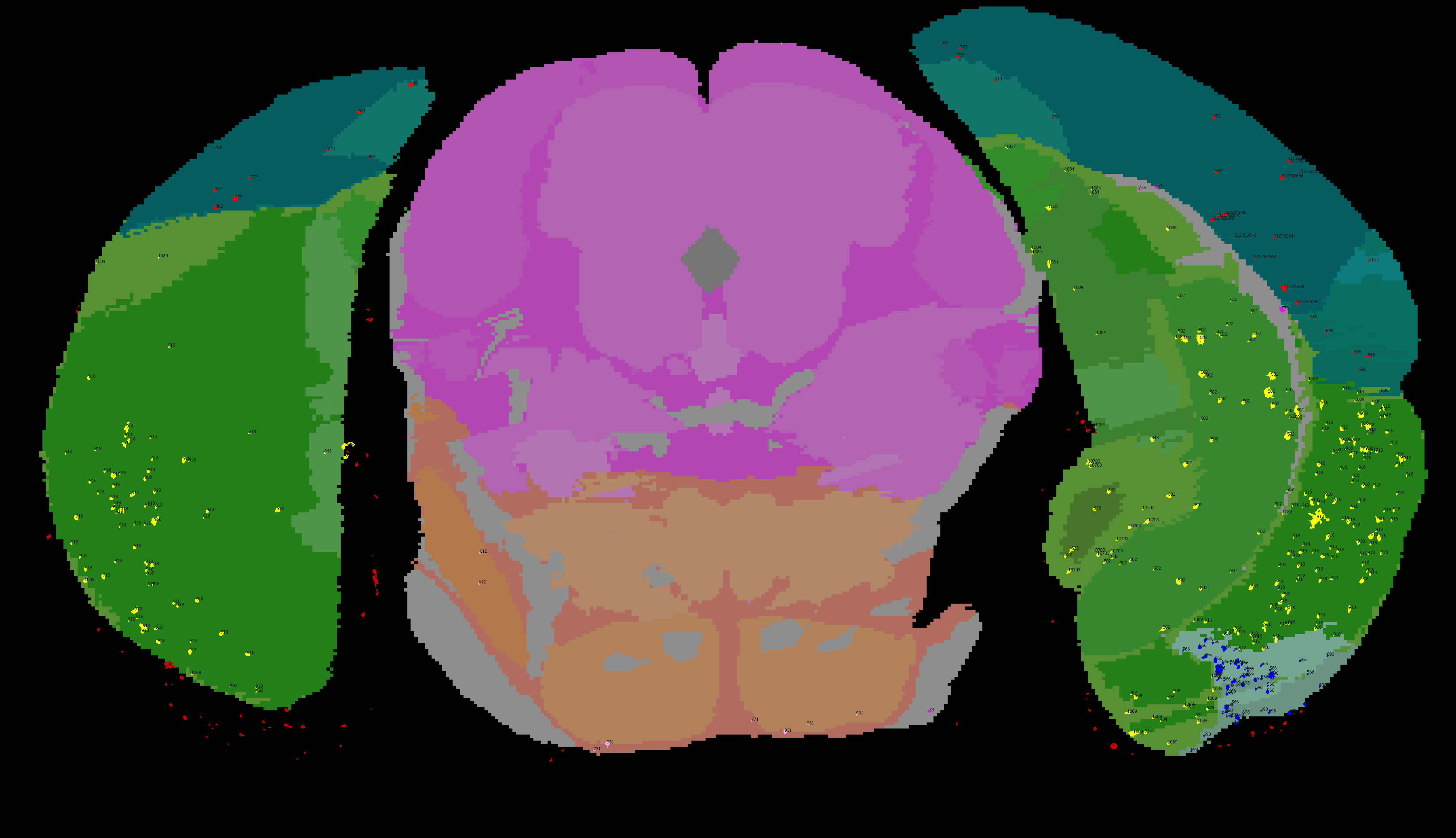

Supplement: Supplementary file 2 [file Data_Sheet_1.ZIP › Supplementary_material_Yates/pE-Abeta/tg2576_m287_pGlu_s224_Object Predictions.png]

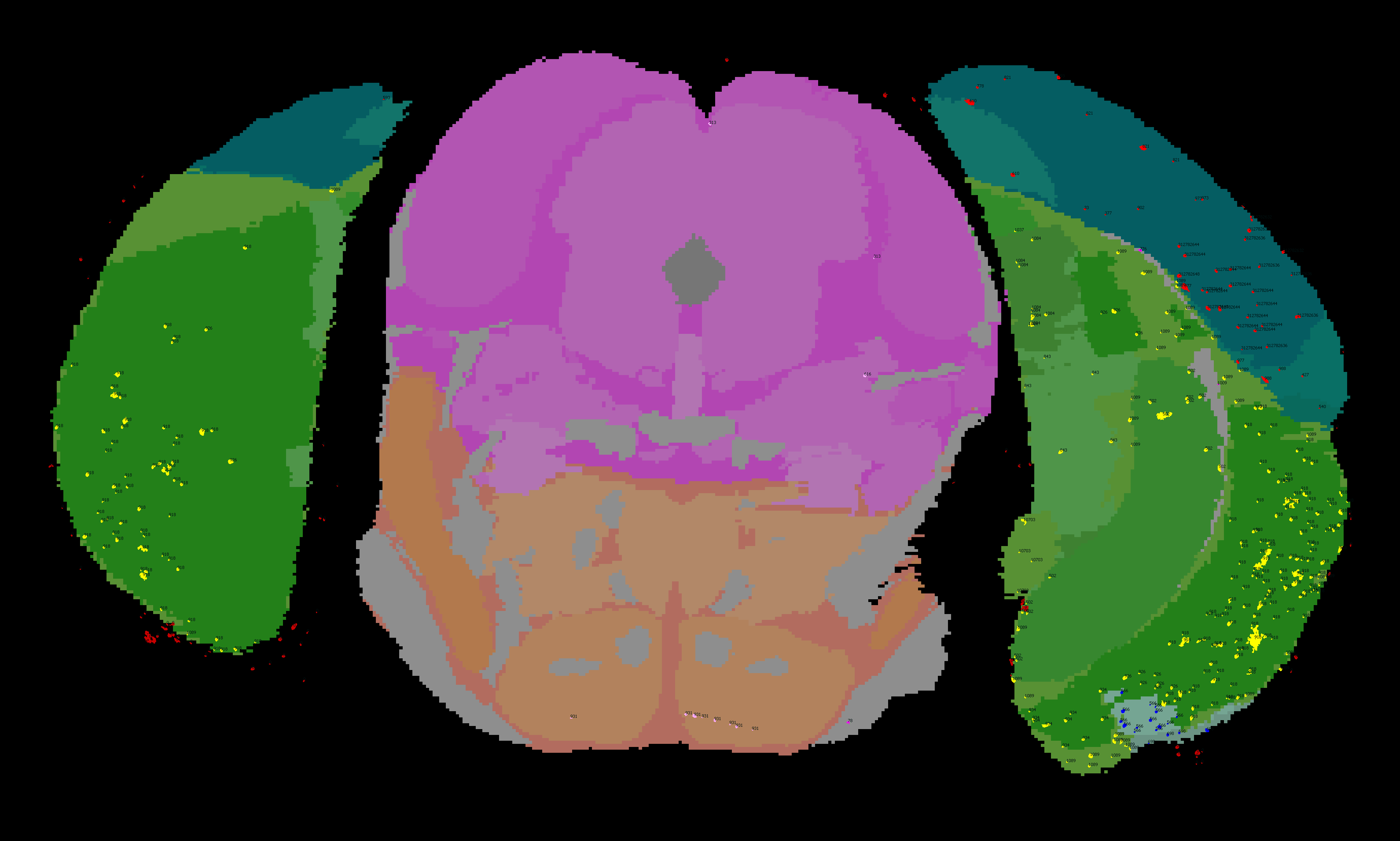

Supplement: Supplementary file 2 [file Data_Sheet_1.ZIP › Supplementary_material_Yates/pE-Abeta/tg2576_m287_pGlu_s228_Object Predictions.png]

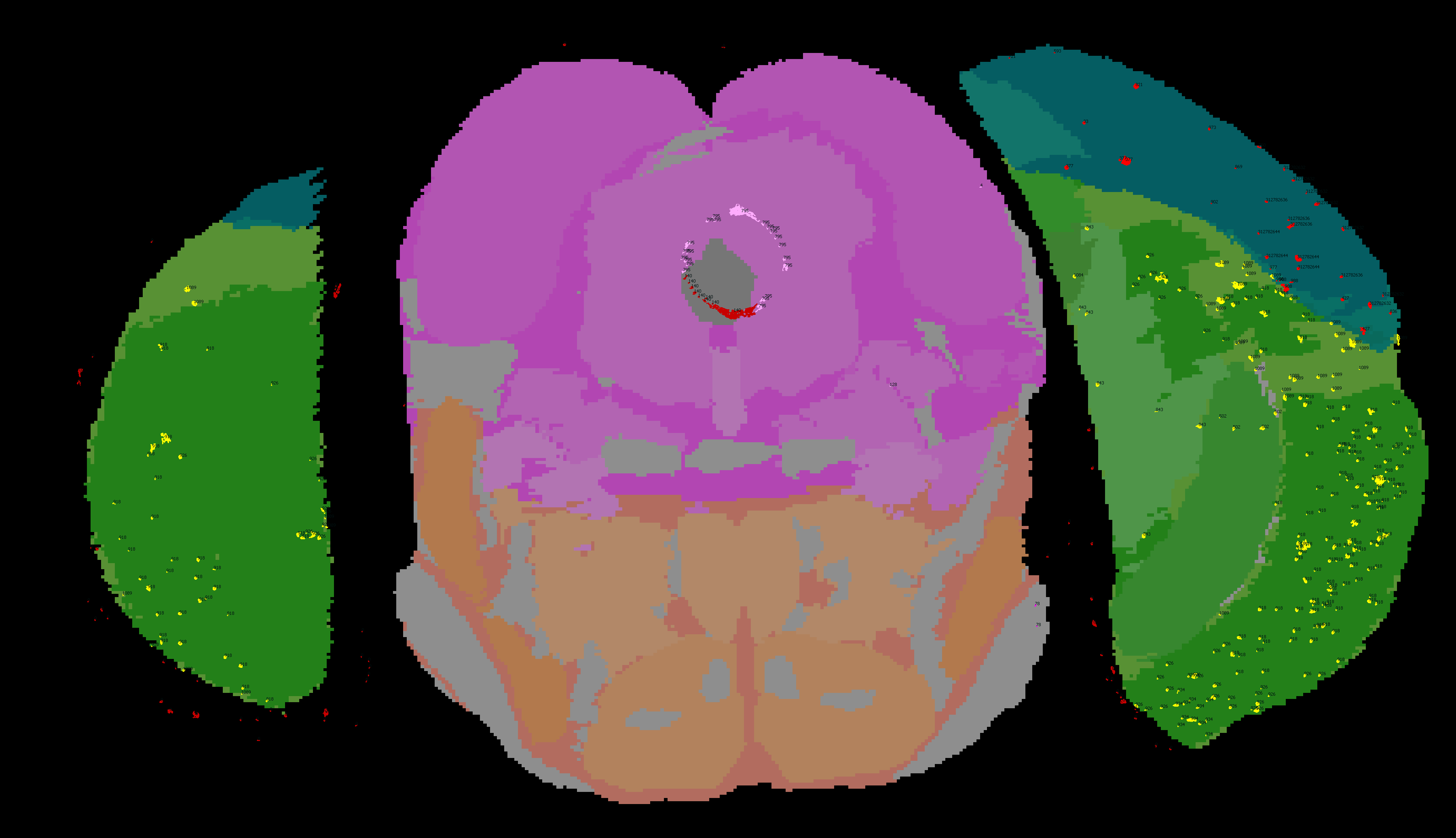

Supplement: Supplementary file 2 [file Data_Sheet_1.ZIP › Supplementary_material_Yates/pE-Abeta/tg2576_m287_pGlu_s232_Object Predictions.png]

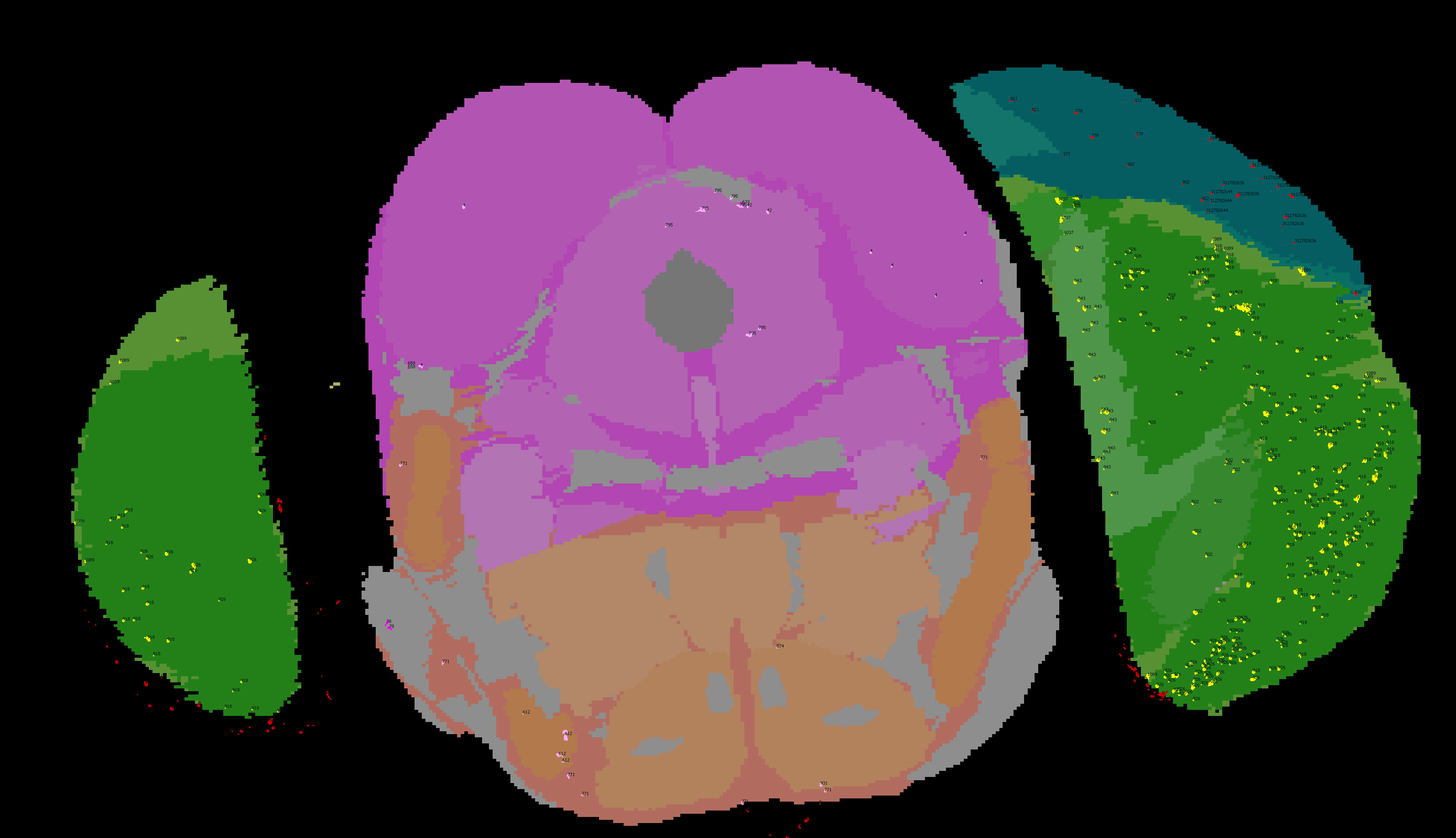

Supplement: Supplementary file 2 [file Data_Sheet_1.ZIP › Supplementary_material_Yates/pE-Abeta/tg2576_m287_pGlu_s236_Object Predictions.png]

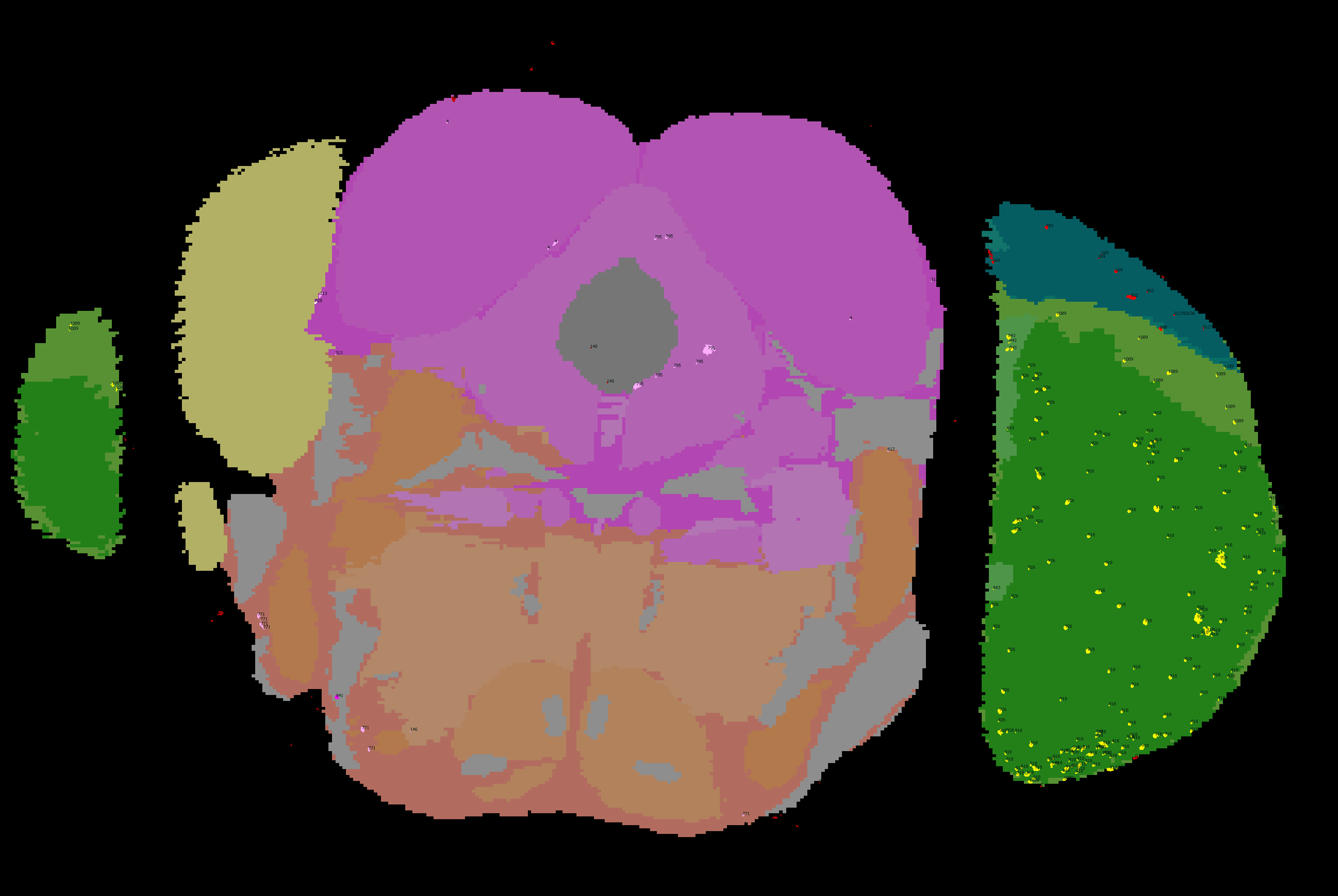

Supplement: Supplementary file 2 [file Data_Sheet_1.ZIP › Supplementary_material_Yates/pE-Abeta/tg2576_m287_pGlu_s240_Object Predictions.png]
