# Supplementary figures and images for: QUINT: Workflow for Quantification and Spatial Analysis of Features in Histological Images From Rodent Brain (part 1 of 2)
Source: Front Neuroinform. 2019 Dec 3;13:75. doi: 10.3389/fninf.2019.00075 (PMC6901597; doi:10.3389/fninf.2019.00075)

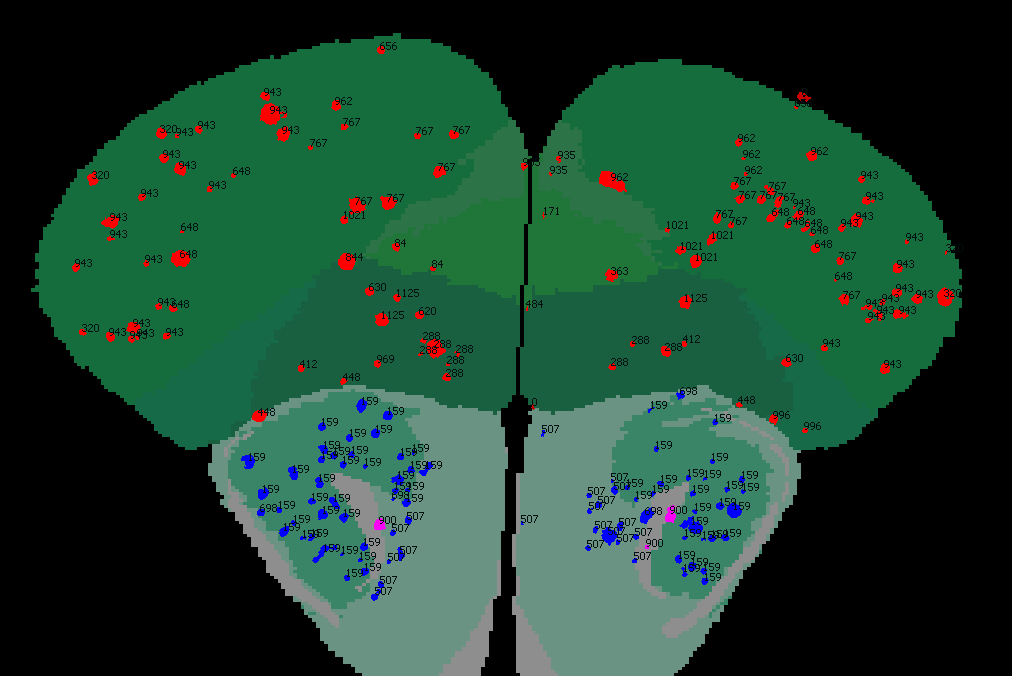

Supplement: Supplementary file 2 [file Data_Sheet_1.ZIP › Supplementary_material_Yates/hAPP/tg2576_m287_1D1_s002_resize_Object Predictions.png]

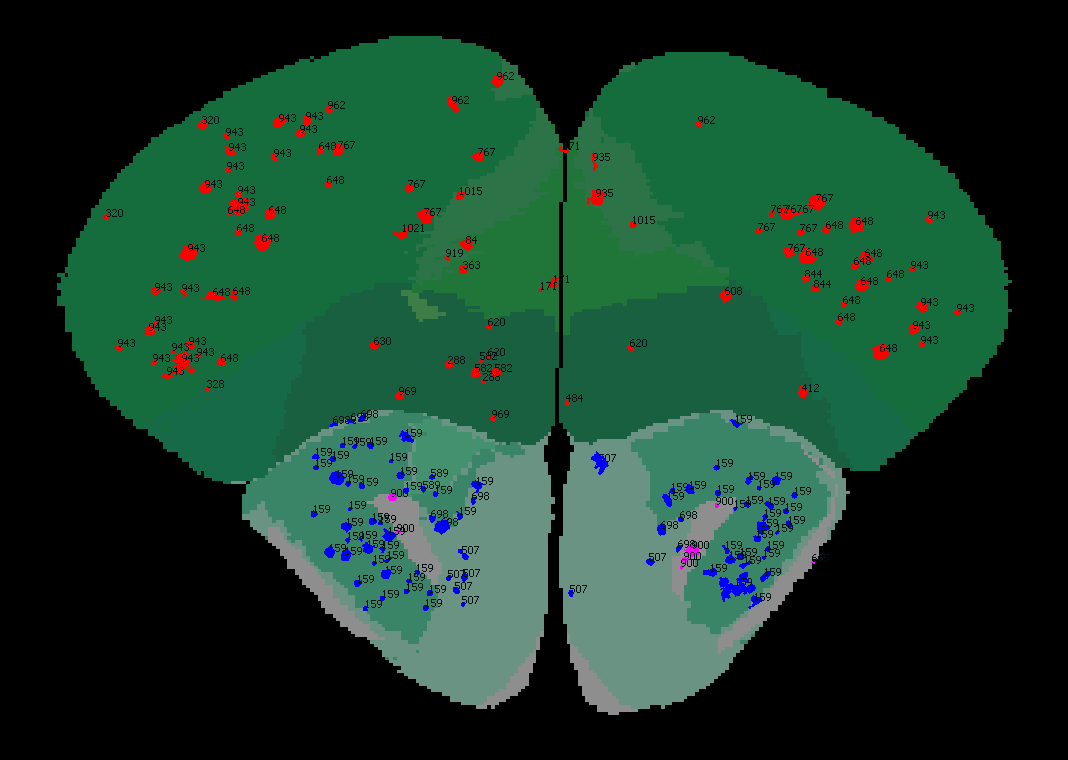

Supplement: Supplementary file 2 [file Data_Sheet_1.ZIP › Supplementary_material_Yates/hAPP/tg2576_m287_1D1_s006_resize_Object Predictions.png]

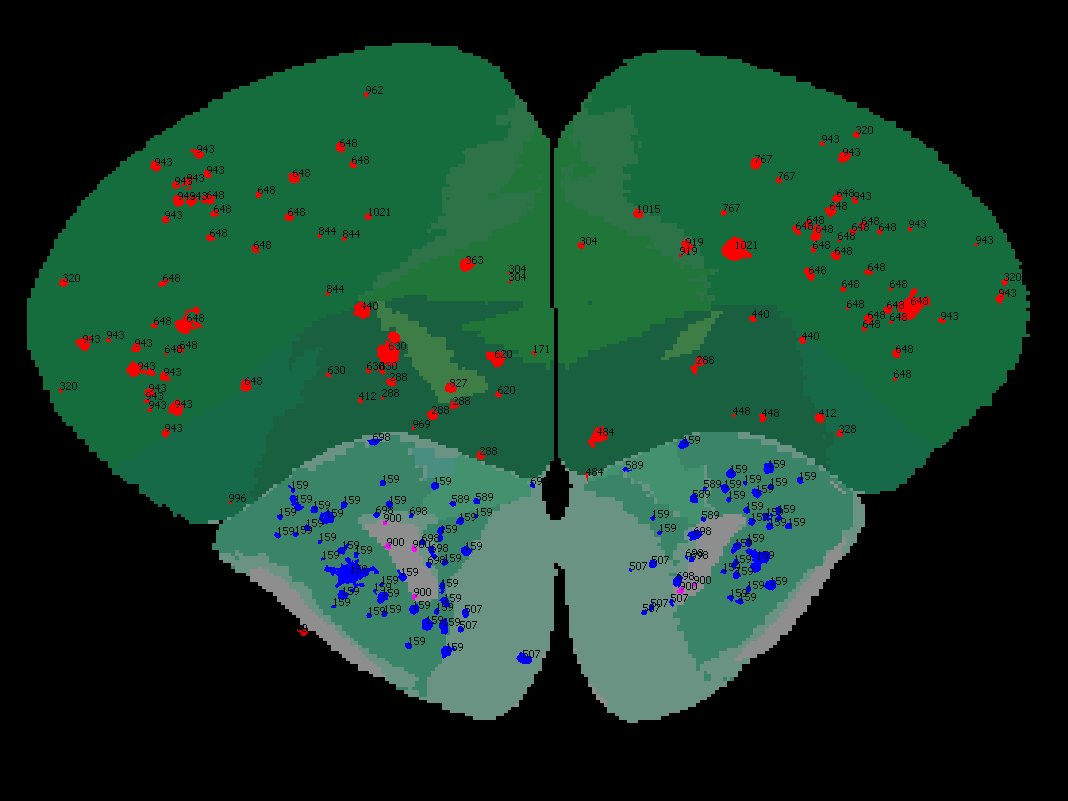

Supplement: Supplementary file 2 [file Data_Sheet_1.ZIP › Supplementary_material_Yates/hAPP/tg2576_m287_1D1_s010_resize_Object Predictions.png]

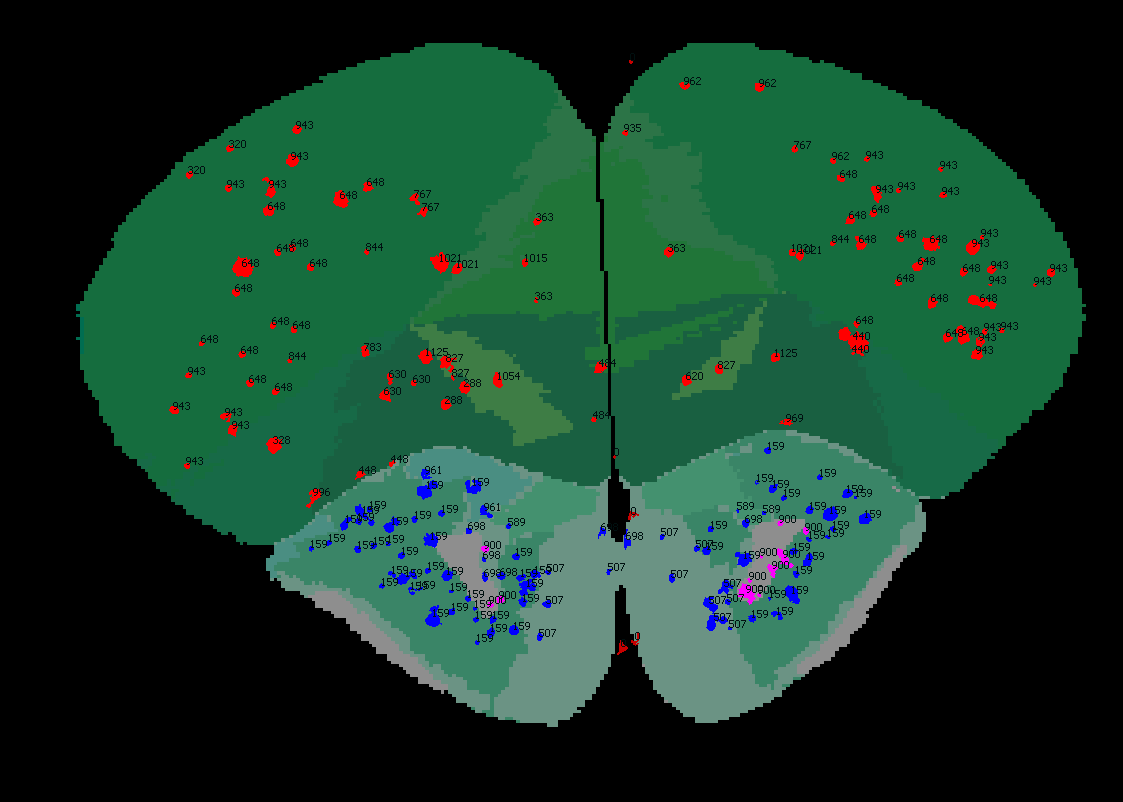

Supplement: Supplementary file 2 [file Data_Sheet_1.ZIP › Supplementary_material_Yates/hAPP/tg2576_m287_1D1_s014_resize_Object Predictions.png]

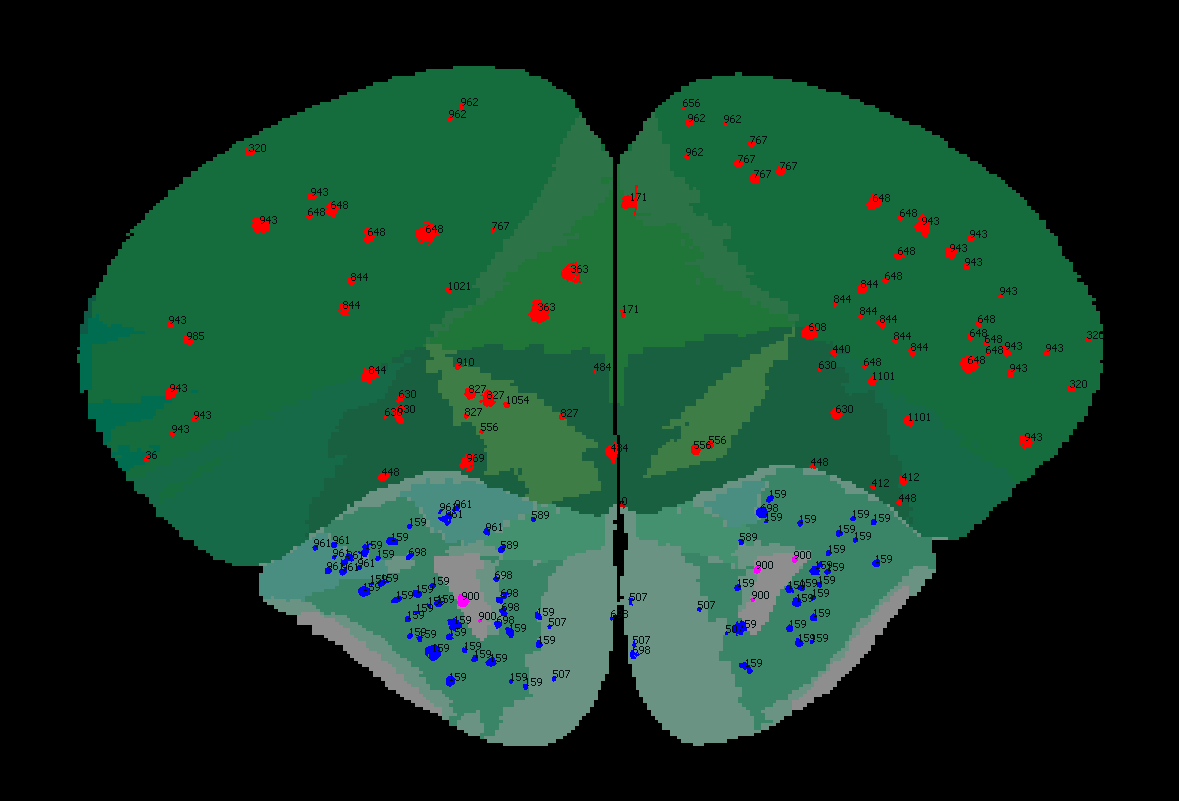

Supplement: Supplementary file 2 [file Data_Sheet_1.ZIP › Supplementary_material_Yates/hAPP/tg2576_m287_1D1_s018_resize_Object Predictions.png]

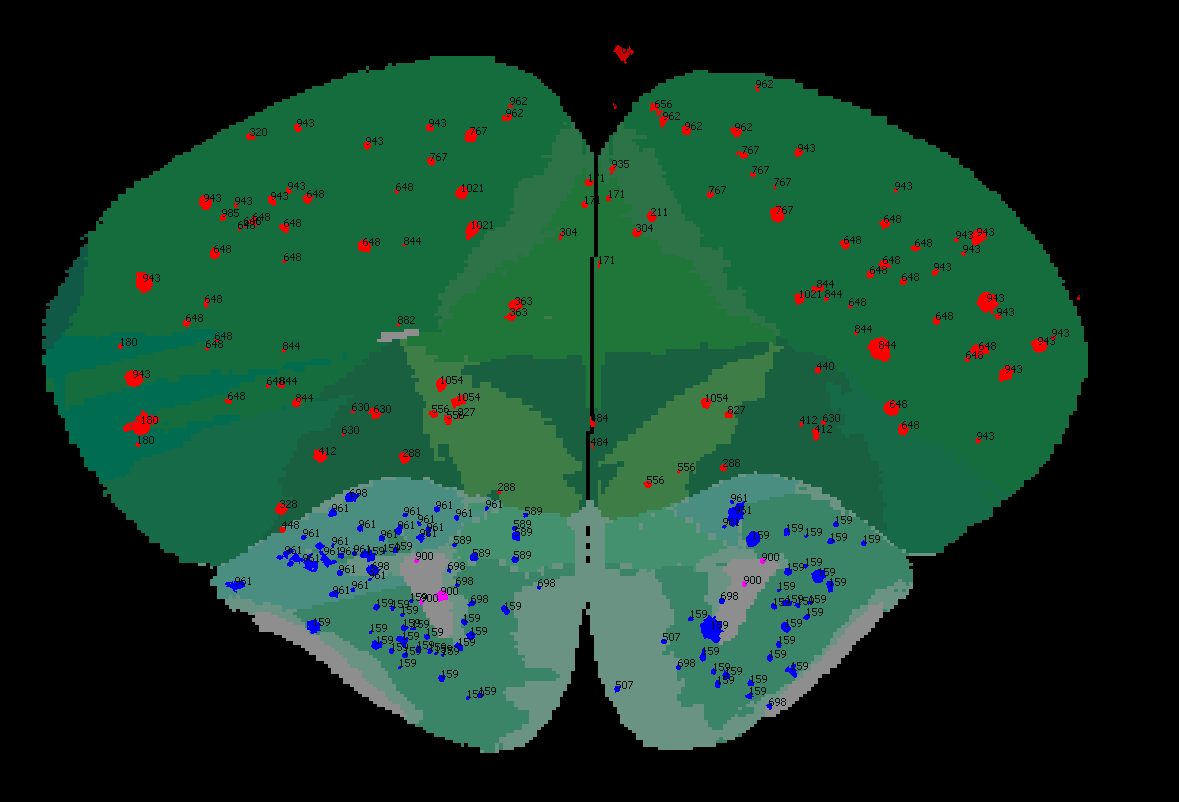

Supplement: Supplementary file 2 [file Data_Sheet_1.ZIP › Supplementary_material_Yates/hAPP/tg2576_m287_1D1_s022_resize_Object Predictions.png]

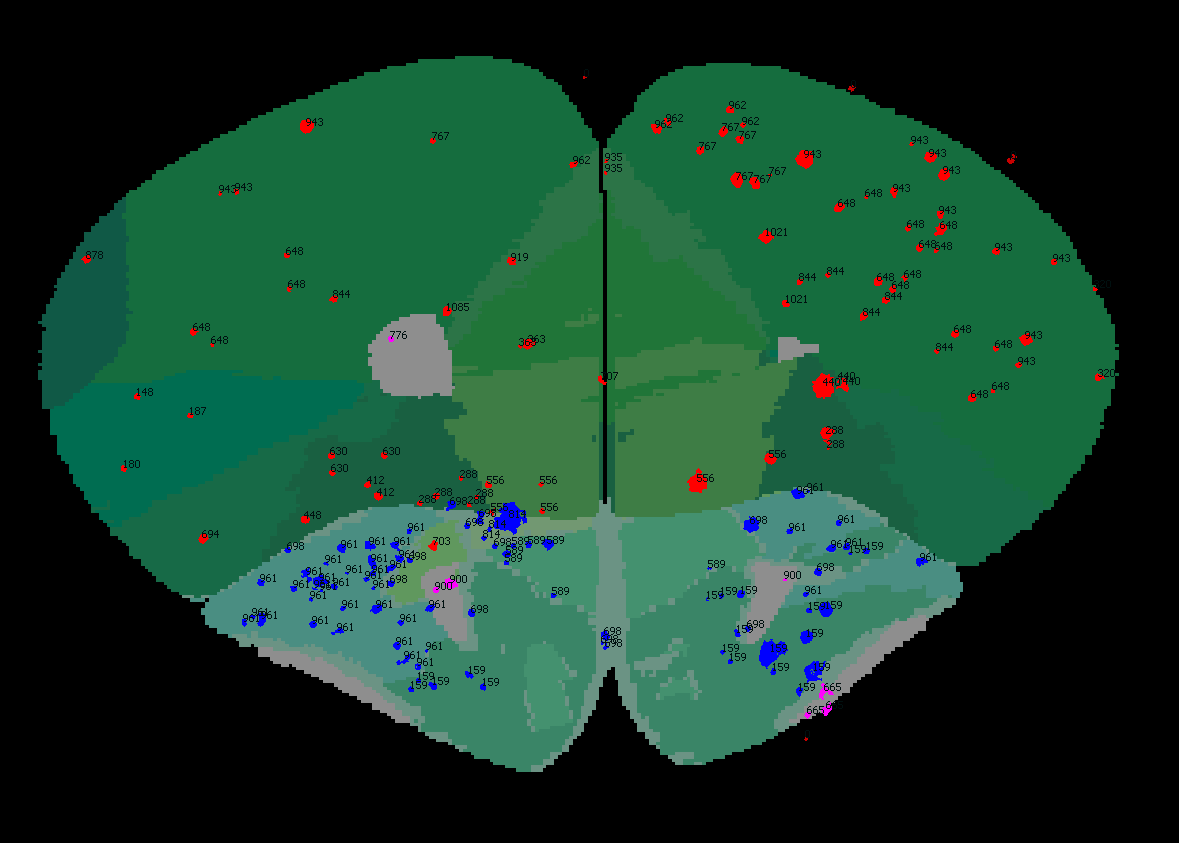

Supplement: Supplementary file 2 [file Data_Sheet_1.ZIP › Supplementary_material_Yates/hAPP/tg2576_m287_1D1_s026_resize_Object Predictions.png]

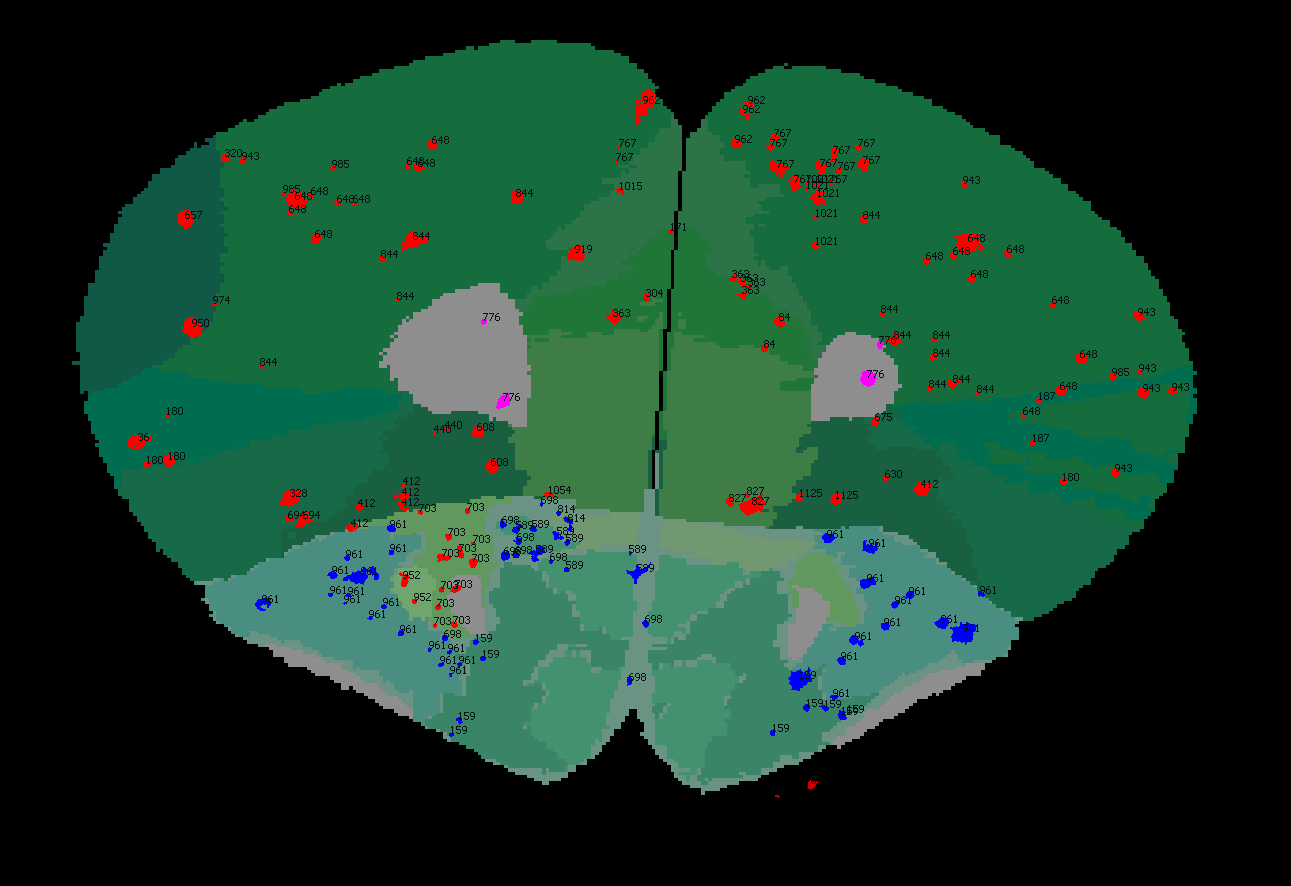

Supplement: Supplementary file 2 [file Data_Sheet_1.ZIP › Supplementary_material_Yates/hAPP/tg2576_m287_1D1_s030_resize_Object Predictions.png]

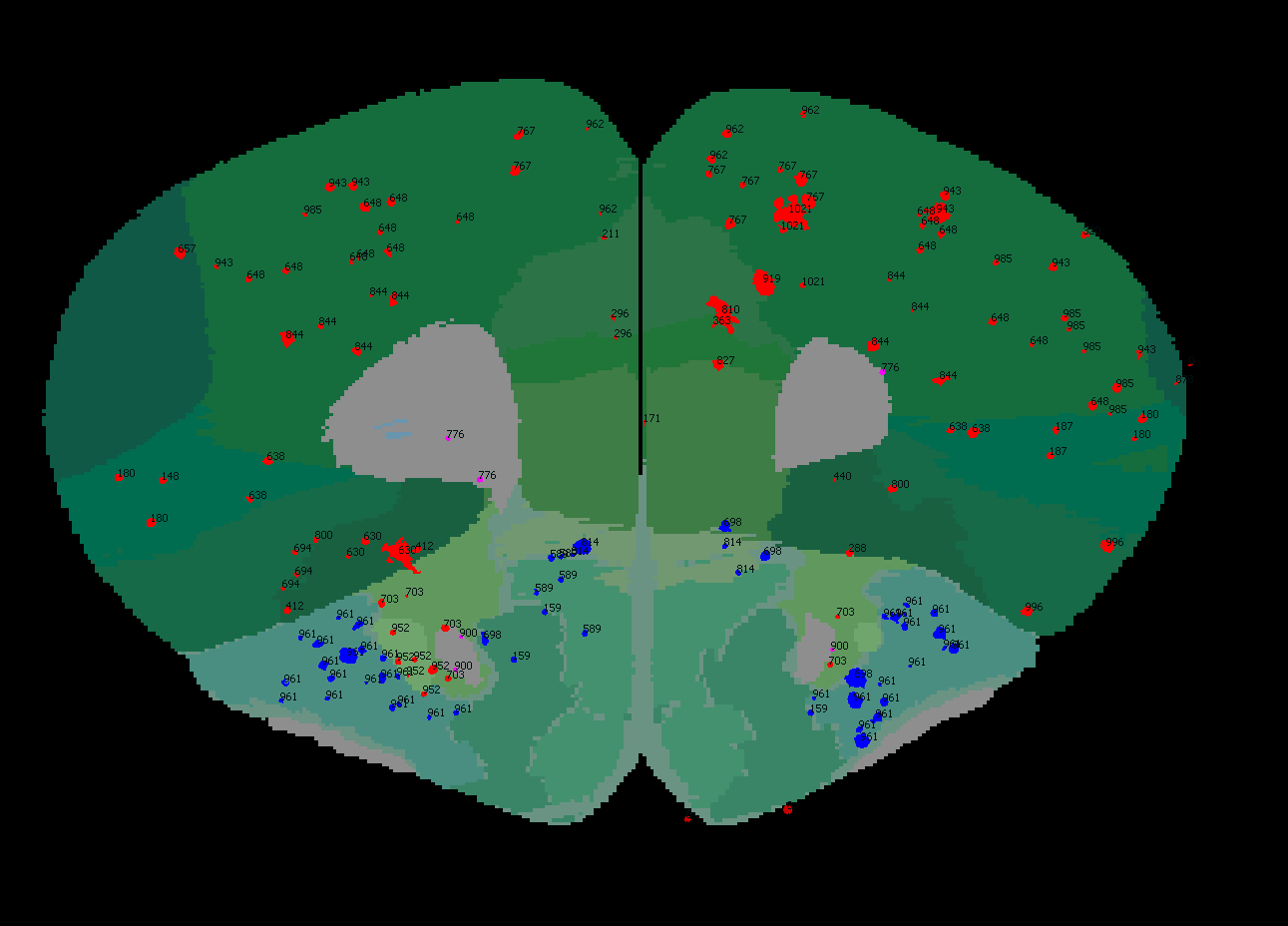

Supplement: Supplementary file 2 [file Data_Sheet_1.ZIP › Supplementary_material_Yates/hAPP/tg2576_m287_1D1_s034_resize_Object Predictions.png]

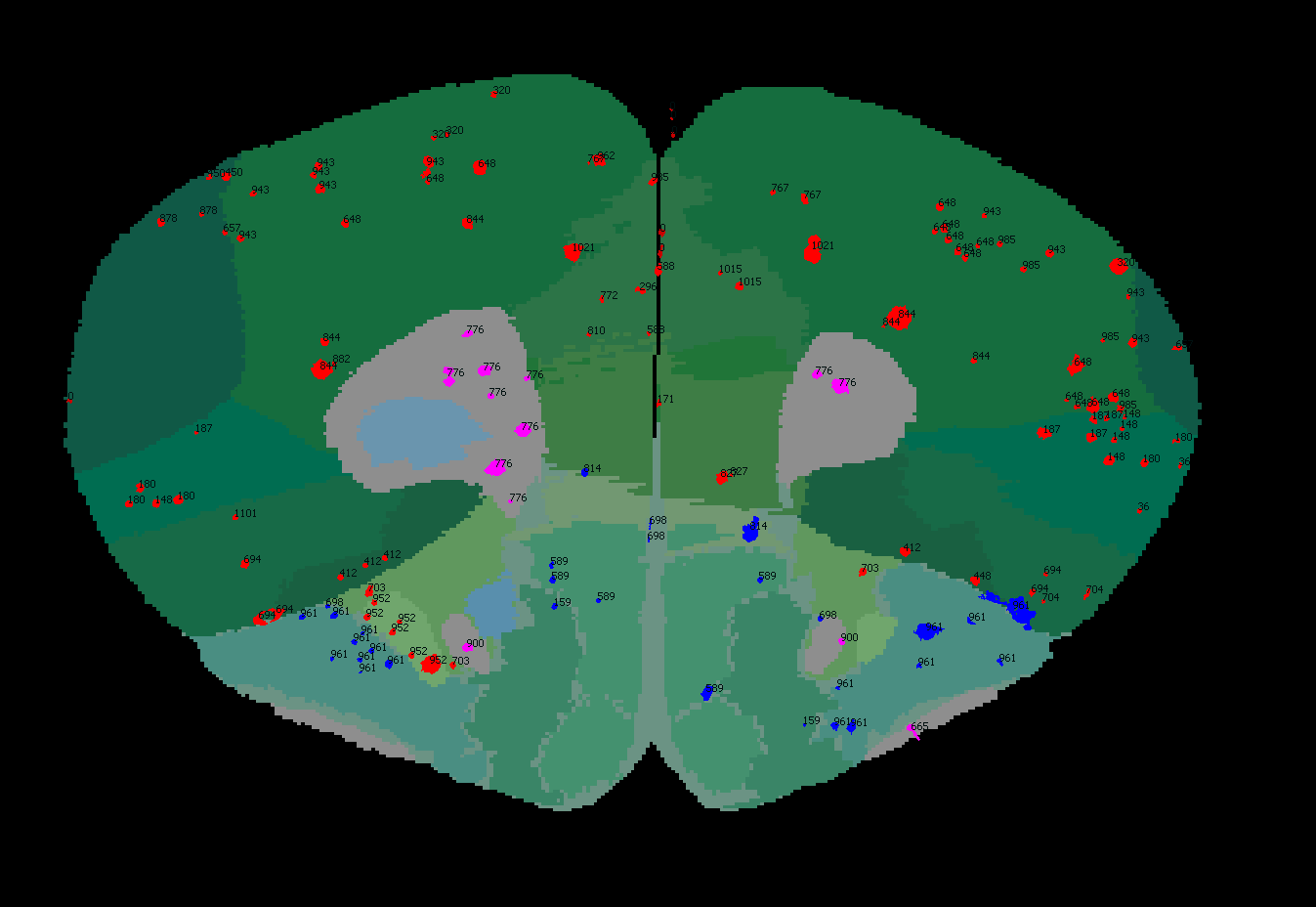

Supplement: Supplementary file 2 [file Data_Sheet_1.ZIP › Supplementary_material_Yates/hAPP/tg2576_m287_1D1_s038_resize_Object Predictions.png]

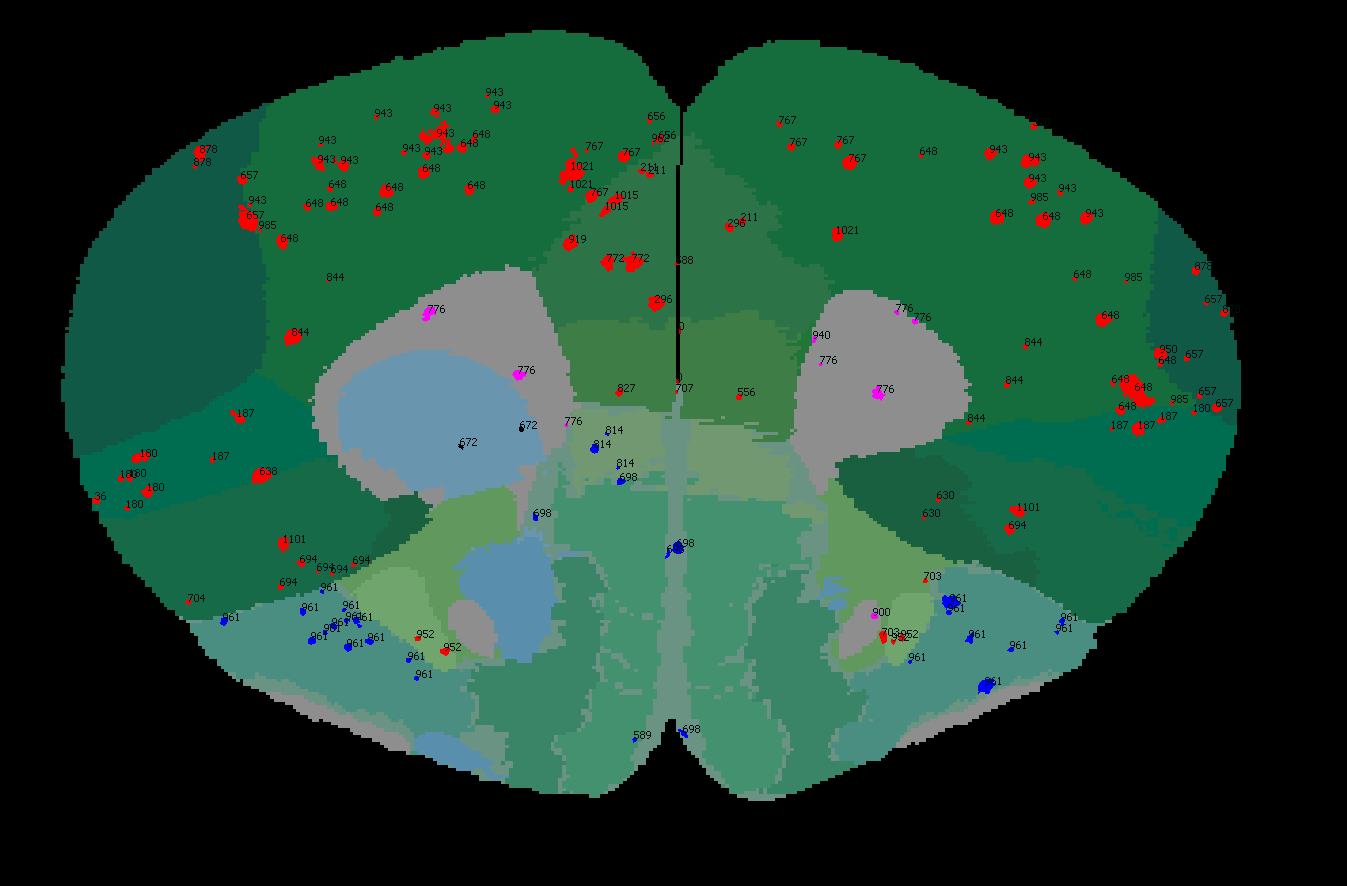

Supplement: Supplementary file 2 [file Data_Sheet_1.ZIP › Supplementary_material_Yates/hAPP/tg2576_m287_1D1_s042_resize_Object Predictions.png]

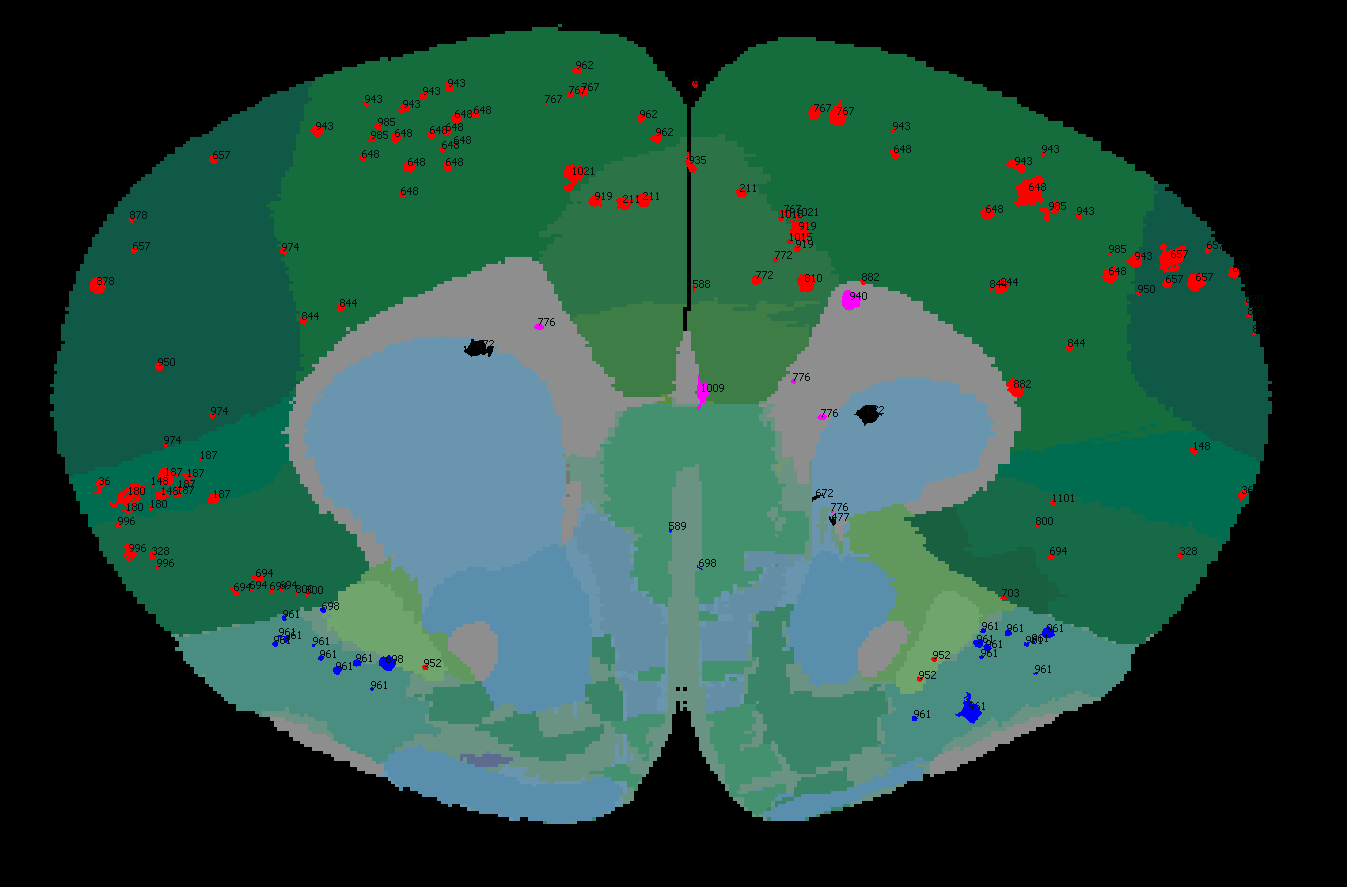

Supplement: Supplementary file 2 [file Data_Sheet_1.ZIP › Supplementary_material_Yates/hAPP/tg2576_m287_1D1_s046_resize_Object Predictions.png]

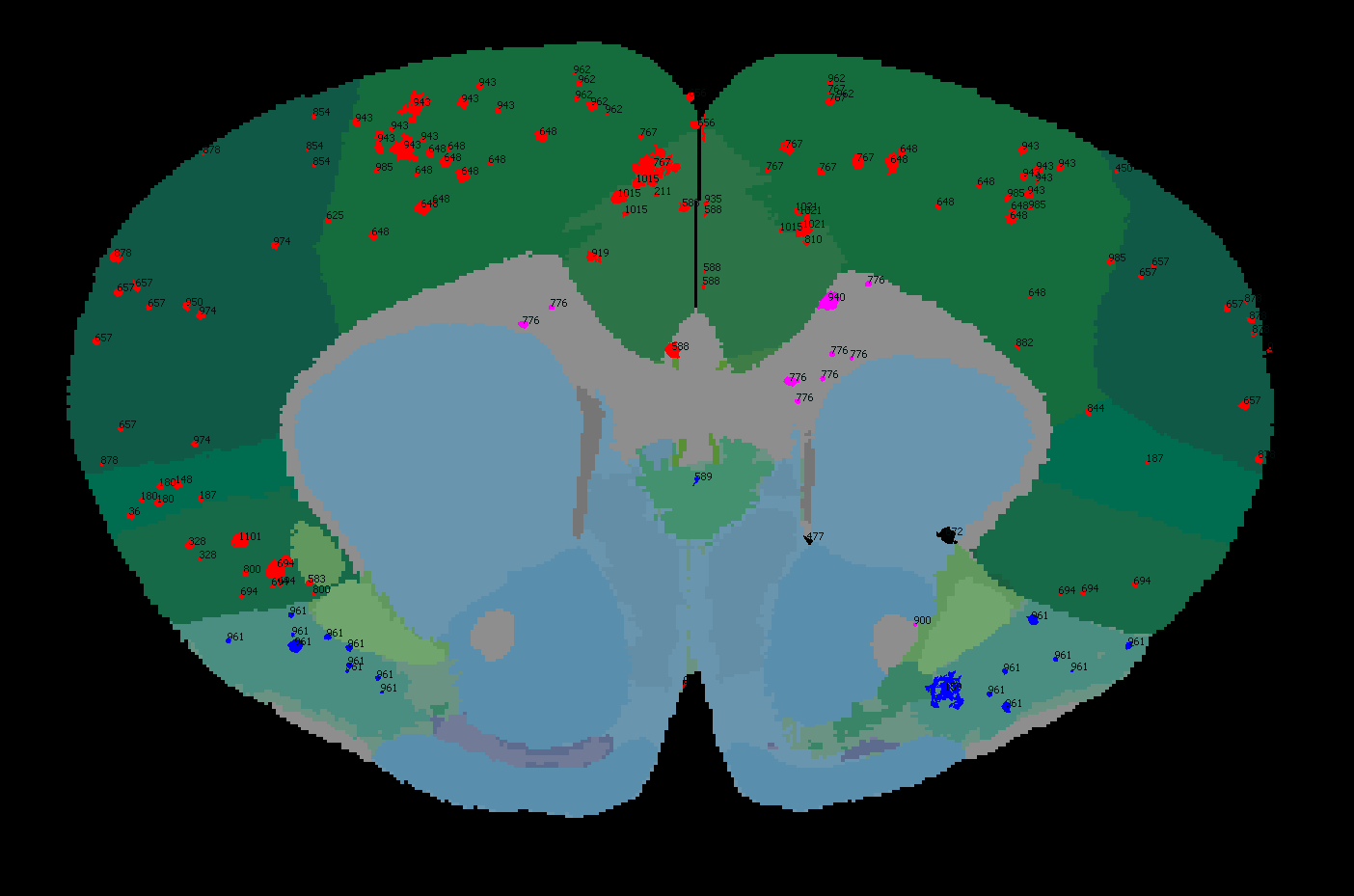

Supplement: Supplementary file 2 [file Data_Sheet_1.ZIP › Supplementary_material_Yates/hAPP/tg2576_m287_1D1_s050_resize_Object Predictions.png]

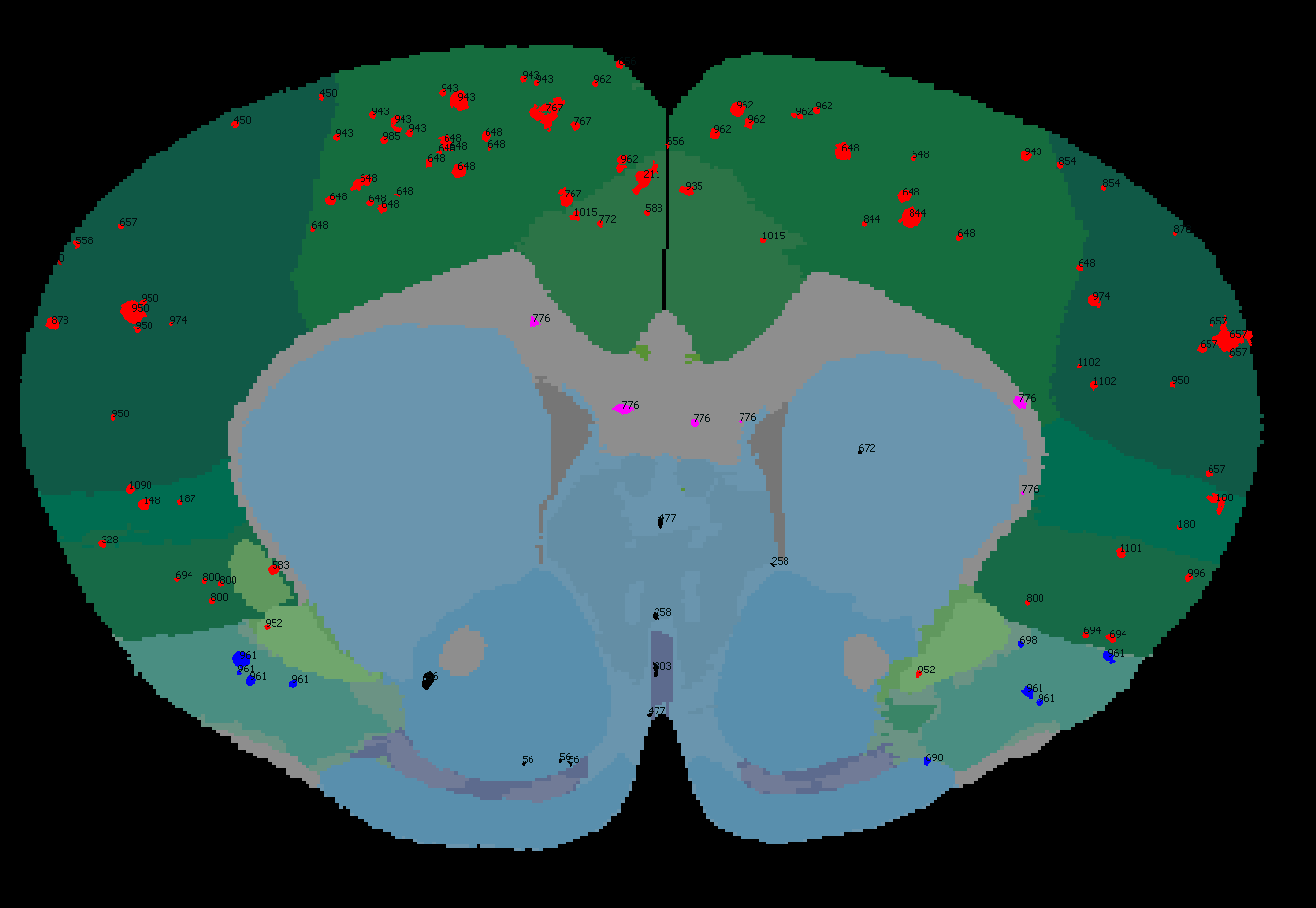

Supplement: Supplementary file 2 [file Data_Sheet_1.ZIP › Supplementary_material_Yates/hAPP/tg2576_m287_1D1_s054_resize_Object Predictions.png]

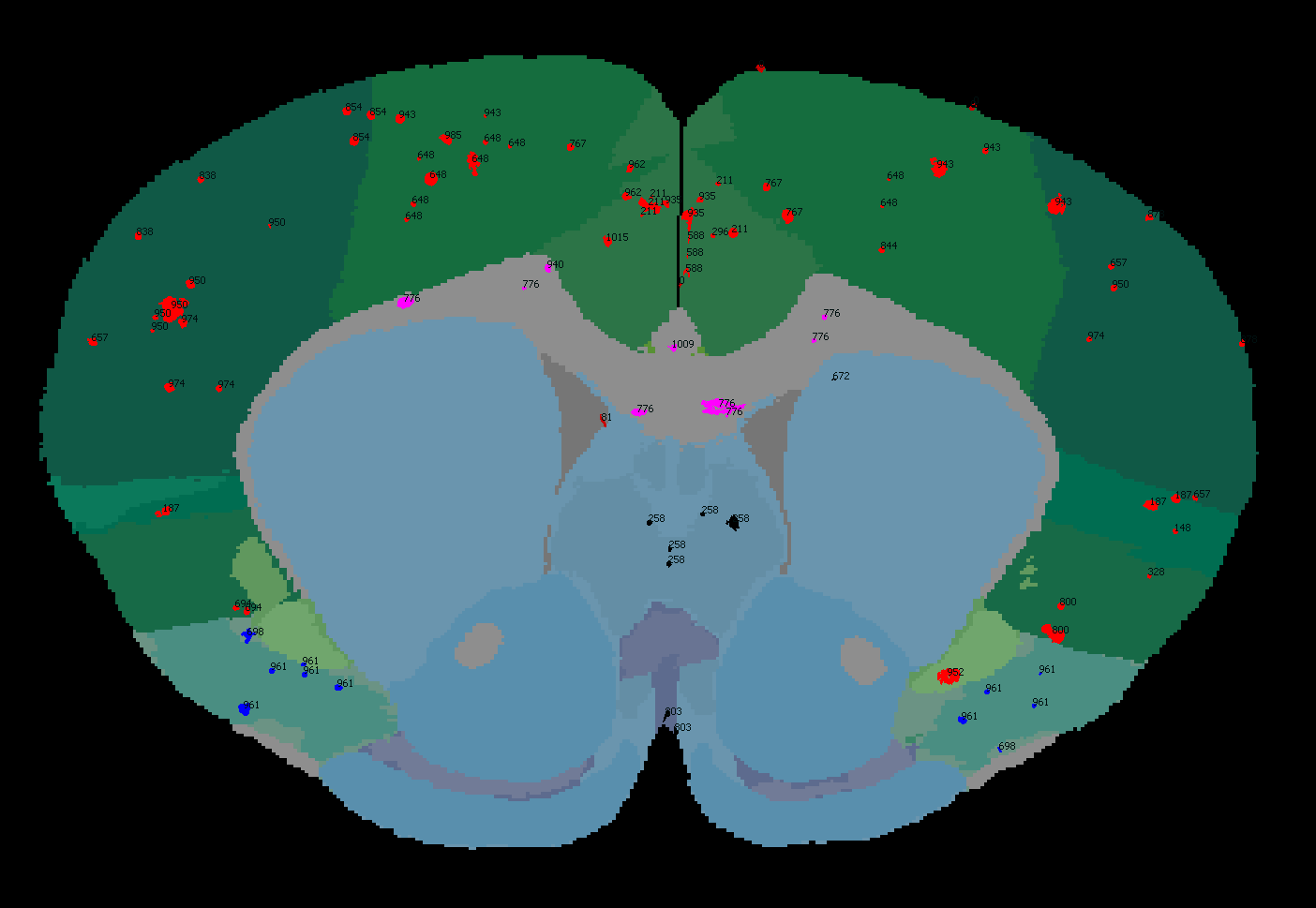

Supplement: Supplementary file 2 [file Data_Sheet_1.ZIP › Supplementary_material_Yates/hAPP/tg2576_m287_1D1_s058_resize_Object Predictions.png]

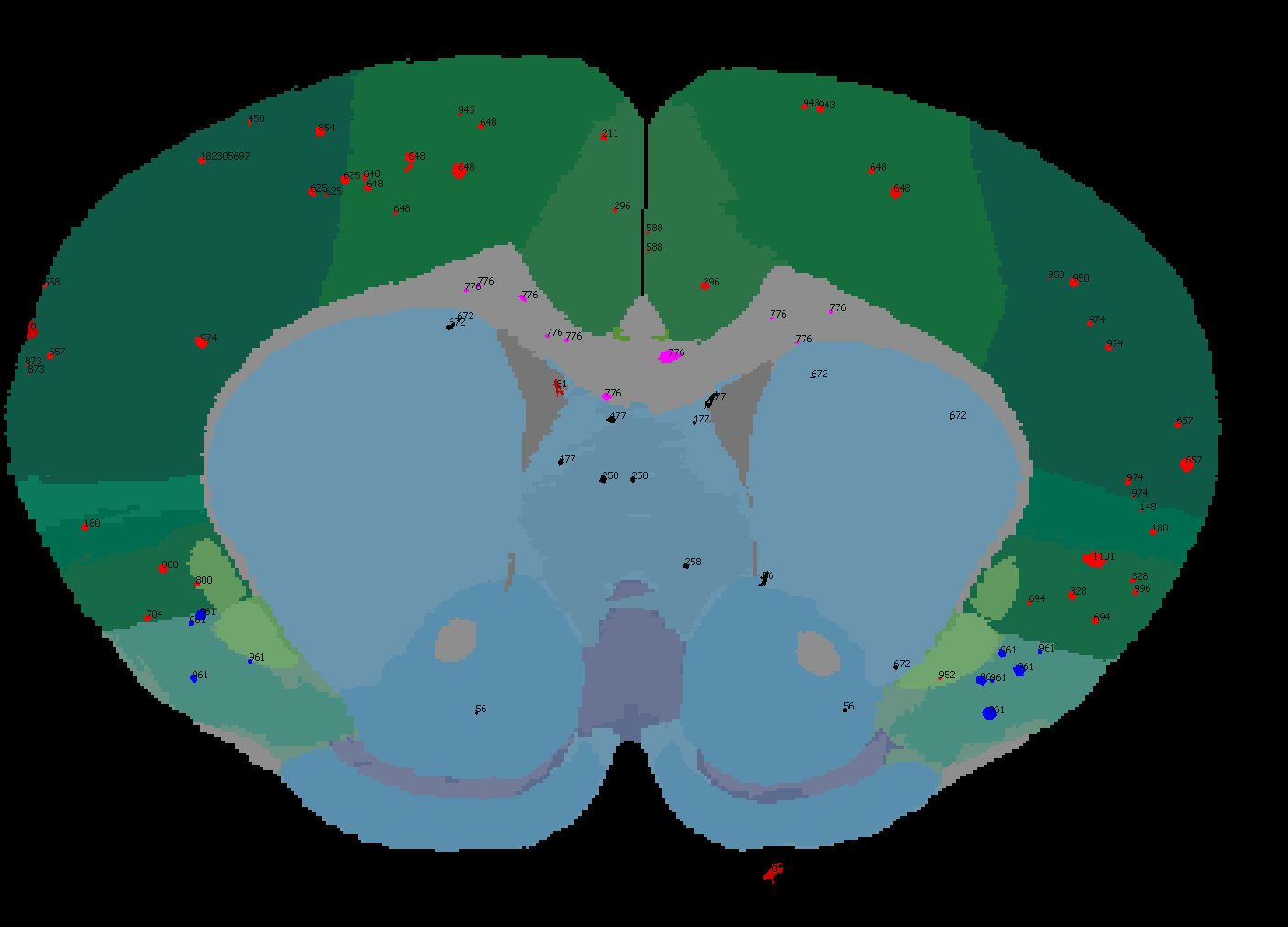

Supplement: Supplementary file 2 [file Data_Sheet_1.ZIP › Supplementary_material_Yates/hAPP/tg2576_m287_1D1_s062_resize_Object Predictions.png]

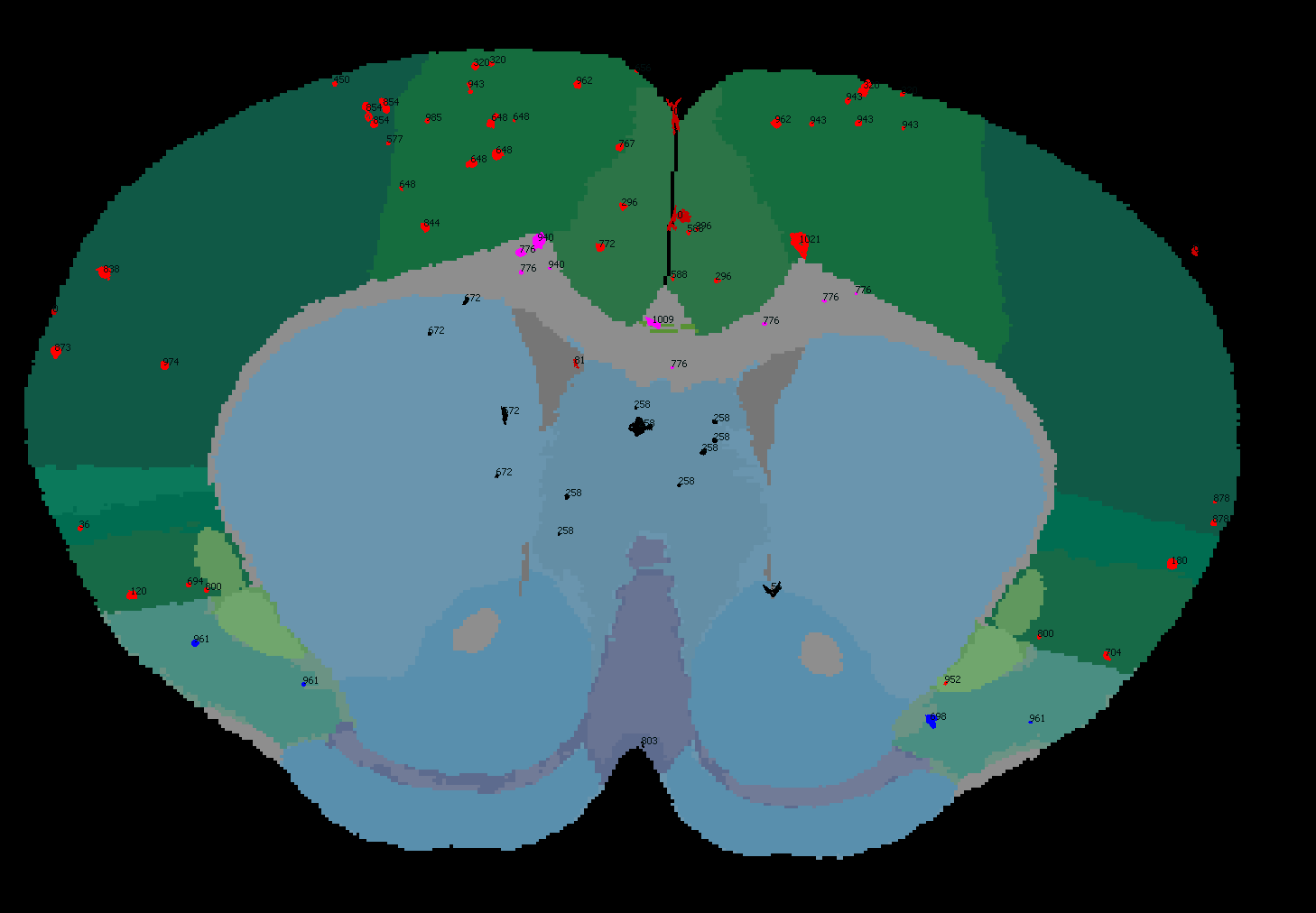

Supplement: Supplementary file 2 [file Data_Sheet_1.ZIP › Supplementary_material_Yates/hAPP/tg2576_m287_1D1_s066_resize_Object Predictions.png]

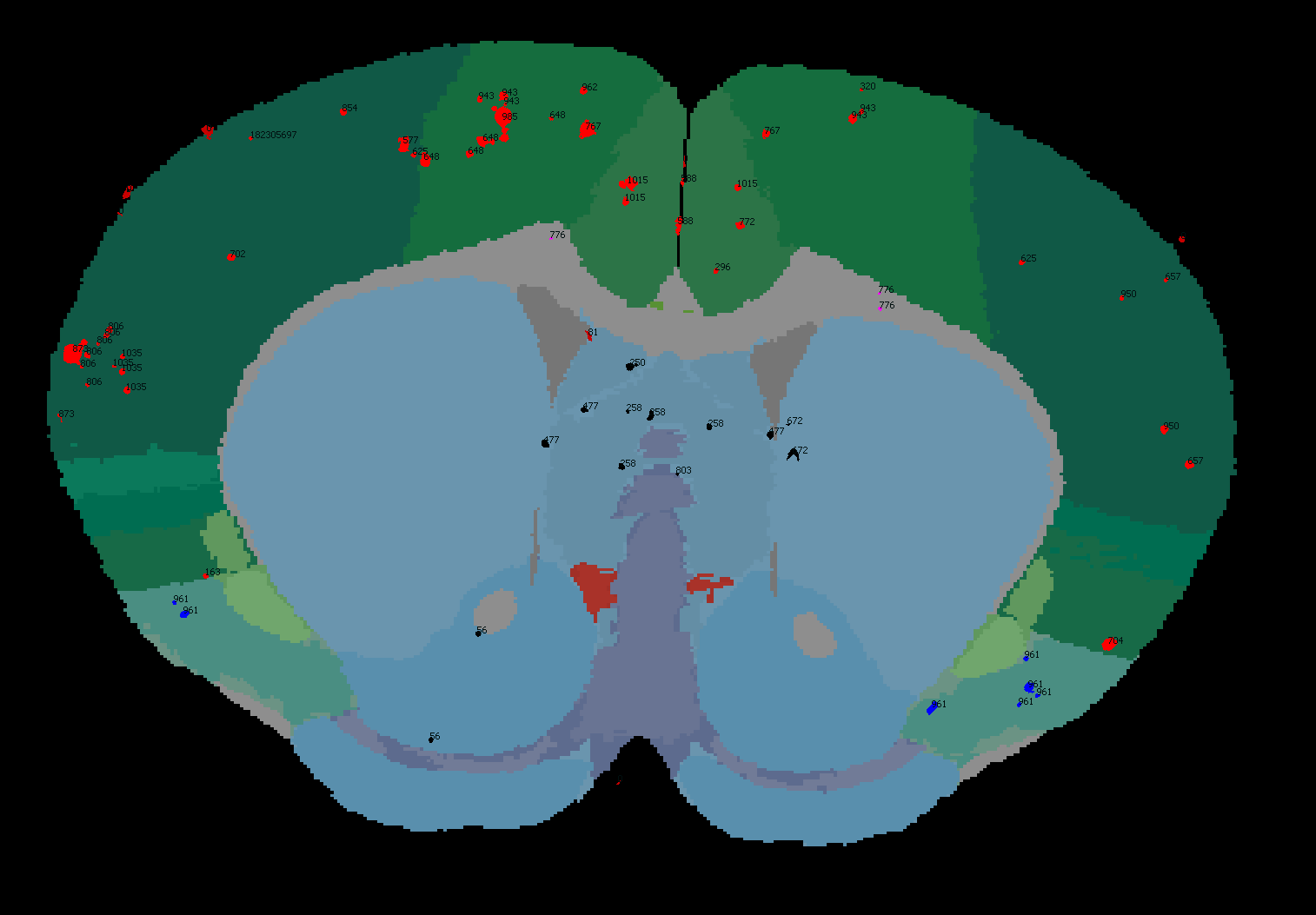

Supplement: Supplementary file 2 [file Data_Sheet_1.ZIP › Supplementary_material_Yates/hAPP/tg2576_m287_1D1_s070_resize_Object Predictions.png]

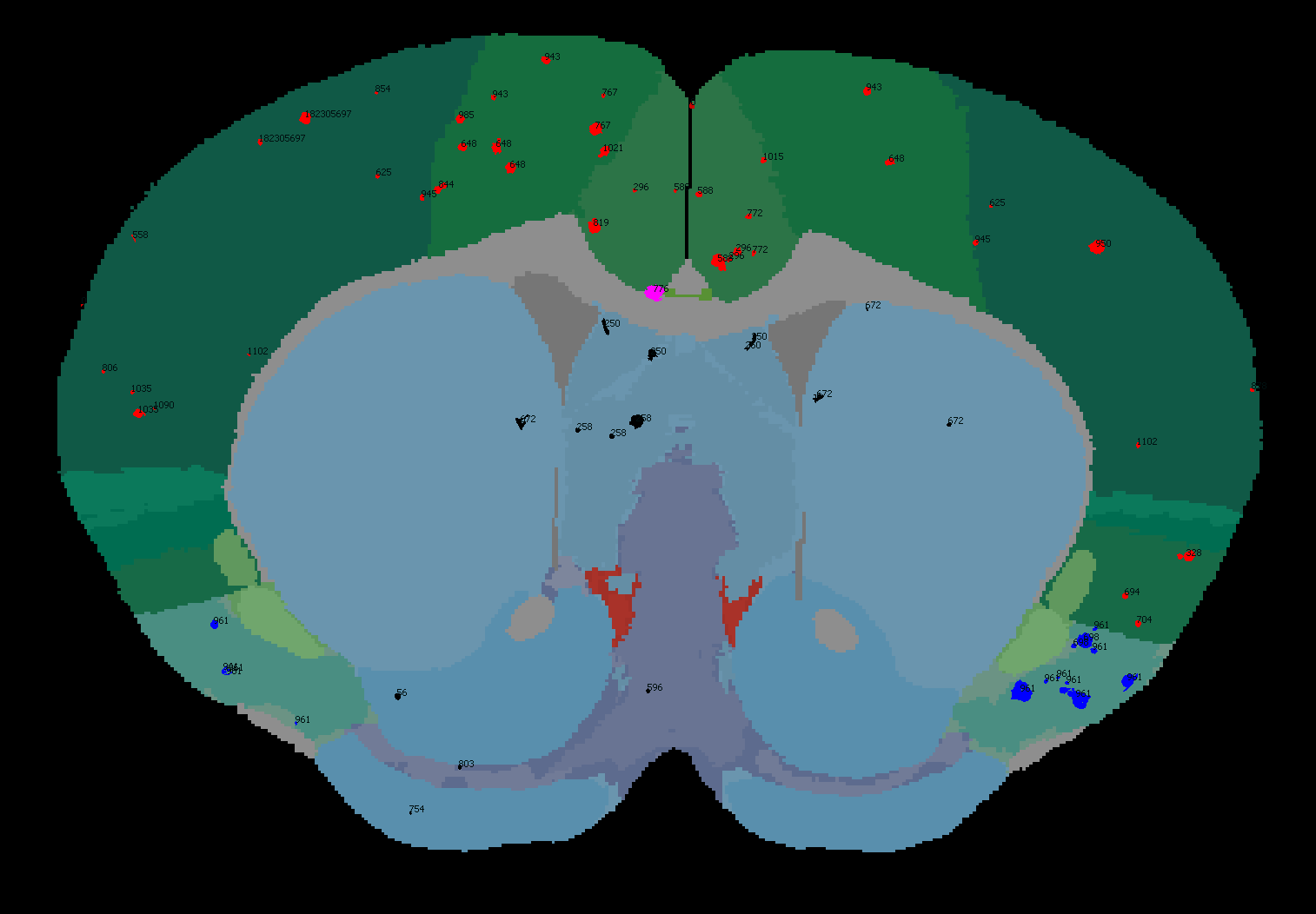

Supplement: Supplementary file 2 [file Data_Sheet_1.ZIP › Supplementary_material_Yates/hAPP/tg2576_m287_1D1_s074_resize_Object Predictions.png]

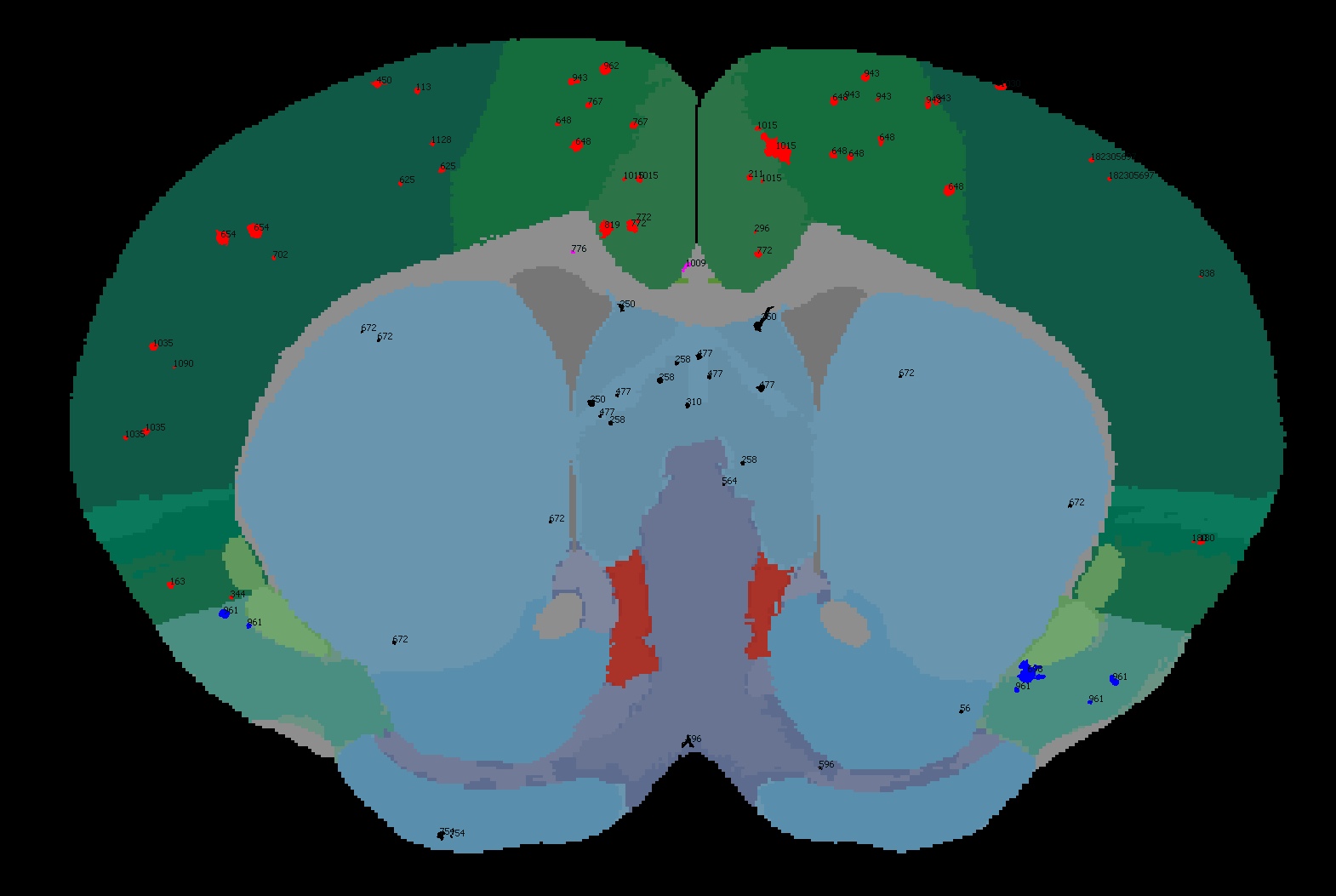

Supplement: Supplementary file 2 [file Data_Sheet_1.ZIP › Supplementary_material_Yates/hAPP/tg2576_m287_1D1_s078_resize_Object Predictions.png]

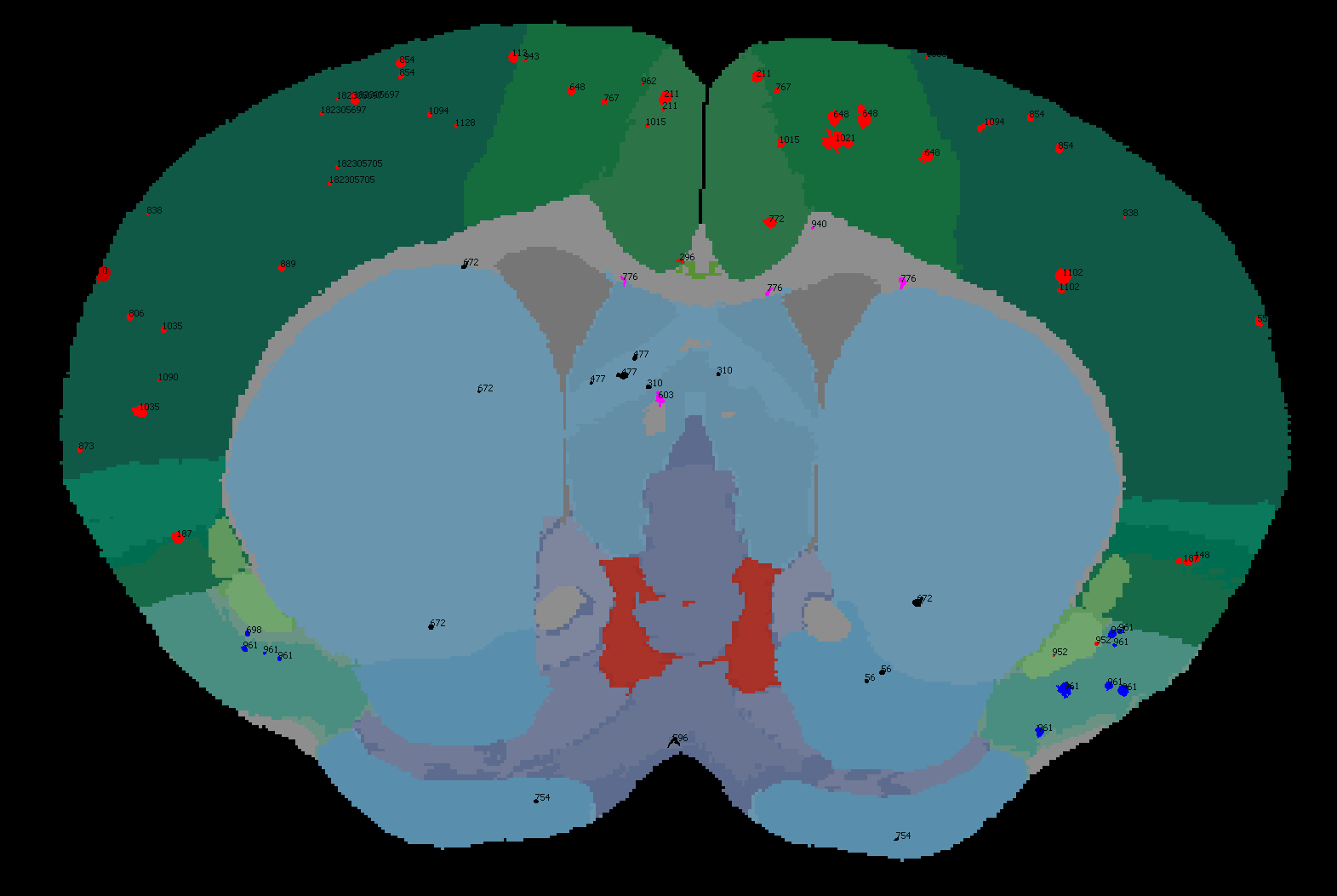

Supplement: Supplementary file 2 [file Data_Sheet_1.ZIP › Supplementary_material_Yates/hAPP/tg2576_m287_1D1_s082_resize_Object Predictions.png]

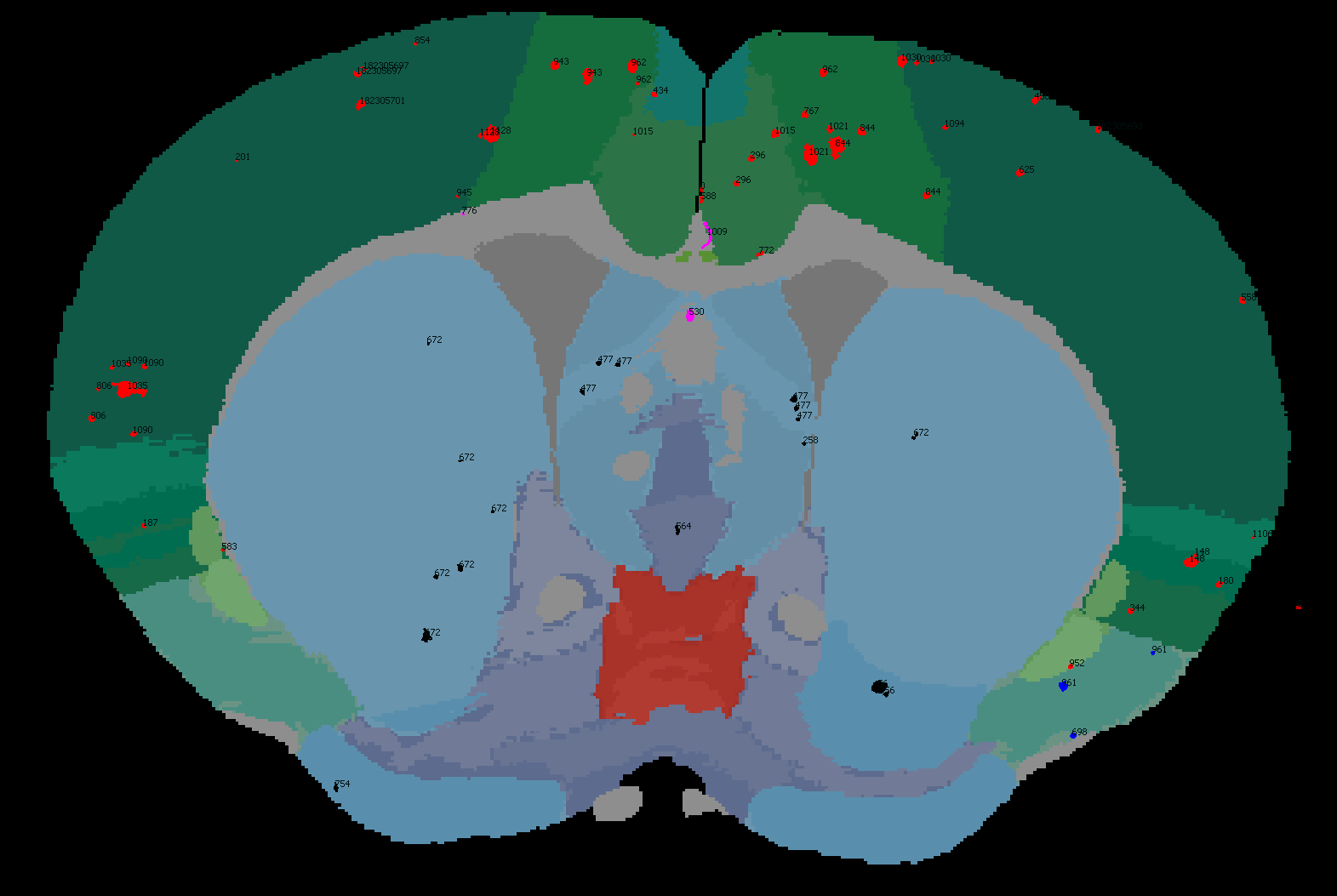

Supplement: Supplementary file 2 [file Data_Sheet_1.ZIP › Supplementary_material_Yates/hAPP/tg2576_m287_1D1_s086_resize_Object Predictions.png]

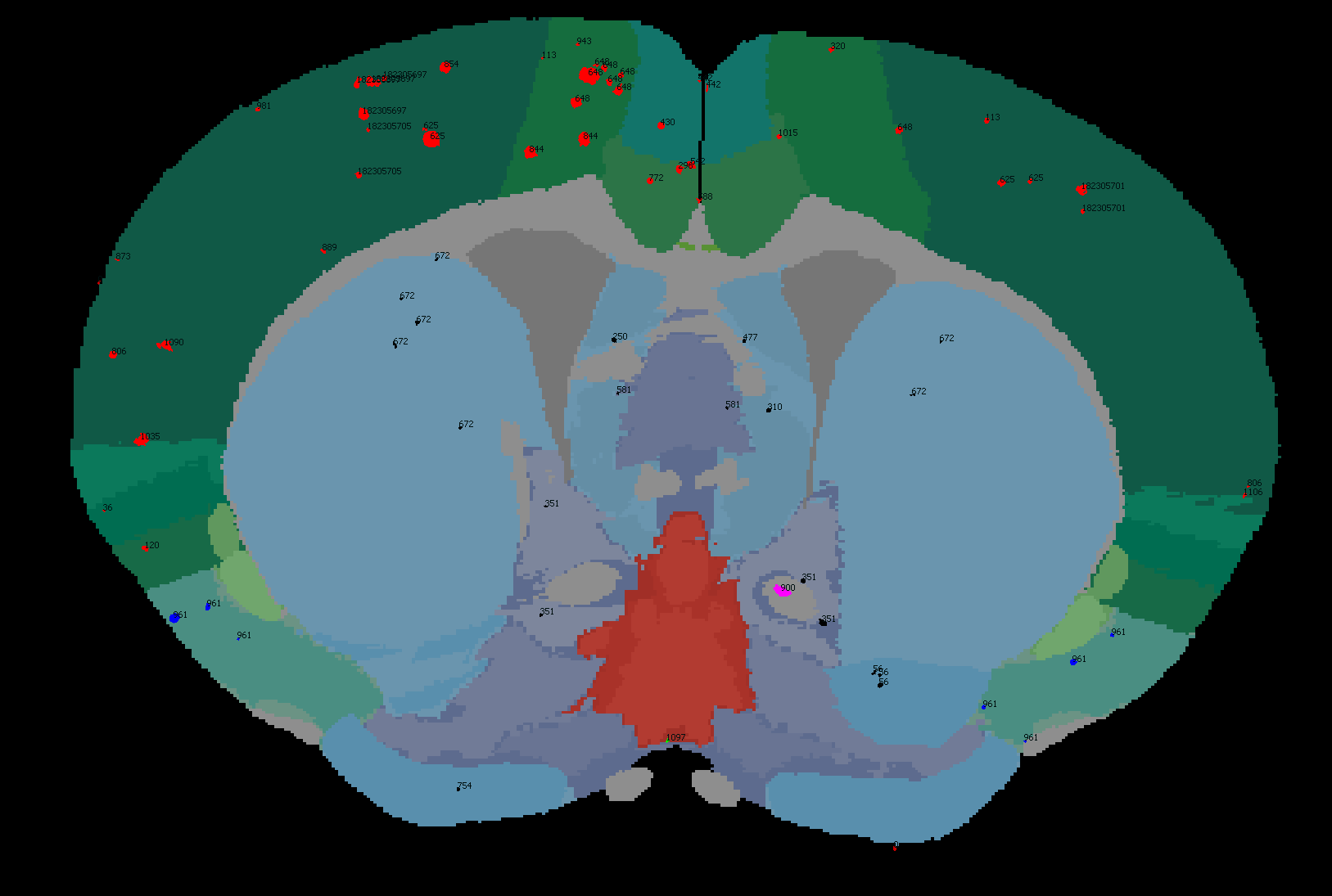

Supplement: Supplementary file 2 [file Data_Sheet_1.ZIP › Supplementary_material_Yates/hAPP/tg2576_m287_1D1_s090_resize_Object Predictions.png]

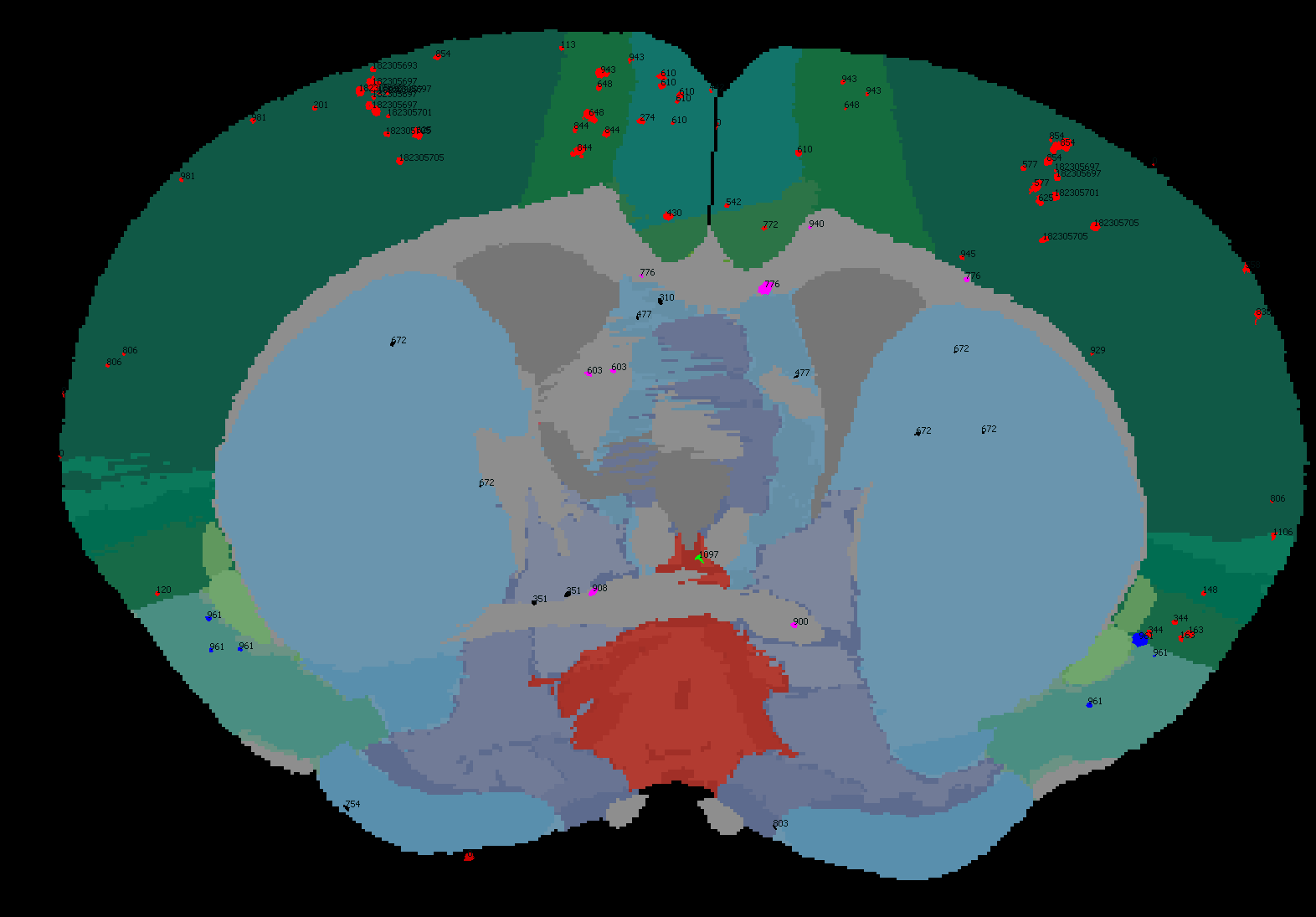

Supplement: Supplementary file 2 [file Data_Sheet_1.ZIP › Supplementary_material_Yates/hAPP/tg2576_m287_1D1_s094_resize_Object Predictions.png]

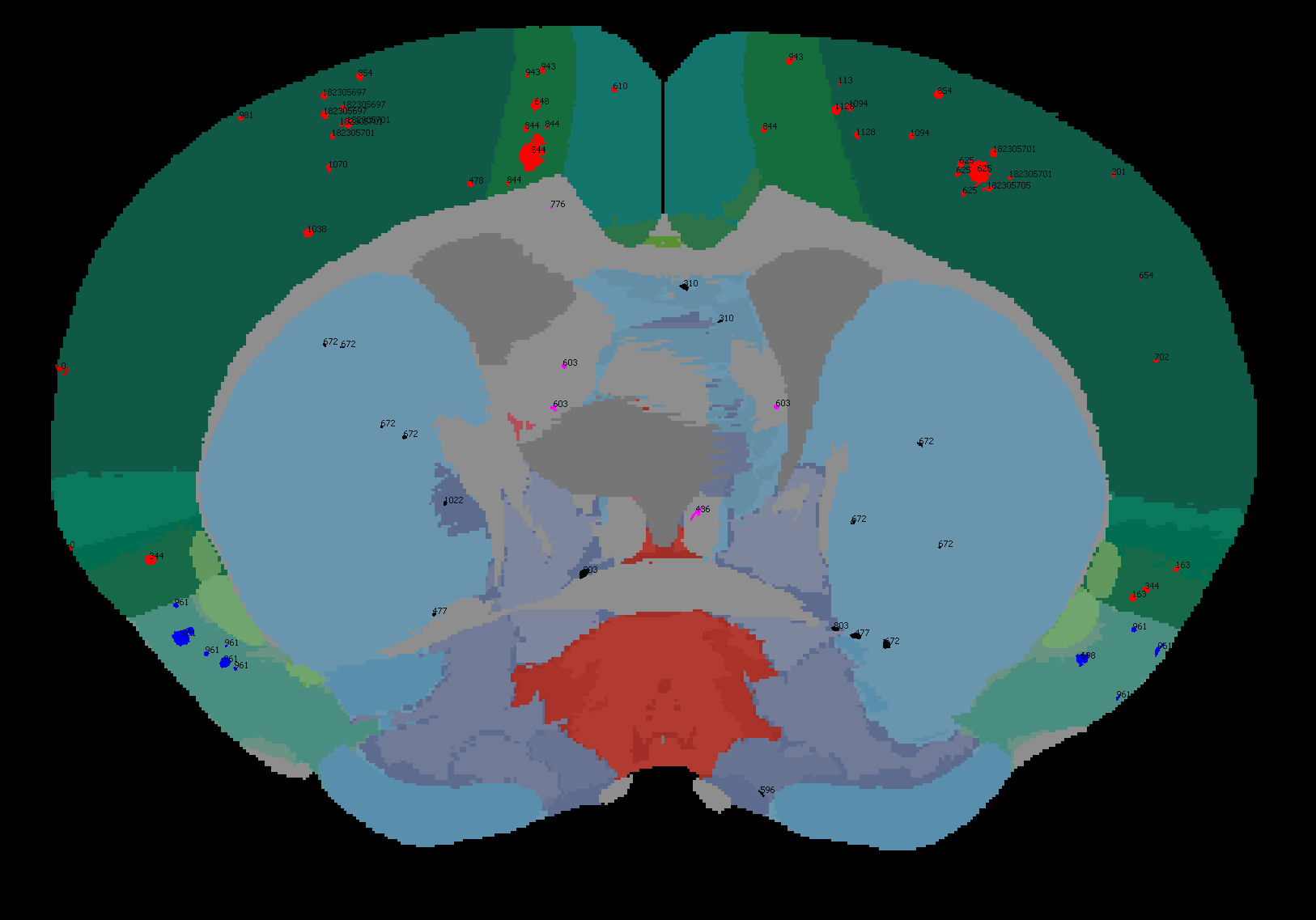

Supplement: Supplementary file 2 [file Data_Sheet_1.ZIP › Supplementary_material_Yates/hAPP/tg2576_m287_1D1_s098_resize_Object Predictions.png]

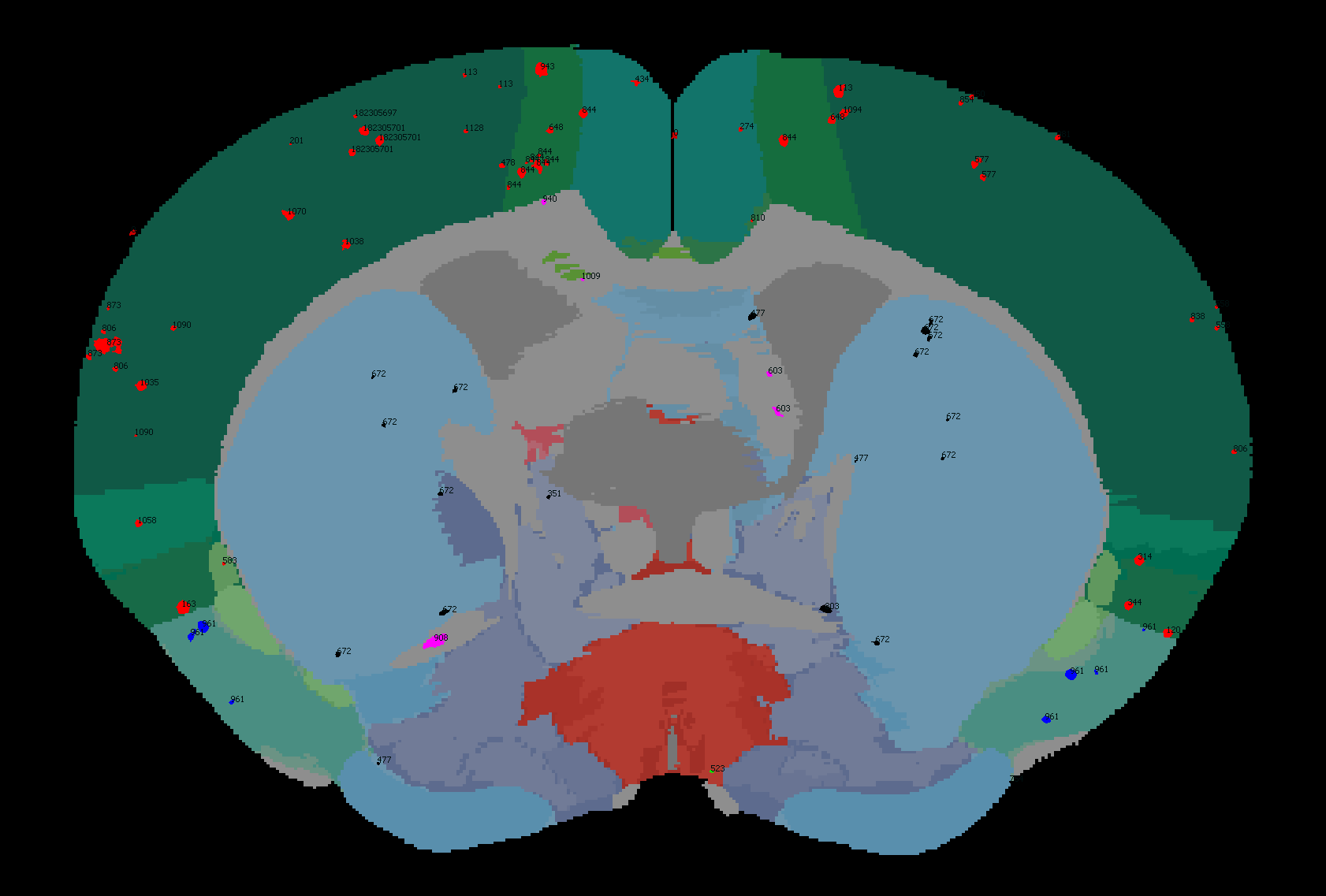

Supplement: Supplementary file 2 [file Data_Sheet_1.ZIP › Supplementary_material_Yates/hAPP/tg2576_m287_1D1_s102_resize_Object Predictions.png]

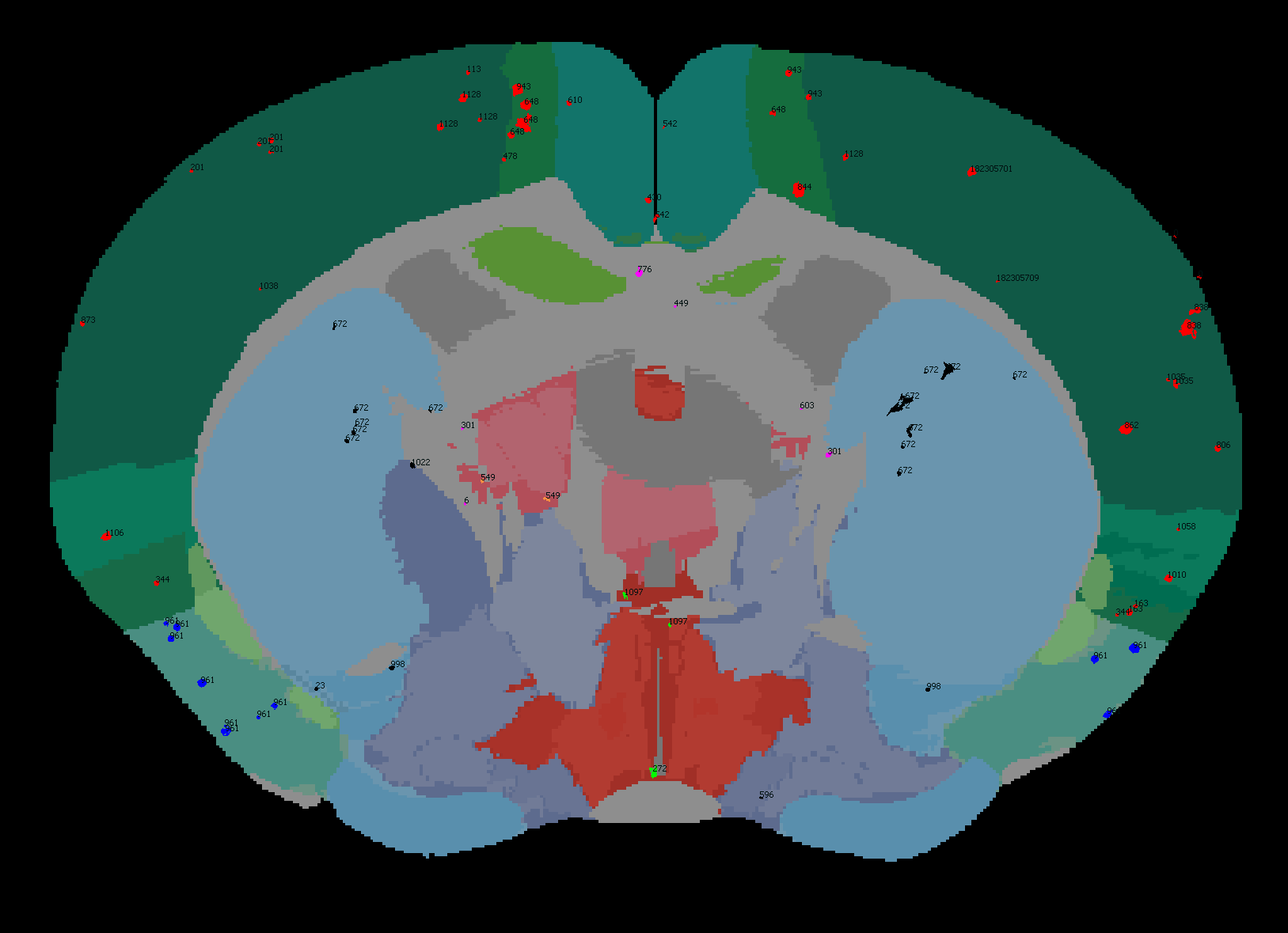

Supplement: Supplementary file 2 [file Data_Sheet_1.ZIP › Supplementary_material_Yates/hAPP/tg2576_m287_1D1_s106_resize_Object Predictions.png]

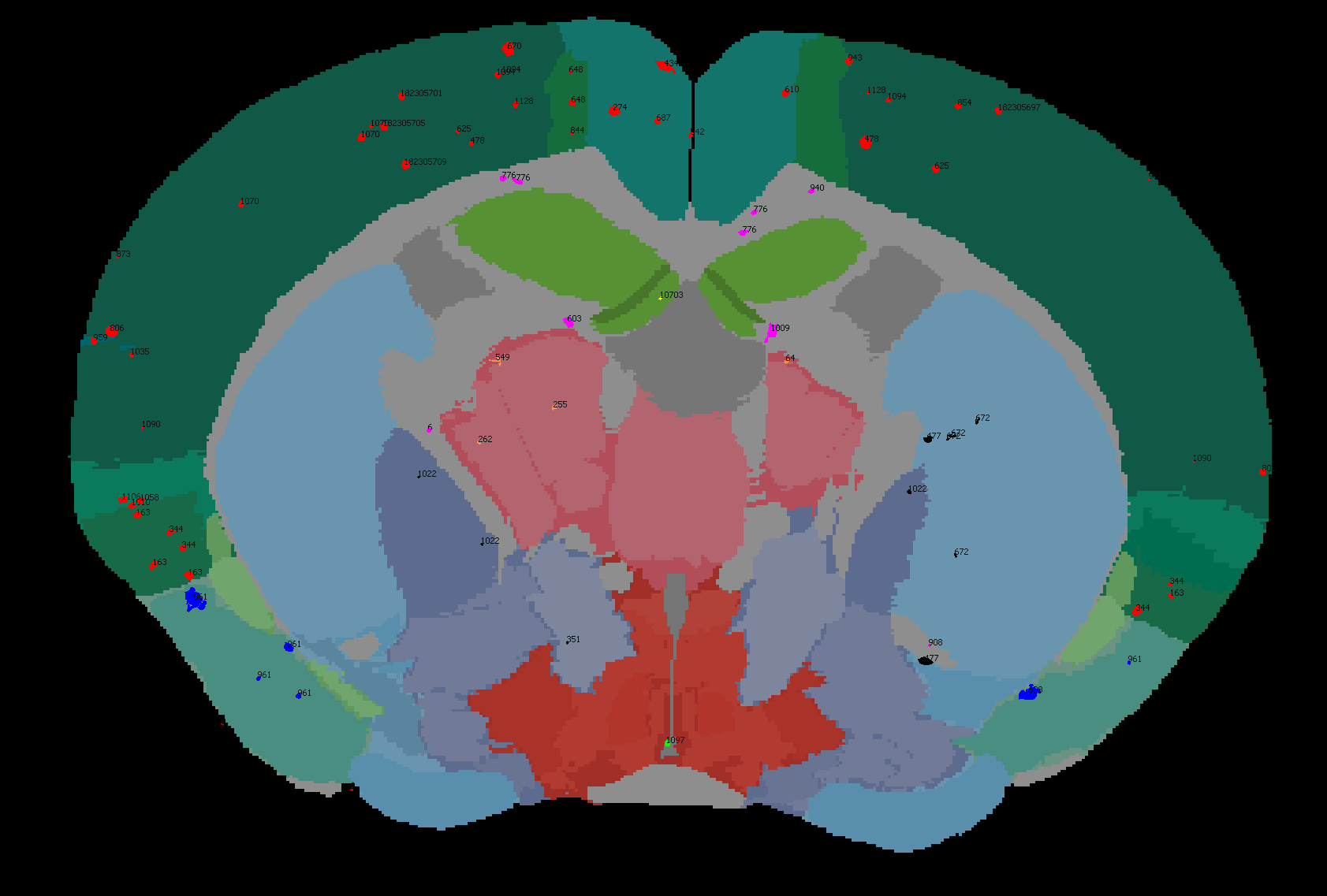

Supplement: Supplementary file 2 [file Data_Sheet_1.ZIP › Supplementary_material_Yates/hAPP/tg2576_m287_1D1_s110_resize_Object Predictions.png]

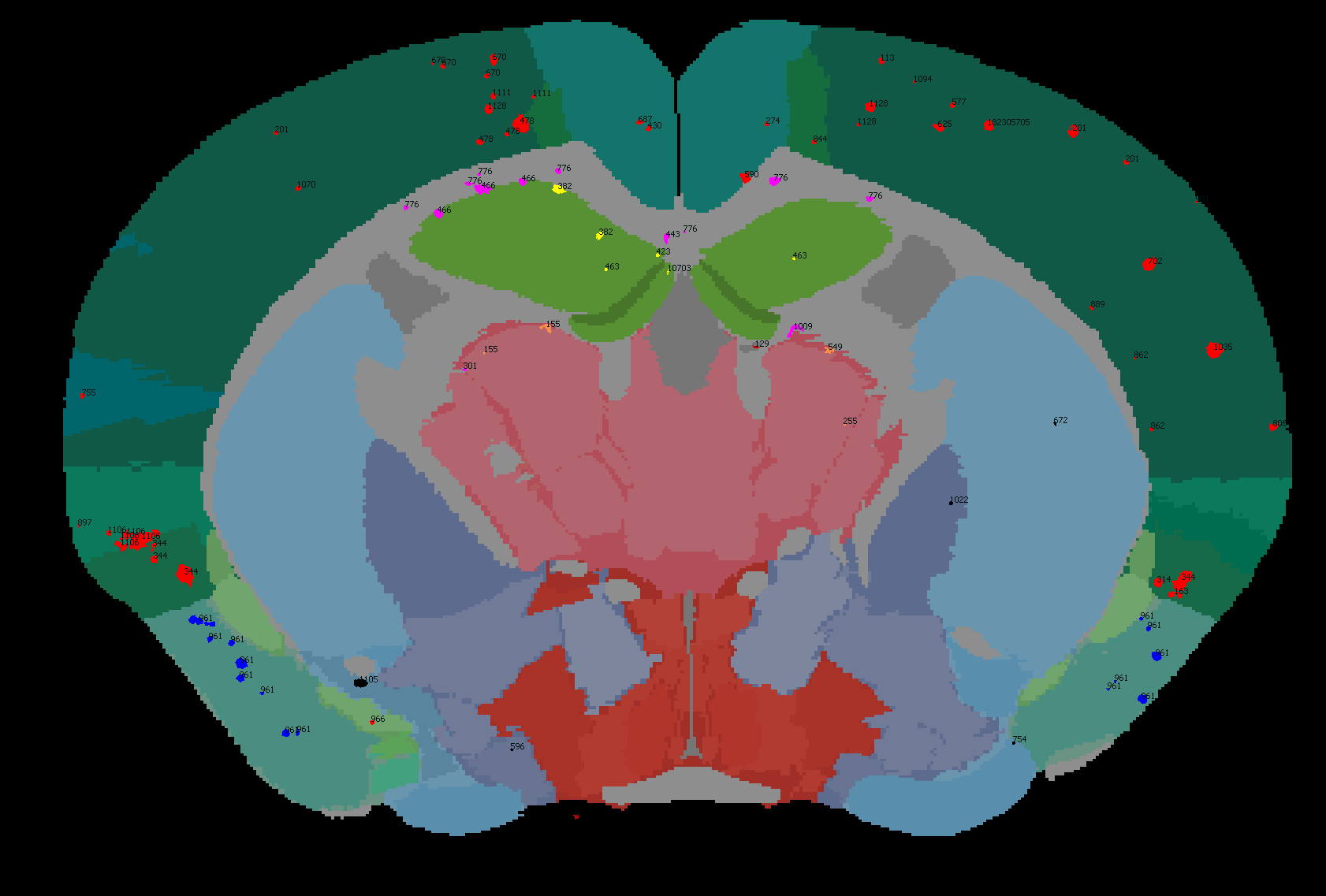

Supplement: Supplementary file 2 [file Data_Sheet_1.ZIP › Supplementary_material_Yates/hAPP/tg2576_m287_1D1_s114_resize_Object Predictions.png]

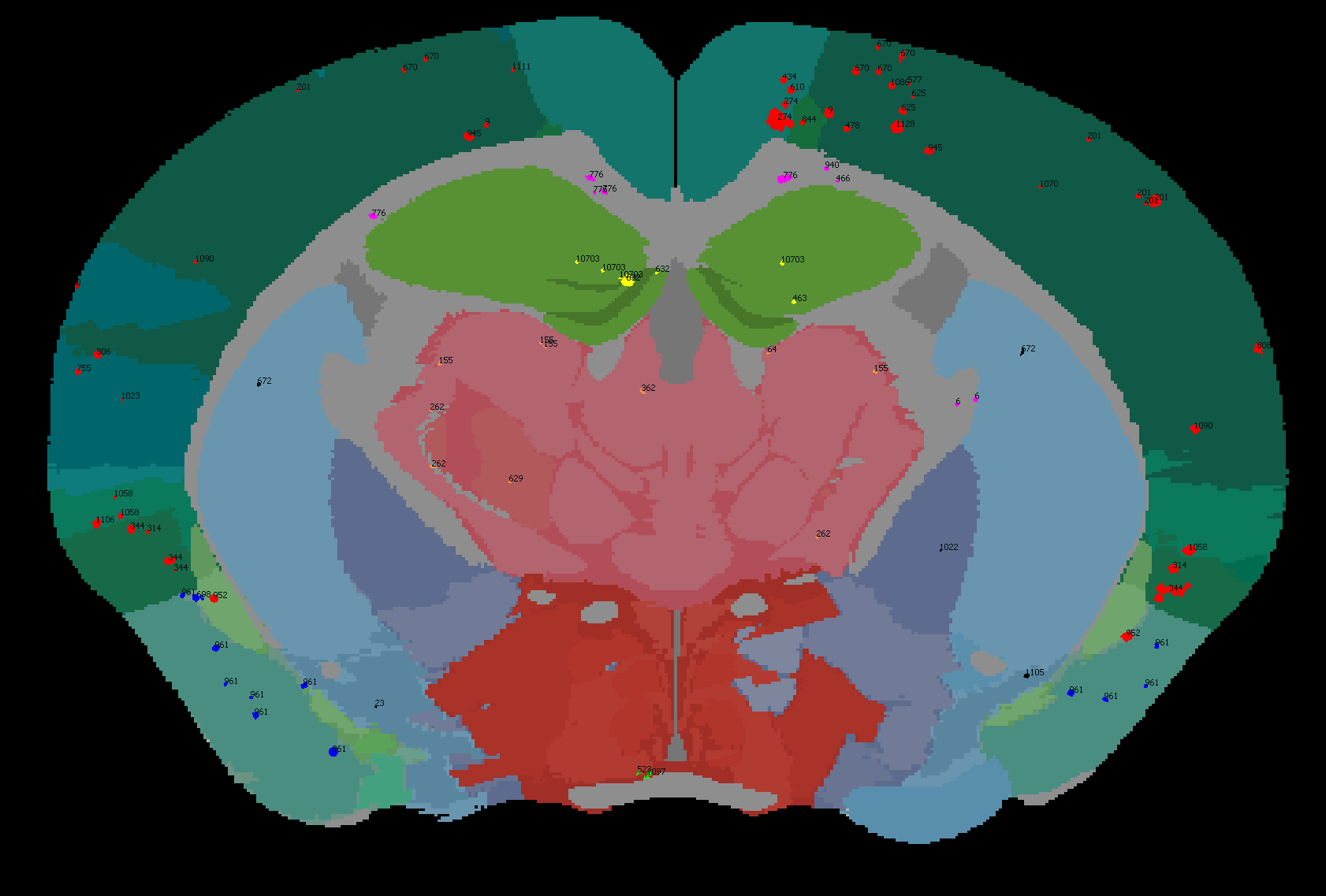

Supplement: Supplementary file 2 [file Data_Sheet_1.ZIP › Supplementary_material_Yates/hAPP/tg2576_m287_1D1_s118_resize_Object Predictions.png]

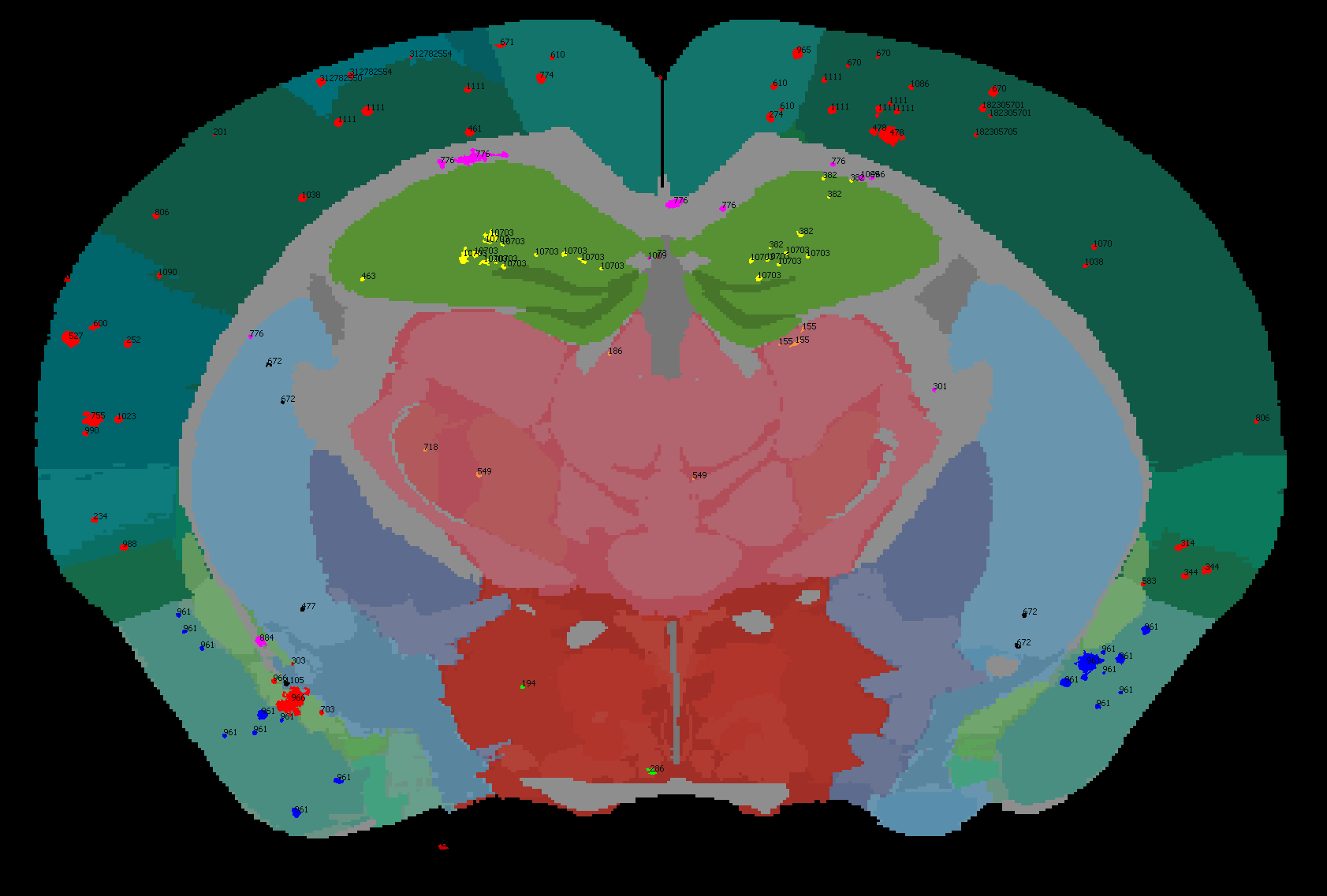

Supplement: Supplementary file 2 [file Data_Sheet_1.ZIP › Supplementary_material_Yates/hAPP/tg2576_m287_1D1_s122_resize_Object Predictions.png]

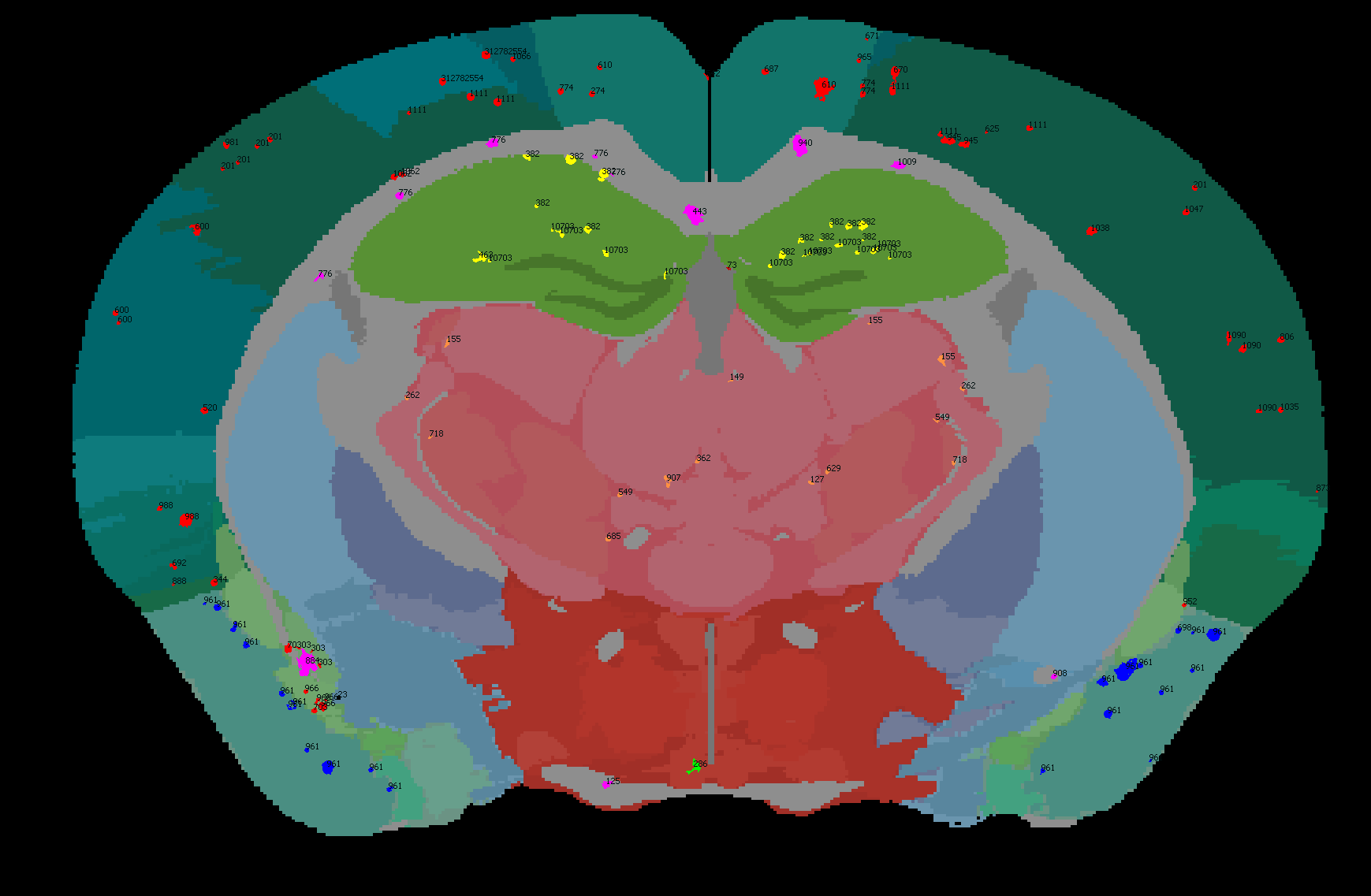

Supplement: Supplementary file 2 [file Data_Sheet_1.ZIP › Supplementary_material_Yates/hAPP/tg2576_m287_1D1_s126_resize_Object Predictions.png]

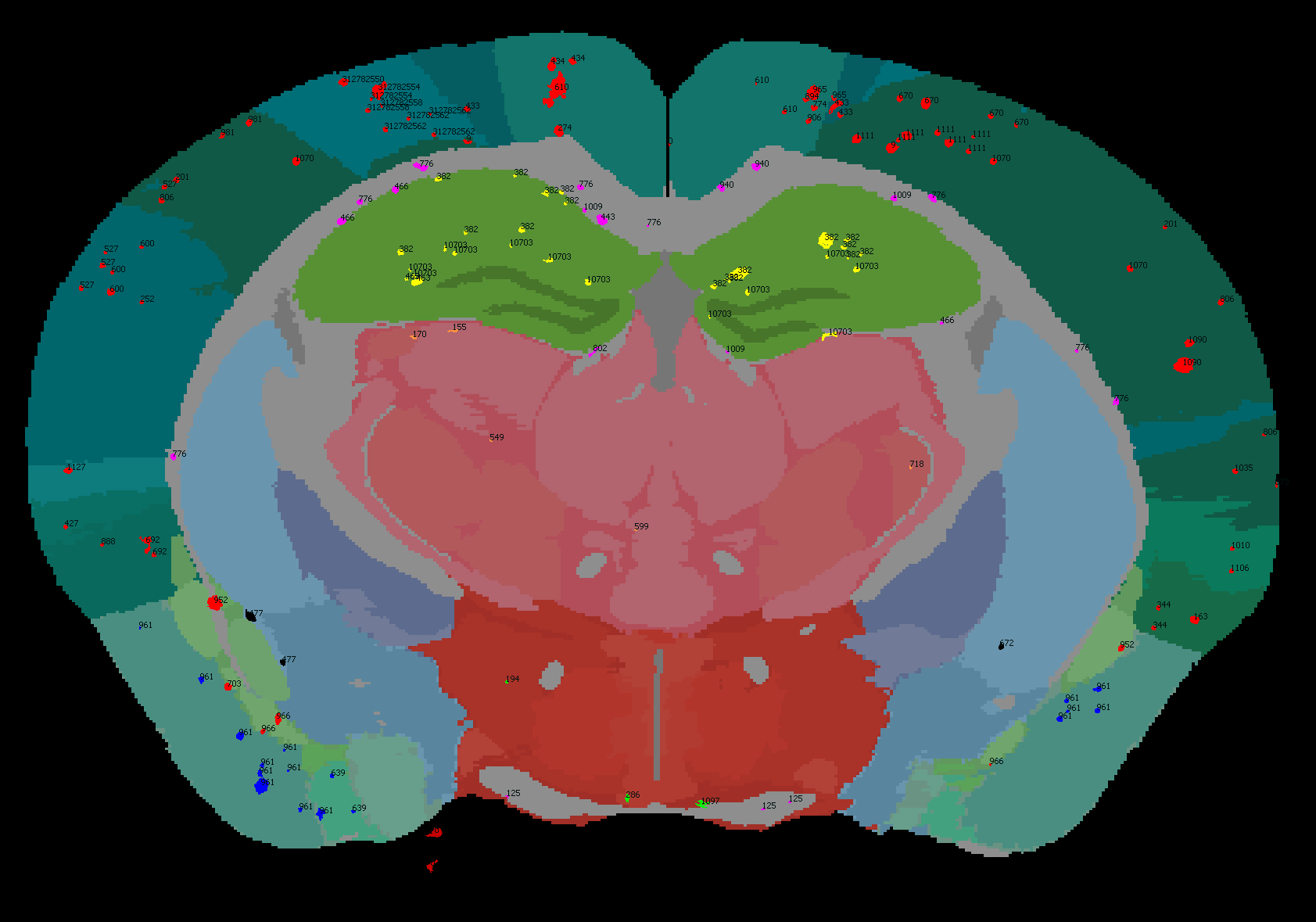

Supplement: Supplementary file 2 [file Data_Sheet_1.ZIP › Supplementary_material_Yates/hAPP/tg2576_m287_1D1_s130_resize_Object Predictions.png]

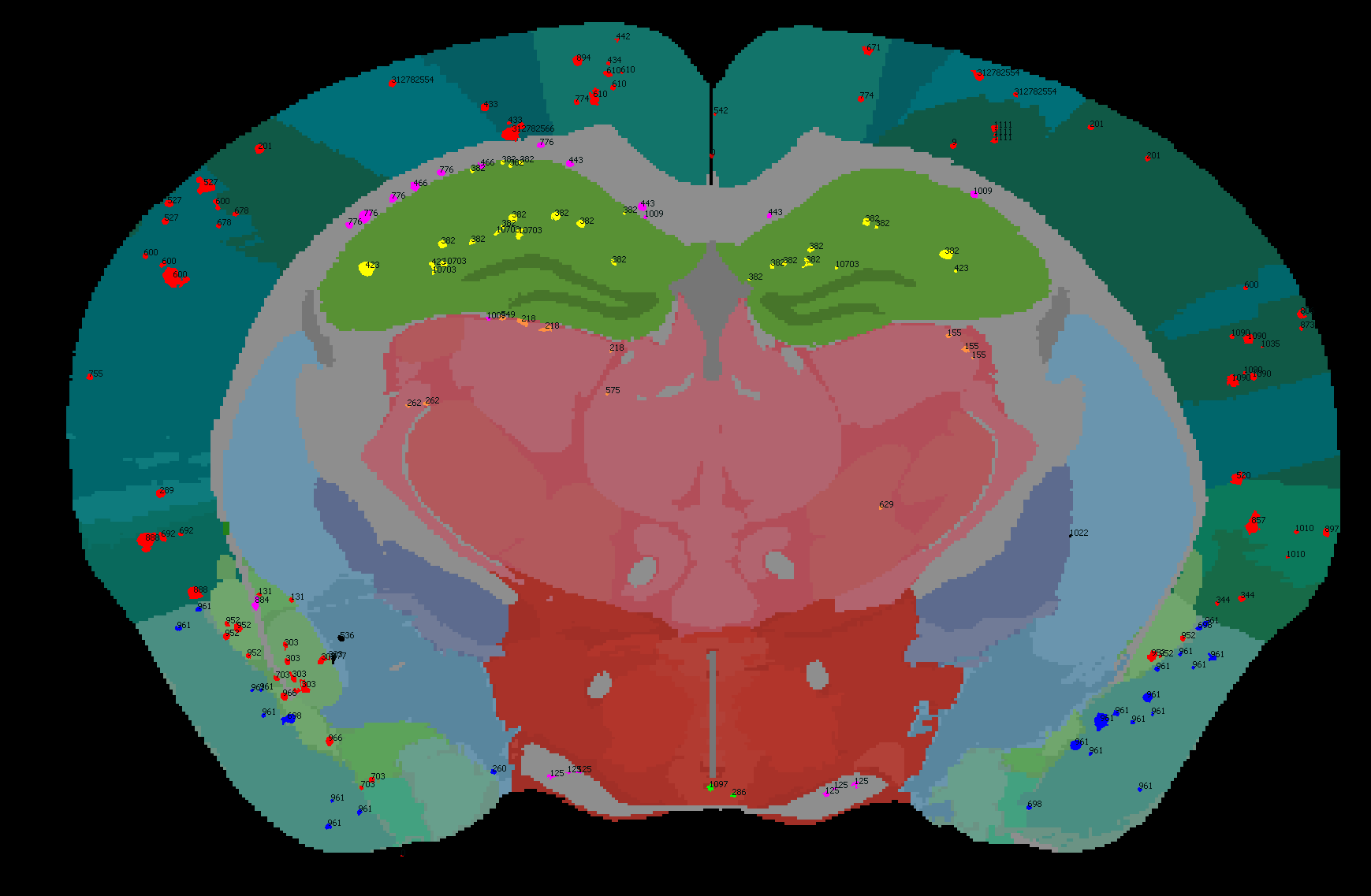

Supplement: Supplementary file 2 [file Data_Sheet_1.ZIP › Supplementary_material_Yates/hAPP/tg2576_m287_1D1_s134_resize_Object Predictions.png]

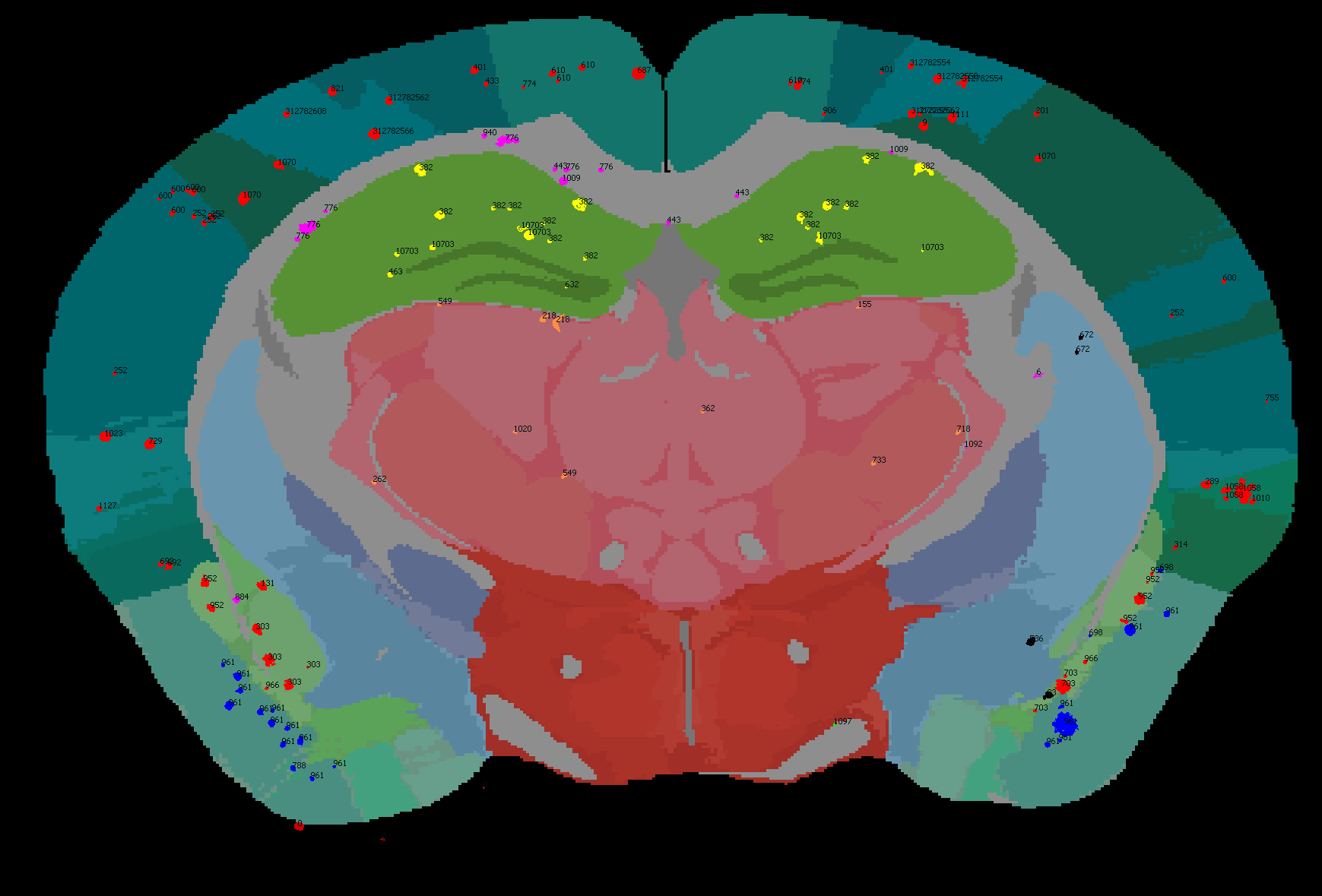

Supplement: Supplementary file 2 [file Data_Sheet_1.ZIP › Supplementary_material_Yates/hAPP/tg2576_m287_1D1_s138_resize_Object Predictions.png]

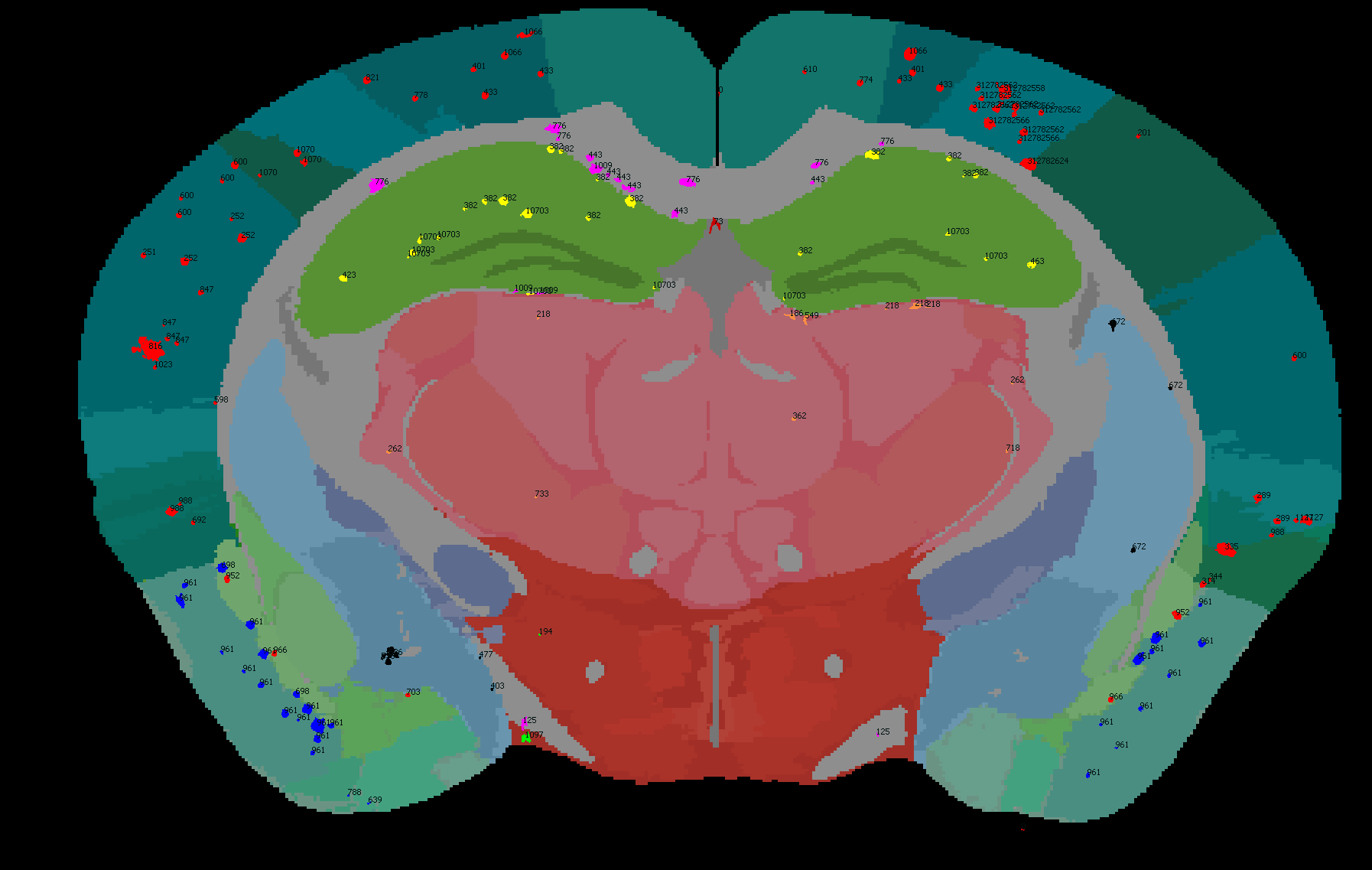

Supplement: Supplementary file 2 [file Data_Sheet_1.ZIP › Supplementary_material_Yates/hAPP/tg2576_m287_1D1_s142_resize_Object Predictions.png]

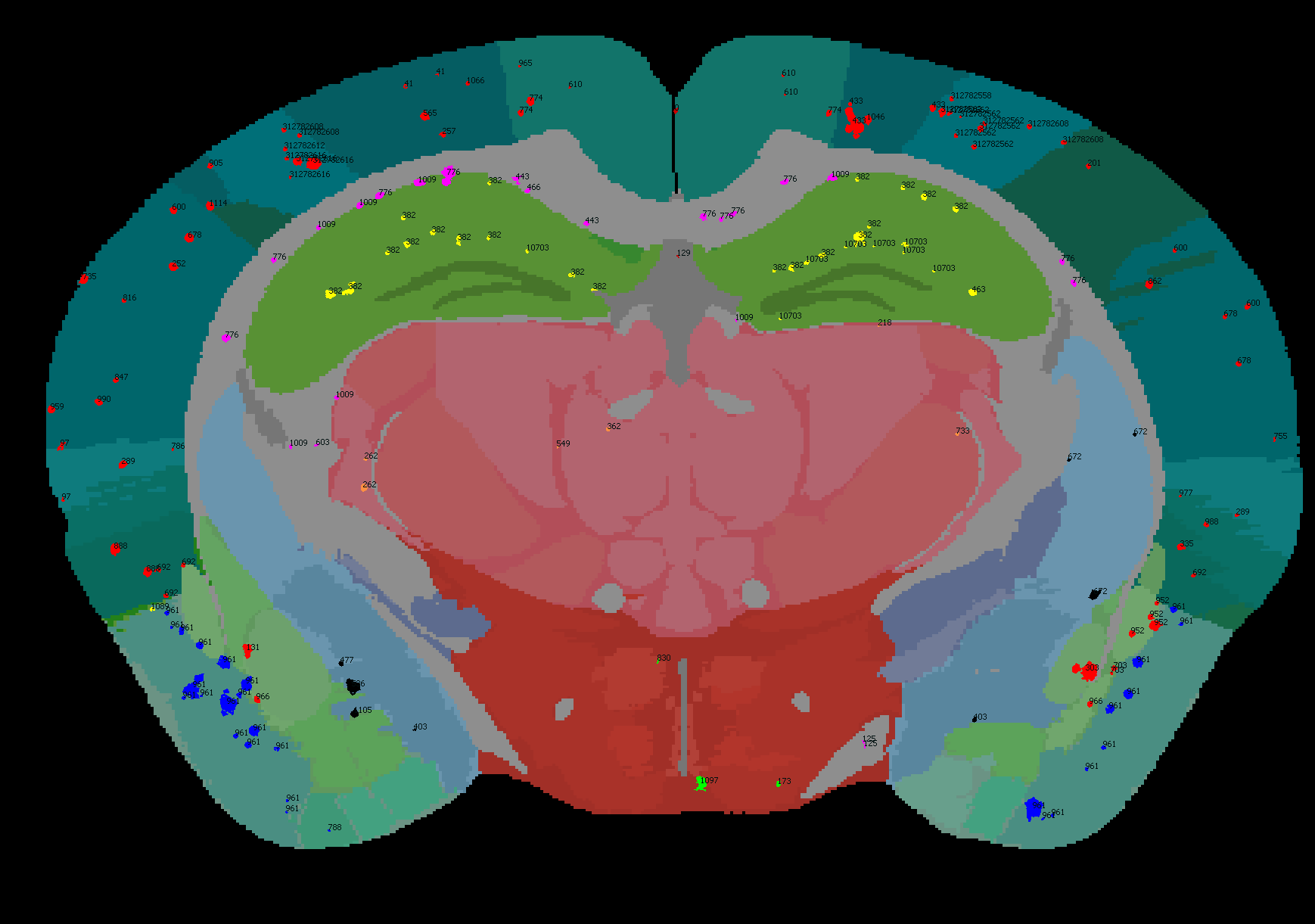

Supplement: Supplementary file 2 [file Data_Sheet_1.ZIP › Supplementary_material_Yates/hAPP/tg2576_m287_1D1_s146_resize_Object Predictions.png]

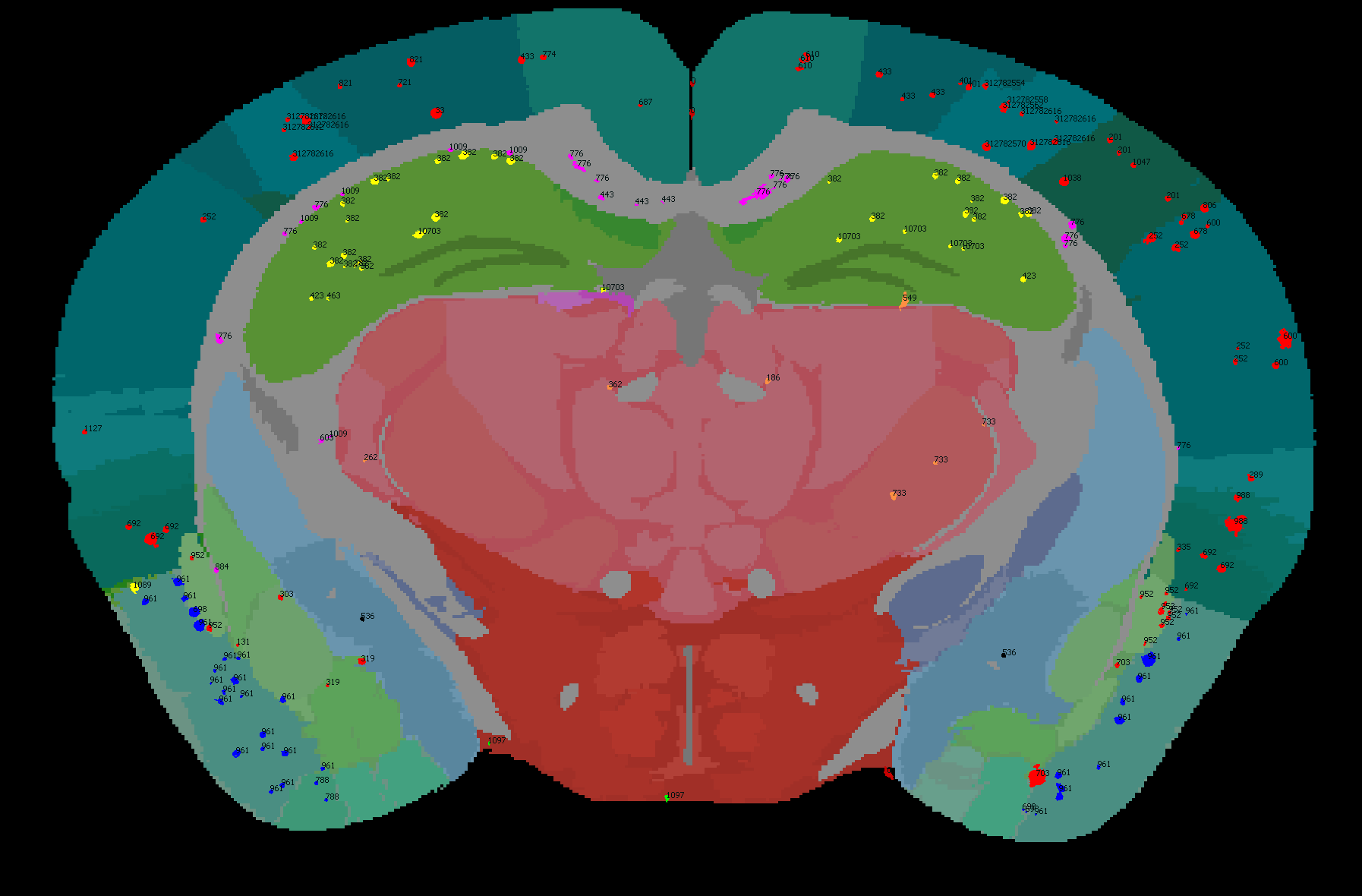

Supplement: Supplementary file 2 [file Data_Sheet_1.ZIP › Supplementary_material_Yates/hAPP/tg2576_m287_1D1_s150_resize_Object Predictions.png]

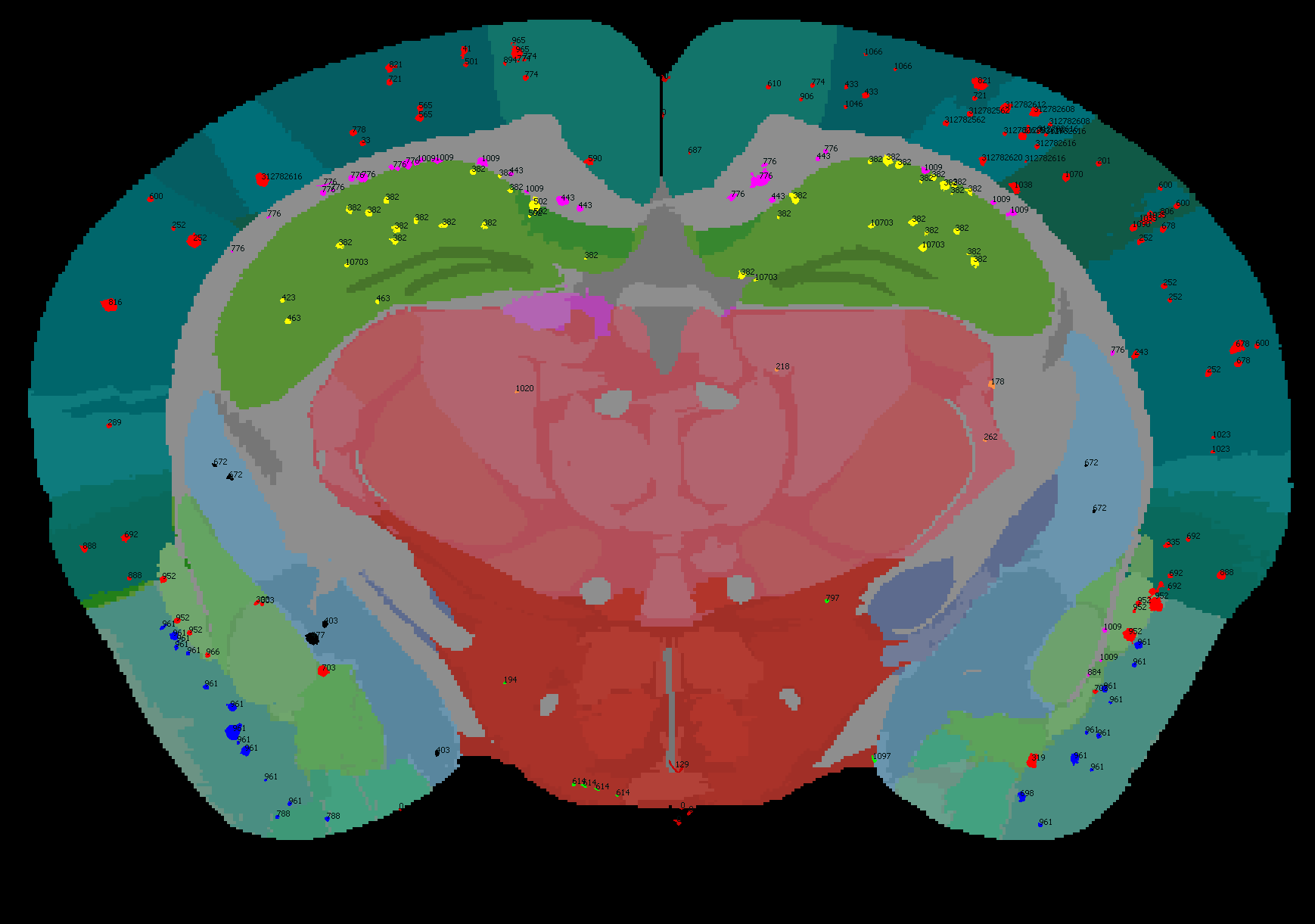

Supplement: Supplementary file 2 [file Data_Sheet_1.ZIP › Supplementary_material_Yates/hAPP/tg2576_m287_1D1_s154_resize_Object Predictions.png]

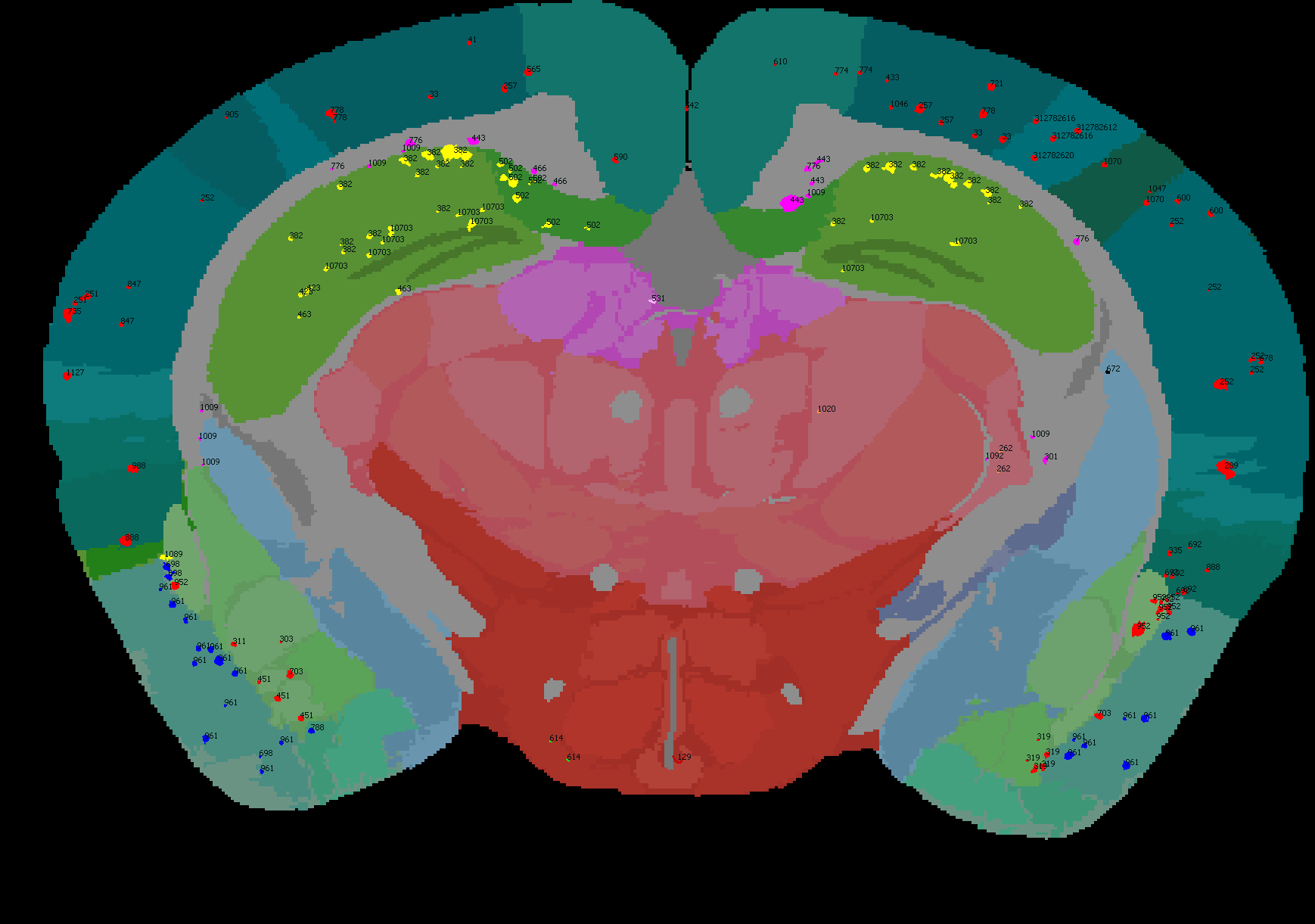

Supplement: Supplementary file 2 [file Data_Sheet_1.ZIP › Supplementary_material_Yates/hAPP/tg2576_m287_1D1_s158_resize_Object Predictions.png]

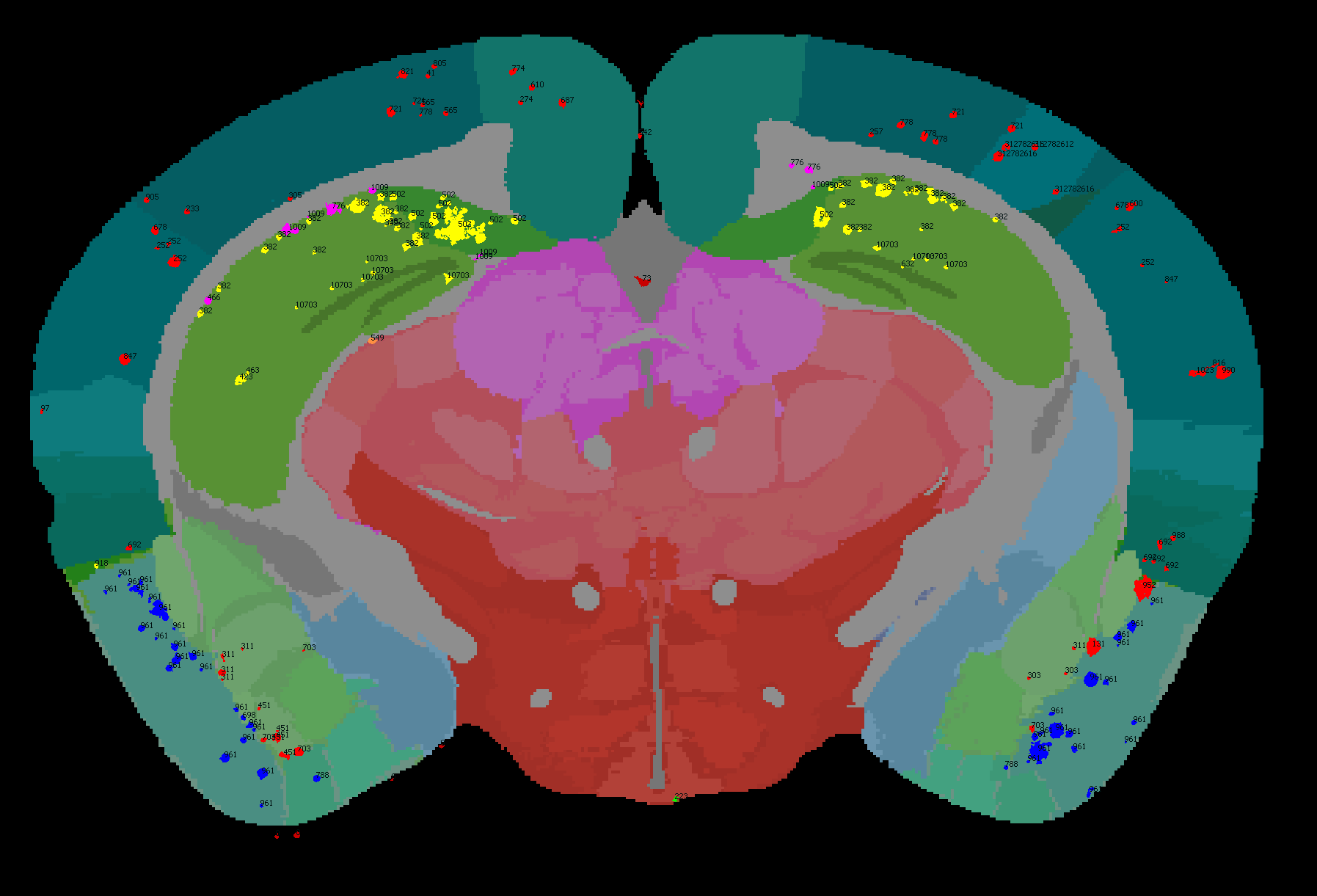

Supplement: Supplementary file 2 [file Data_Sheet_1.ZIP › Supplementary_material_Yates/hAPP/tg2576_m287_1D1_s166_resize_Object Predictions.png]

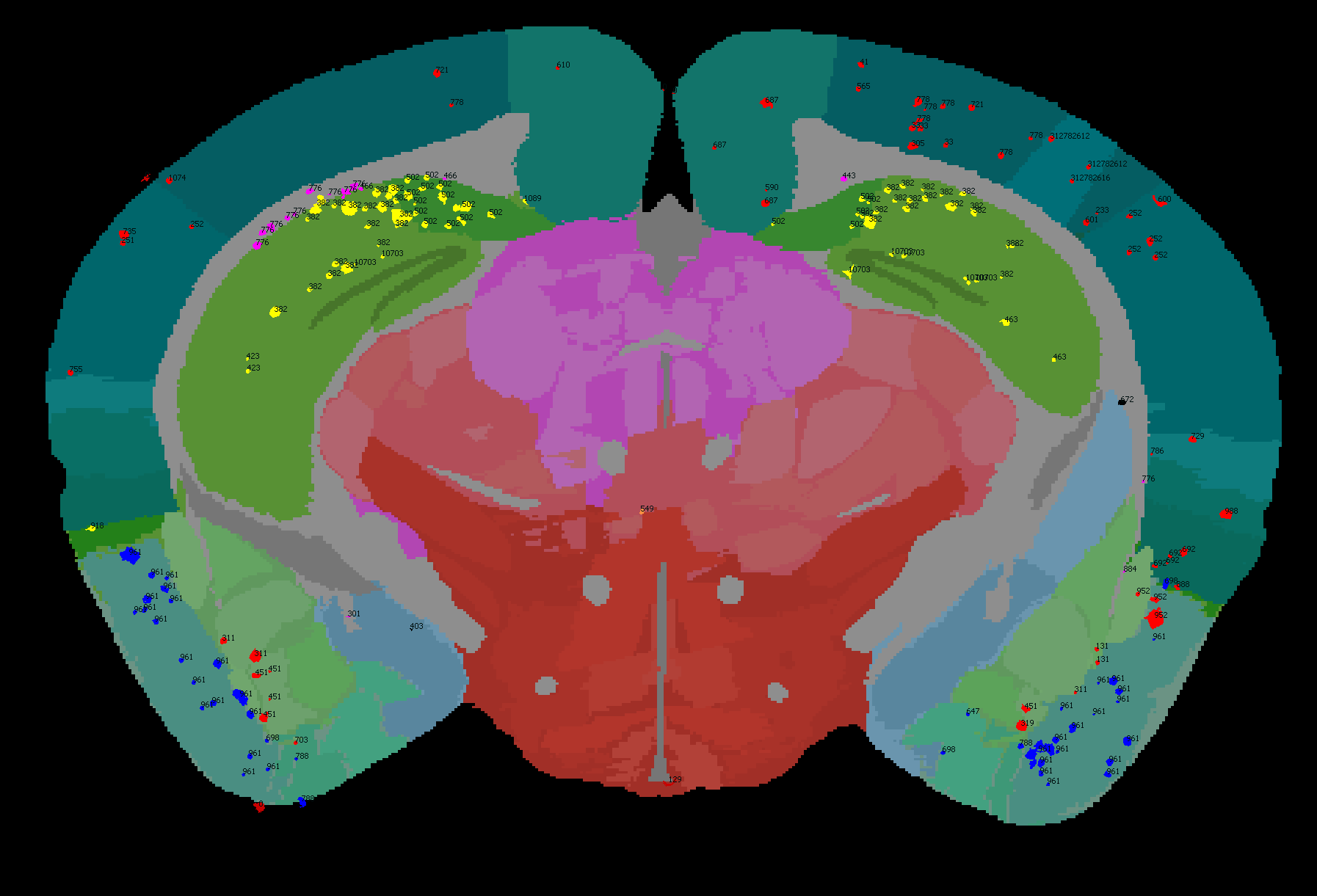

Supplement: Supplementary file 2 [file Data_Sheet_1.ZIP › Supplementary_material_Yates/hAPP/tg2576_m287_1D1_s170_resize_Object Predictions.png]

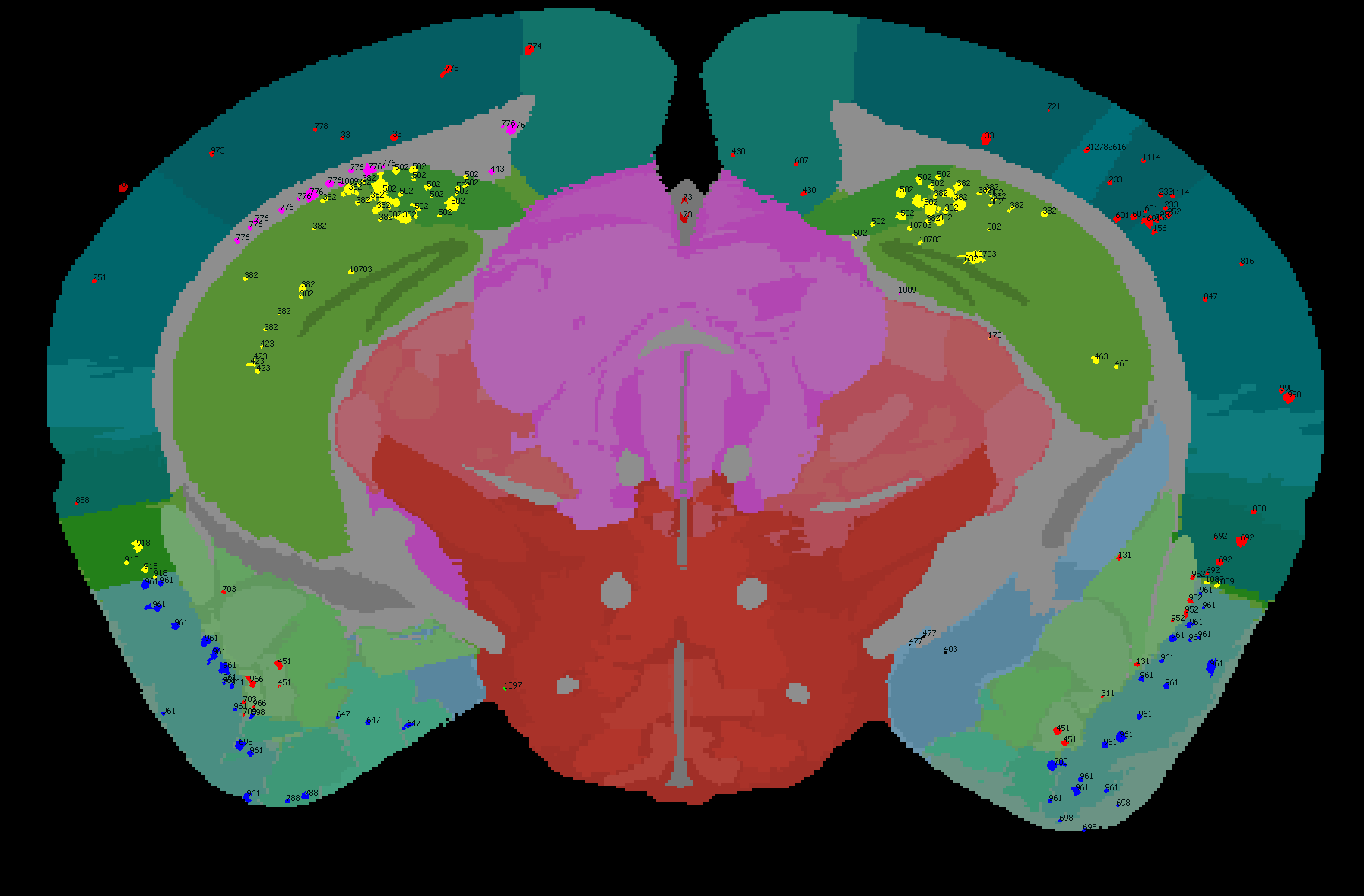

Supplement: Supplementary file 2 [file Data_Sheet_1.ZIP › Supplementary_material_Yates/hAPP/tg2576_m287_1D1_s174_resize_Object Predictions.png]

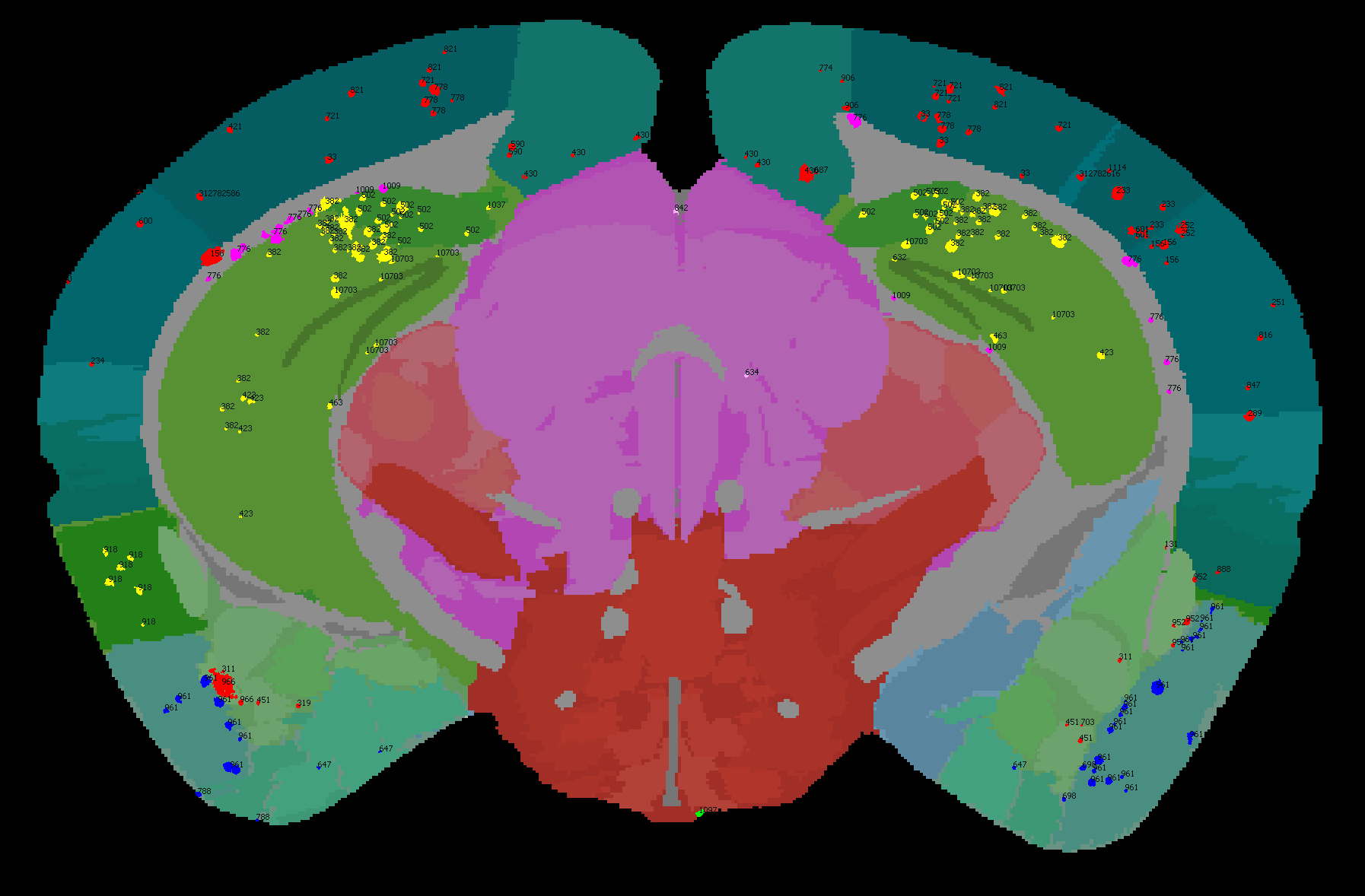

Supplement: Supplementary file 2 [file Data_Sheet_1.ZIP › Supplementary_material_Yates/hAPP/tg2576_m287_1D1_s178_resize_Object Predictions.png]

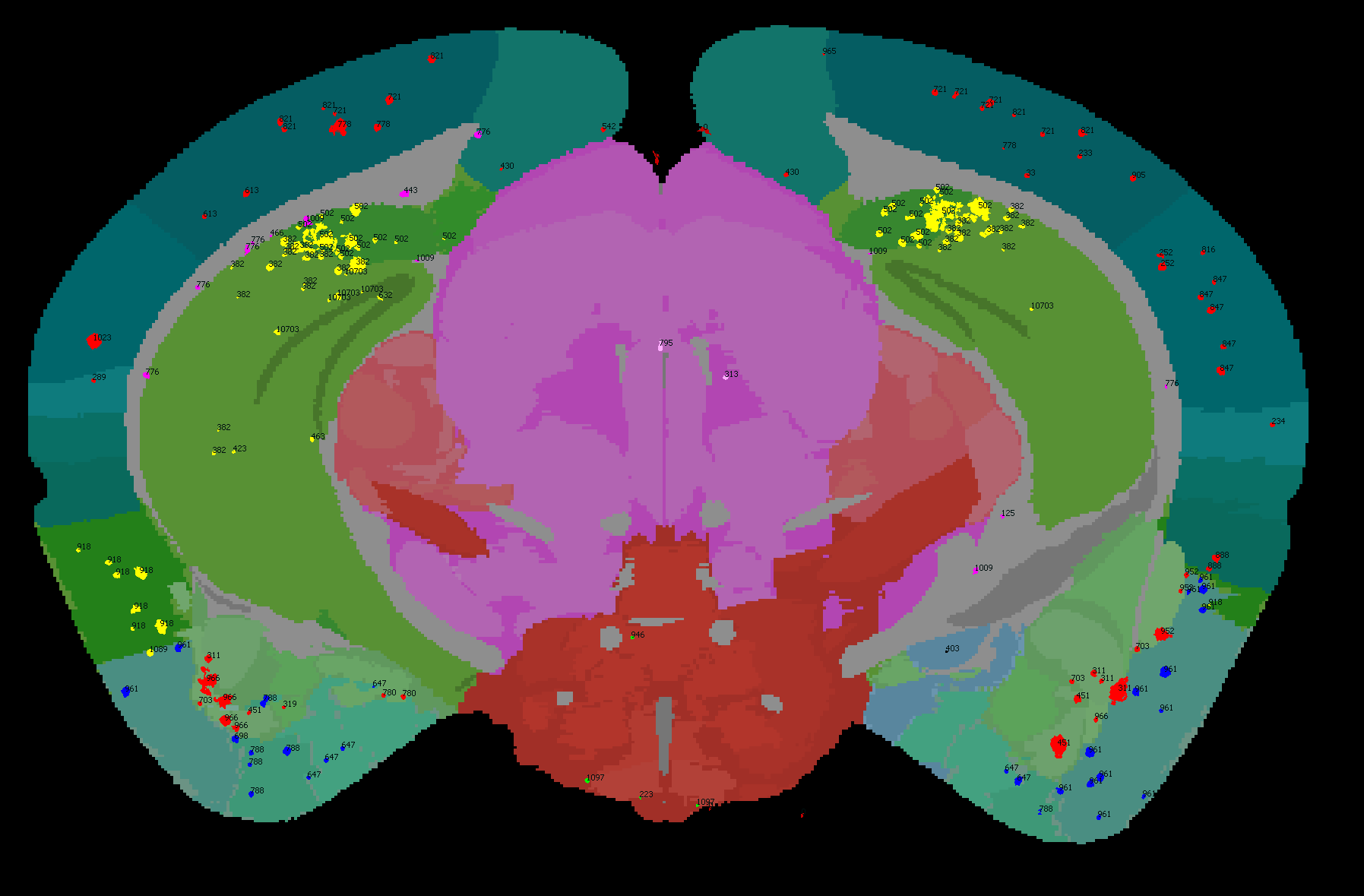

Supplement: Supplementary file 2 [file Data_Sheet_1.ZIP › Supplementary_material_Yates/hAPP/tg2576_m287_1D1_s182_resize_Object Predictions.png]

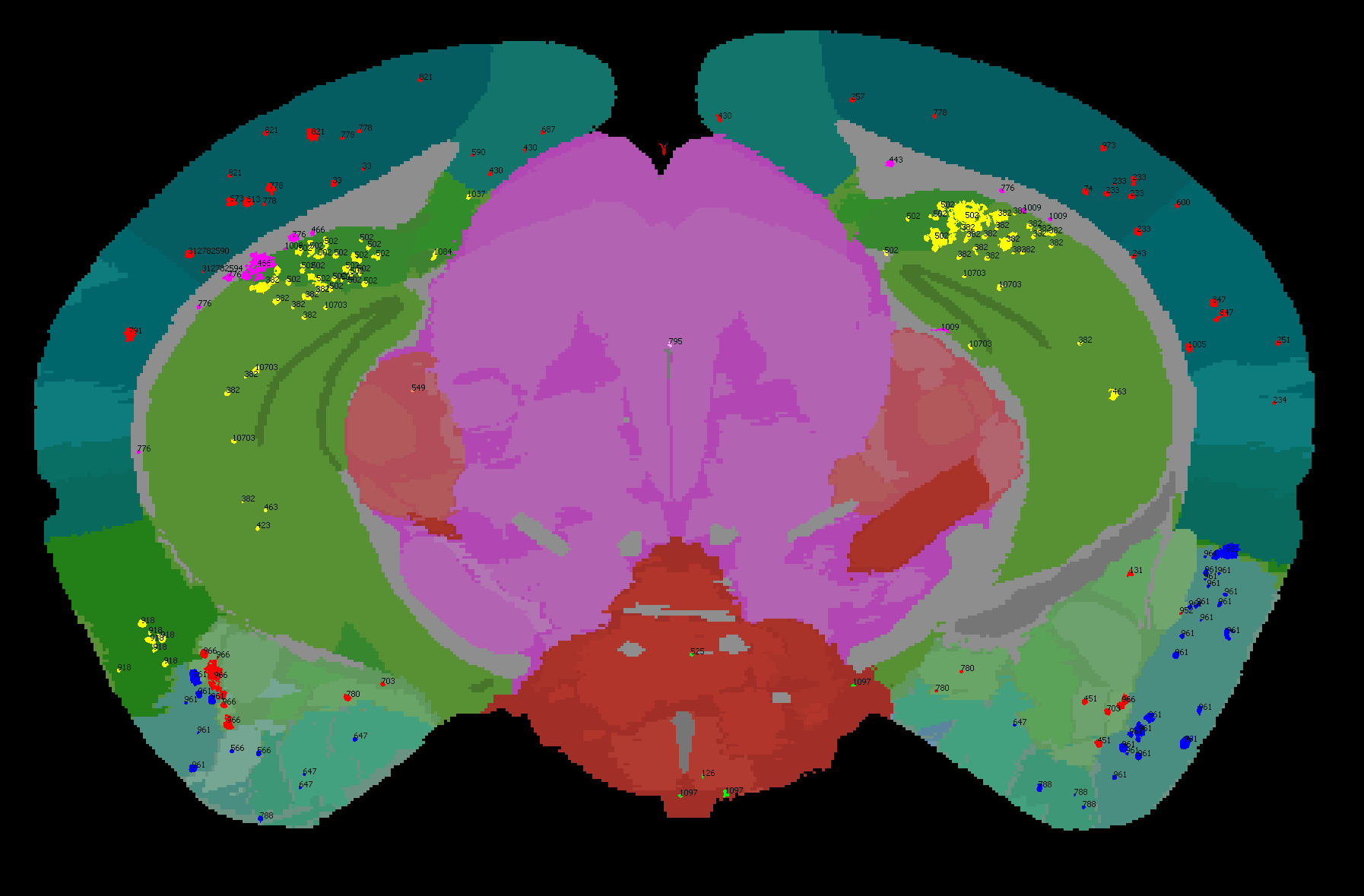

Supplement: Supplementary file 2 [file Data_Sheet_1.ZIP › Supplementary_material_Yates/hAPP/tg2576_m287_1D1_s186_resize_Object Predictions.png]

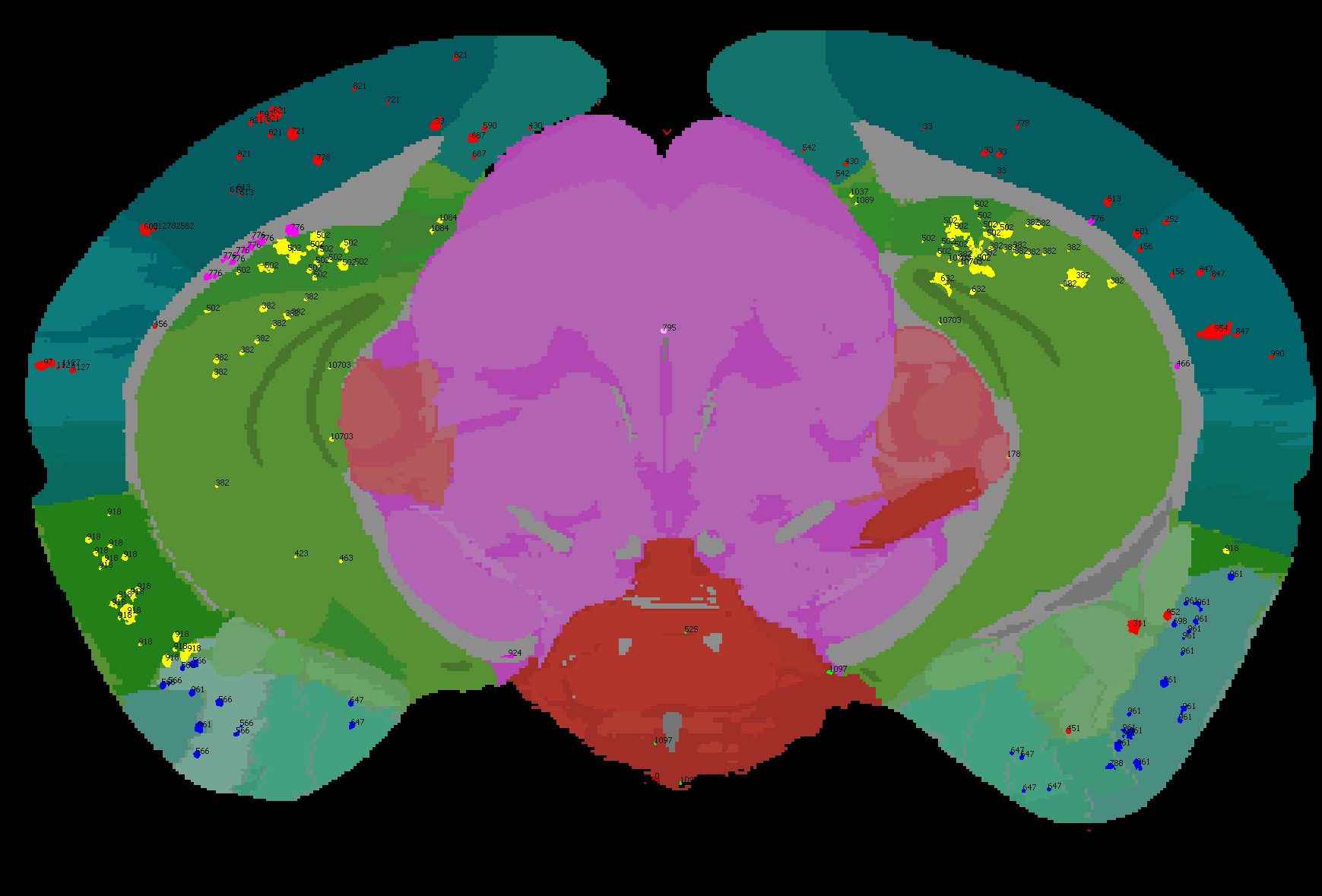

Supplement: Supplementary file 2 [file Data_Sheet_1.ZIP › Supplementary_material_Yates/hAPP/tg2576_m287_1D1_s190_resize_Object Predictions.png]

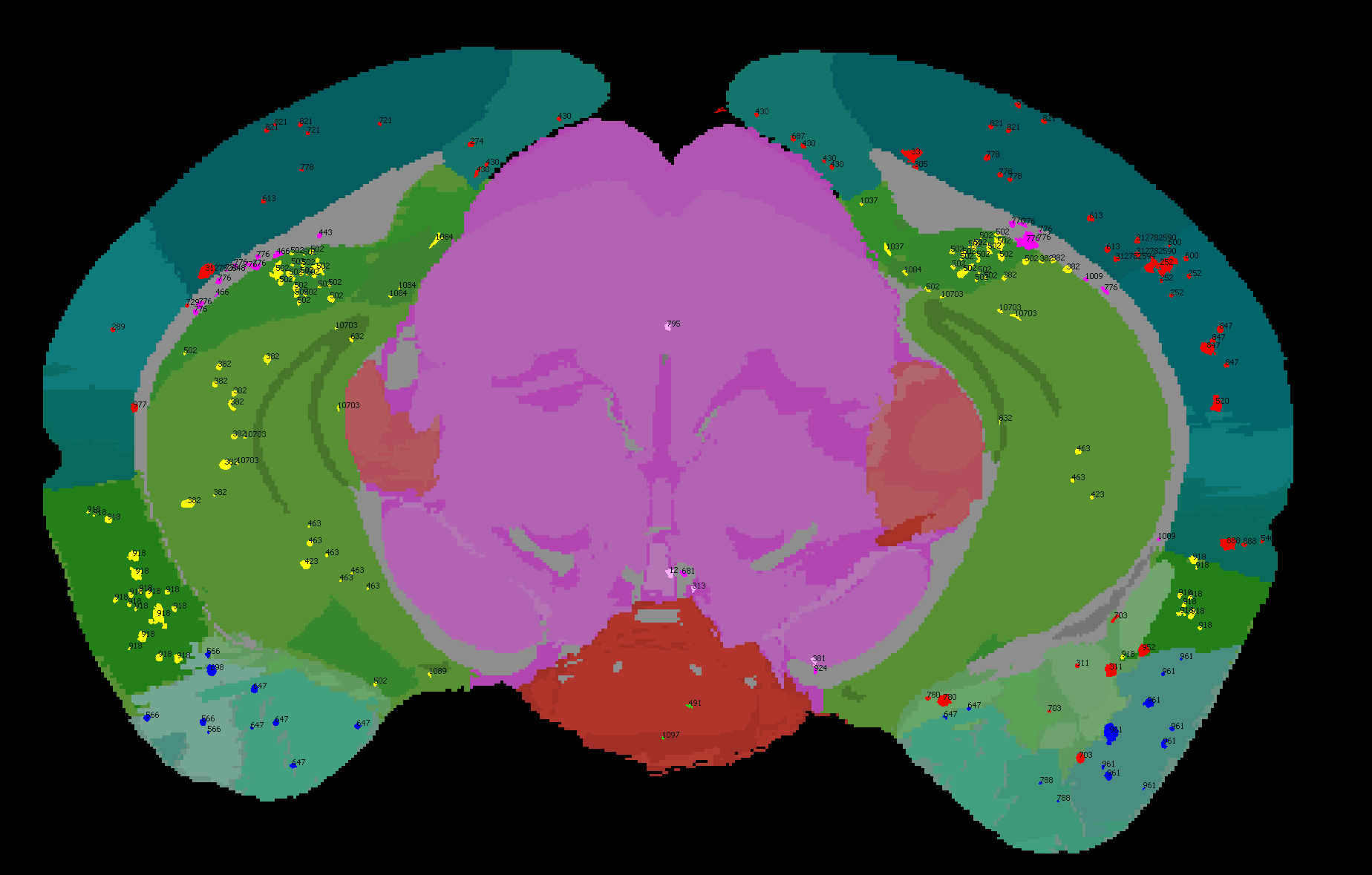

Supplement: Supplementary file 2 [file Data_Sheet_1.ZIP › Supplementary_material_Yates/hAPP/tg2576_m287_1D1_s194_resize_Object Predictions.png]

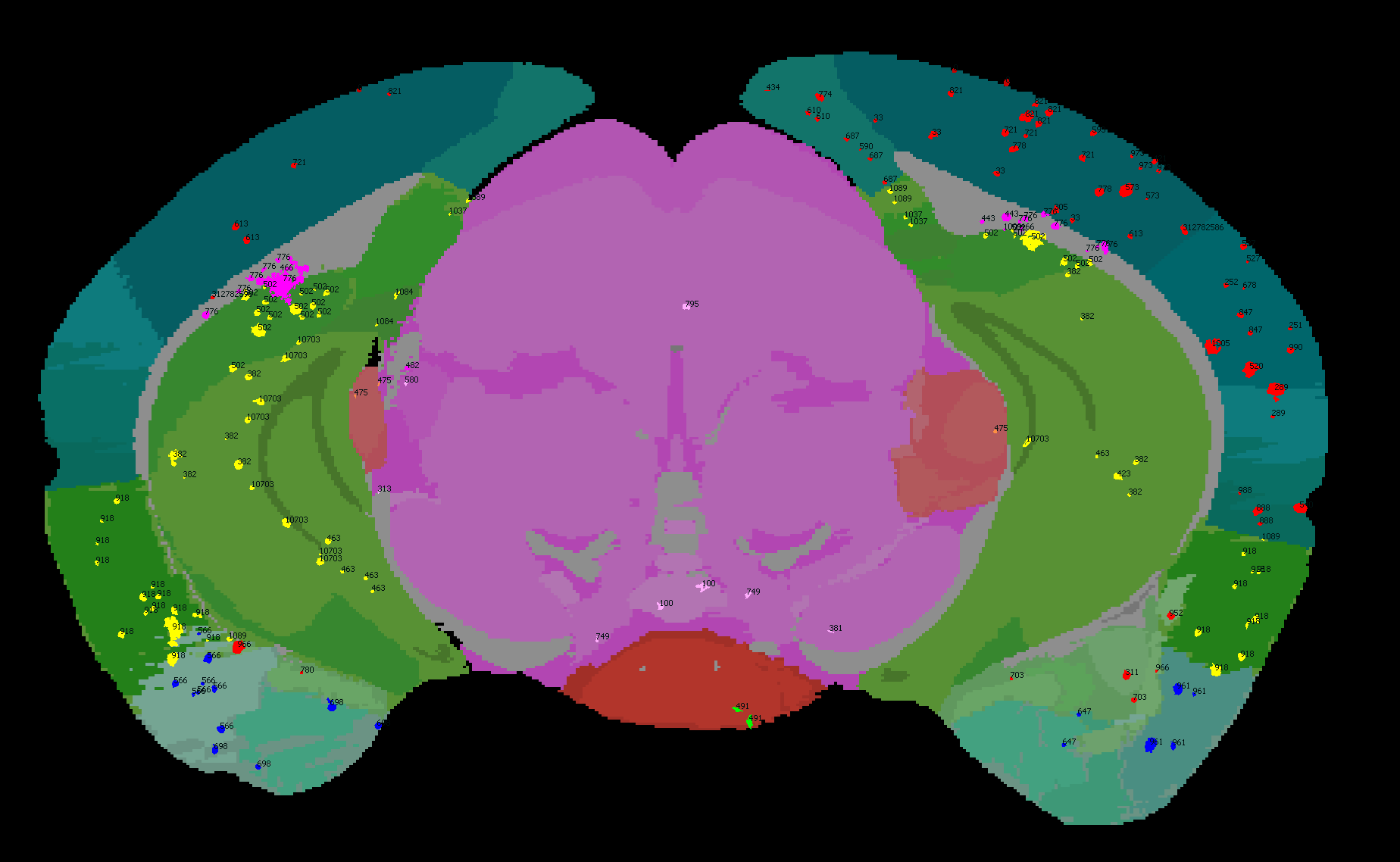

Supplement: Supplementary file 2 [file Data_Sheet_1.ZIP › Supplementary_material_Yates/hAPP/tg2576_m287_1D1_s198_resize_Object Predictions.png]

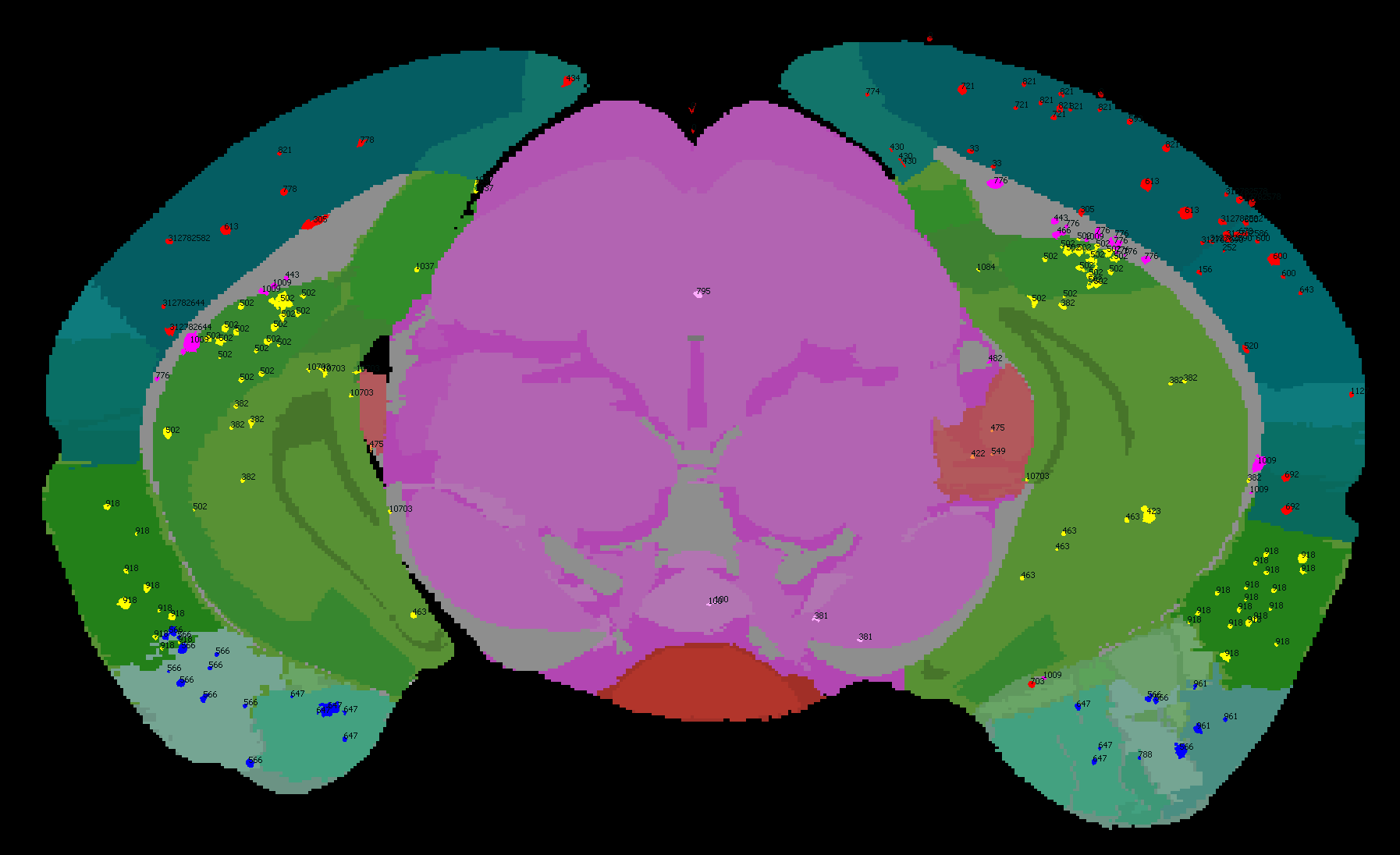

Supplement: Supplementary file 2 [file Data_Sheet_1.ZIP › Supplementary_material_Yates/hAPP/tg2576_m287_1D1_s202_resize_Object Predictions.png]

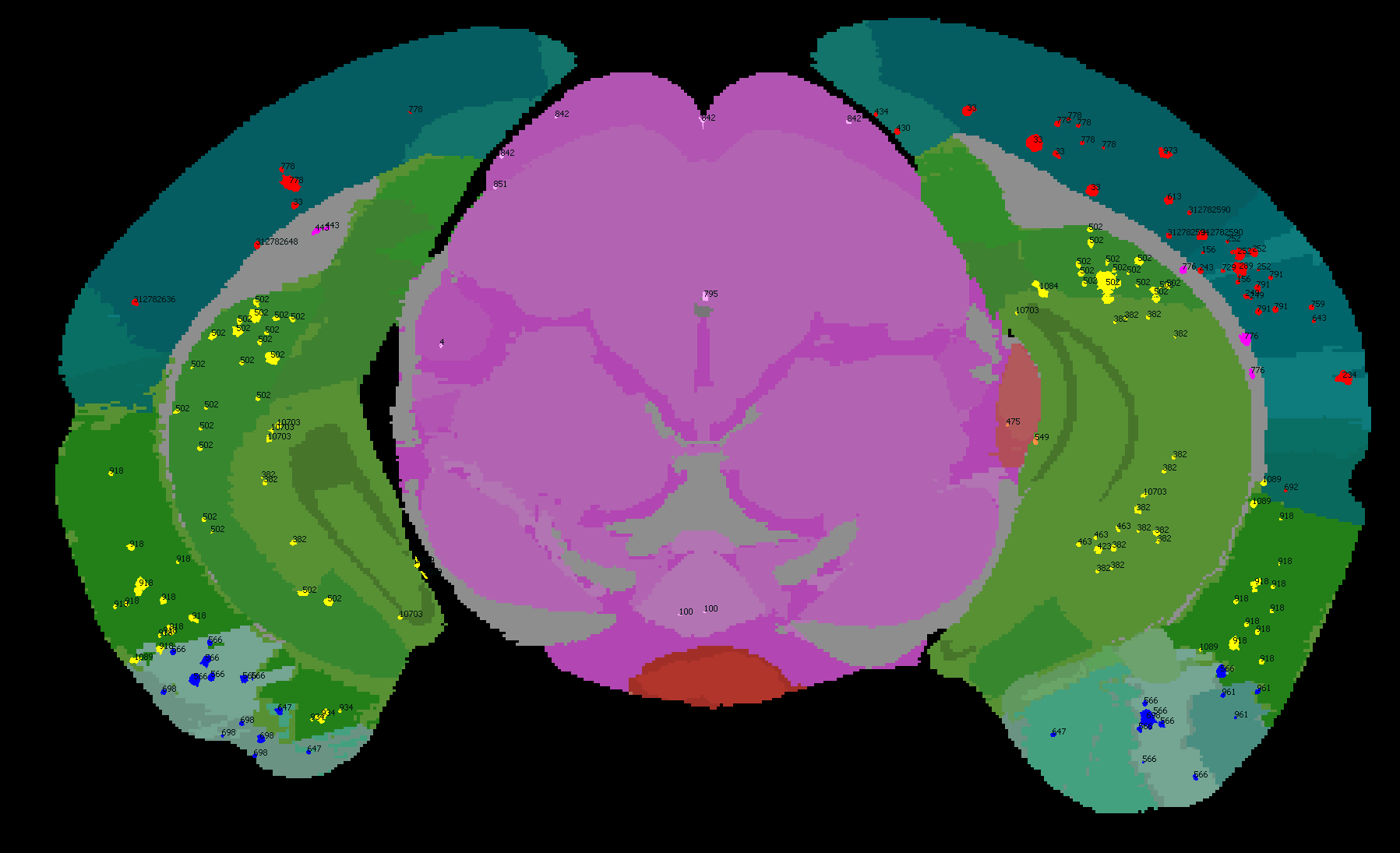

Supplement: Supplementary file 2 [file Data_Sheet_1.ZIP › Supplementary_material_Yates/hAPP/tg2576_m287_1D1_s206_resize_Object Predictions.png]

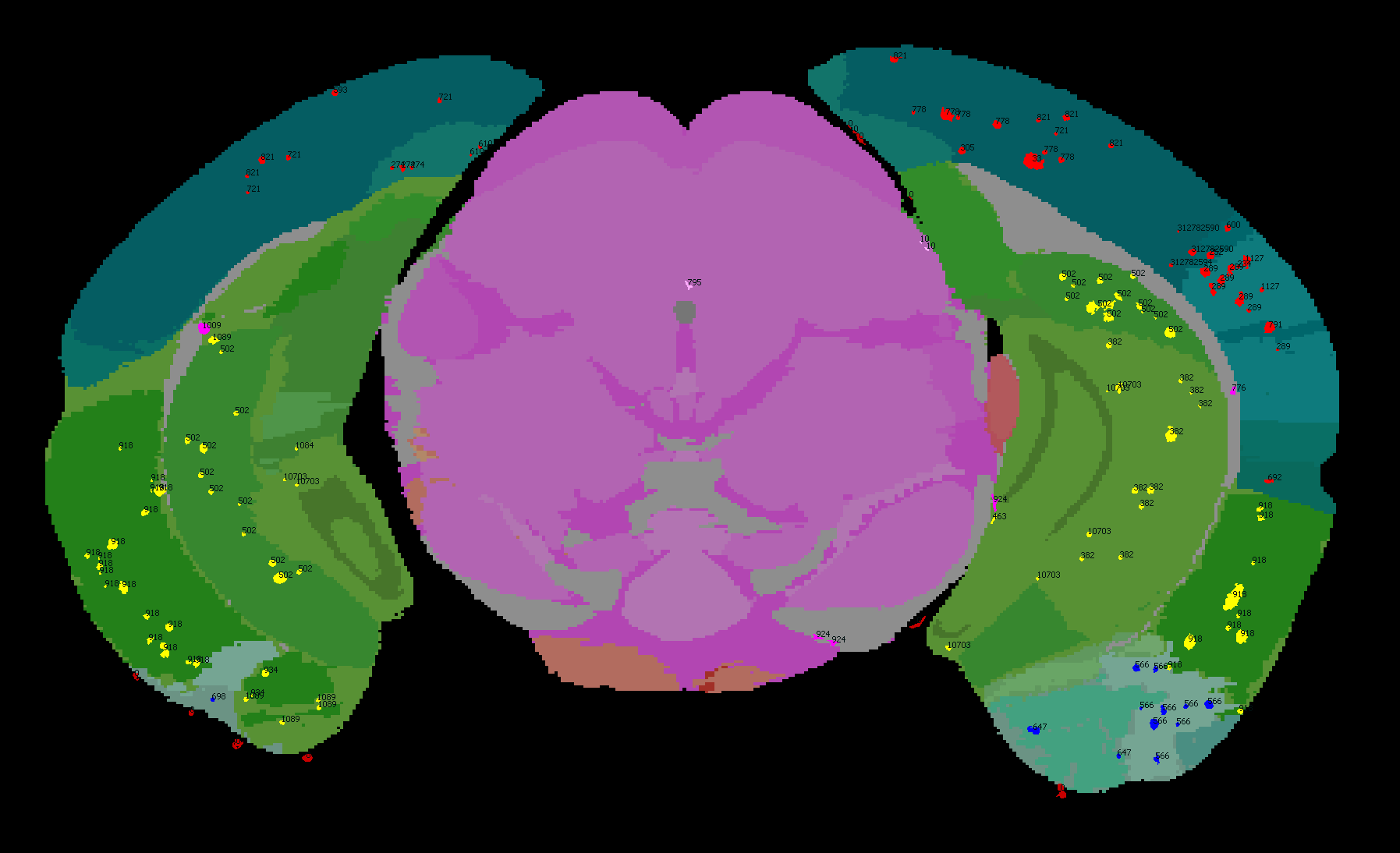

Supplement: Supplementary file 2 [file Data_Sheet_1.ZIP › Supplementary_material_Yates/hAPP/tg2576_m287_1D1_s210_resize_Object Predictions.png]

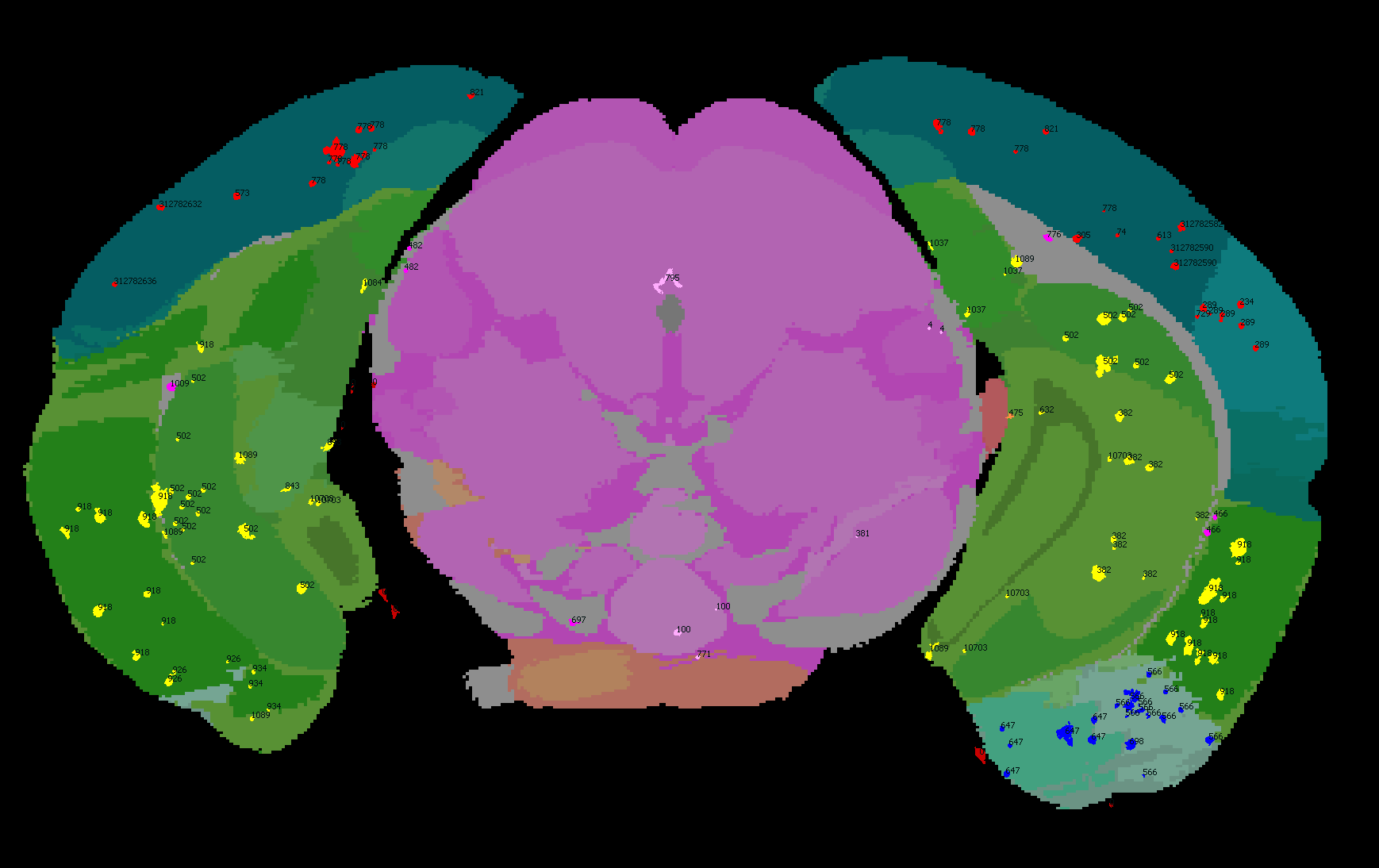

Supplement: Supplementary file 2 [file Data_Sheet_1.ZIP › Supplementary_material_Yates/hAPP/tg2576_m287_1D1_s214_resize_Object Predictions.png]

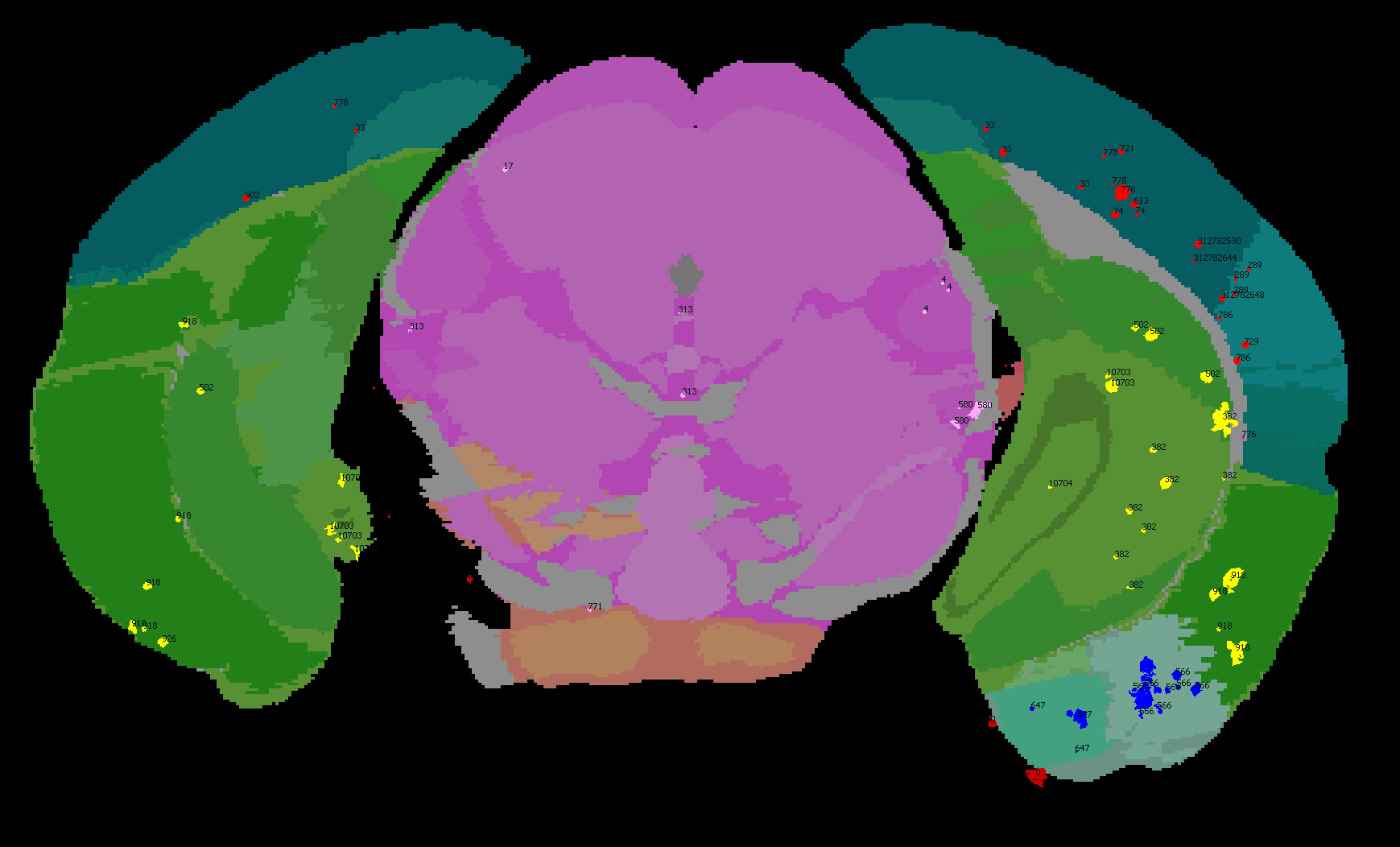

Supplement: Supplementary file 2 [file Data_Sheet_1.ZIP › Supplementary_material_Yates/hAPP/tg2576_m287_1D1_s218_resize_Object Predictions.png]

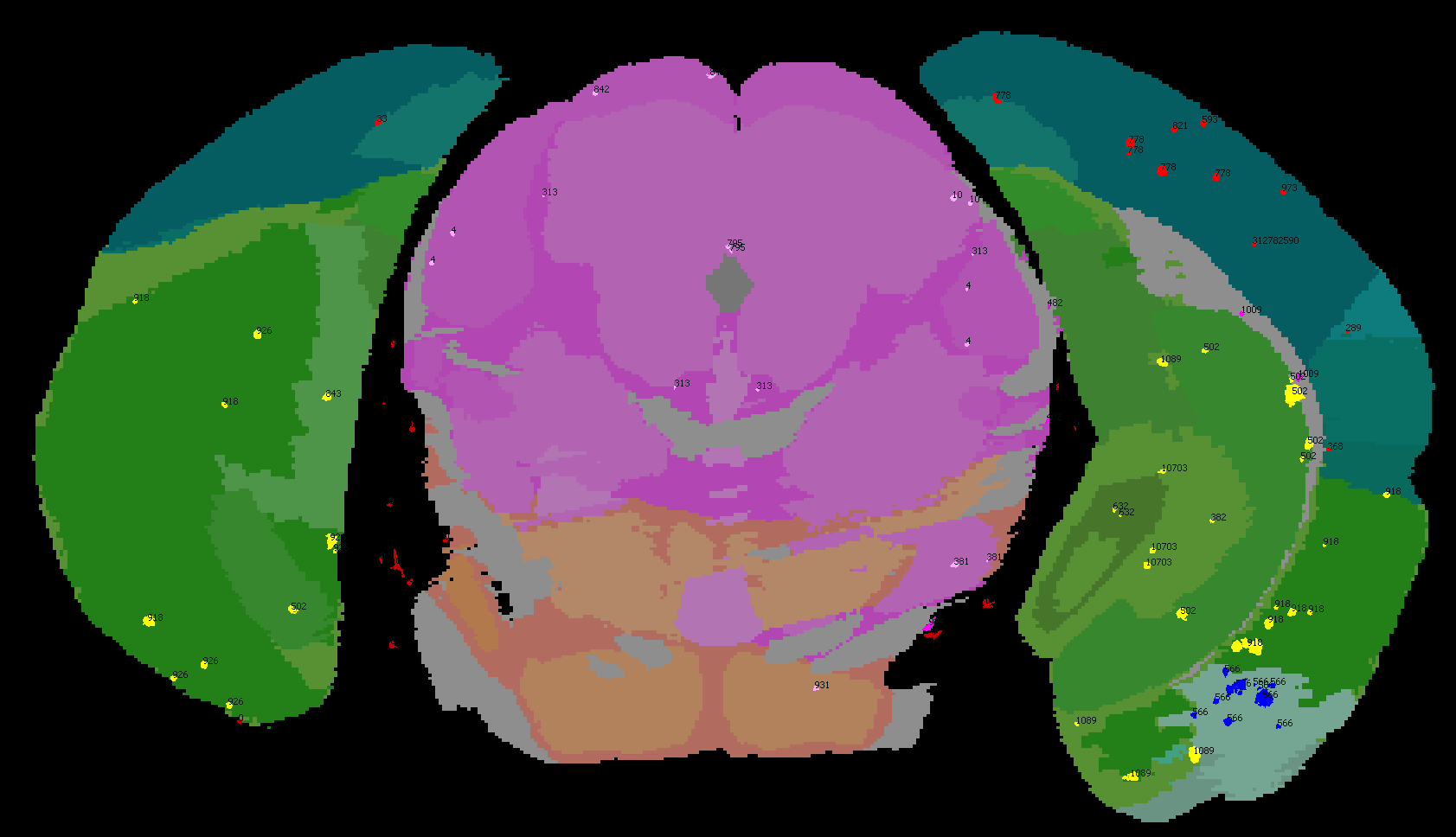

Supplement: Supplementary file 2 [file Data_Sheet_1.ZIP › Supplementary_material_Yates/hAPP/tg2576_m287_1D1_s222_resize_Object Predictions.png]

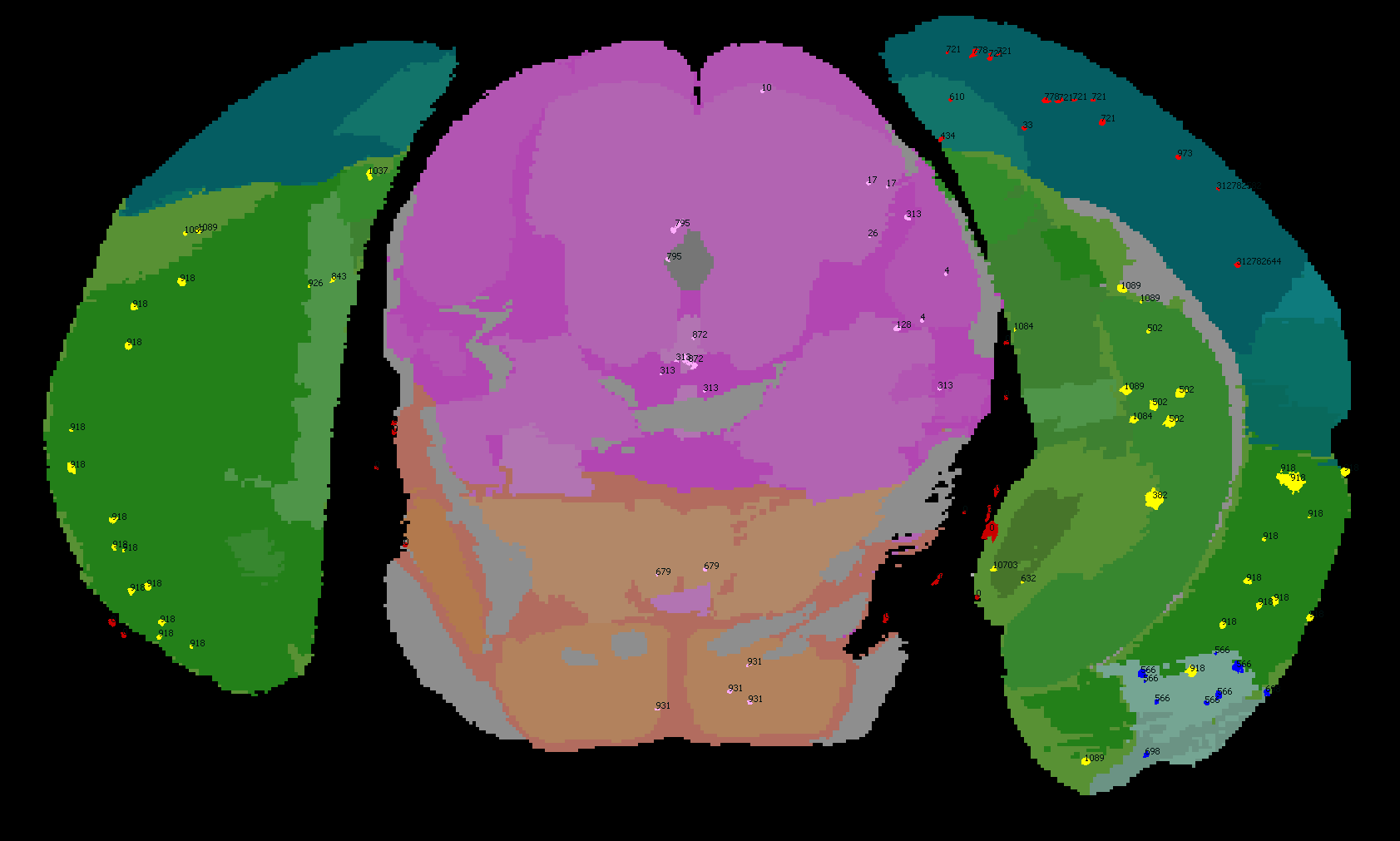

Supplement: Supplementary file 2 [file Data_Sheet_1.ZIP › Supplementary_material_Yates/hAPP/tg2576_m287_1D1_s226_resize_Object Predictions.png]

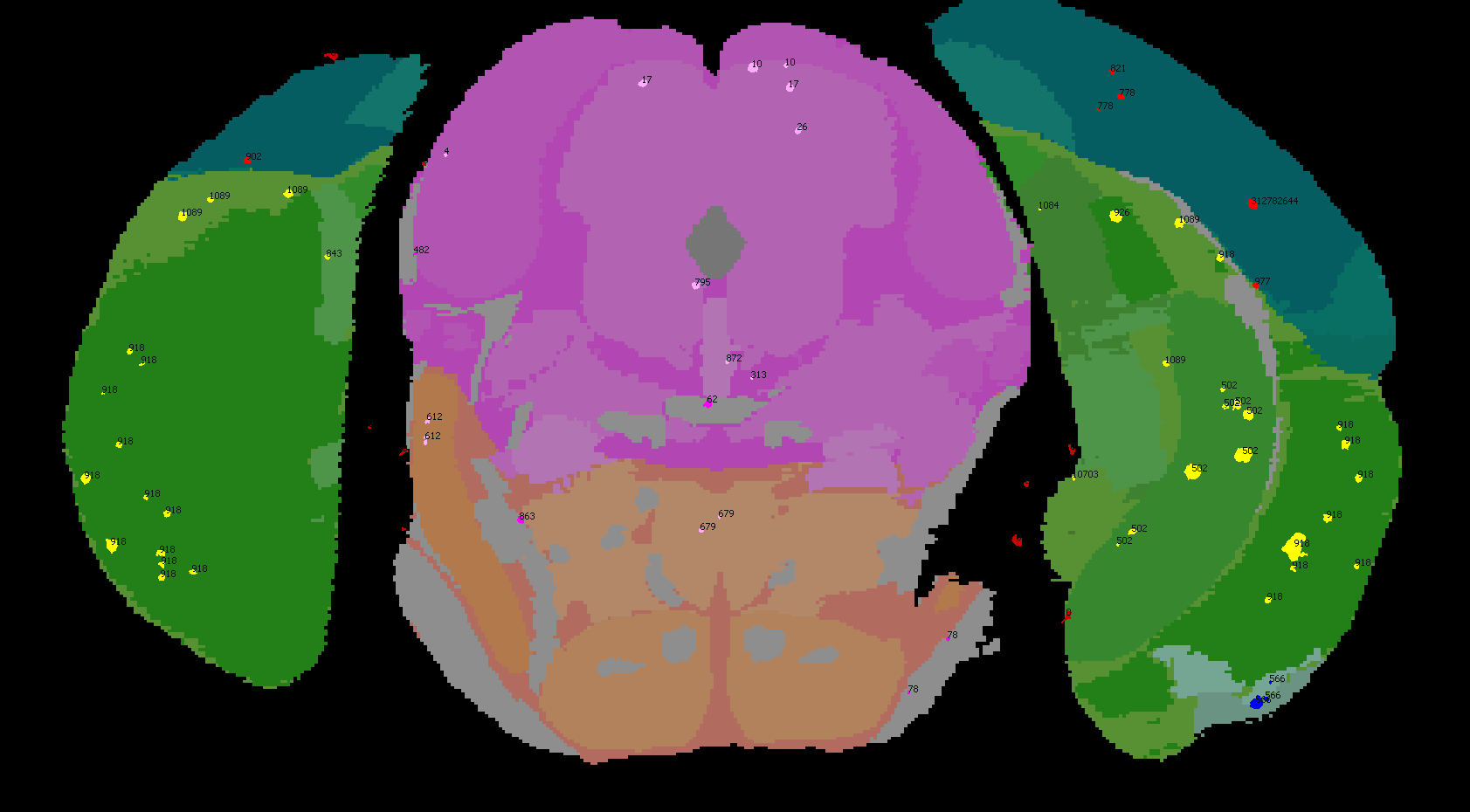

Supplement: Supplementary file 2 [file Data_Sheet_1.ZIP › Supplementary_material_Yates/hAPP/tg2576_m287_1D1_s230_resize_Object Predictions.png]

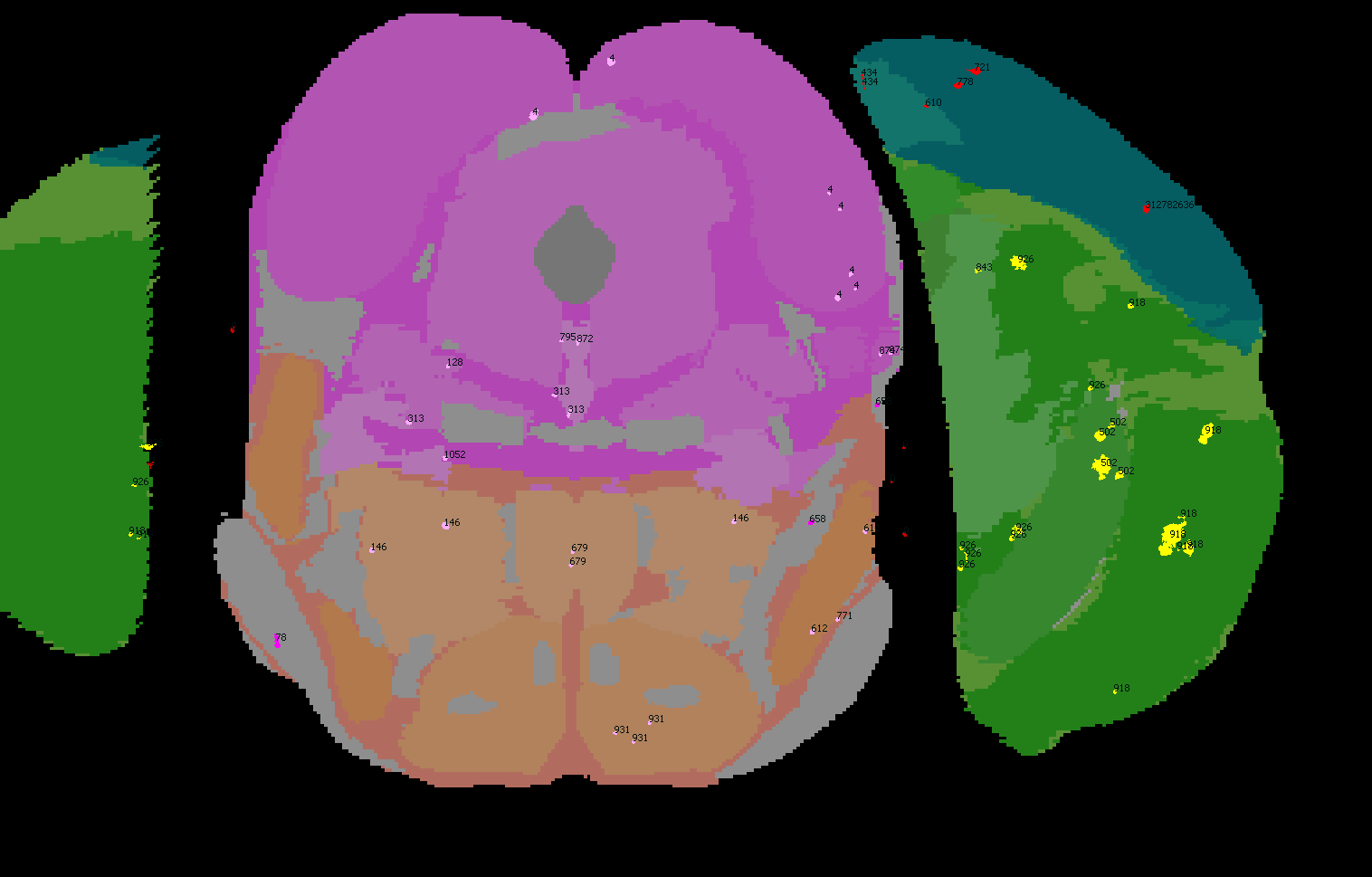

Supplement: Supplementary file 2 [file Data_Sheet_1.ZIP › Supplementary_material_Yates/hAPP/tg2576_m287_1D1_s234_resize_Object Predictions.png]

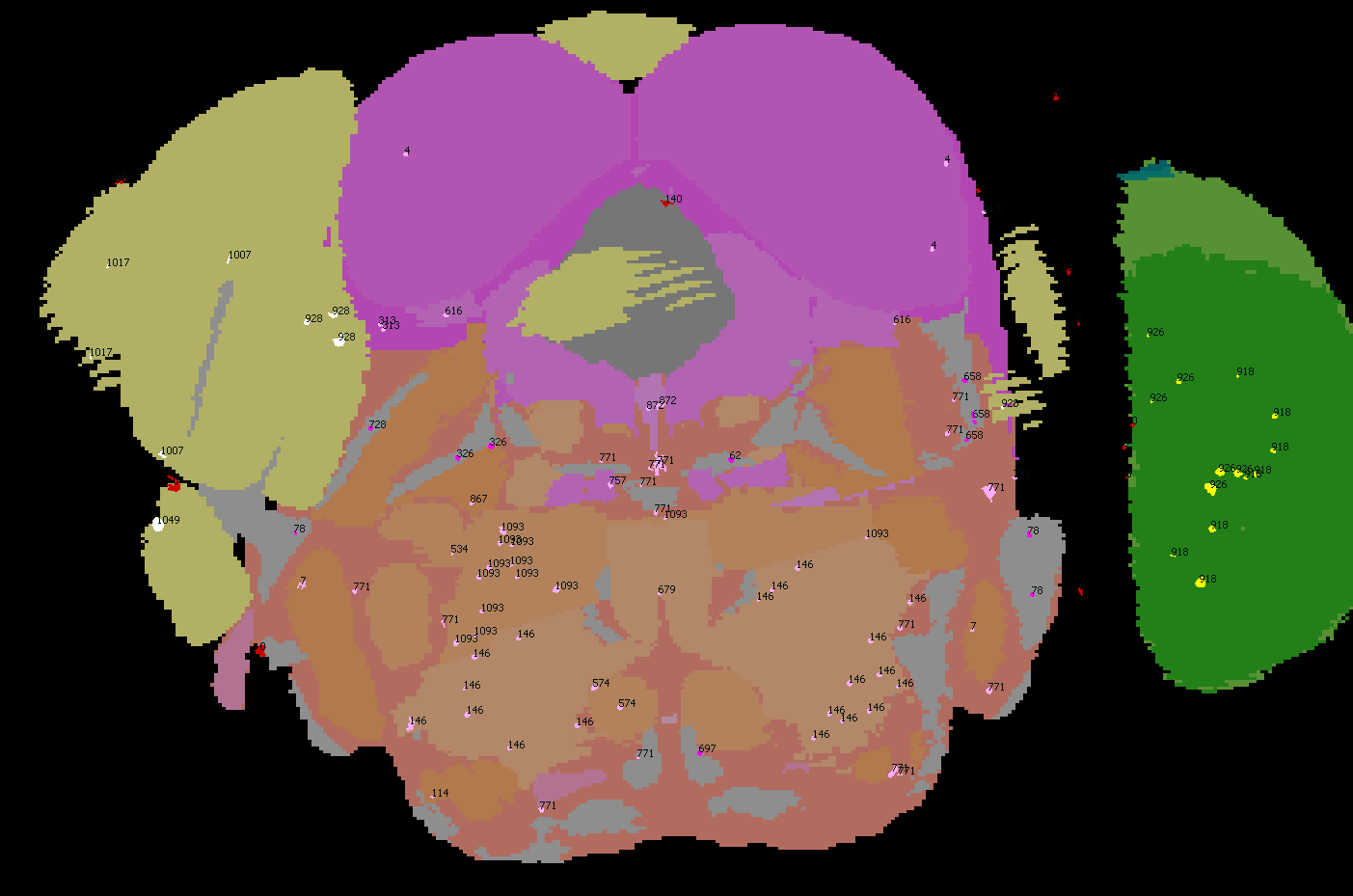

Supplement: Supplementary file 2 [file Data_Sheet_1.ZIP › Supplementary_material_Yates/hAPP/tg2576_m287_1D1_s238_resize_Object Predictions.png]

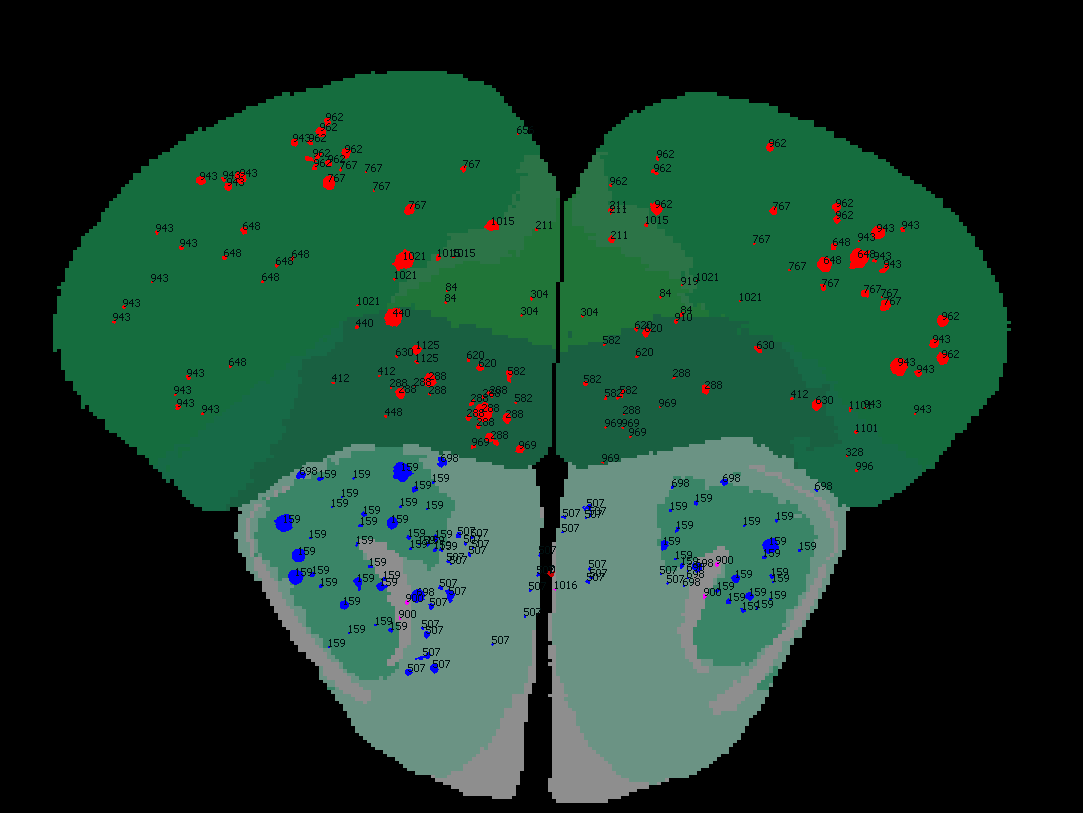

Supplement: Supplementary file 2 [file Data_Sheet_1.ZIP › Supplementary_material_Yates/pan-Abeta/tg2576_m287_4G8_s003_resize_Object Predictions.png]

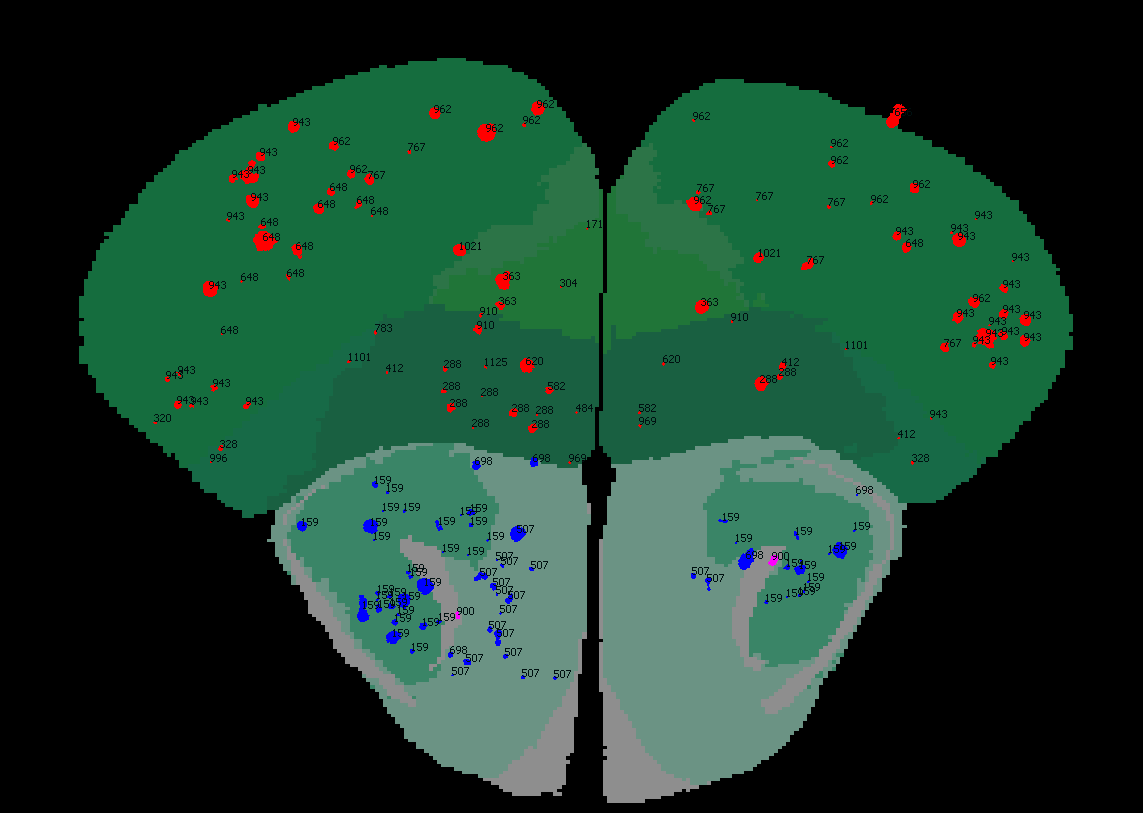

Supplement: Supplementary file 2 [file Data_Sheet_1.ZIP › Supplementary_material_Yates/pan-Abeta/tg2576_m287_4G8_s007_resize_Object Predictions.png]

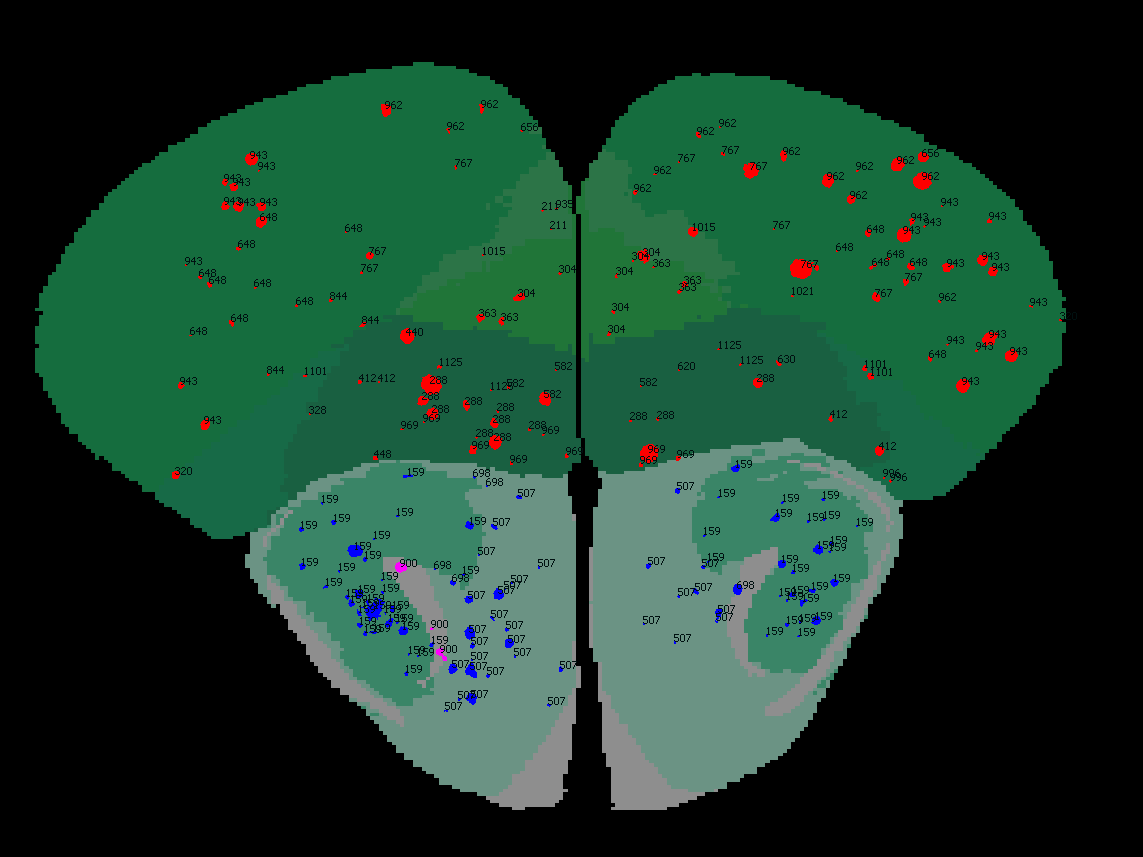

Supplement: Supplementary file 2 [file Data_Sheet_1.ZIP › Supplementary_material_Yates/pan-Abeta/tg2576_m287_4G8_s011_resize_Object Predictions.png]

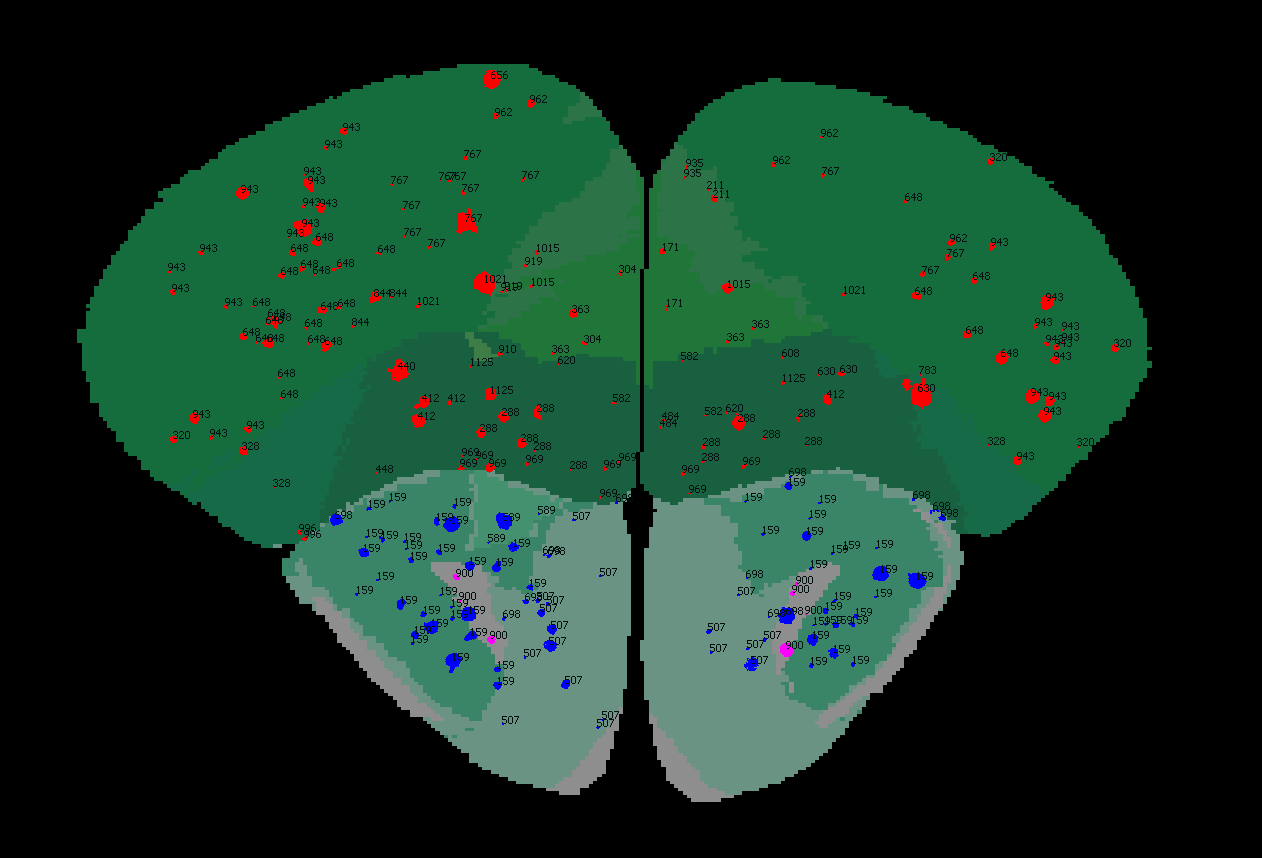

Supplement: Supplementary file 2 [file Data_Sheet_1.ZIP › Supplementary_material_Yates/pan-Abeta/tg2576_m287_4G8_s015_resize_Object Predictions.png]

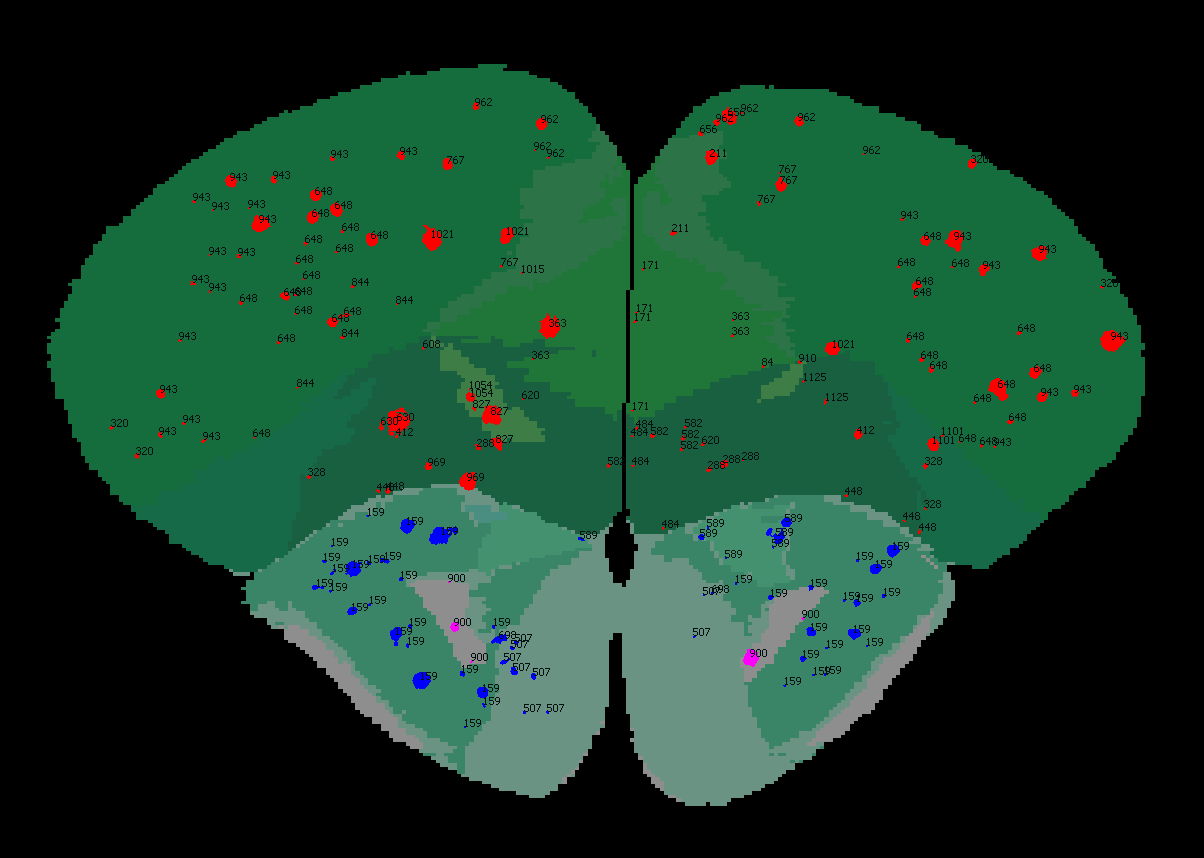

Supplement: Supplementary file 2 [file Data_Sheet_1.ZIP › Supplementary_material_Yates/pan-Abeta/tg2576_m287_4G8_s019_resize_Object Predictions.png]

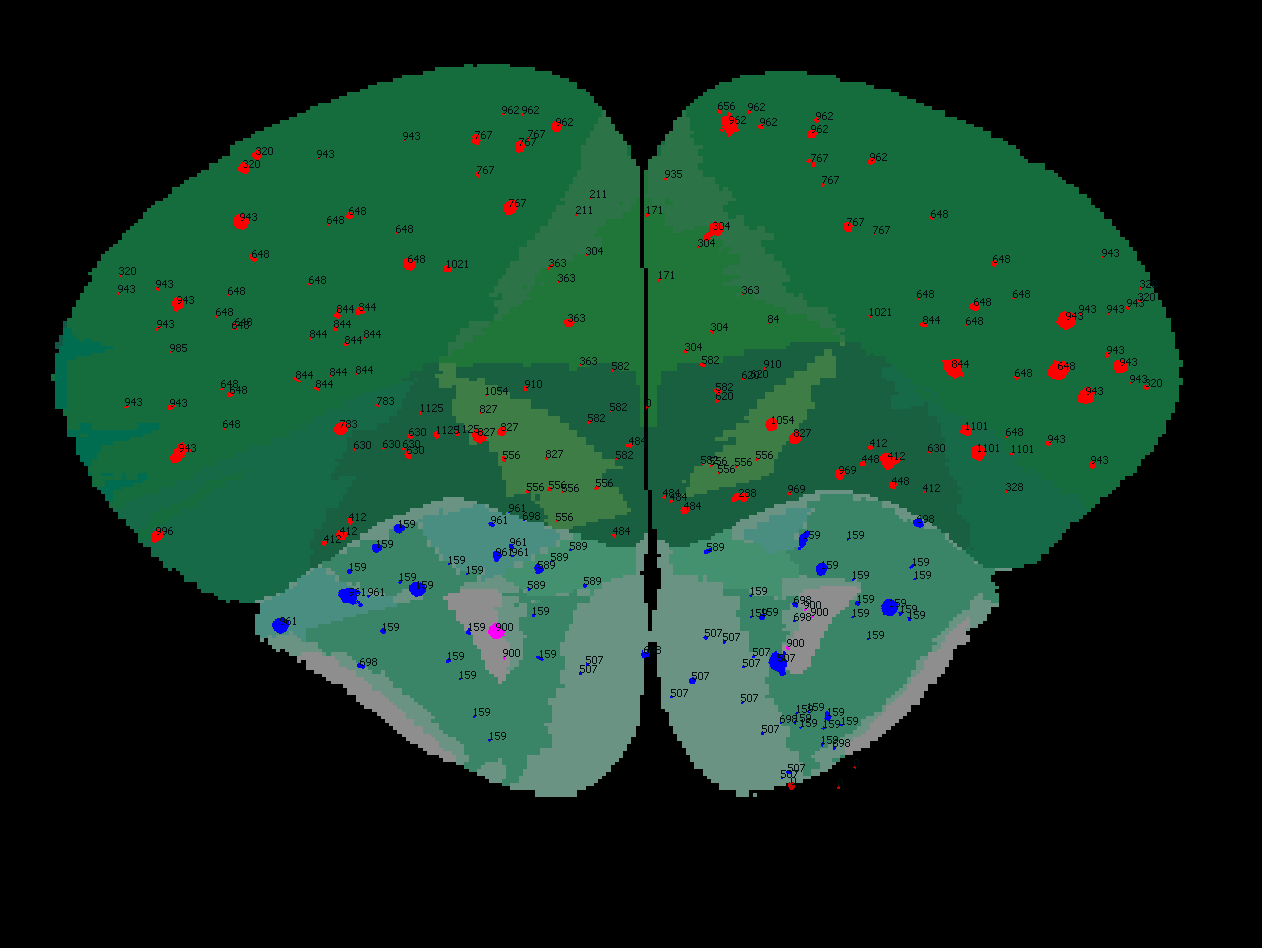

Supplement: Supplementary file 2 [file Data_Sheet_1.ZIP › Supplementary_material_Yates/pan-Abeta/tg2576_m287_4G8_s023_resize_Object Predictions.png]

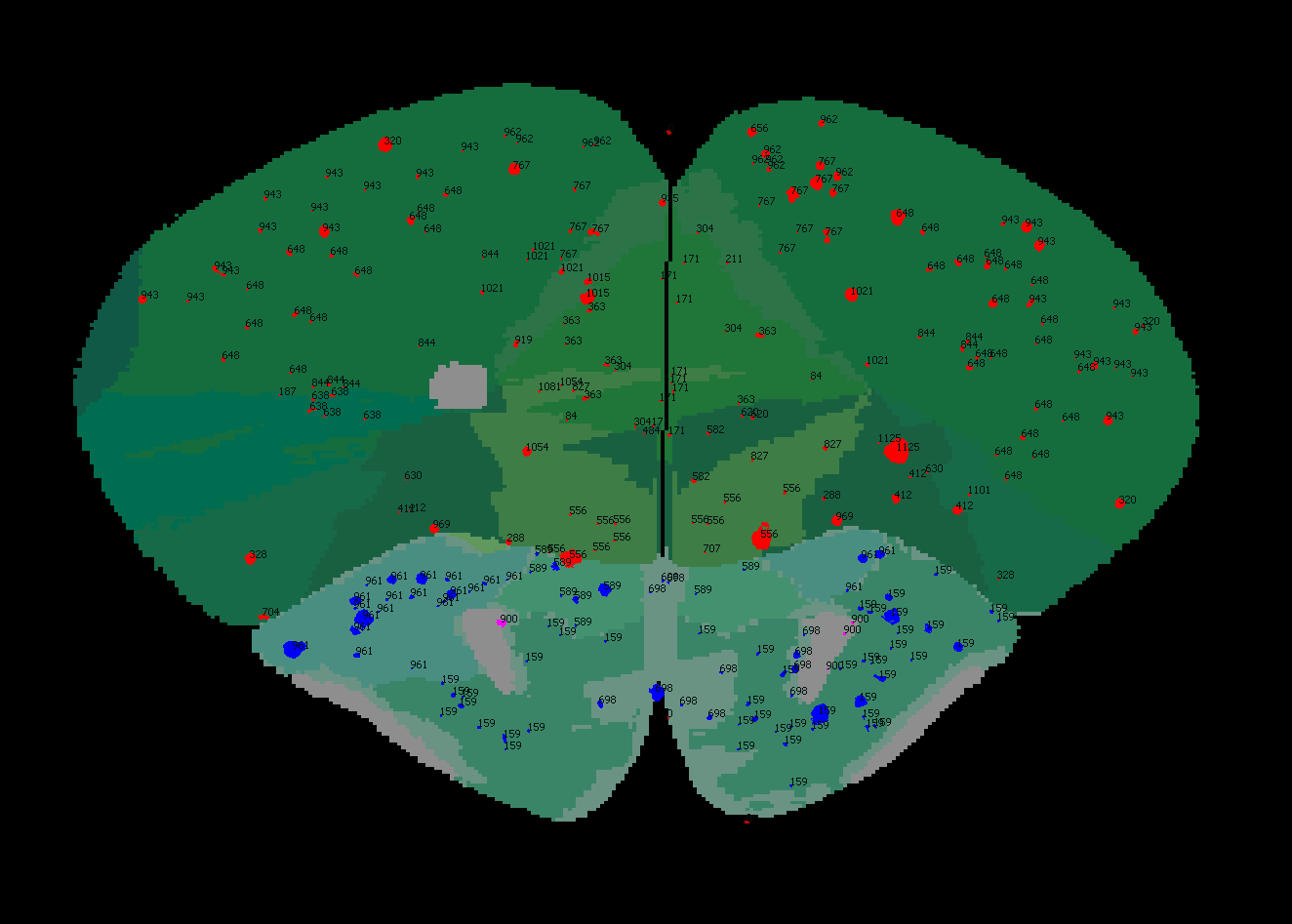

Supplement: Supplementary file 2 [file Data_Sheet_1.ZIP › Supplementary_material_Yates/pan-Abeta/tg2576_m287_4G8_s027_resize_Object Predictions.png]

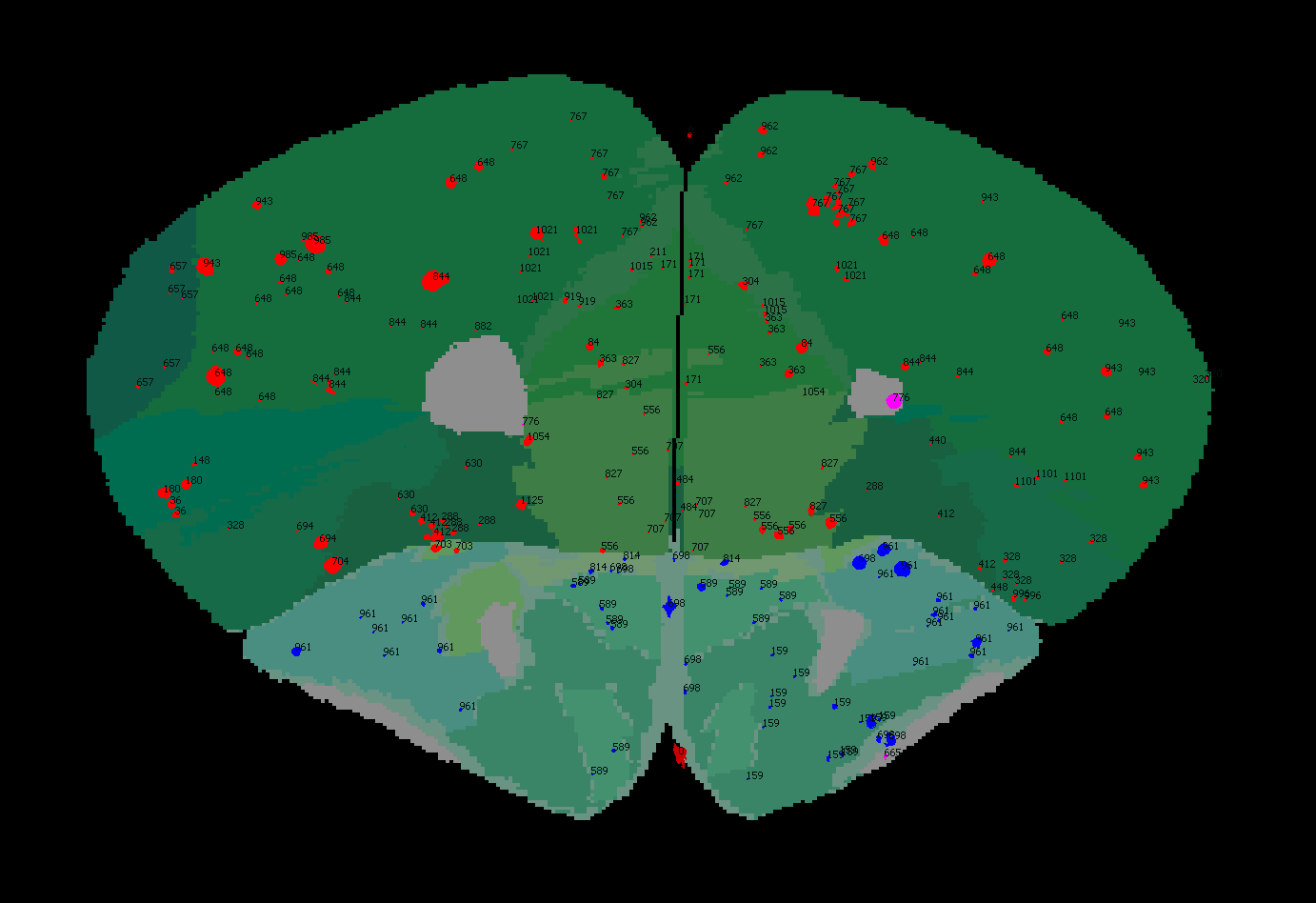

Supplement: Supplementary file 2 [file Data_Sheet_1.ZIP › Supplementary_material_Yates/pan-Abeta/tg2576_m287_4G8_s031_resize_Object Predictions.png]

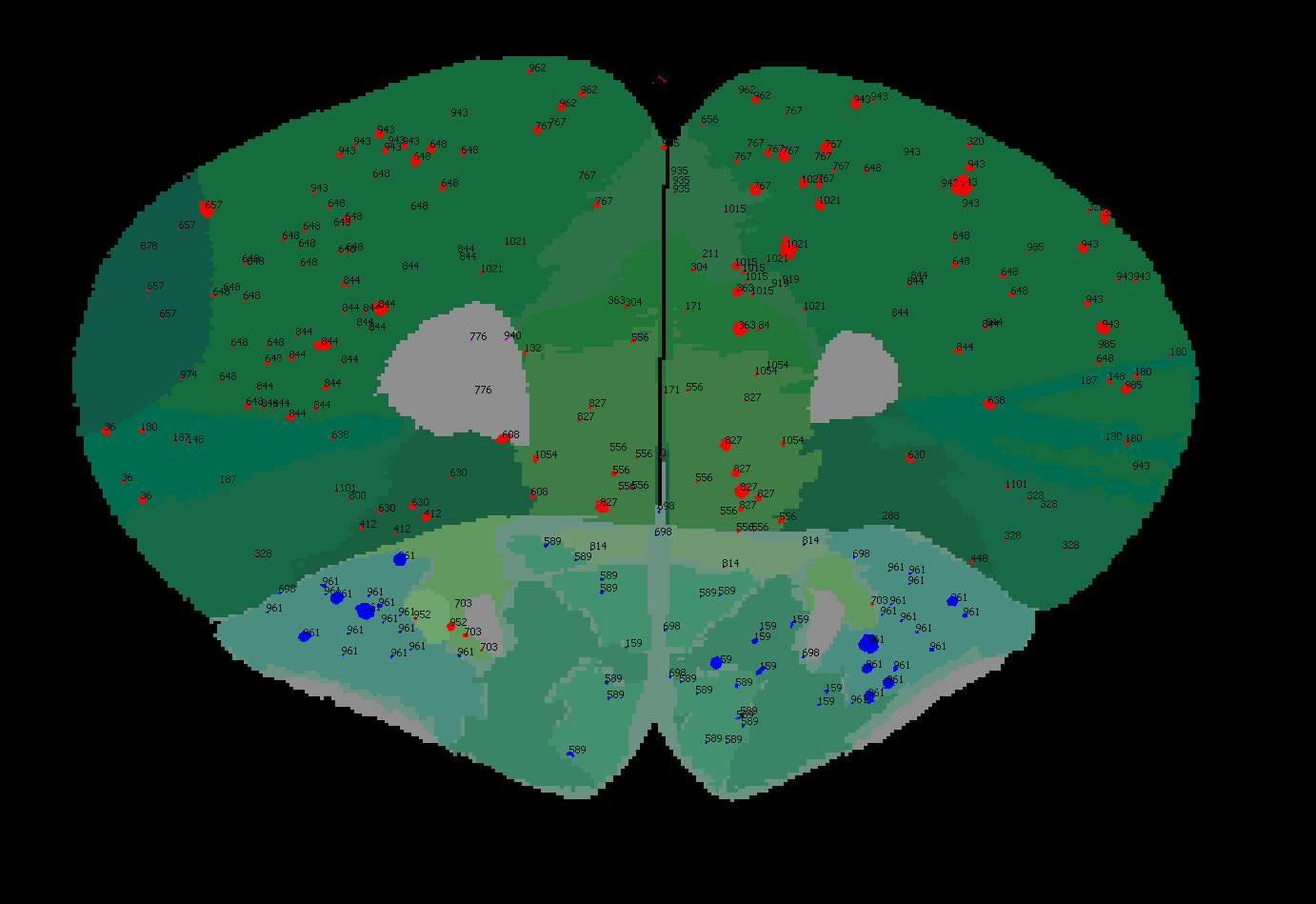

Supplement: Supplementary file 2 [file Data_Sheet_1.ZIP › Supplementary_material_Yates/pan-Abeta/tg2576_m287_4G8_s035_resize_Object Predictions.png]

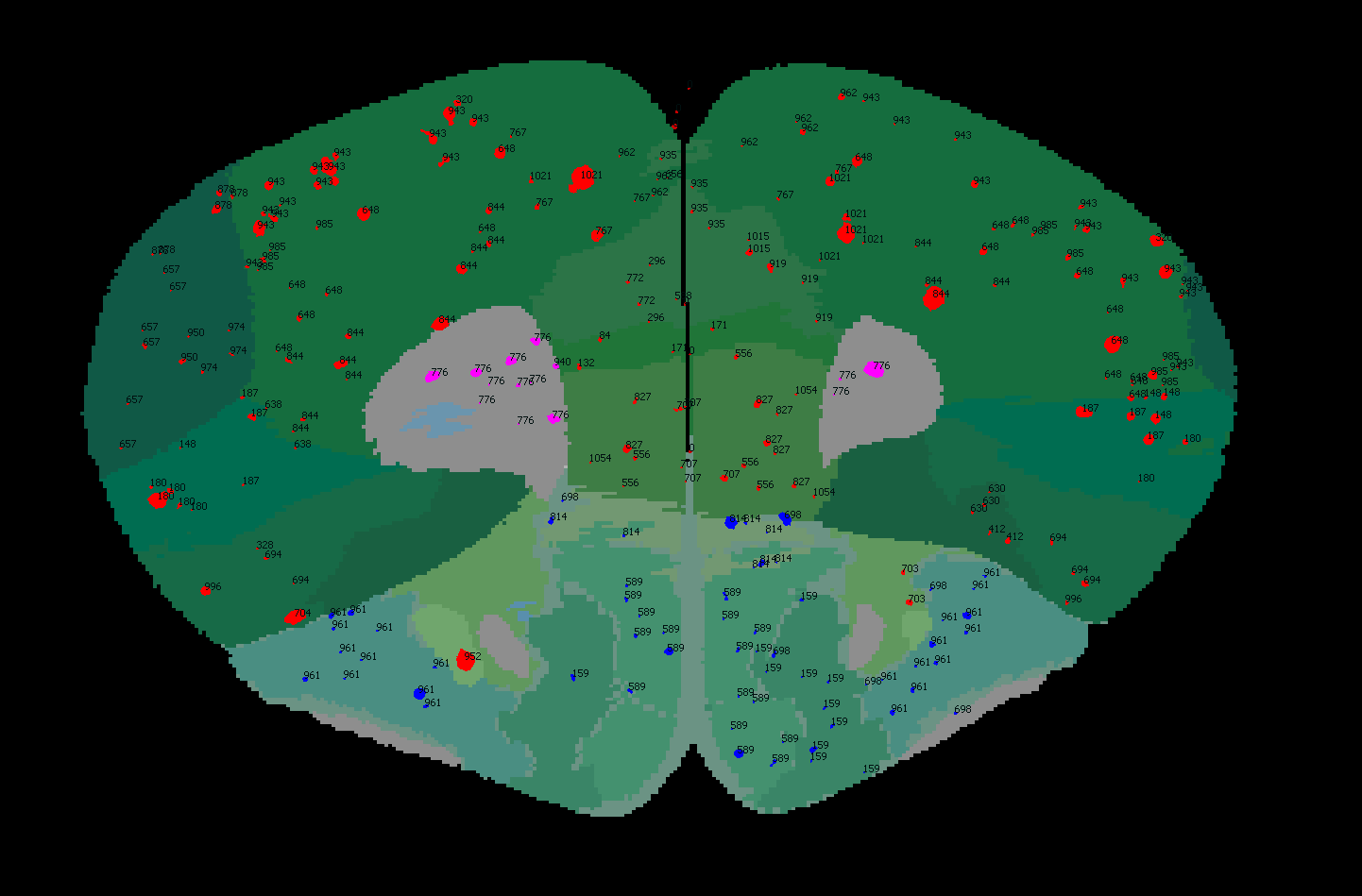

Supplement: Supplementary file 2 [file Data_Sheet_1.ZIP › Supplementary_material_Yates/pan-Abeta/tg2576_m287_4G8_s039_resize_Object Predictions.png]

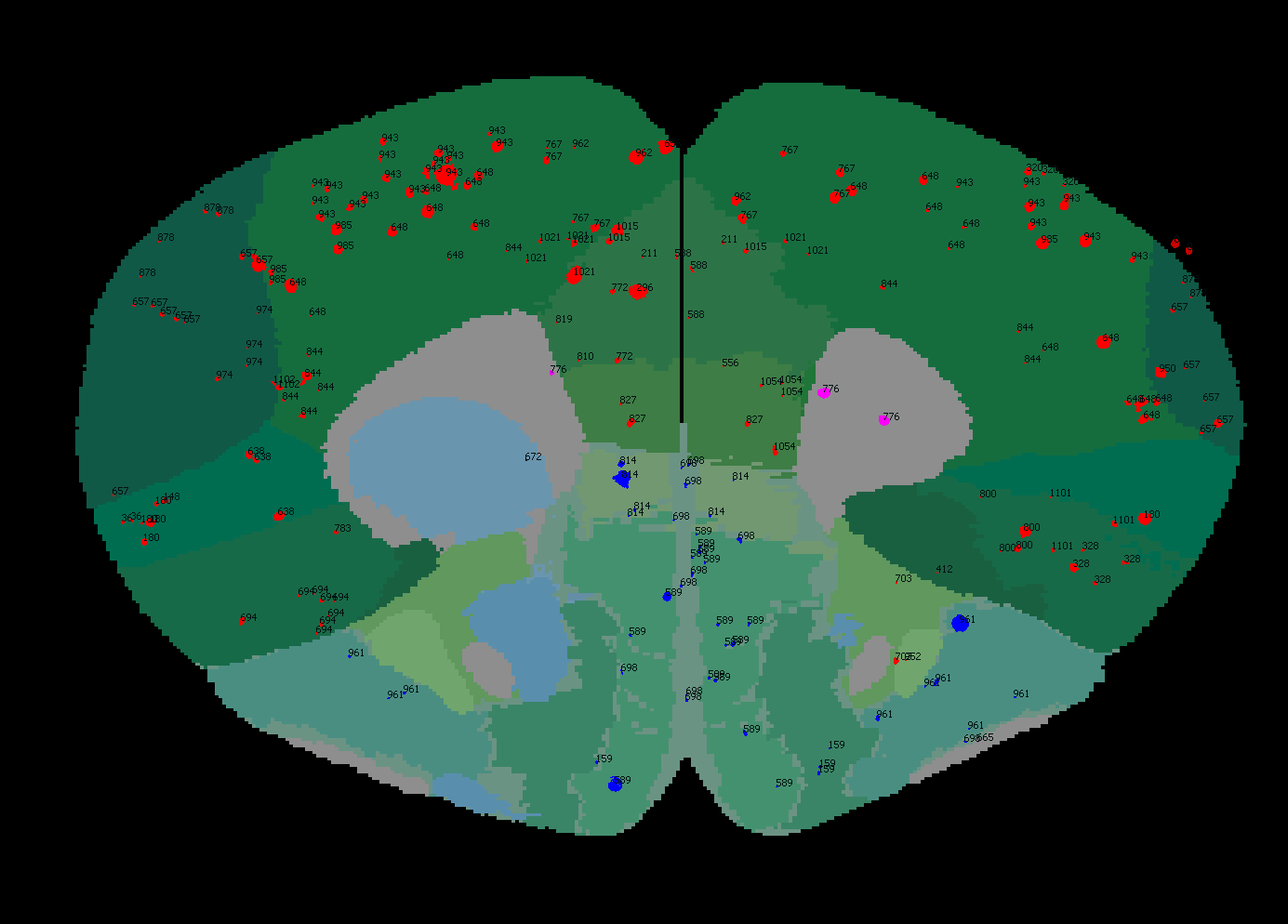

Supplement: Supplementary file 2 [file Data_Sheet_1.ZIP › Supplementary_material_Yates/pan-Abeta/tg2576_m287_4G8_s043_resize_Object Predictions.png]

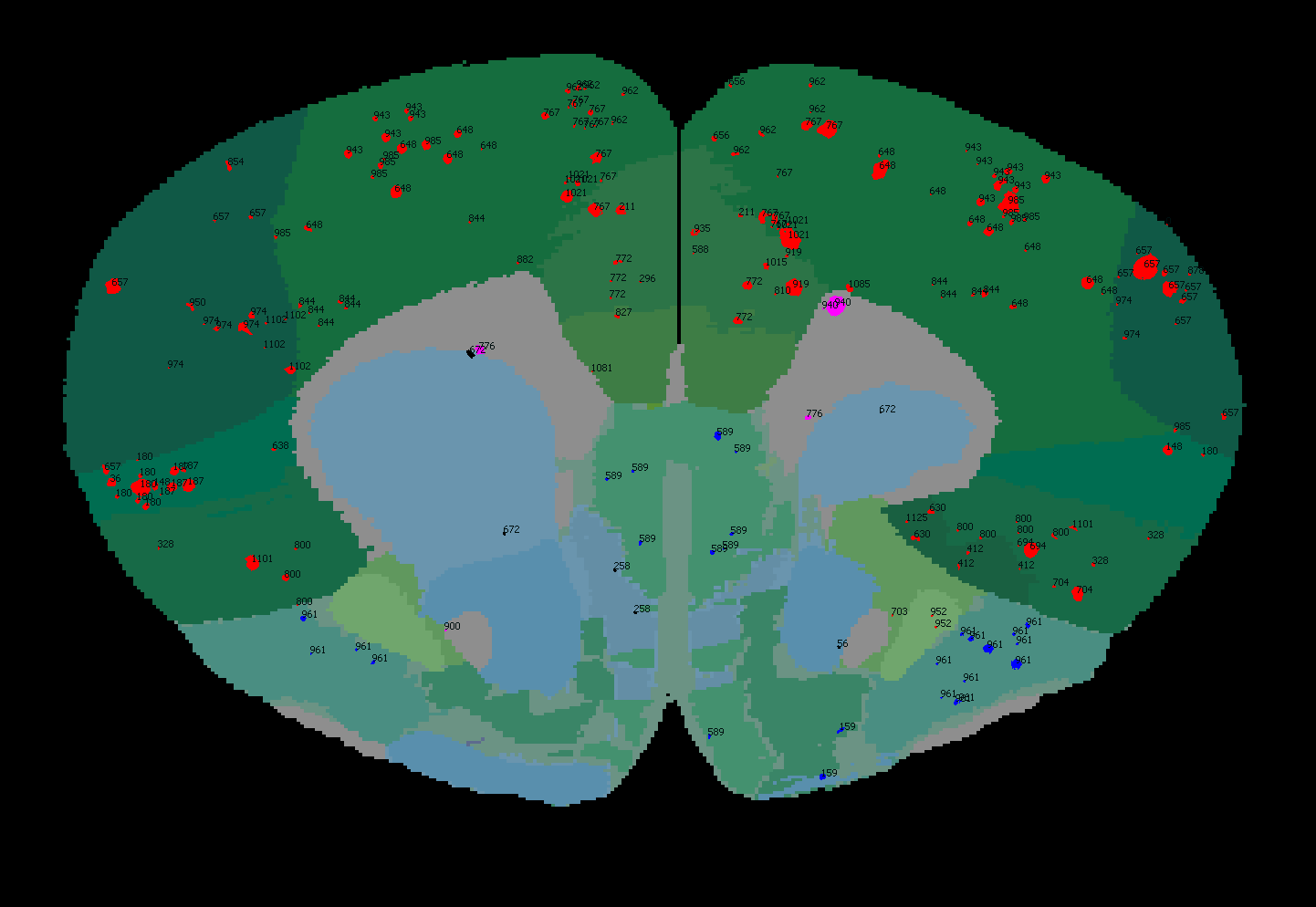

Supplement: Supplementary file 2 [file Data_Sheet_1.ZIP › Supplementary_material_Yates/pan-Abeta/tg2576_m287_4G8_s047_resize_Object Predictions.png]

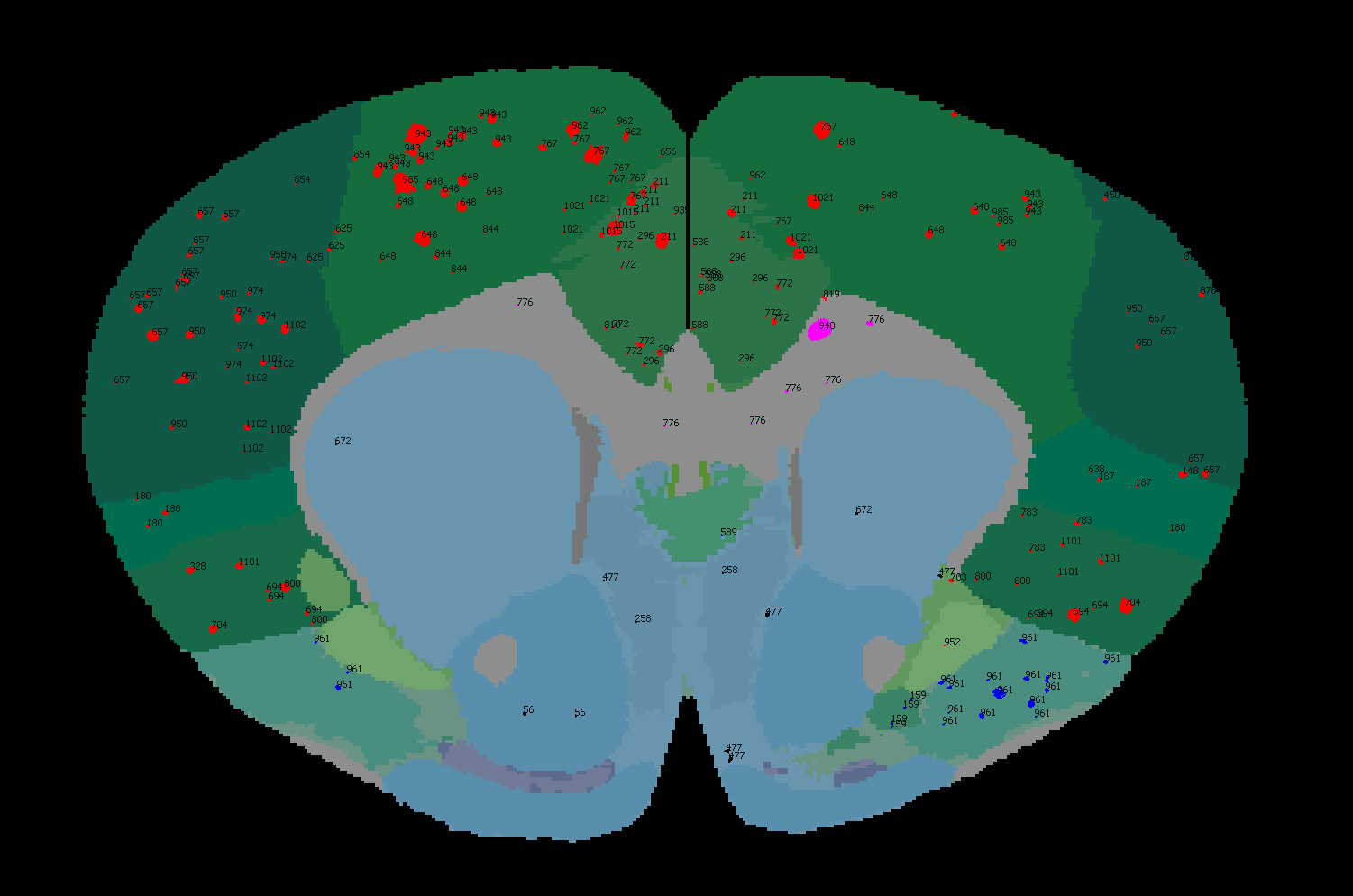

Supplement: Supplementary file 2 [file Data_Sheet_1.ZIP › Supplementary_material_Yates/pan-Abeta/tg2576_m287_4G8_s051_resize_Object Predictions.png]

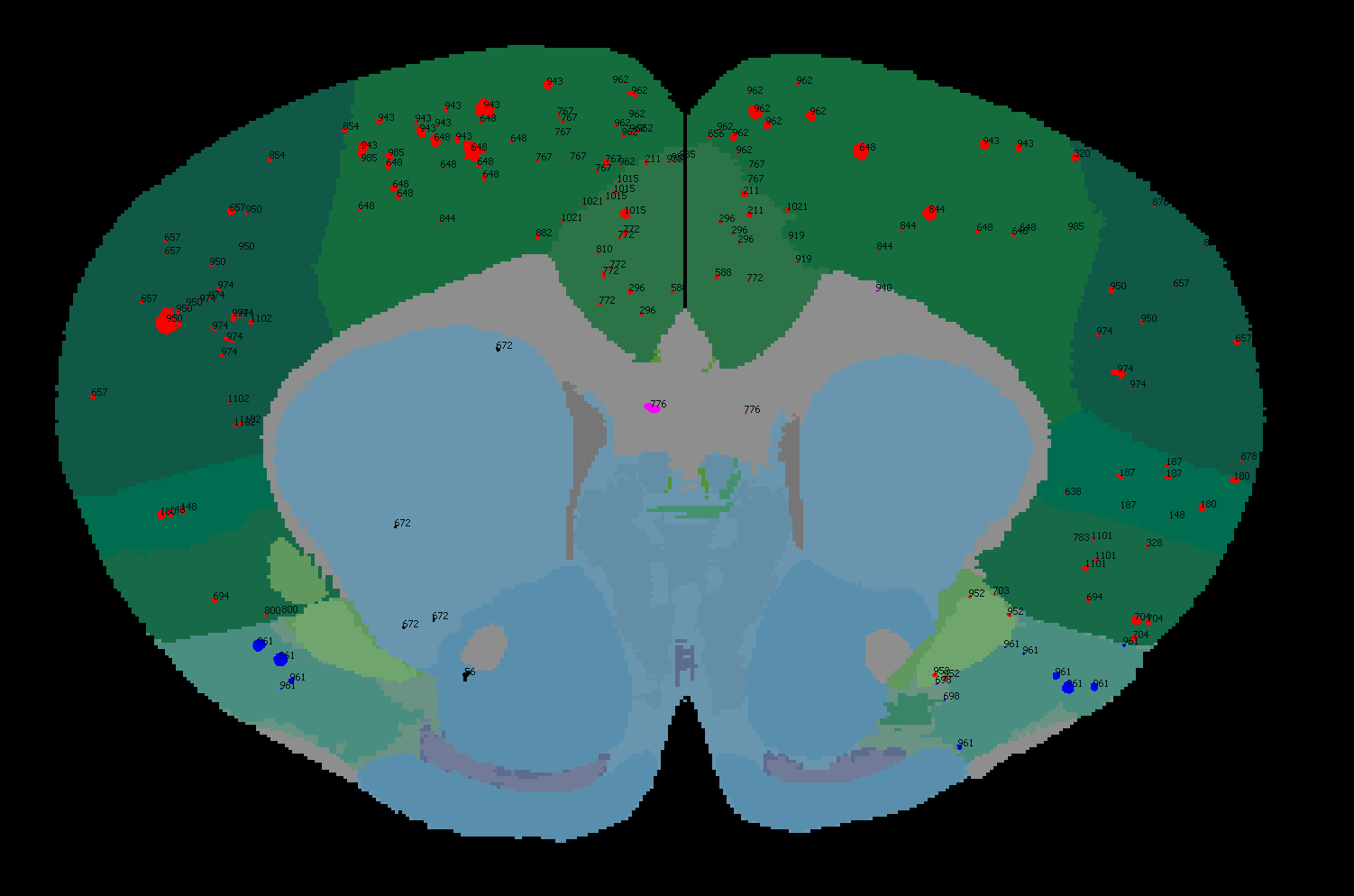

Supplement: Supplementary file 2 [file Data_Sheet_1.ZIP › Supplementary_material_Yates/pan-Abeta/tg2576_m287_4G8_s055_resize_Object Predictions.png]

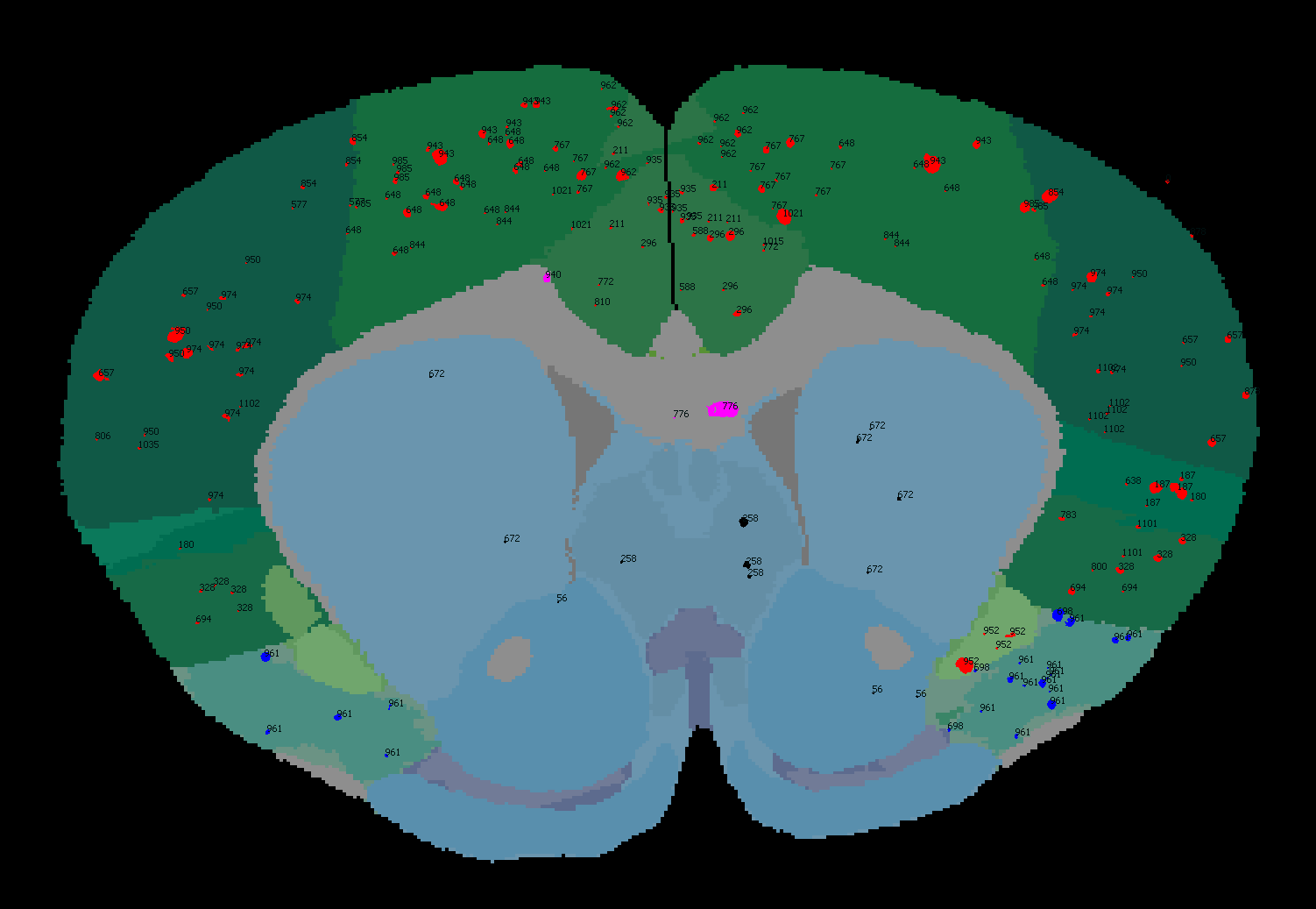

Supplement: Supplementary file 2 [file Data_Sheet_1.ZIP › Supplementary_material_Yates/pan-Abeta/tg2576_m287_4G8_s059_resize_Object Predictions.png]

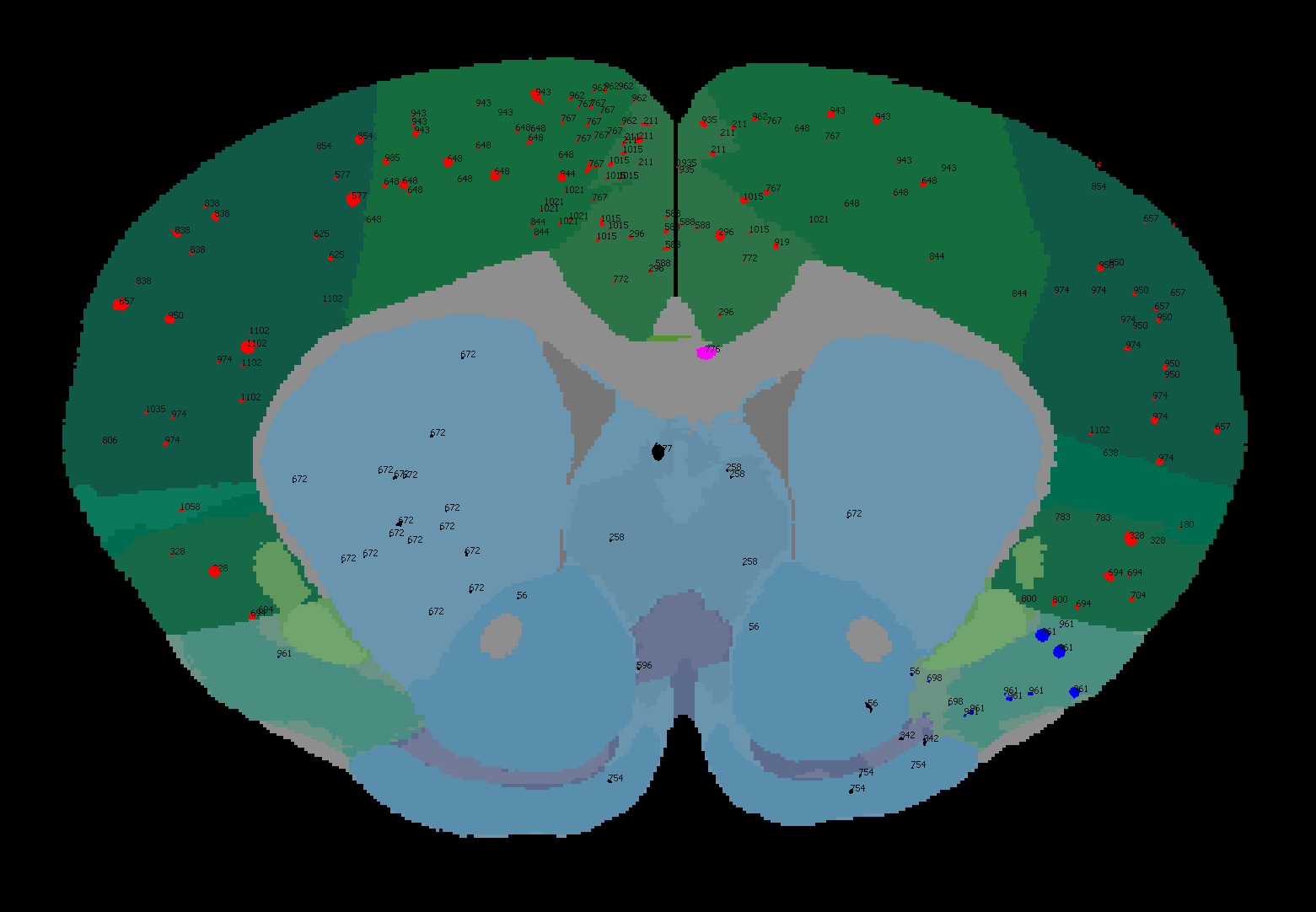

Supplement: Supplementary file 2 [file Data_Sheet_1.ZIP › Supplementary_material_Yates/pan-Abeta/tg2576_m287_4G8_s063_resize_Object Predictions.png]

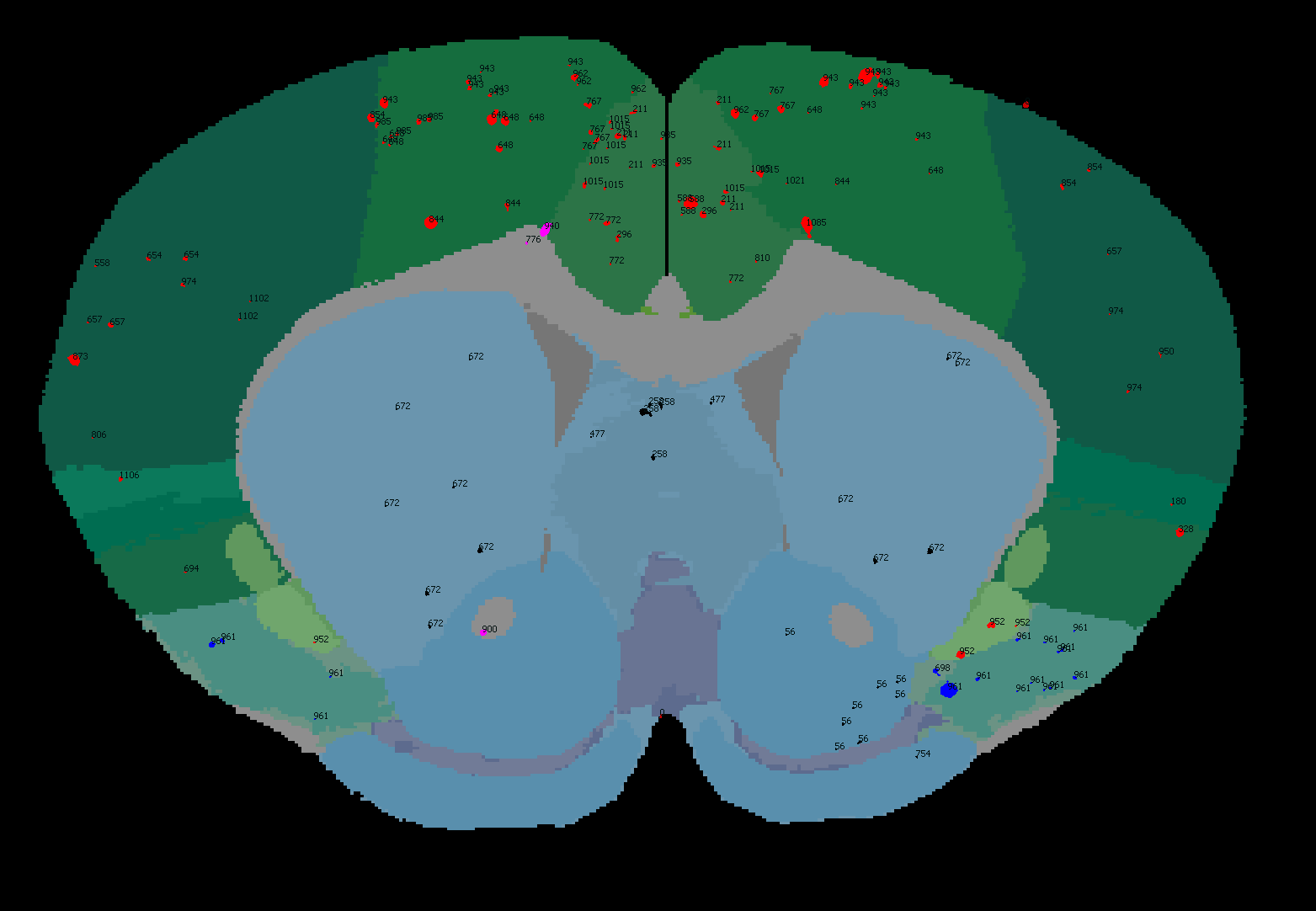

Supplement: Supplementary file 2 [file Data_Sheet_1.ZIP › Supplementary_material_Yates/pan-Abeta/tg2576_m287_4G8_s067_resize_Object Predictions.png]

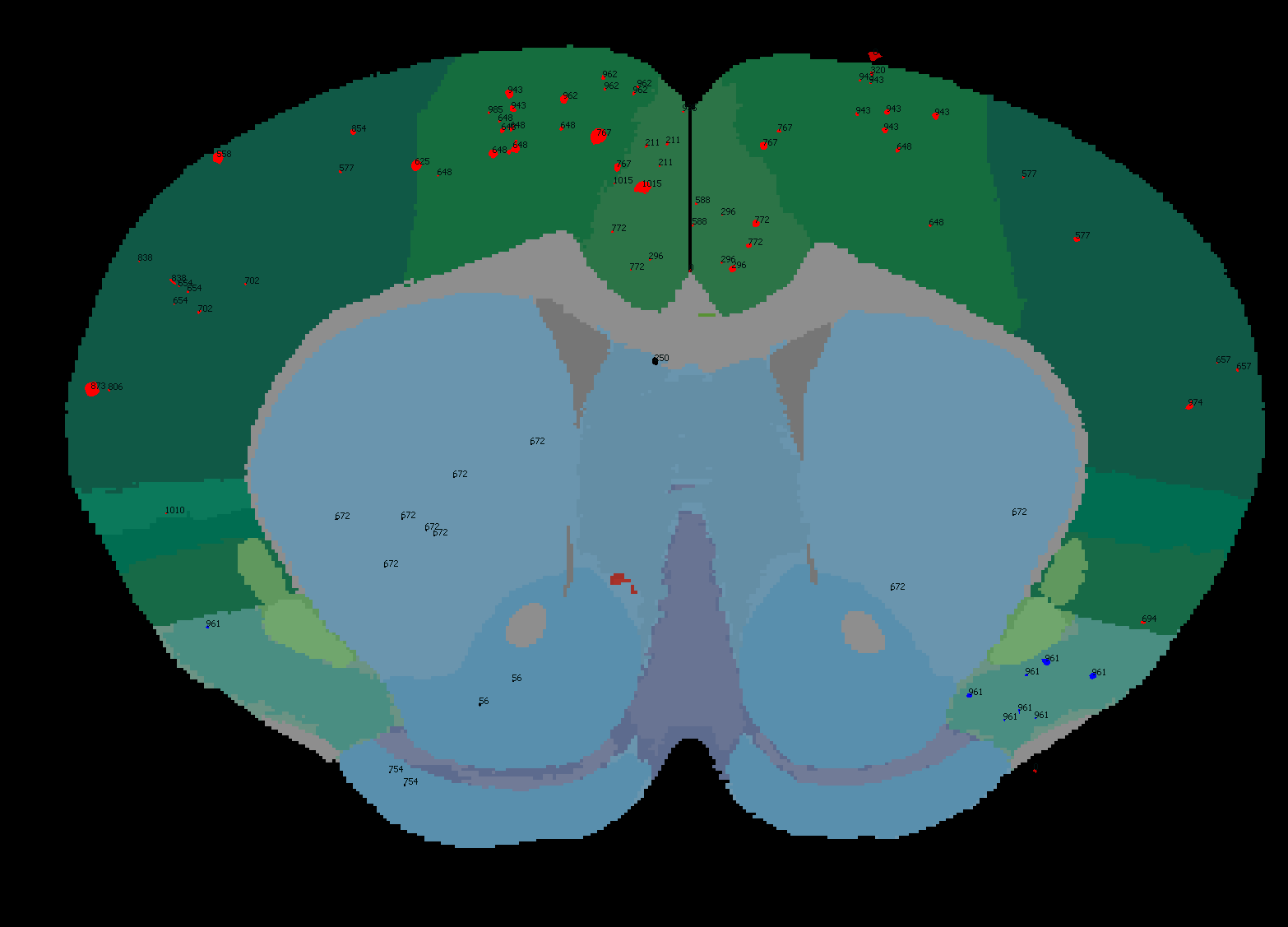

Supplement: Supplementary file 2 [file Data_Sheet_1.ZIP › Supplementary_material_Yates/pan-Abeta/tg2576_m287_4G8_s071_resize_Object Predictions.png]

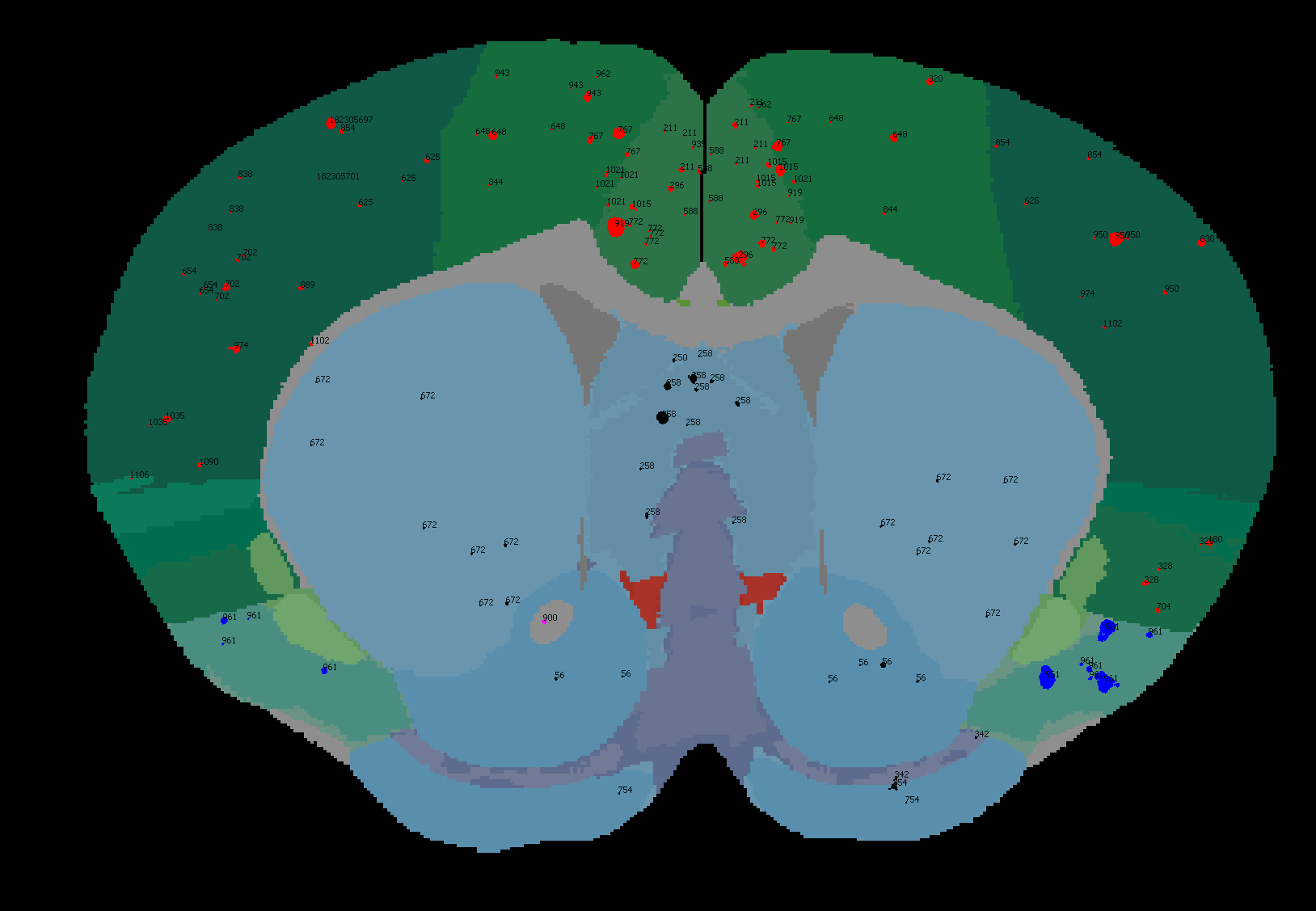

Supplement: Supplementary file 2 [file Data_Sheet_1.ZIP › Supplementary_material_Yates/pan-Abeta/tg2576_m287_4G8_s075_resize_Object Predictions.png]

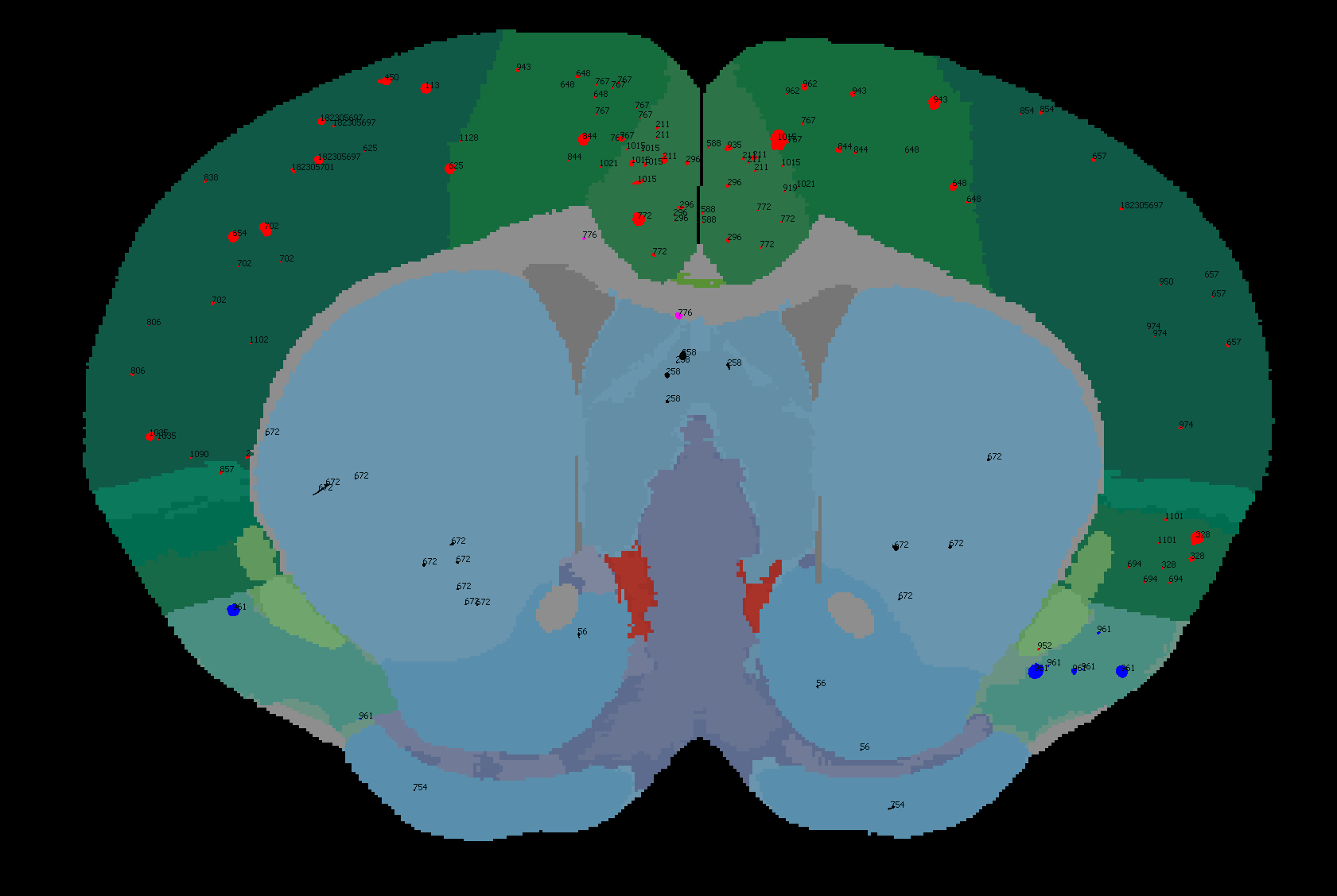

Supplement: Supplementary file 2 [file Data_Sheet_1.ZIP › Supplementary_material_Yates/pan-Abeta/tg2576_m287_4G8_s079_resize_Object Predictions.png]

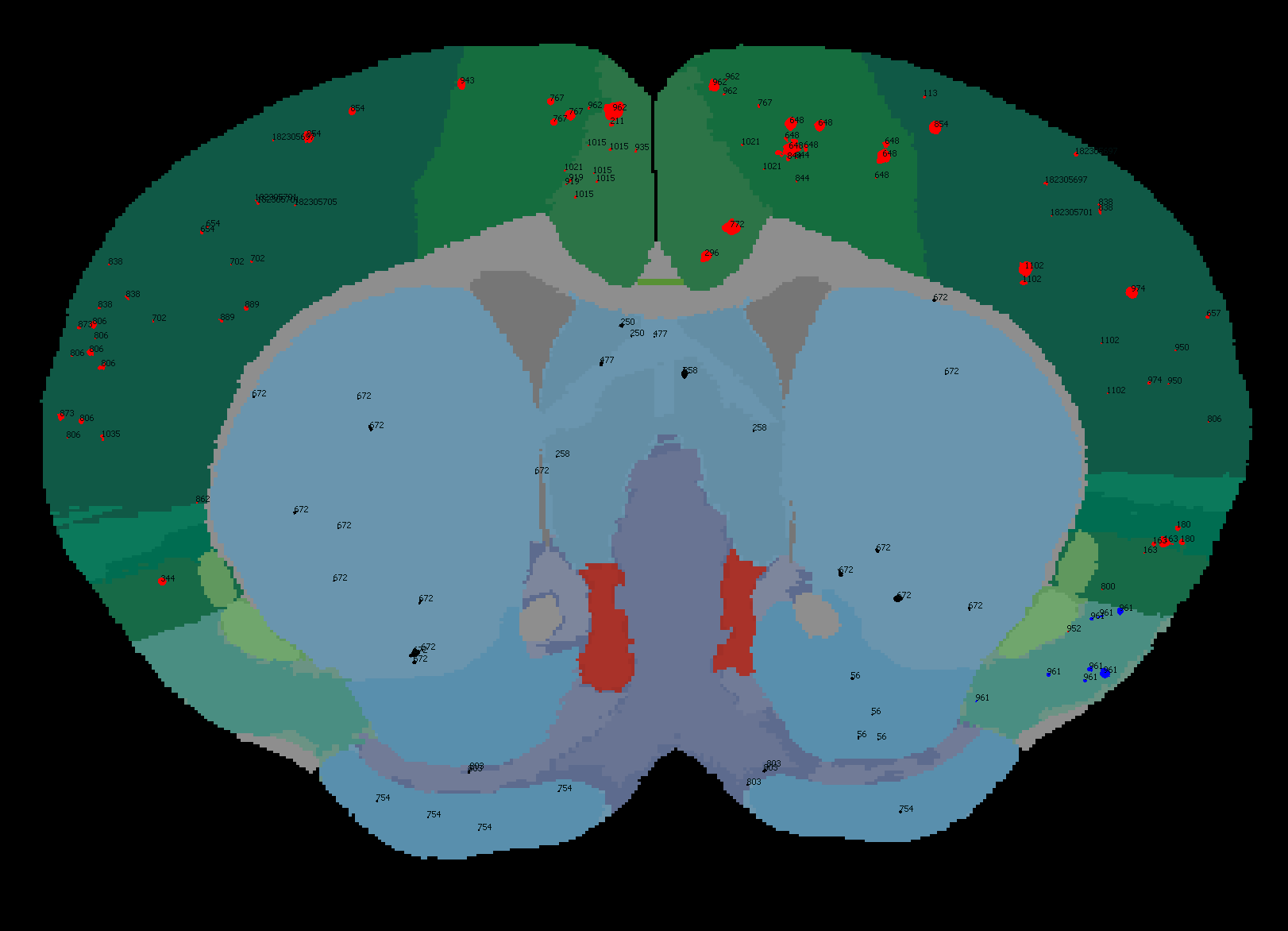

Supplement: Supplementary file 2 [file Data_Sheet_1.ZIP › Supplementary_material_Yates/pan-Abeta/tg2576_m287_4G8_s083_resize_Object Predictions.png]

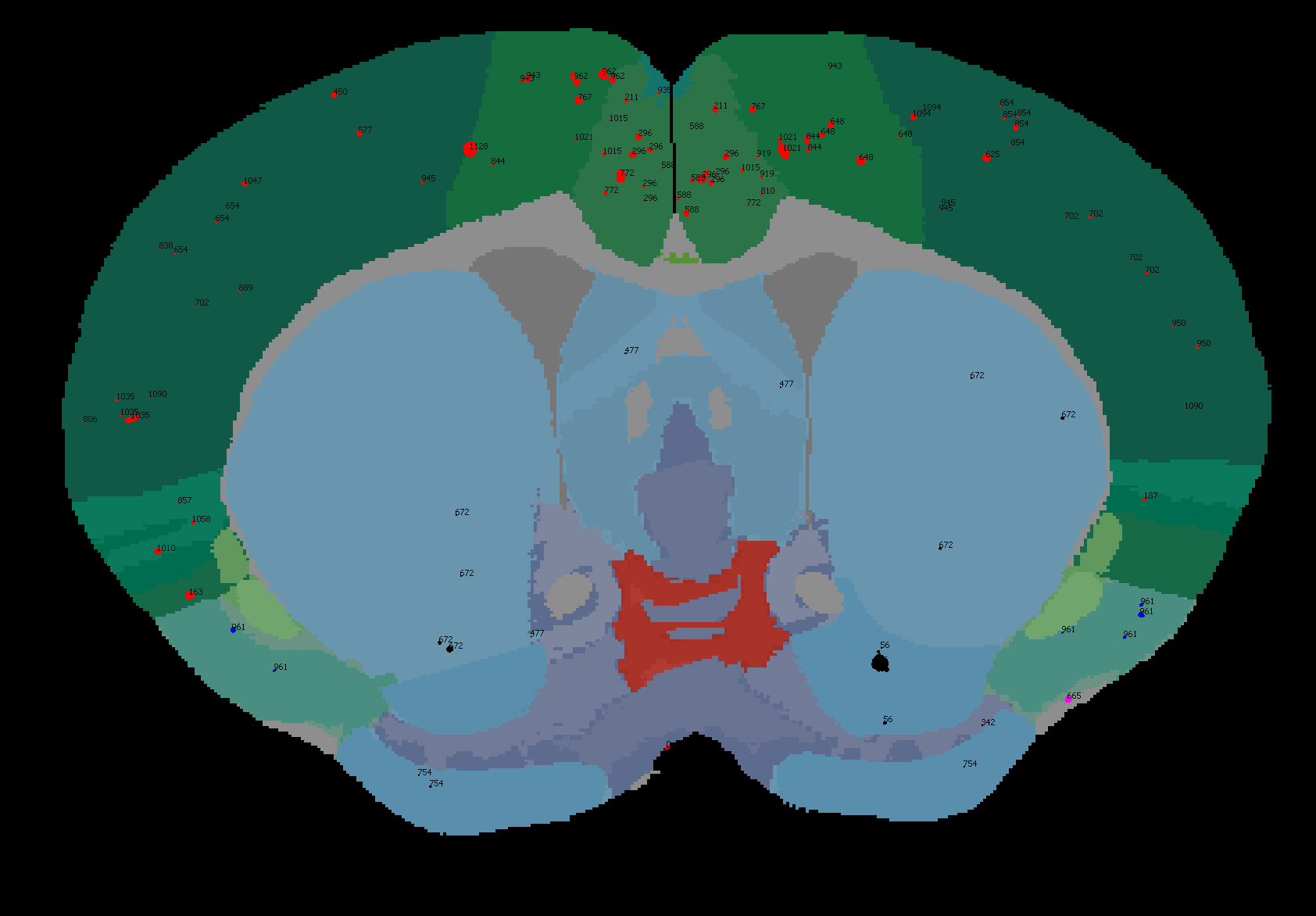

Supplement: Supplementary file 2 [file Data_Sheet_1.ZIP › Supplementary_material_Yates/pan-Abeta/tg2576_m287_4G8_s087_resize_Object Predictions.png]

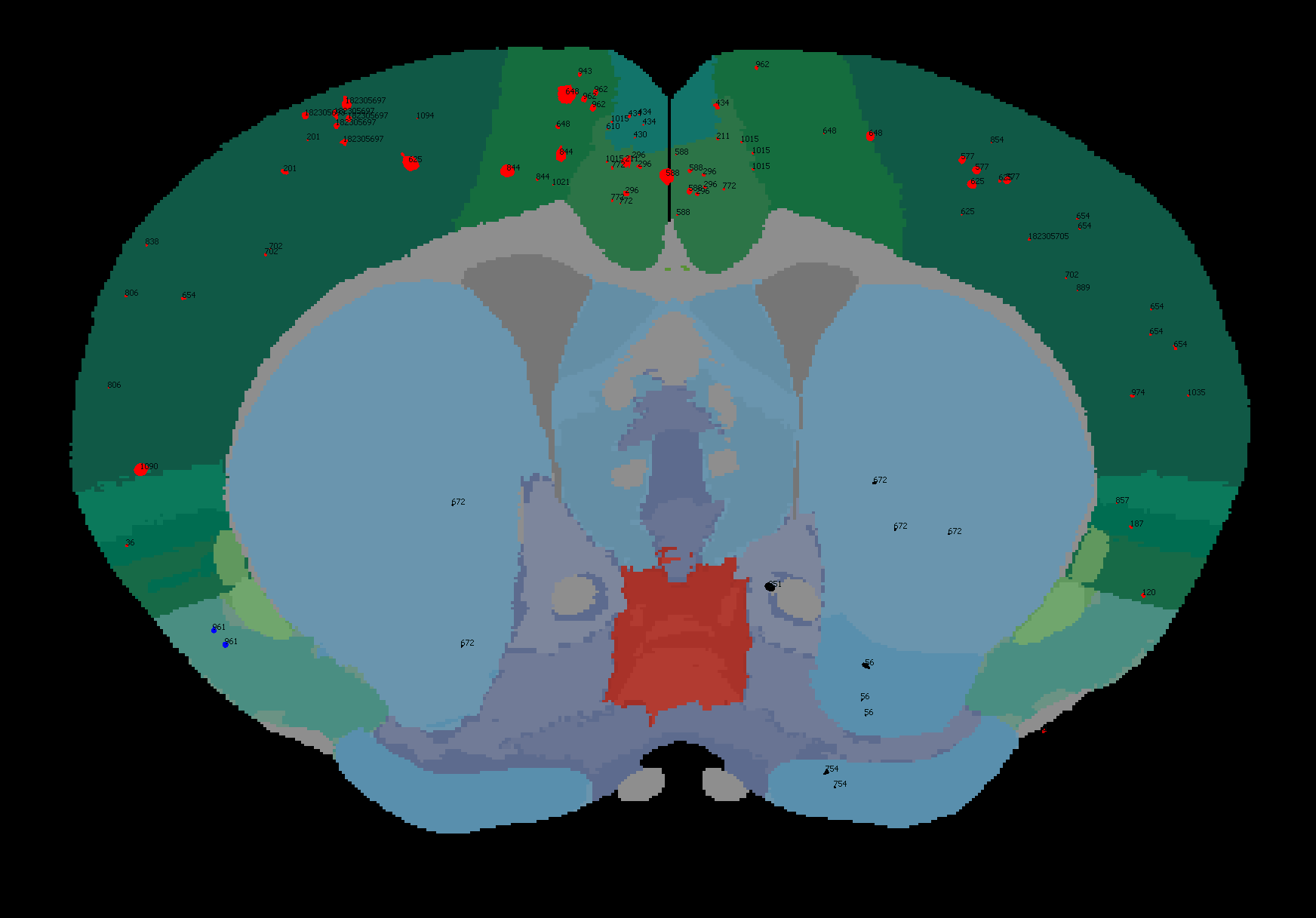

Supplement: Supplementary file 2 [file Data_Sheet_1.ZIP › Supplementary_material_Yates/pan-Abeta/tg2576_m287_4G8_s091_resize_Object Predictions.png]

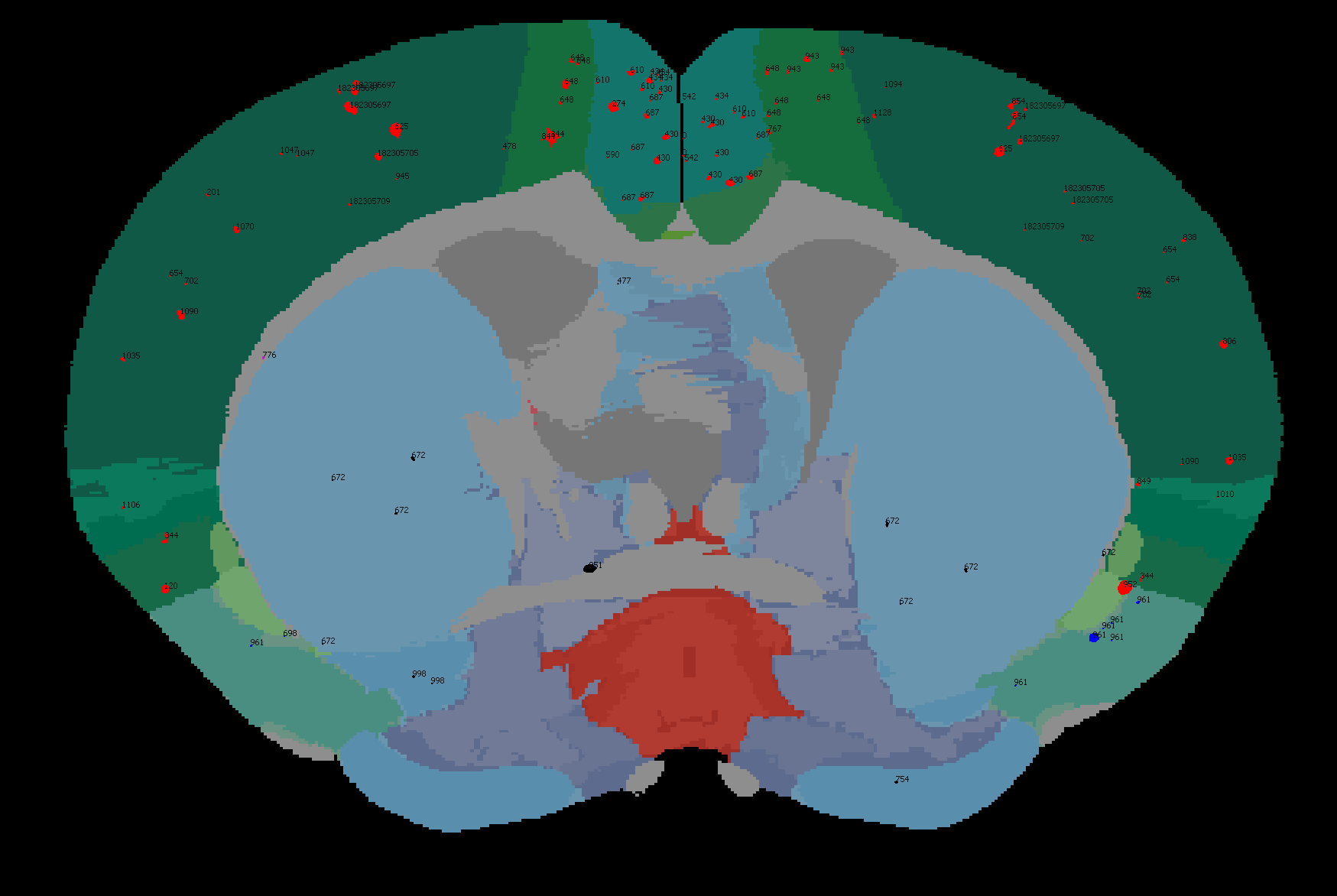

Supplement: Supplementary file 2 [file Data_Sheet_1.ZIP › Supplementary_material_Yates/pan-Abeta/tg2576_m287_4G8_s095_resize_Object Predictions.png]

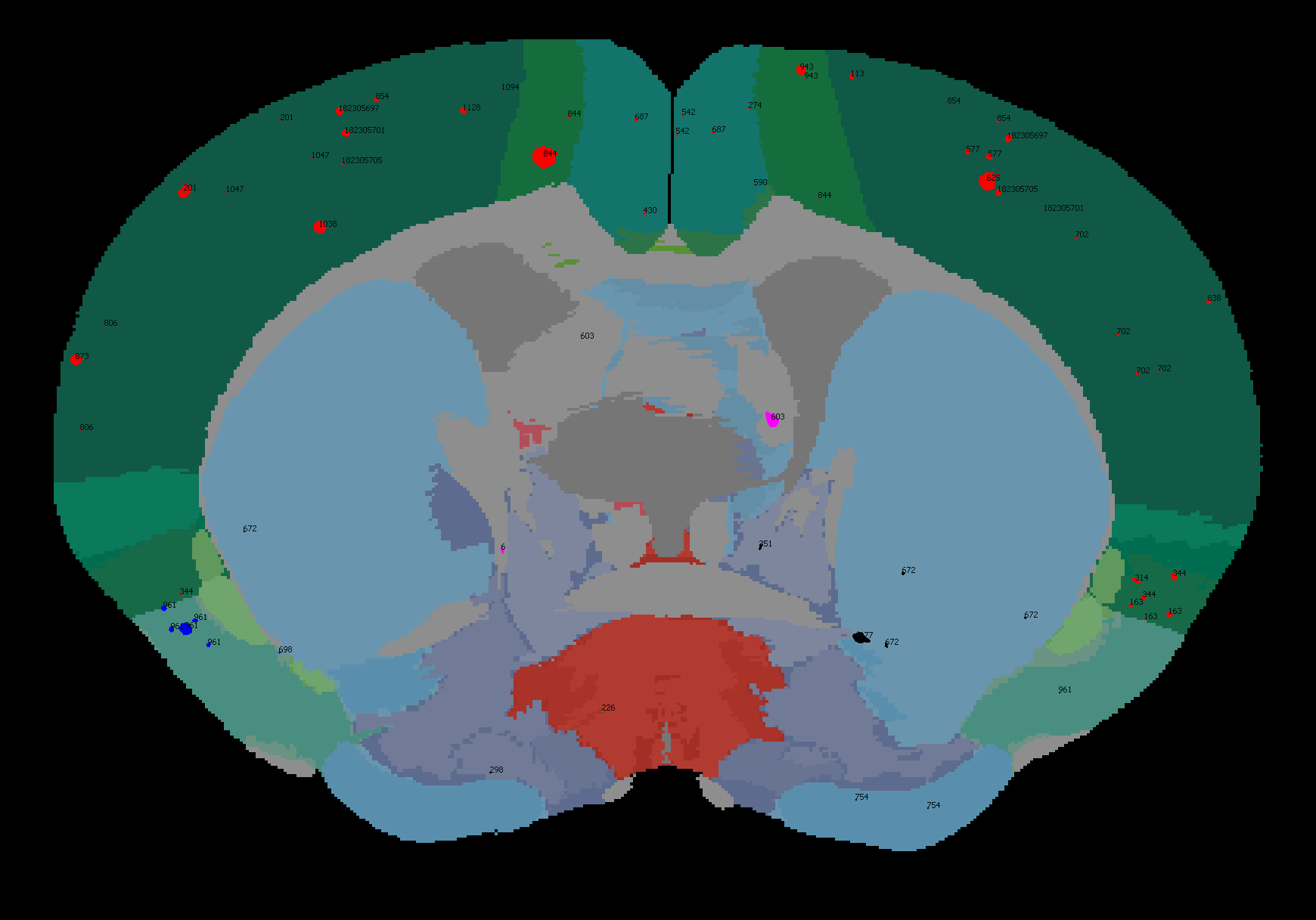

Supplement: Supplementary file 2 [file Data_Sheet_1.ZIP › Supplementary_material_Yates/pan-Abeta/tg2576_m287_4G8_s099_resize_Object Predictions.png]

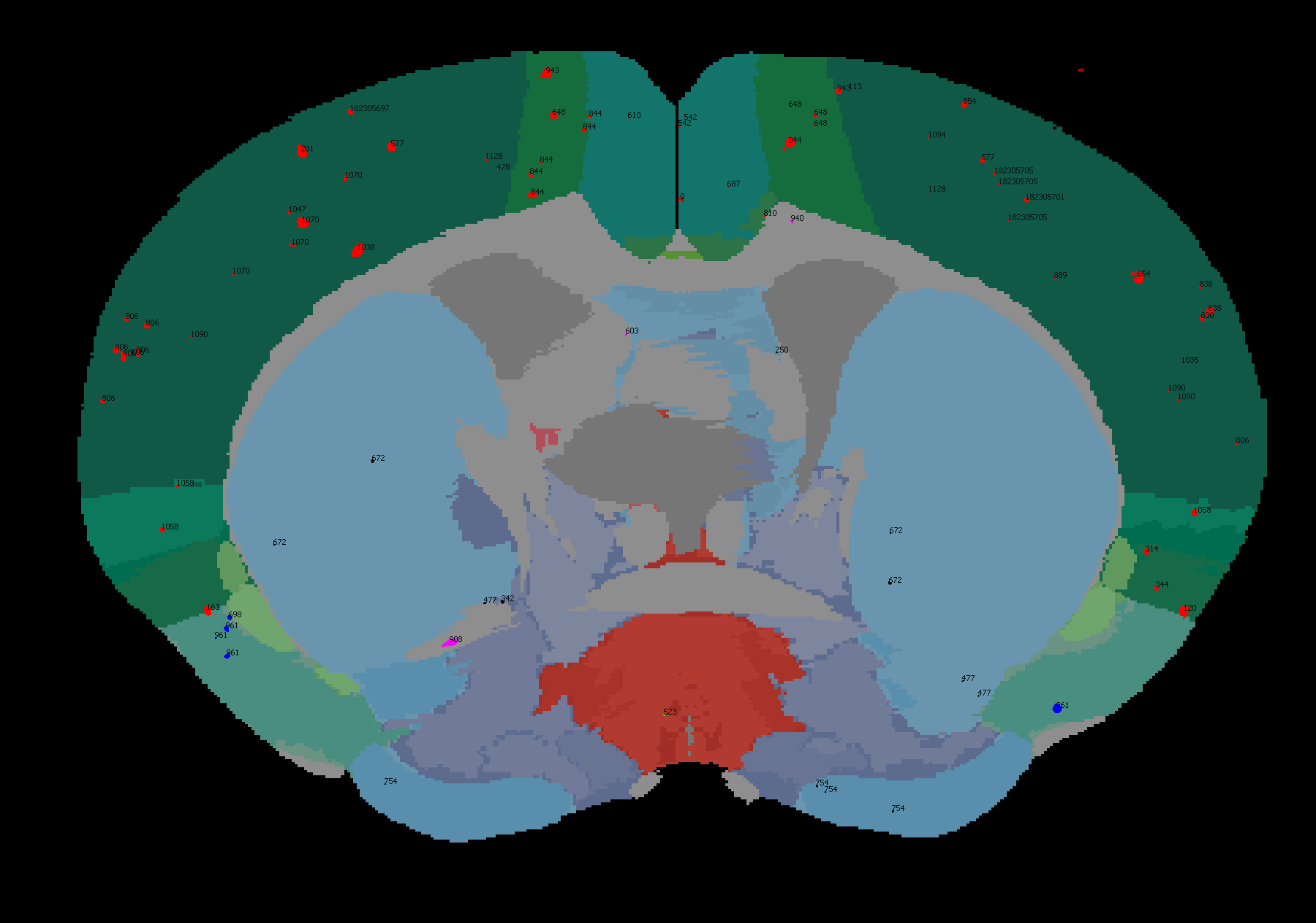

Supplement: Supplementary file 2 [file Data_Sheet_1.ZIP › Supplementary_material_Yates/pan-Abeta/tg2576_m287_4G8_s103_resize_Object Predictions.png]

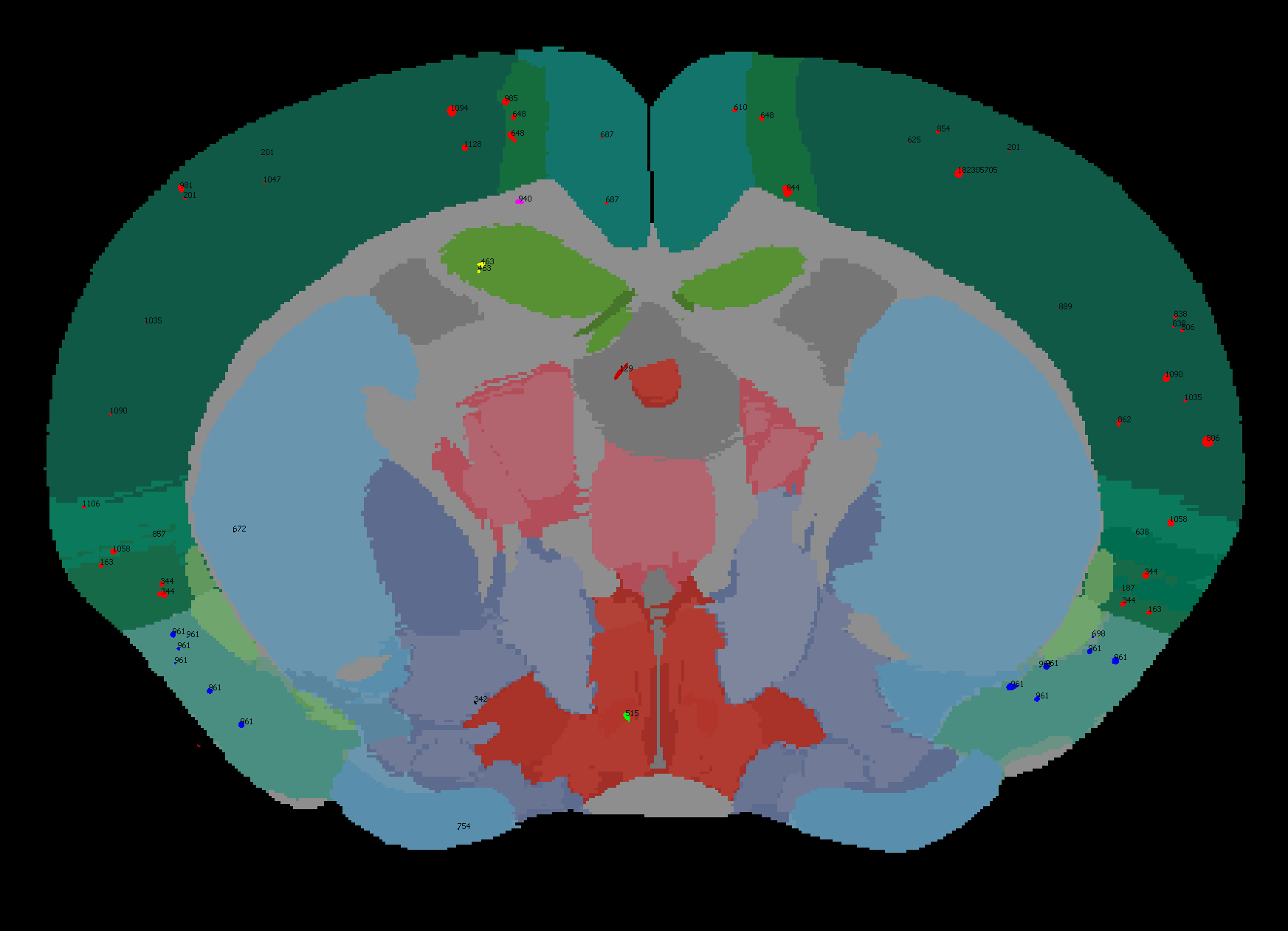

Supplement: Supplementary file 2 [file Data_Sheet_1.ZIP › Supplementary_material_Yates/pan-Abeta/tg2576_m287_4G8_s107_resize_Object Predictions.png]

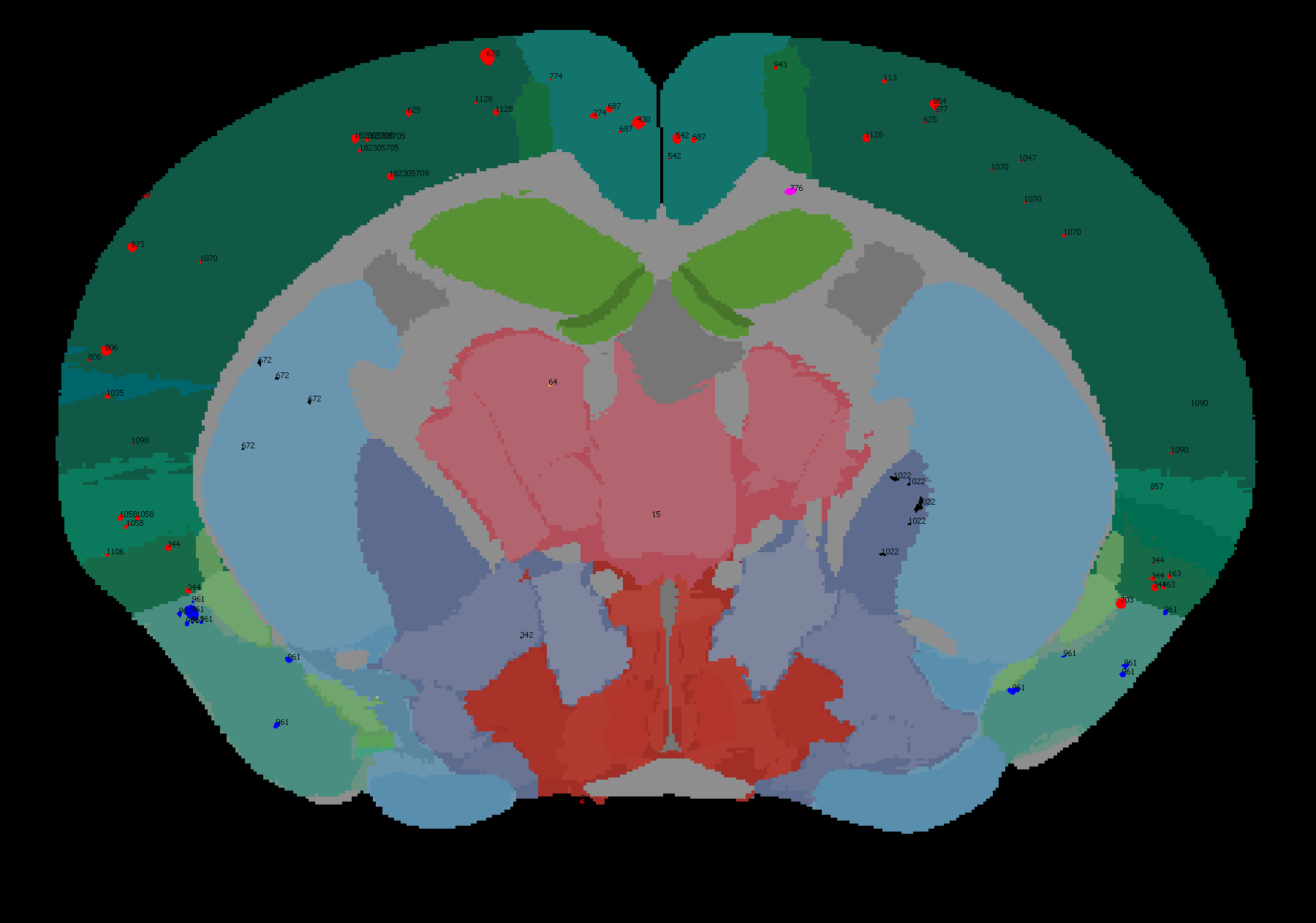

Supplement: Supplementary file 2 [file Data_Sheet_1.ZIP › Supplementary_material_Yates/pan-Abeta/tg2576_m287_4G8_s111_resize_Object Predictions.png]

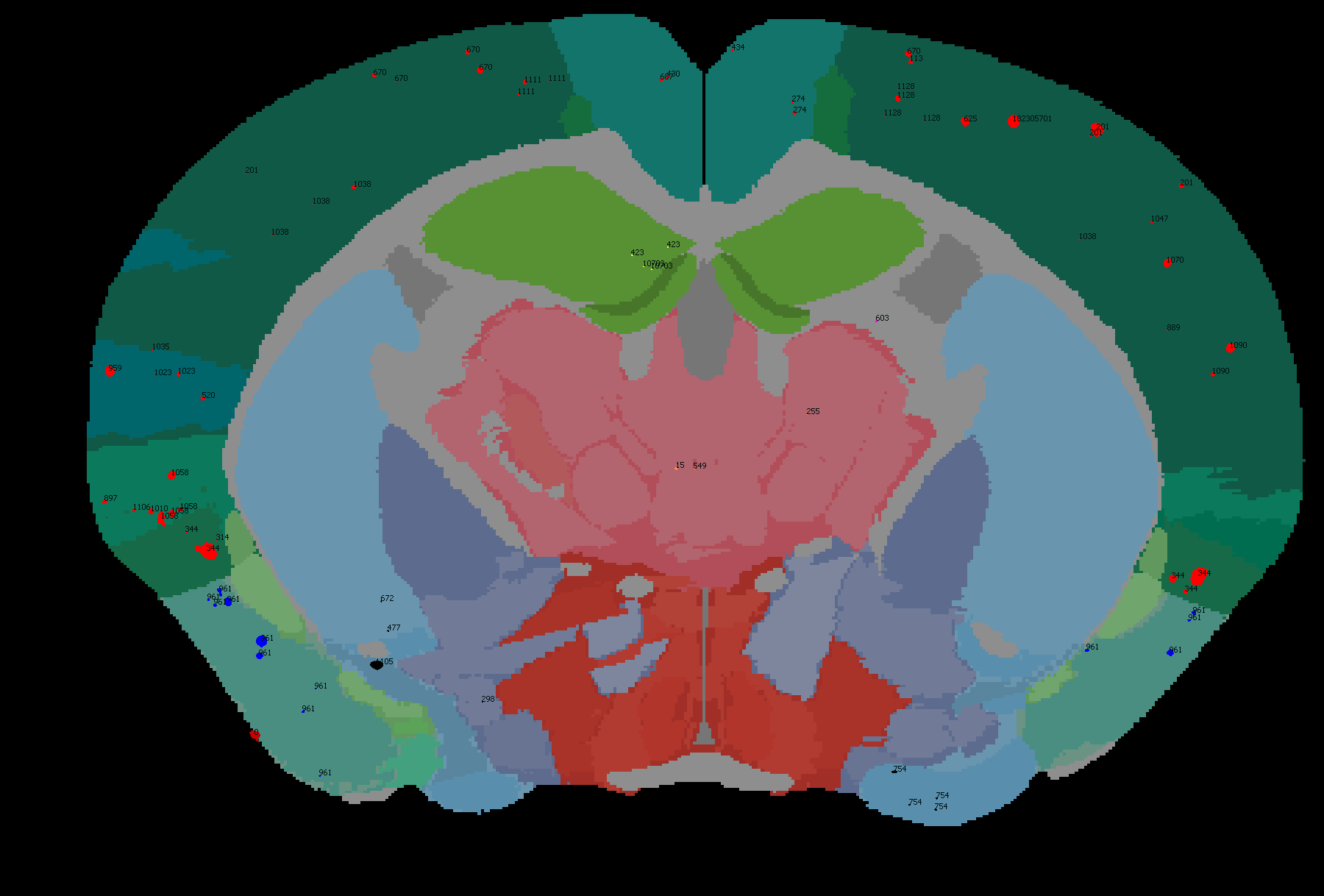

Supplement: Supplementary file 2 [file Data_Sheet_1.ZIP › Supplementary_material_Yates/pan-Abeta/tg2576_m287_4G8_s115_resize_Object Predictions.png]

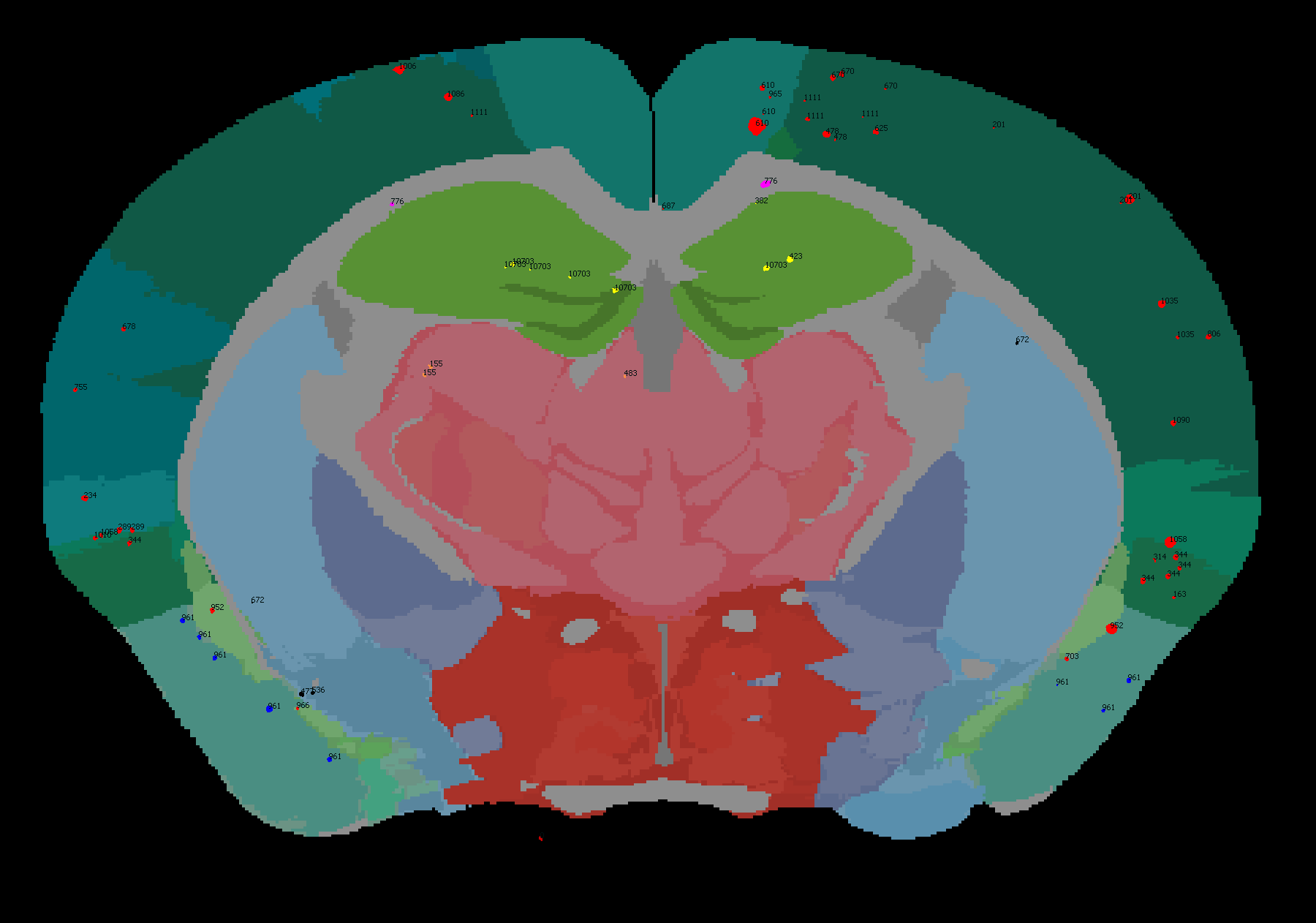

Supplement: Supplementary file 2 [file Data_Sheet_1.ZIP › Supplementary_material_Yates/pan-Abeta/tg2576_m287_4G8_s119_resize_Object Predictions.png]

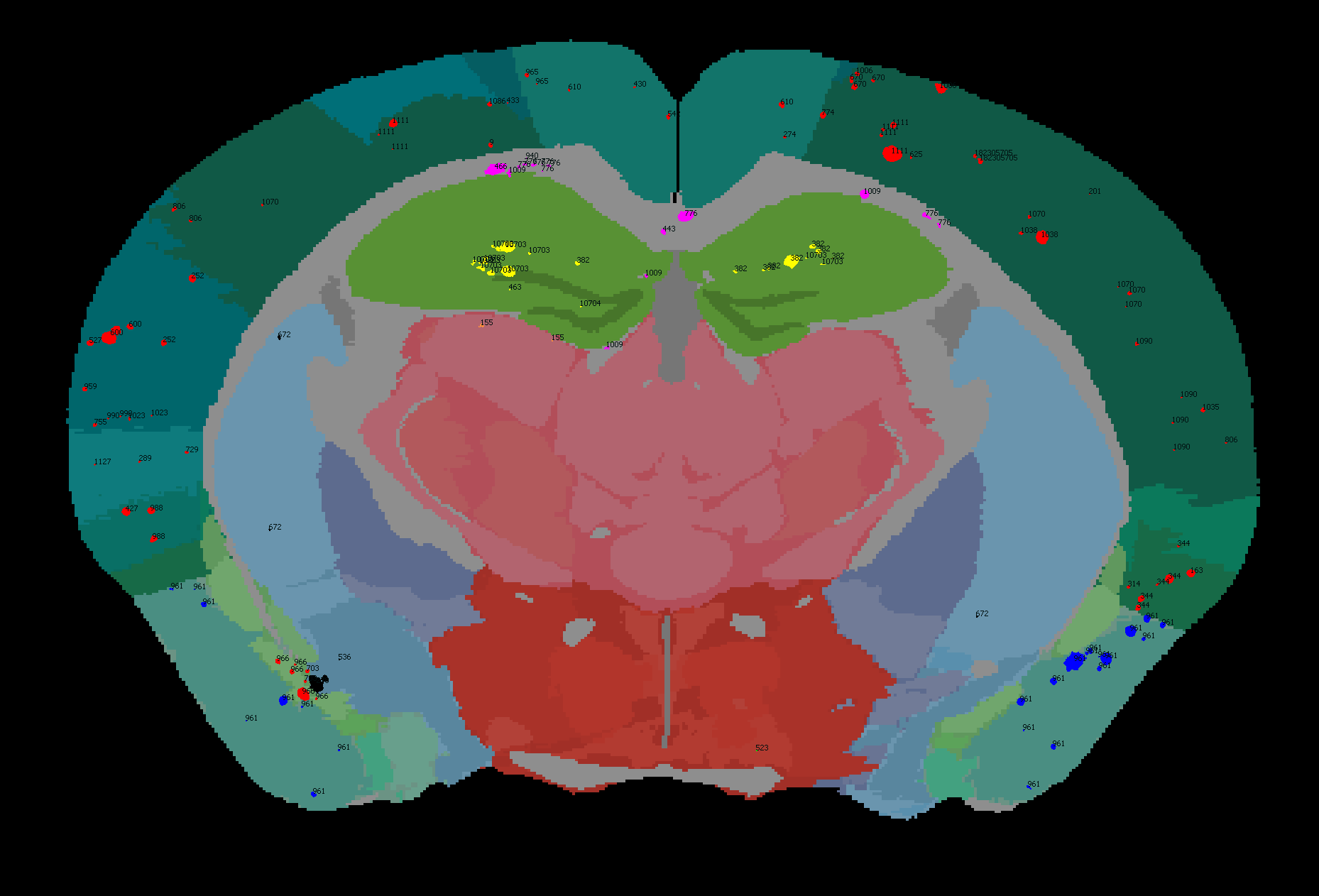

Supplement: Supplementary file 2 [file Data_Sheet_1.ZIP › Supplementary_material_Yates/pan-Abeta/tg2576_m287_4G8_s123_resize_Object Predictions.png]

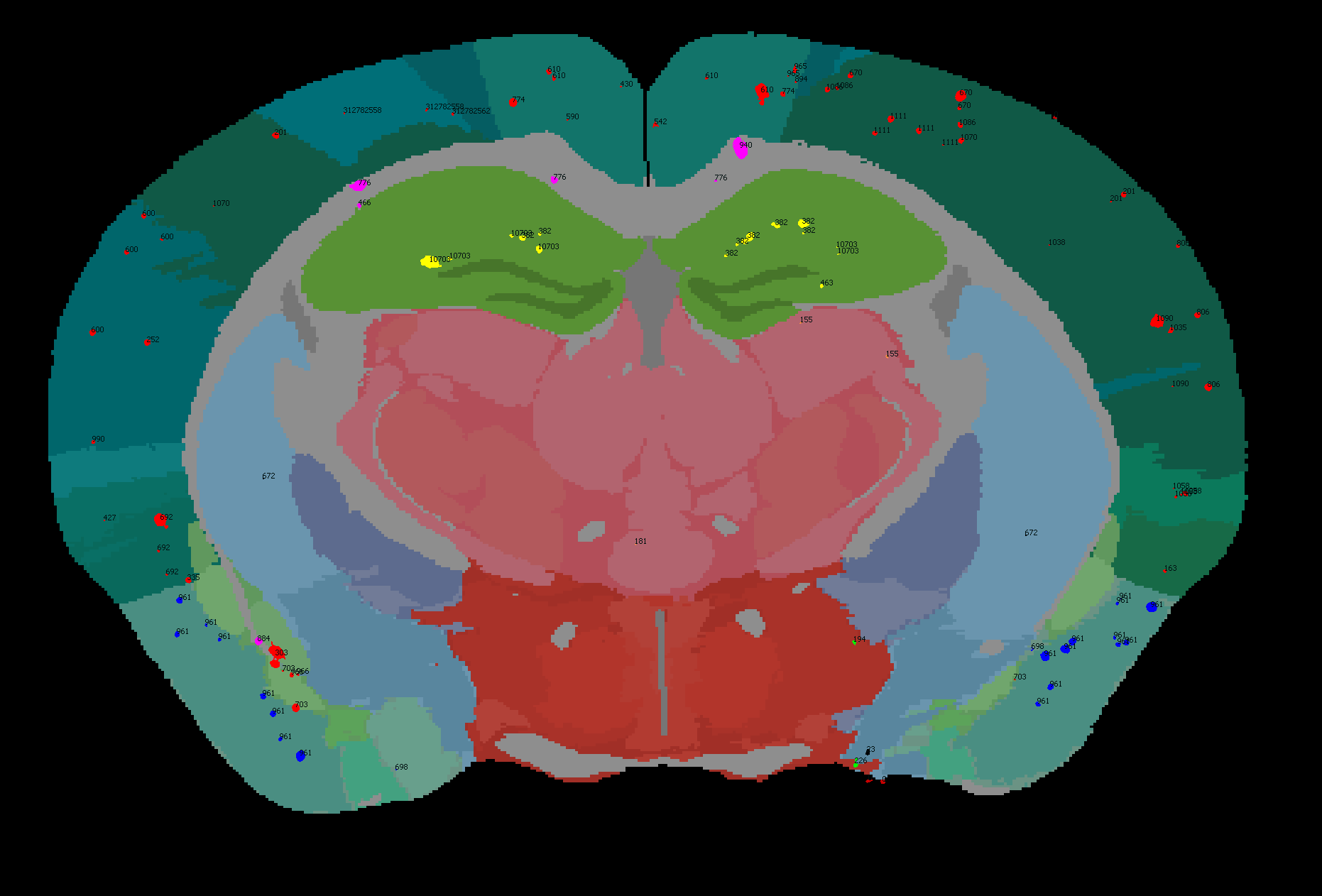

Supplement: Supplementary file 2 [file Data_Sheet_1.ZIP › Supplementary_material_Yates/pan-Abeta/tg2576_m287_4G8_s127_resize_Object Predictions.png]

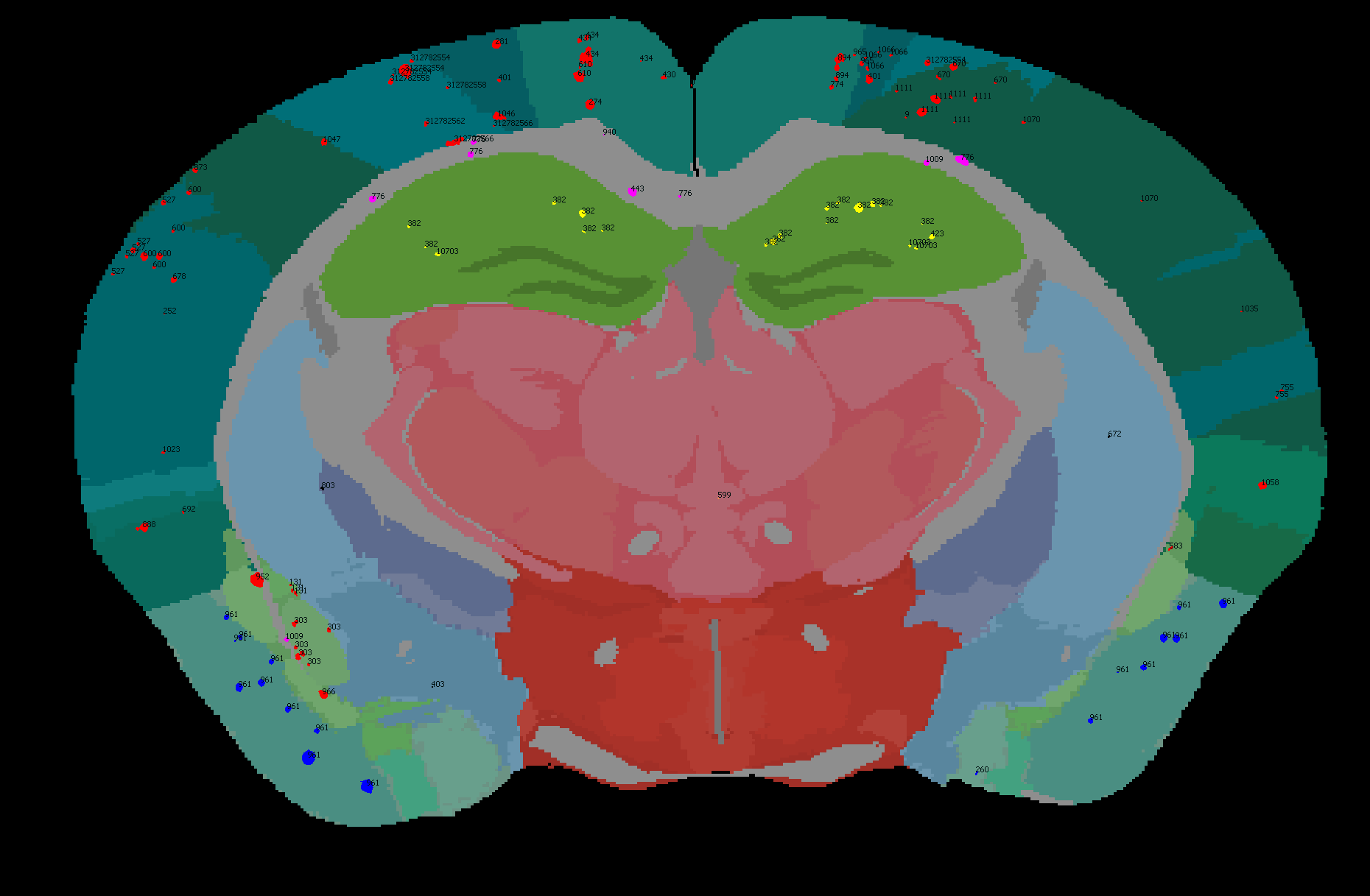

Supplement: Supplementary file 2 [file Data_Sheet_1.ZIP › Supplementary_material_Yates/pan-Abeta/tg2576_m287_4G8_s131_resize_Object Predictions.png]

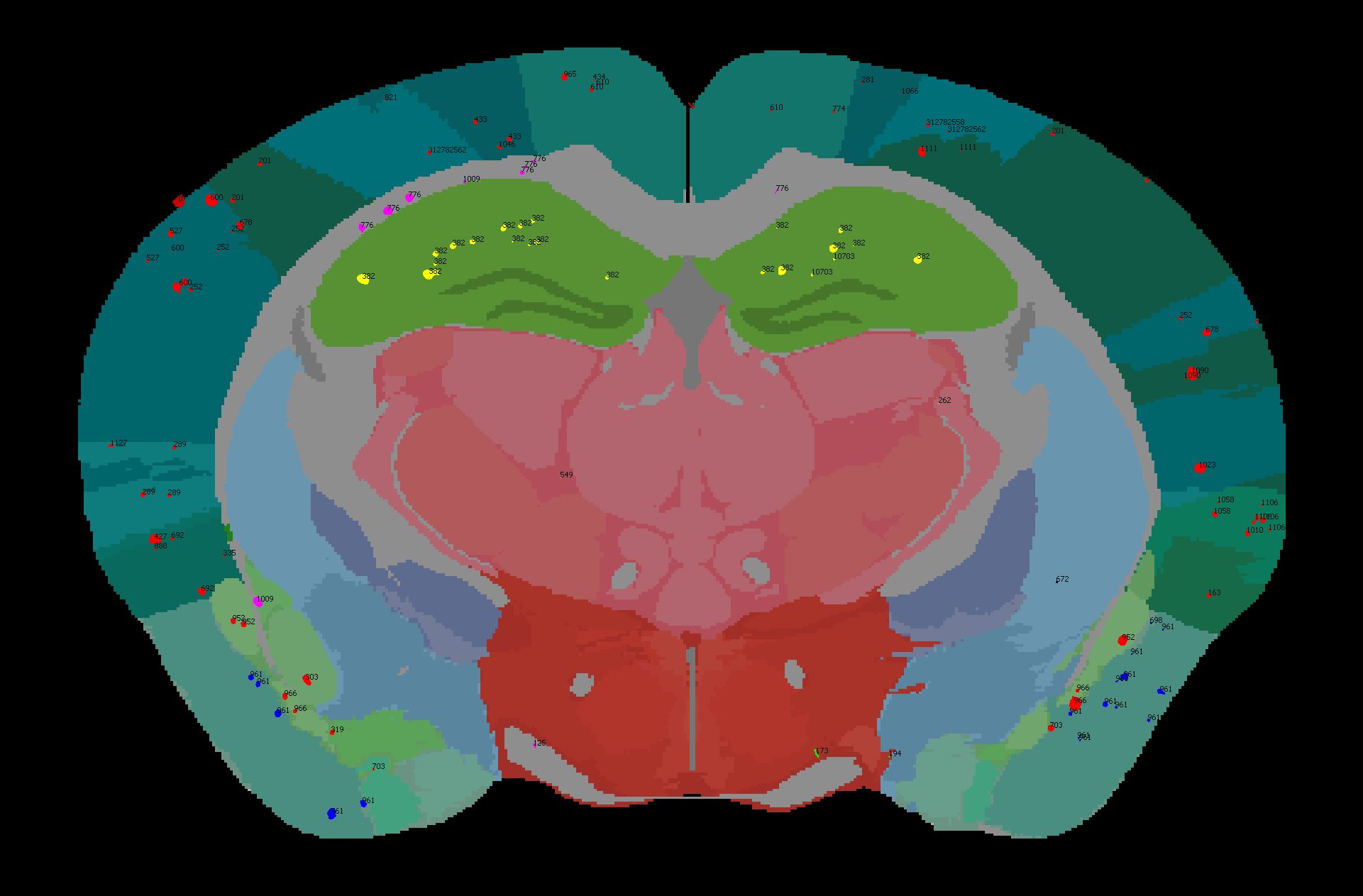

Supplement: Supplementary file 2 [file Data_Sheet_1.ZIP › Supplementary_material_Yates/pan-Abeta/tg2576_m287_4G8_s135_resize_Object Predictions.png]

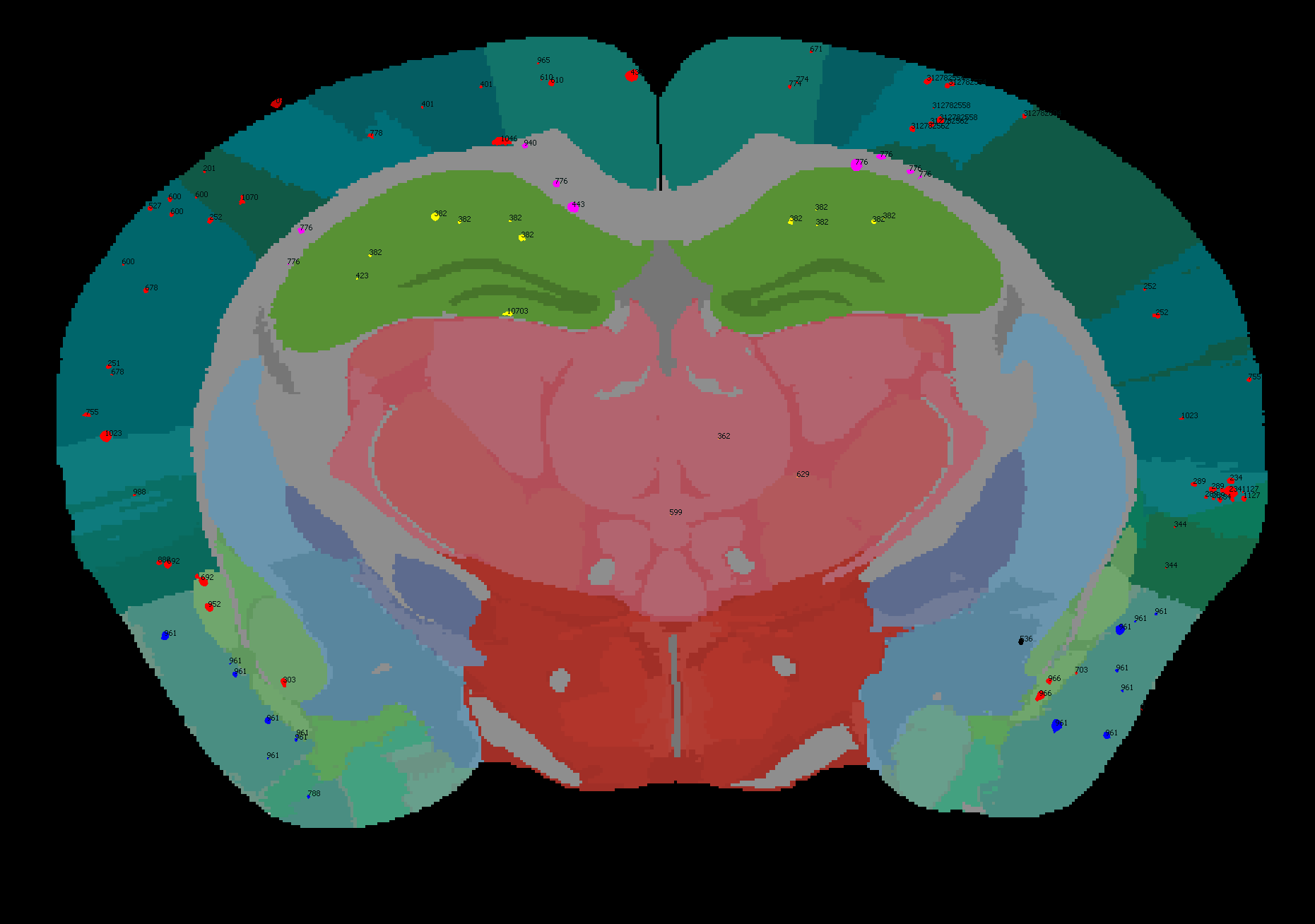

Supplement: Supplementary file 2 [file Data_Sheet_1.ZIP › Supplementary_material_Yates/pan-Abeta/tg2576_m287_4G8_s139_resize_Object Predictions.png]

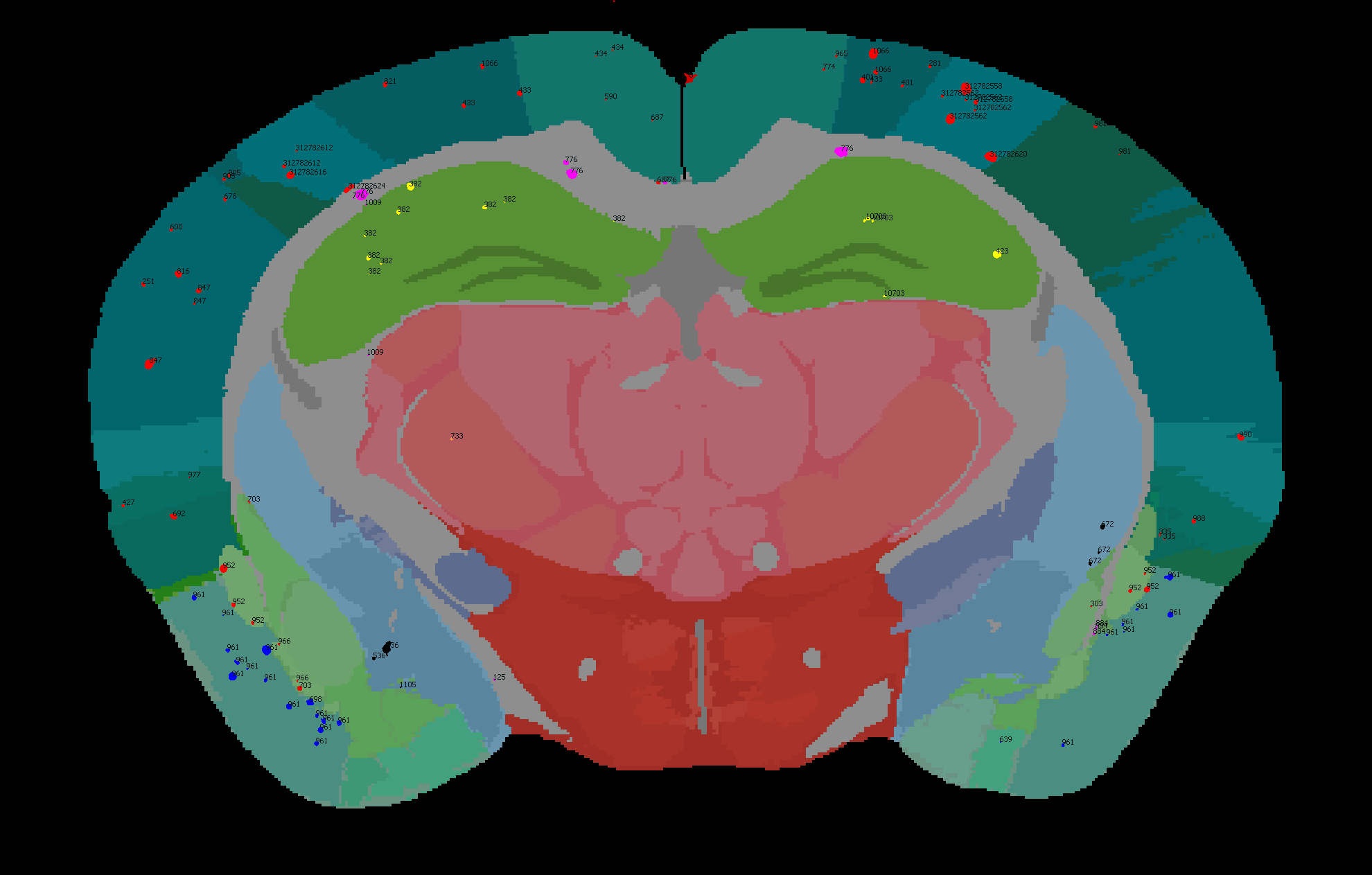

Supplement: Supplementary file 2 [file Data_Sheet_1.ZIP › Supplementary_material_Yates/pan-Abeta/tg2576_m287_4G8_s143_resize_Object Predictions.png]

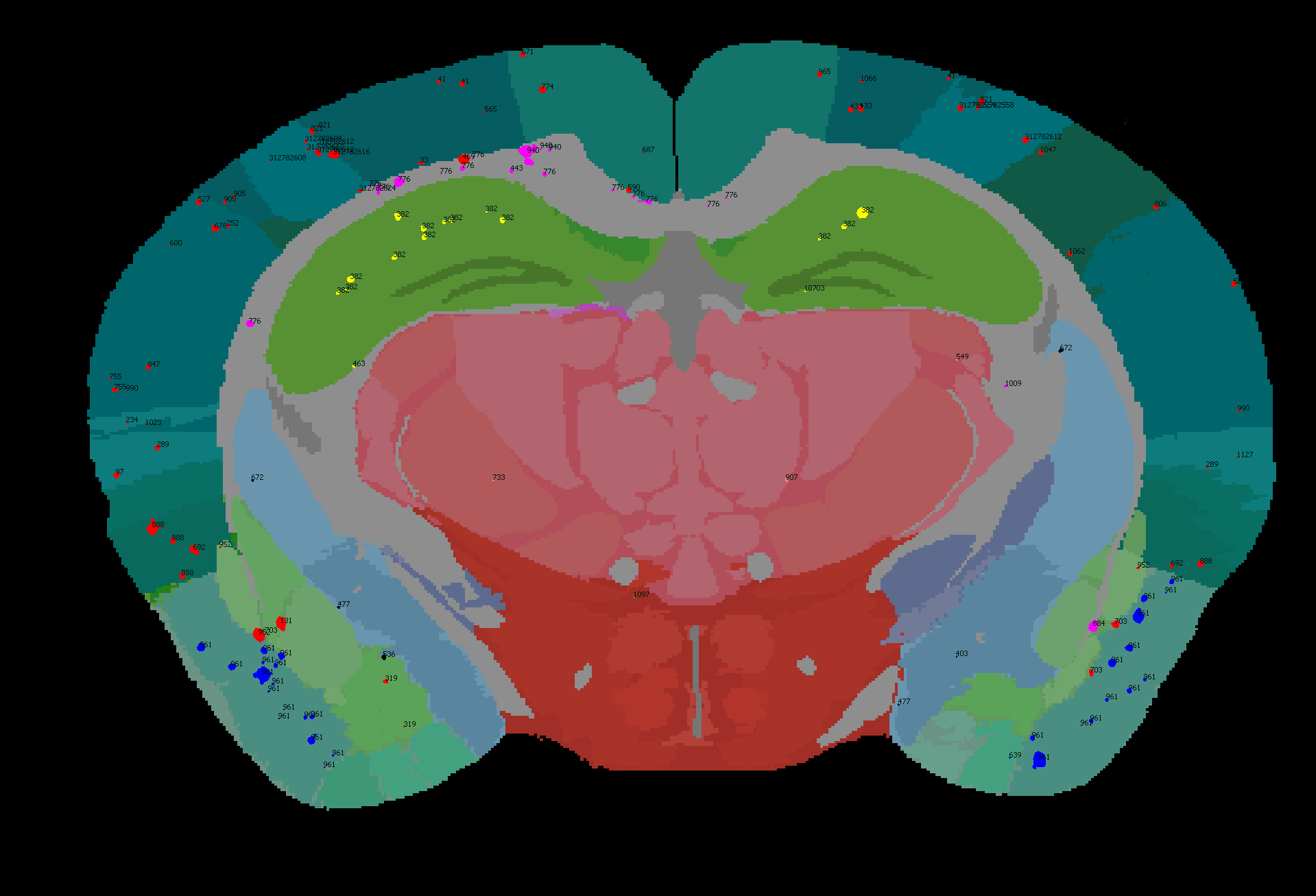

Supplement: Supplementary file 2 [file Data_Sheet_1.ZIP › Supplementary_material_Yates/pan-Abeta/tg2576_m287_4G8_s147_resize_Object Predictions.png]

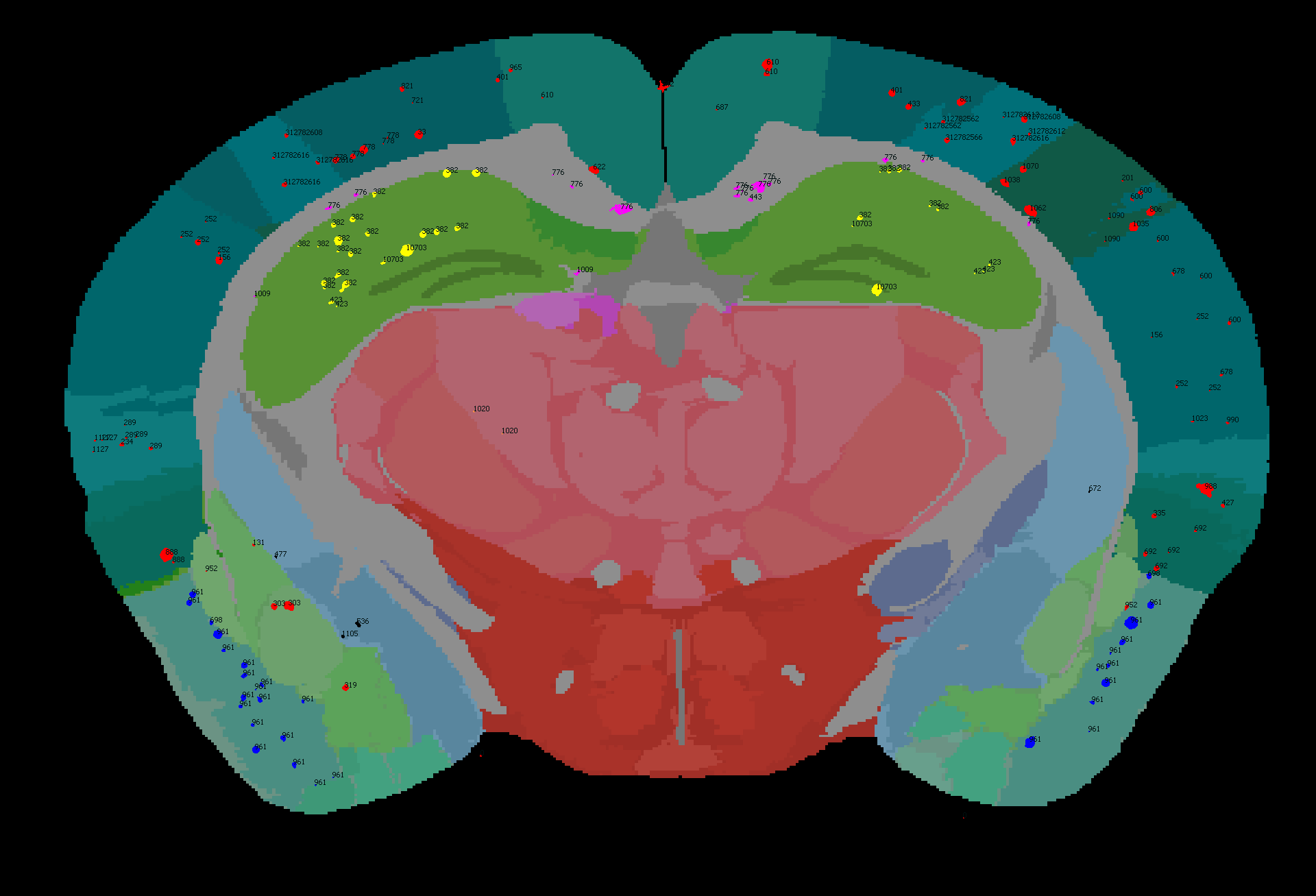

Supplement: Supplementary file 2 [file Data_Sheet_1.ZIP › Supplementary_material_Yates/pan-Abeta/tg2576_m287_4G8_s151_resize_Object Predictions.png]

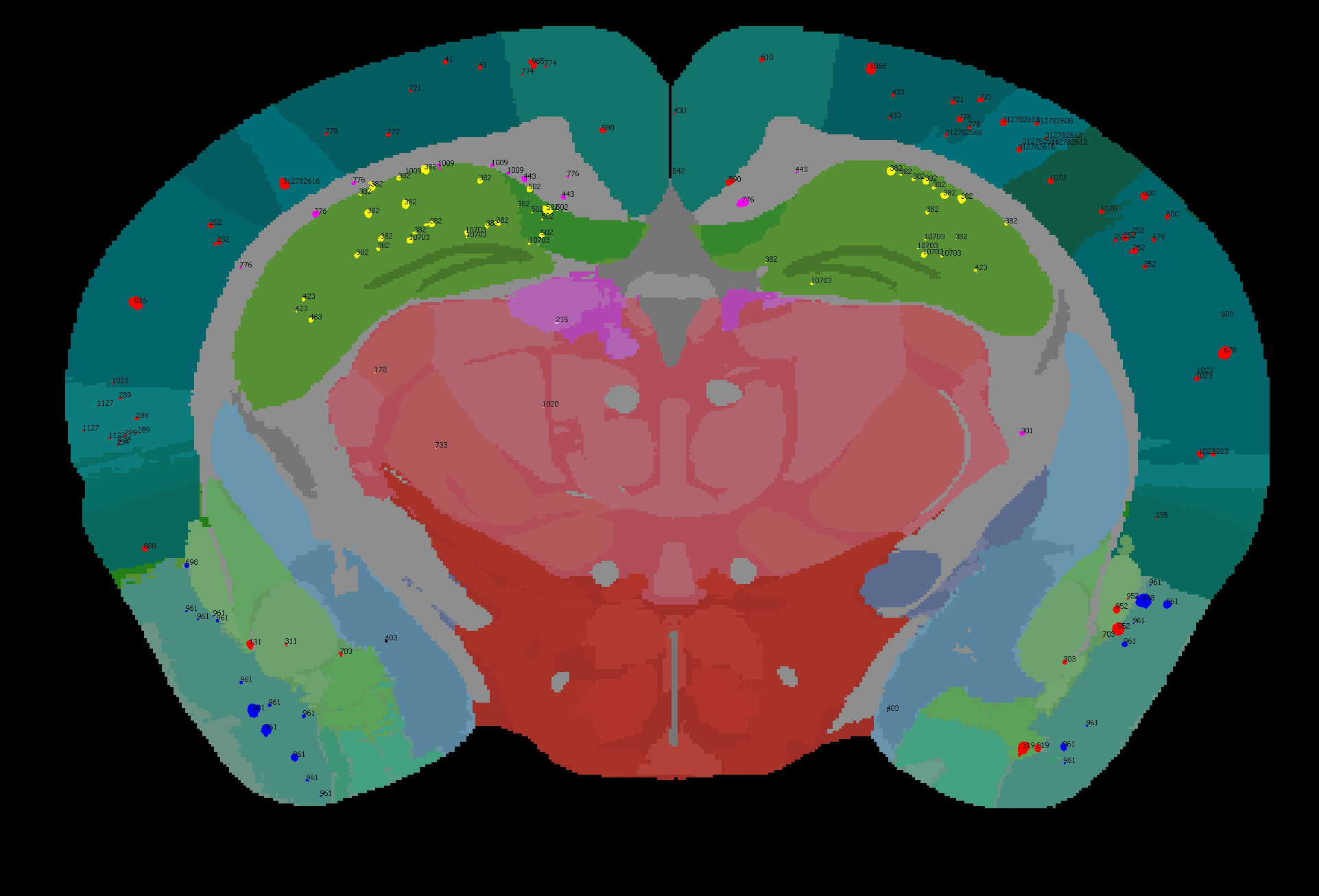

Supplement: Supplementary file 2 [file Data_Sheet_1.ZIP › Supplementary_material_Yates/pan-Abeta/tg2576_m287_4G8_s155_resize_Object Predictions.png]

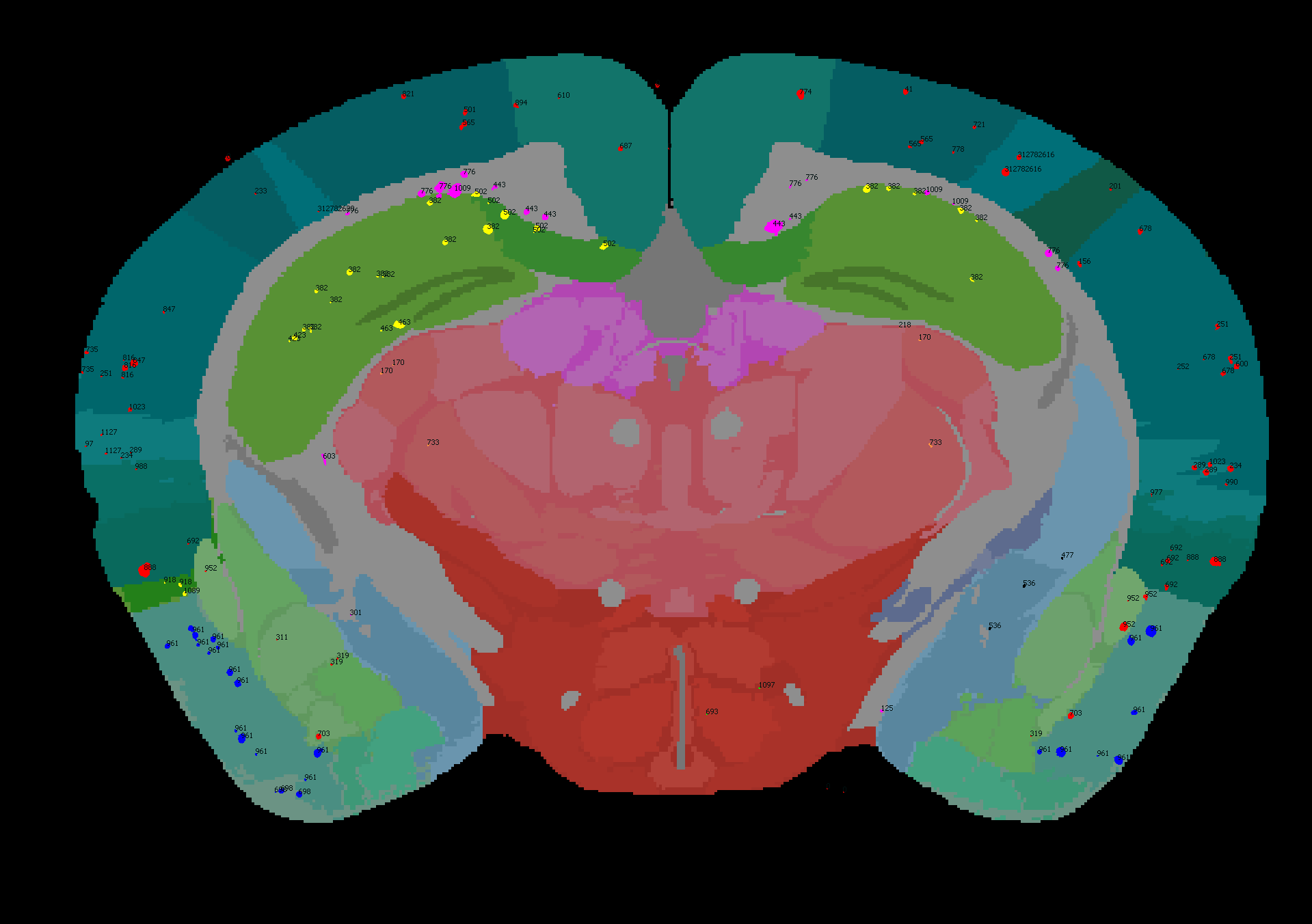

Supplement: Supplementary file 2 [file Data_Sheet_1.ZIP › Supplementary_material_Yates/pan-Abeta/tg2576_m287_4G8_s159_resize_Object Predictions.png]

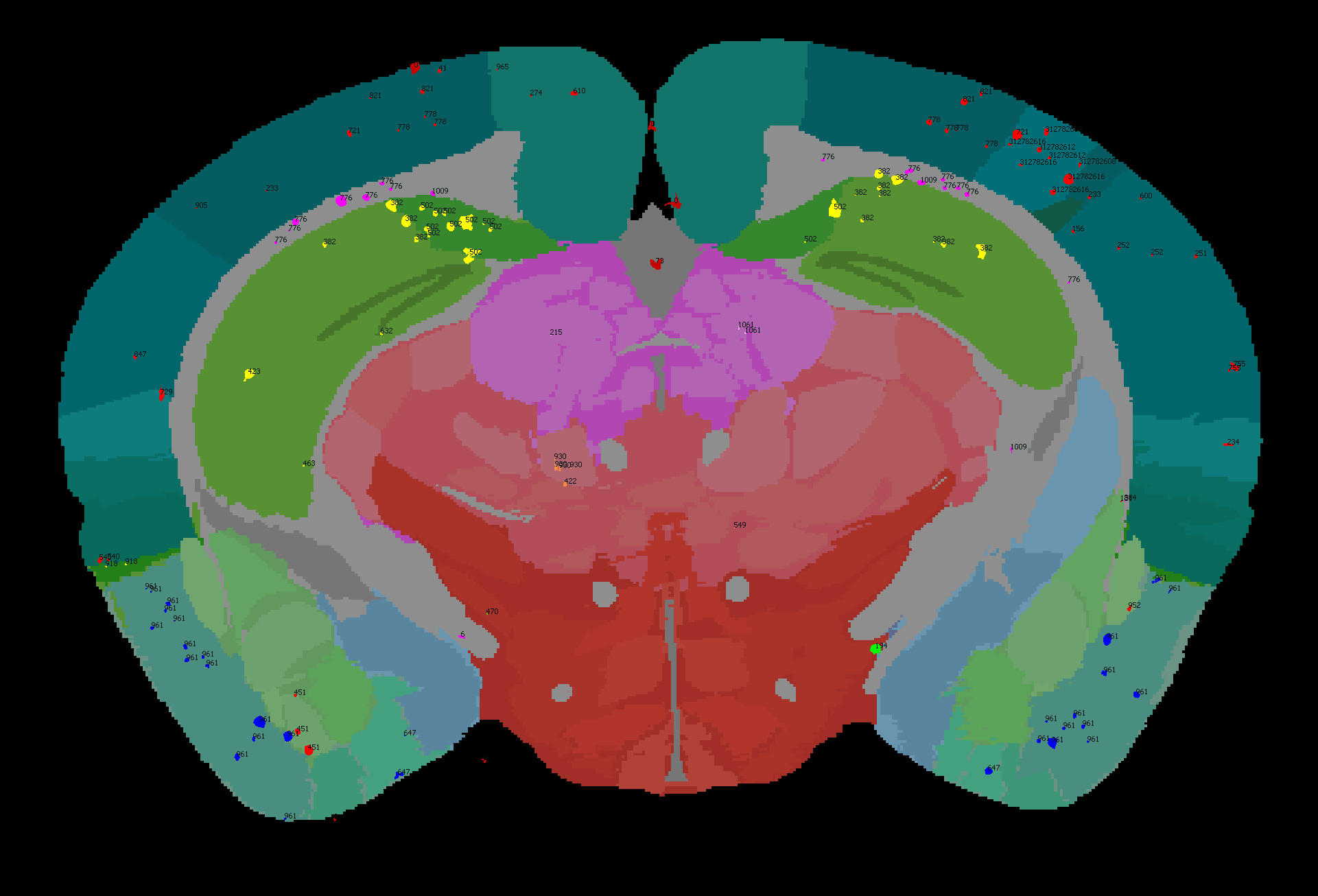

Supplement: Supplementary file 2 [file Data_Sheet_1.ZIP › Supplementary_material_Yates/pan-Abeta/tg2576_m287_4G8_s167_resize_Object Predictions.png]
